# Diversification of Pharmaceuticals via Late-Stage Hydrazination

**DOI:** 10.1021/acscatal.5c03458

**Published:** 2025-07-29

**Authors:** Tongliang Zhou, Chaoyue Zhao, Shiyi Yang, Elwira Bisz, Błażej Dziuk, Roger Lalancette, Roman Szostak, Xin Hong, Michal Szostak

**Affiliations:** † Department of Chemistry, 67206 Rutgers University, 73 Warren Street, Newark, New Jersey 07102, United States; ‡ Center of Chemistry for Frontier Technologies, Department of Chemistry, State Key Laboratory of Clean Energy Utilization, Zhejiang University, Hangzhou 310027, P.R. China; School of Chemistry and Chemical Engineering, Henan Normal University, Xinxiang 453007, P.R. China; § Department of Chemistry, 49576 Opole University, 48 Oleska Street, Opole 45-052, Poland; ∥ Department of Chemistry, University of Science and Technology, Norwida 4/6, Wroclaw 50-373, Poland; ⊥ Department of Chemistry, 535248 Wroclaw University, F. Joliot-Curie 14, Wroclaw 50-383, Poland; # Ningbo Key Laboratory of Agricultural Germplasm Resources Mining and Environmental Regulation, College of Science and Technology, Ningbo University, Ningbo 315300, China

**Keywords:** N-heterocyclic carbene, imidazo[1,5-*a*]pyridin-3-ylidene, precatalyst, hydrazine, late-stage functionalization

## Abstract

Developments in organic synthesis over the past century have greatly enabled the discovery of life-saving medicines. In this context, over the past two decades, palladium-catalyzed cross-coupling reactions have transformed the exploration of emerging therapeutics. However, the cross-coupling between aryl halides and hydrazine, NH_2_NH_2_, the smallest bis-nitrogen nucleophile, has been a long-standing challenge due to the reducing capacity of hydrazine and the presence of multiple N–H bonds. These advances have significantly lagged behind modern cross-coupling technologies despite the broad utility of arylhydrazines to serve as a springboard for the discovery of innovative medicines. Herein, we report a general platform for the diversification of pharmaceuticals by late-stage hydrazination. By designing biaryl, sterically demanding biaryl and flexible N-heterocyclic carbene ligands with strong σ-donation and controlled architecture of the catalytic pocket, we have established selective palladium-catalyzed cross-coupling of aryl halides with hydrazine to give highly valuable arylhydrazines. By using this method, we have achieved direct cross-coupling of a variety of complex pharmaceuticals covering various metabolic diseases ranging from life-changing anticancer to blockbuster antiallergic drugs to give broadly useful arylhydrazines that can be converted *in situ* into heterocyclic frameworks. The developed class of ligands shows notably high %V_bur_, while retaining the full flexibility of the catalytic pocket. In this catalysis approach, a remarkably broad range of aryl chlorides and aryl bromides can be systematically applied as cross-coupling partners using mild carbonate bases. The developed ligands feature biaryl-controlled steric environment of the catalytic pocket in combination with strong σ-donicity, which facilitates and integrates individual elementary steps of the catalytic cycle, such as oxidative addition, reductive elimination from Pd center, as well as protection of Pd­(II) intermediate from overreduction. Extensive computational studies have been conducted to gain insight into the mechanism of the coupling and elucidate the key role of biaryl and sterically flexible N-heterocyclic carbene ligands. The presented reactivity establishes a powerful entry into the late-stage cross-coupling with challenging nucleophiles for drug discovery and development.

## Graphical abstract

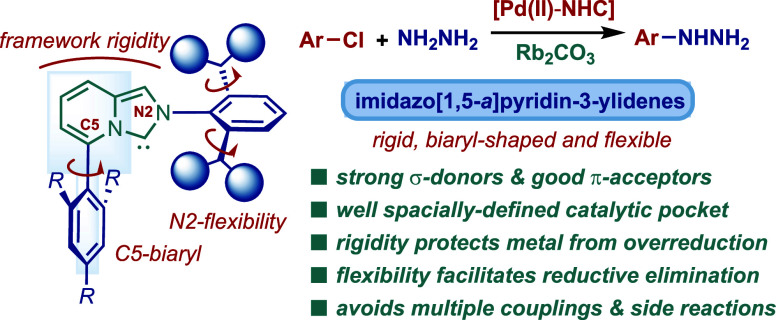

## Introduction

Breakthroughs in organic synthesis over the past decades have revolutionized modern drug discovery.^1,2^ From the discovery of dyes and metal-catalyzed asymmetric hydrogenations to cross-coupling reactions, these processes have had a transformative impact on innovations in drug development.^3–5^ In particular, recent advances in transition-metal-catalysis have led to the invention of powerful processes that enable rapid identification of new privileged scaffolds by late-stage modification of existing drug molecules and conduct comprehensive structure–activity relationship explorations, thus driving the discovery of new medicines (Figure 1A).^6–8^ In this context, the reactions that embed privileged motifs, such as amines, and enable rapid molecular diversification have been considered particularly valuable.^9–13^

**Figure 1:**
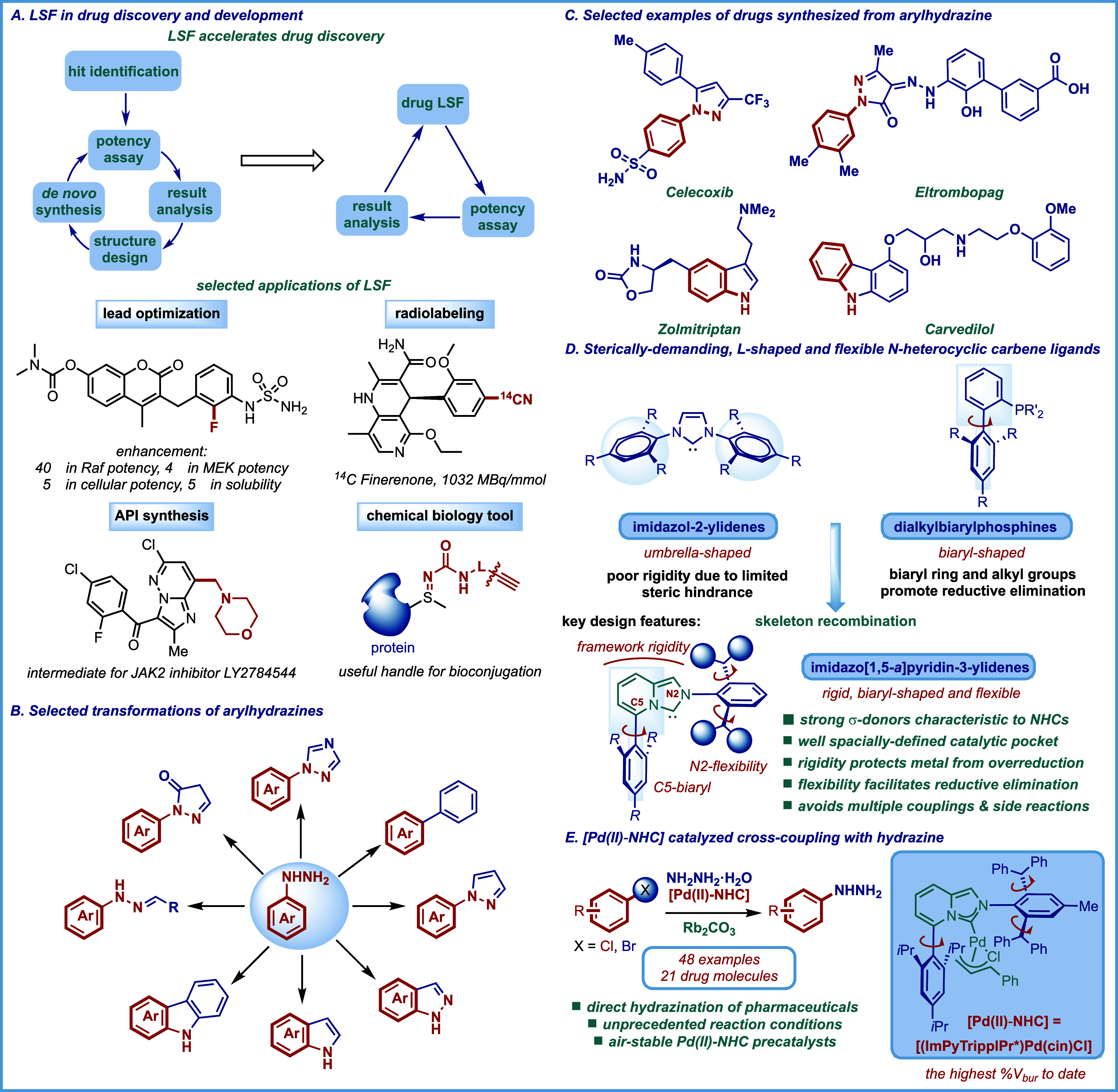
(A) Rise of LSF in drug discovery and development. (B) Synthetically valuable transformations involving arylhydrazines. (C) Examples of pharmaceuticals that feature heterocycles derived from arylhydrazine. (D) Sterically demanding, biaryl-restricted, and N-wingtip flexible N-heterocyclic carbene ligands: promoting elementary steps by combining ligand restriction with flexibility. (E) [(ImPyTrippIPr*)­Pd­(cin)­Cl]-catalyzed cross-coupling with hydrazine described in this report.

For decades, arylhydrazines have been recognized as fundamental intermediates in drug discovery.^14,15^ Most crucially, arylhydrazines have been routinely used for the construction of valuable nitrogen heterocycles to unlock new molecular scaffolds.^16–18^ Heterocycles, such as pyrazoles, indoles, carbazoles, triazoles, indazoles, and β-lactams, are constructed from arylhydrazines as privileged structural motifs in a wide array of biologically active molecules **(** Figure 1 **B,C).** ^19,20^ However, the development of chemoselective methods for the synthesis of arylhydrazines from widely available and highly functionalized starting materials has been a long-standing synthetic challenge. Traditional methods for the synthesis of arylhydrazines have relied on nucleophilic aromatic substitution of haloarenes or diazotization of anilines.^21,22^ However, these methods are limited to electron-deficient and specifically substituted substrates or pose explosion hazards due to the generation of unstable diazonium intermediates, and are restricted by the properties of starting materials.^23,24^ In the last two decades, tremendous advances have been made in palladium-catalyzed cross-coupling reactions of amines.^25,26^ At present, palladium-catalyzed aminations are a “go-to” method for the synthesis of new pharmacophores in the pharmaceutical industry to access new active molecules and address the needs of pharmaceutical development.^27–32^ However, the cross-coupling between aryl halides and hydrazine, NH_2_NH_2_, the smallest bis-nitrogen nucleophile, has been a long-standing challenge due to the reducing capacity of hydrazine and the presence of multiple N–H bonds.^33^ The advances in hydrazine cross-coupling have significantly lagged modern cross-coupling reactions despite the broad utility of arylhydrazines to serve as a springboard for the discovery of innovative medicines.^13–20^ At present, highly chemoselective cross-coupling with hydrazine has been extremely difficult to achieve despite representing a crucial goal for therapeutic applications owing to the diverse applicability of arylhydrazines.

There are several key challenges that must be addressed for the development of broadly applicable cross-coupling of hydrazines: (1) hydrazine is a strong reductant, resulting in the reduction of Pd­(II) to inactive Pd black;^34^ (2) the presence of multiple N–H bonds means that multiple coupling products are typically obtained;^35^ (3) the cleavage of N–N bonds in arylhydrazines in the presence of metals is facile;^36^ and (4) reductive elimination of hydrazine from the metal center is challenging using the vast majority of common ligands due to small size of hydrazine, disfavoring this elementary step.^25,26^

Considering the utility of arylhydrazines, efforts have been made toward the development of palladium-catalyzed cross-coupling of aryl halides with hydrazine. Cross-coupling using phosphine ligands has been developed;^37–39^ however, these methods have poor functional group tolerance due to the necessity for strong *tert*-butoxide bases. A catalytic system using hydroxides has been developed; however, this system suffers from narrow substrate scope and many substrates necessitate *tert*-butoxide bases.^40^ At present, rapid, broadly applicable, and general methods for cross-coupling of aryl halides with hydrazine that would meet the demands of late-stage functionalization of pharmaceuticals in pursuit of novel, more selective, and more active therapeutic agents remain an unmet challenge for this synthetically valuable transformation.

We anticipated that the development of chemoselective cross-coupling of hydrazine with aryl halides necessitates the departure from traditional ligand scaffolds toward new ligand architectures. As part of our program in catalysis,^41–44^ we designed new biaryl, sterically demanding, L-shaped and flexible N-heterocyclic carbene ligands with strong σ-donation and controlled architecture of the catalytic pocket. By using this system, we have established an unprecedented method for selective palladium-catalyzed cross-coupling of aryl halides and hydrazine using mild base to give highly valuable arylhydrazines and achieved direct cross-coupling of >20 complex pharmaceuticals covering various metabolic diseases (Figure 1D,E). The developed ligands feature controlled steric environment of the catalytic pocket in combination with strong σ-donicity and notably high %*V*_bur_, which facilitate and integrate individual elementary steps of the catalytic cycle and protect Pd­(II) intermediate from overreduction.^45,46^ The presented reactivity platform establishes a powerful entry into cross-coupling with challenging nucleophiles for drug discovery and development.

## Methods

### Reaction Development

4-Chlorotoluene and hydrazine hydrate were selected as model substrates (Table 1). The *in situ* protocol, in which a palladium precursor and base are premixed prior to addition of substrates, was used for initial ligand screening. Sodium *tert*-butoxide was selected as a base for initial ligand comparison.^37–39^ In line with our hypothesis, standard imidazol-2-ylidene ligands, IMes and IPr, gave little or no coupling under the reaction conditions (entries 1–2), indicating that traditional NHC ligands are not suitable for this challenging coupling. To our delight, sterically defined imidazo­[1,5-*a*]­pyridin-3-ylidene ImPyMesMes gave 40% yield of the desired monoarylated product **2a** (entry 3), indicating that biaryl L-shaped ImPy (ImPy = imidazo­[1,5-*a*]­pyridin-3-ylidene) ligand architecture might be conducive to hydrazine coupling.^47–49^ Both the yield and arylation selectivity were improved as we increased the steric rigidity of the ligand at C5 and N2 positions in the order of ImPyTrippDipp > ImPyTrippMes > ImPyMesDipp > ImPyMesMes (entries 3–6). The most structurally rigid ImPyTrippDipp afforded 79% of the cross-coupling product (entry 6).

**Table 1:**
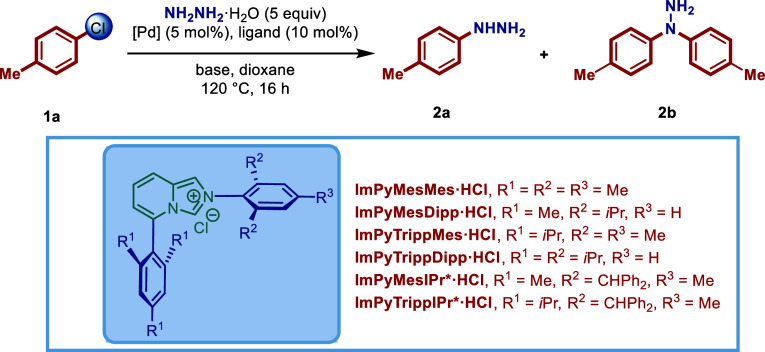
Optimization of the Reaction Conditions^a^

entry	[Pd]/ligand	base	conversion of **1a** (%)^b^	yield of **2a** (%)^b^	ratio (**2a**/**2b**)^b^
1	[Pd(cin)Cl]_2_/ IMes·HCl	NaO*t*Bu	34	0	–^c^
2	[Pd(cin)Cl]_2_/ IPr·HCl	NaO*t*Bu	63	16	2.1/1
3	[Pd(cin)Cl]_2_/ ImPyMesMes·HCl	NaO*t*Bu	>99	40	6.7/1
4	[Pd(cin)Cl]_2_/ ImPyMesDipp·HCl	NaO*t*Bu	>99	67	9/1
5	[Pd(cin)Cl]_2_/ ImPyTrippMes·HCl	NaO*t*Bu	>99	73	10.8/1
6	[Pd(cin)Cl]_2_/ImPyTrippDipp·HCl	NaO*t*Bu	>99	79	27.6/1
7	[Pd(cin)Cl]_2_/ ImPyMesIPr*·HCl	NaO*t*Bu	>99	90	19/1
8	[Pd(cin)Cl]_2_/ ImPyTrippIPr*·HCl	NaO*t*Bu	>99	91	21.2/1
9	[(ImPyTrippIPr*)Pd(cin)Cl]	NaO*t*Bu	>99	79	65.7/1
10^d^	[(ImPyTrippIPr*)Pd(cin)Cl]	KOH	>99	90	49/1
11^d^	[(ImPyTrippIPr*)Pd(cin)Cl]	K_3_PO_4_	>99	79	32.2/1
12^d^	[(ImPyTrippIPr*)Pd(cin)Cl]	Na_3_PO_4_	>99	31	65.7/1
13^d^	[(ImPyTrippIPr*)Pd(cin)Cl]	KF	59	18	–^c^
14^d^	[(ImPyTrippIPr*)Pd(cin)Cl]	KOAc	63	11	–^c^
15^d^	[(ImPyTrippIPr*)Pd(cin)Cl]	K_2_CO_3_	>99	71	14.4/1
16^d^	[(ImPyTrippIPr*)Pd(cin)Cl]	Cs_2_CO_3_	>99	85	27.6/1
17^d^	[(ImPyTrippIPr*)Pd(cin)Cl]	Rb_2_CO_3_	>99	90	49/1
18^d^ ^,^ ^e^	[(ImPyTrippIPr*)Pd(cin)Cl]	Rb_2_CO_3_	>99	94	32.3/1

a Conditions: 4-chlorotoluene (0.2 mmol, 1.0 equiv), NH_2_NH_2_·H_2_O (5 equiv), [Pd­(cin)­Cl]_2_ (2.5 mol %)/ligand (10 mol %) or [(ImPyTripIPr*)­Pd­(cin)­Cl] (5 mol %), base (1.5 equiv), dioxane (0.10 M), 120 °C, 16 h.

b Determined by ^1^H NMR.

c **2b** < 2%.

d 2 equiv of base.

e 3 equiv of base.

f Dioxane (0.20 M).

At this point, since we were unable to further optimize the reaction conditions using known ImPy ligands, we reasoned that an improvement in catalytic reactivity could be achieved by more sterically demanding ligands that simultaneously accommodate flexibility at the catalytic pocket. Thus, sterically demanding, L-shaped and flexible ImPy ligands ImPyMesIPr* and ImPyTrippIPr* were synthesized, in which the traditional N2 substituent in imidazo­[1,5-*a*]­pyridin-3-ylidene framework was replaced by a flexible 2,6-dibenzhydryl-4-methylphenyl wingtip,^50^ while keeping the rigid biaryl substitution at the C5 carbon. In this ligand architecture (1), the steric C5 encumbrance around the metal center stabilizes the ArPd­(II)­X intermediate and protects it from overreduction by hydrazine; (2) reductive elimination is accelerated by the C5 steric bulk of the ligand, after hydrazine coordination and deprotonation enabled by flexibility at N2; (3) the merger of N2 flexible and C5 hindered environment suppresses further arylation of the monoarylated product and other side reactions.

To our delight, ImPyMesIPr* delivered the desired coupling product in a 90% yield (entry 7). Further improvement in reactivity was observed using the more encumbered ImPyTrippIPr*, which produced the desired coupling product in 91% yield (entry 8). It is worth noting that biaryl sterically demanding, C5-rigid and N2-flexible ligands ImPyMesIPr* and ImPyTrippIPr* feature an electron-rich catalyst pocket resulting from the biaryl-C5 arrangement and rotatable N2-substitution. This permits facilitating substrate coordination and reductive elimination steps, while maintaining strong σ-donation characteristic to NHC ligands (*vide infra*).

### Precatalyst Development

Having identified ImPyTrippIPr* as the most efficient ligand, our next goal was to perform this challenging cross-coupling with a weak base. The use of weak bases enables synthetically preferred, well-defined Pd­(II)–NHC precatalysts with a 1:1 ratio of Pd to ligand, while achieving functional group tolerance unavailable with current phosphine-based methods.^51^ Notably, the use of weak bases is critical for the development of general late-stage functionalization protocols for broad application in drug discovery.^3–8^

Thus, [(ImPyTrippIPr*)­Pd­(cin)­Cl] featuring cinnamyl as an ancillary ligand was synthesized smoothly from the ImPy salt and [Pd­(cin)­Cl]_2_ in an 83% yield. It is notable that [(ImPyTrippIPr*)­Pd­(cin)­Cl] is an air- and moisture-stable solid, with no decomposition observed after storing for 3 months on benchtop. The complex was fully characterized by X-ray crystallography (Figure 2). The X-ray crystallographic analysis revealed the C–Pd, Pd–Cl, and Pd–C­(Ph) bond lengths of 2.024, 2.378, and 2.241 Å, which are in the range for Pd­(II)-allyl type complexes ([(IPr)­Pd­(cin)­Cl], C–Pd, 2.040 Å; Pd–Cl, 2.348 Å; Pd–C­(Ph), 2.280 Å).^51^ The topographical steric map showed (%*V*_bur_) of 46.2% with 61.8, 41.2, 50.0, 32.0% for each quadrant using the approach established by Cavallo.^52^ Interestingly, the quadrants corresponding to the C5 aryl group are more crowded than the quadrants corresponding to the N2 wingtips, even though the Tripp (Tripp = 2,4,6-triisopropylphenyl) substituent at the C5 position is less sterically demanding than the 2,6-dibenzhydryl-4-methylbenzyl substituent at the N2 position. For comparison, the classic imidazol-2-ylidene [(IPr)­Pd­(cin)­Cl] features a significantly smaller (%*V*_bur_) of 34.3%.^51^ Thus, it is evident that the catalytic pocket of ImPyTrippIPr* is characterized by two simultaneous effects: (1) steric shielding at the C5 position and (2) flexible twisting at the N2 position, which together furnish a unique spatial pocket around the metal center, arising from C-biaryl-restricted and N-wingtip-flexible ligand topology.

**Figure 2:**
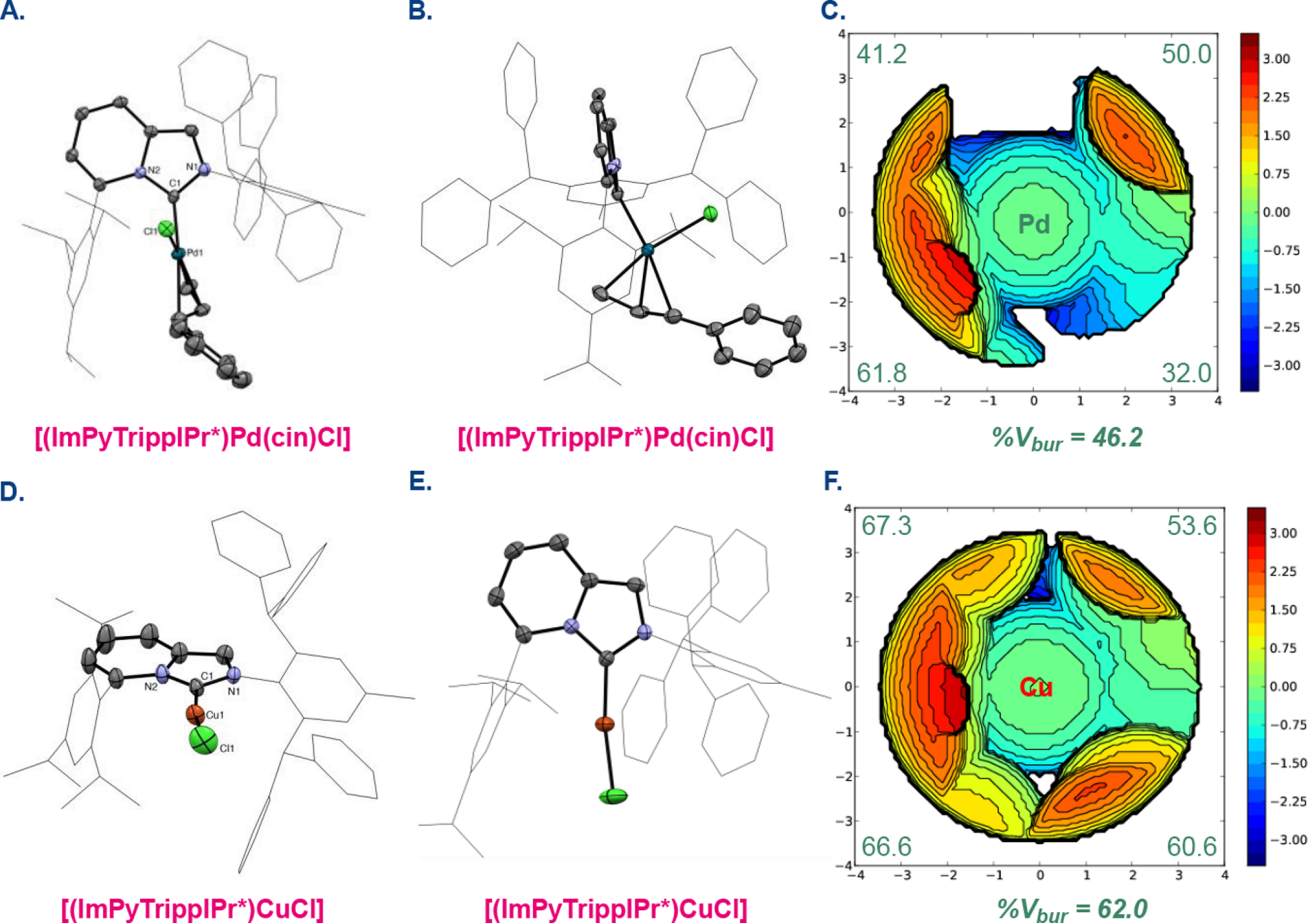
(A,B) X-ray structure of [(ImPyTrippIPr*)­Pd­(cin)­Cl], front and side view. (C) Topographical steric map of [(ImPyTrippIPr*)­Pd­(cin)­Cl] showing %*V*_bur_ per quadrant. CCDC 2271723. (D,E) X-ray structure of [(ImPyTrippIPr*)­CuCl], side and front view. (F) Topographical steric map of [(ImPyTrippIPr*)­CuCl] showing %*V*_bur_ per quadrant. CCDC 2271724. See SI for the selected bond lengths and angles.

With this precatalyst in hand, we found that a variety of weak bases can be used for selective cross-coupling with hydrazine (Table 1, entries 9–15). Most remarkably, even weak bases, such as potassium phosphate or potassium carbonate, were identified as suitable bases. To our knowledge, this is the first example of weak bases affording high yield in hydrazine cross-coupling.^37^ This finding also shows that strong bases are not required for high selectivity.^40^ Other bases such as Na_3_PO_4_ and K_2_CO_3_ gave promising results for future studies (entries 13–15). Carbonate bases showed the highest reactivity (entries 15–17). The optimal base identified was more soluble rubidium carbonate, which produced the cross-coupling product in 90% yield (entry 17). The yield was further improved by adjusting the reaction concentration (entry 18). It should be noted that this cross-coupling is highly practical: (1) the catalyst, [(ImPyTrippIPr*)­Pd­(cin)­Cl], is air- and moisture-stable, (2) the reactions are performed on the benchtop, and (3) the mild reaction conditions provide opportunity for wide functional group tolerance, which is essential for the late-stage functionalization of complex pharmaceuticals and applications in drug discovery contexts. It is also interesting to note that *in situ* catalyst provides better yield although lower selectivity than those of the preformed precatalyst (entries 8–9). This trend in reactivity is often observed in Pd-NHC catalysis.^45^ Excess ligand could recoordinate to deactivated unligated off-cycle Pd(0) before inactive palladium black is formed, regenerating on-cycle NHC monoligated Pd(0).^45b^ It is also possible that mixed NHC-Pd-aNHC complexes are formed, which have a beneficial effect on certain reactions.^45c^

### Substrate Scope

With the optimized conditions in hand, we next investigated the scope of cross-coupling with hydrazine (Table 2). The products were converted *in situ* to 3,5-dimethyl-N-arylpyrazoles using acetylacetone in the presence of trifluoroacetic acid to facilitate isolation and demonstrate direct synthesis of valuable nitrogen heterocycles directly from arylhydrazines.^39,40^ As shown, a broad array of aryl chlorides can be used as substrates to produce cross-coupling products in good to excellent yields. Aryl chlorides decorated with various electron-neutral (**3a**–**3c**), electron-donating (**3d**), and electron-withdrawing (**3e**, **3f**) groups afforded the hydrazination products in high yields. Notably, the reaction is not sensitive to steric hindrance, as 2-methyl (**3c**) was readily tolerated. Most notably, substrates with a broad palette of sensitive functional groups are tolerated, including nitriles (**3f**), tertiary amides (**3h**), secondary amides (**3i**, **3j**), sulfonamides (**3k**), and esters (**3m**, **3p**). Furthermore, medicinally relevant heterocycles, including pyridines (**3l**–**3n**), quinolines (**3o**), and indoles (**3p**) are competent substrates, delivering valuable N-hydrazinated products in good to high yields. Since aryl bromides are often used as practical electrophiles in drug development,^53^ we expanded the scope of the reaction to aryl bromides as alternative coupling partners. Notably, we established that aryl bromides can be utilized without modification of the reaction conditions with excellent functional group tolerance and broad compatibility with electron-withdrawing (**3q**–**3r**, **3t**–**3u**, **3x**), electron-rich (**3s**, **3w**), and electron-neutral (**3v**, **3y**–**3z**) substrates. Importantly, electrophilic functional groups, such as esters (**3t**–**3u**, **3aa**), sulfides (**3w**), and sulfoxides (**3x**) are very compatible under these conditions, providing handles for further functionalization.

**Table 2:**
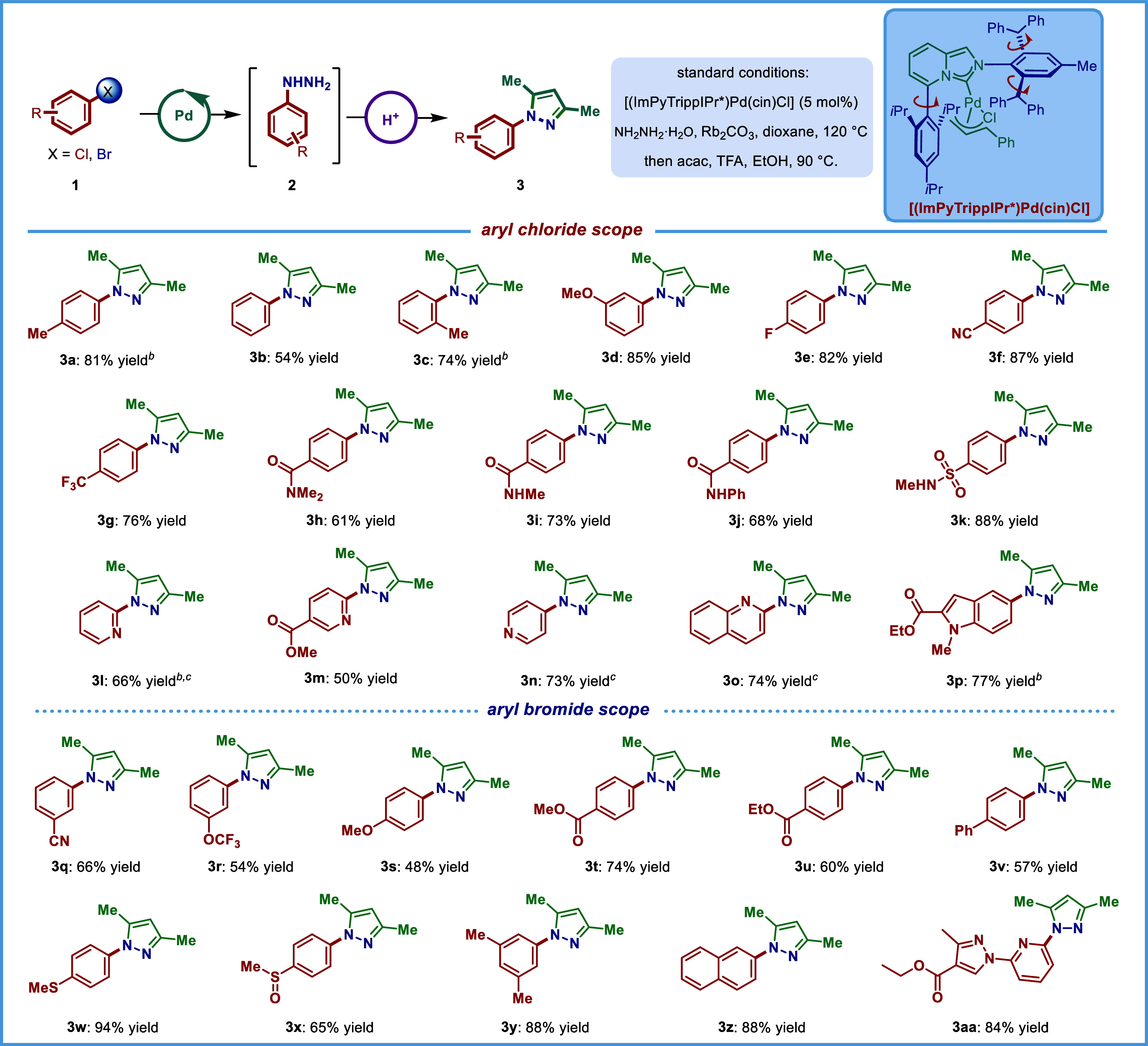
Substrate Scope^a^

a Conditions: **1** (0.2 mmol, 1.0 equiv), NH_2_NH_2_·H_2_O (5 equiv), [(ImPyTrippIPr*)­Pd­(cin)­Cl] (5 mol %), Rb_2_CO_3_ (3 equiv), dioxane (0.20 M), 120 °C, 16 h. Isolated yields.

b w/o TFA.

c <10% yield w/o [Pd].

### Cross-Coupling of Pharmaceuticals

Encouraged by the superb functional group tolerance and generality of the cross-coupling, we moved to the main goal of our study, namely, to the application in late-stage functionalization of complex pharmaceuticals (Table 3). The cross-coupling of pharmaceuticals with hydrazine is significant from the standpoint of functionalization of drug molecules with bis-nitrogen nucleophile that has been routinely exploited for installation of medicinally privileged heterocycles.^13–20^ The present method is notable in that it can be used to selectively install hydrazine in a plethora of drug molecules that feature a broad variety of substitution patterns and sensitive functional groups used across various metabolic diseases for drug discovery. As such, the lipid-lowering agents Fenofibrate (**1ab**) and Clofibrate (**1ac**) reacted with hydrazine in high yields. A diverse range of drugs for the treatment of mental disorders, such as Chlorpromazine (**1ad**), Perphenazine (**1ae**), Haloperidol (**1af**), Penfluridol (**1ag**), Loxapine (**1ah**), Amoxapine (**1ai**), and Chlorprothixene (**1aj**), are excellent substrates, affording the corresponding products in 71–93% yields. Furthermore, (**1ak**) derived from antidiabetic Repaglinide, containing multiple amide groups, is well-compatible. Furthermore, blockbuster antihistamine drugs such as Desloratadine (**1al**) and Loratadine (**1am**) are suitable substrates, delivering the coupling products in excellent yields. Moreover, our protocol is also applicable to amides derived from antigout Probenecid (**1an**) and Febuxostat (**1ao**), antibacterial Flumequine (**1ap**), and anti-inflammatory Isoxepac (**1aq**). Furthermore, selective COX-2 inhibitor Etoricoxib (**1ar**), antimalarial Chloroquine (**1as**), EGFR inhibitor Gefitinib (**1at**), PDE5 inhibitor Avanafil (**1au**), and Tadalafil derivative (**1av**) are amenable to the hydrazination protocol with high efficiency.

**Table 3:**
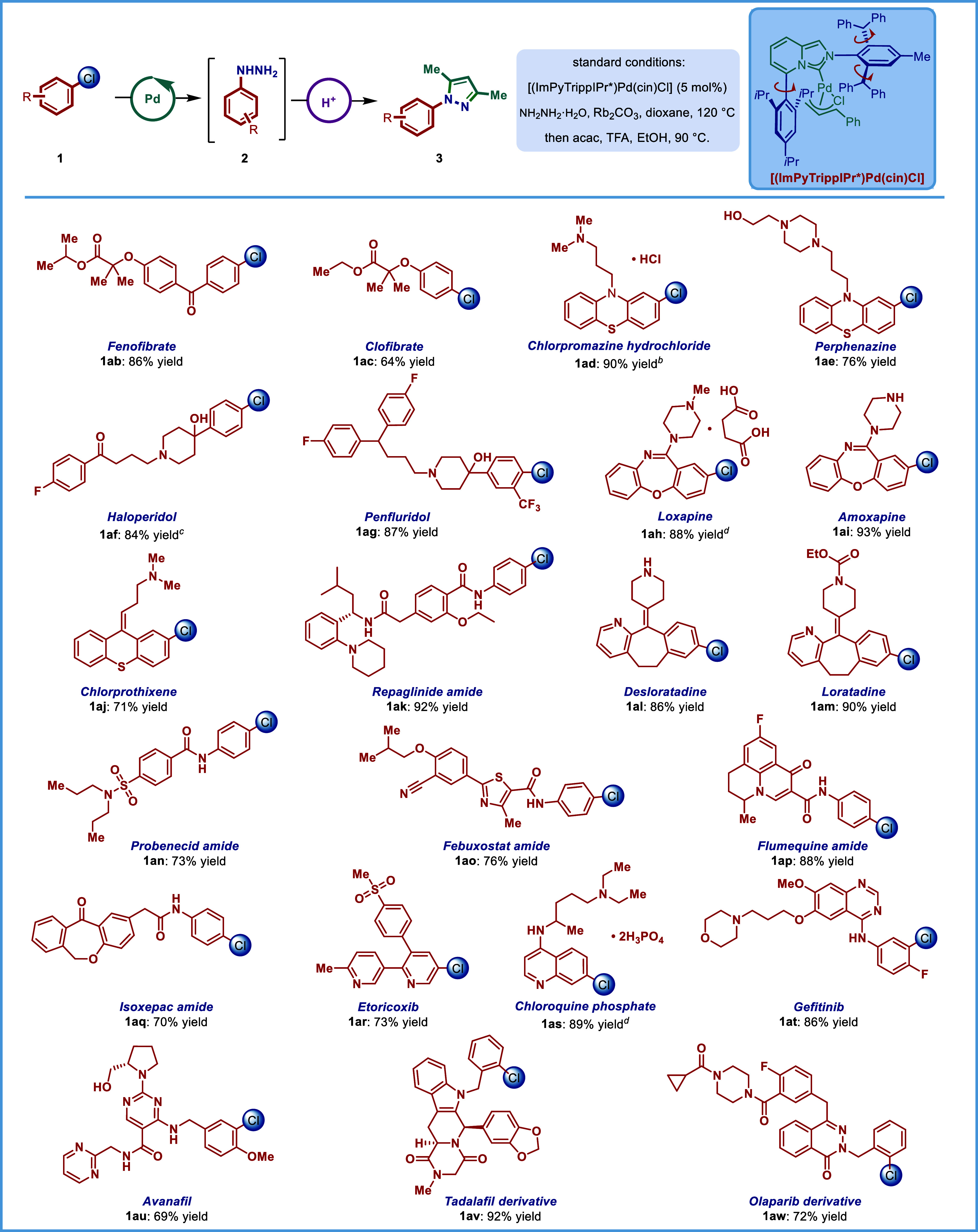
Late-Stage Hydrazination of Pharmaceuticals^a^

a Conditions: **1** (0.2 mmol, 1.0 equiv), NH_2_NH_2_·H_2_O (5 equiv), [(ImPyTrippIPr*)­Pd­(cin)­Cl] (5 mol %), Rb_2_CO_3_ (3 equiv), dioxane (0.20 M), 120 °C, 16 h. Isolated yields.

b Rb_2_CO_3_ (4 equiv).

c NH_2_NH_2_·H_2_O (2 equiv).

d Rb_2_CO_3_ (5 equiv).

Finally, (**1aw**) obtained from PARP inhibitor Olaparib featuring a competitive S_N_Ar site provided the desired product in 72% yield. As illustrated by the examples in Table 3, this methodology can accommodate a broad range of functional groups in complex pharmaceuticals, such as esters (**1ab**–**1ac**), ketones (**1ab**, **1af**), primary alcohols (**1ae**, **1au**), tertiary alcohols (**1af**–**1ag**), alkenes (**1aj**, **1al**, **1am**), ethers (**1ab**–**1ac**), amides (**1ak**, **1am**–**1aq**, **1au**–**1av**), nitriles (**1ao**), secondary amines (**1ai**, **1al**, **1as**–**1au**) and sulfonyl groups (**1ar**). Furthermore, a broad range of privileged heterocycles from the drug discovery standpoint, including phenothiazines (**1ad**–**1ae**), dibenzoxazepines (**1ah**–**1ai**), thioxanthenes (**1aj**), benzo­[5,6]­cyclohepta­[1,2-*b*]­pyridines (**1al**–**1am**), thiazoles (**1ao**), quinolin-4-ones (**1ap**), doxepinones (**1aq**), pyridines (**1ar**), quinolines (**1as**), quinazolines (**1at**), pyrimidines (**1au**), indoles (**1av**), piperazine-2,5-diones (**1av**) and phthalazin-1­(2H)-ones (**1aw**) are perfectly compatible, highlighting the generality of the coupling and its significant potential to make an impact on drug discovery.

It is worth pointing out that a long-standing challenge in the field is that complex substrates possessing functional groups hinder catalyst turnover or engage in unproductive side reactions, particularly in complex synthetic drugs.^3,33^ This challenge has been previously addressed only with stoichiometric palladium.^10^ All Pd-phosphine-catalyzed Buchwald-Hartwig hydrazination methods developed so far can only be applied to simple model substrates. To demonstrate this point, we compared our system with a Pd-phosphine method.^40^

To align with our conditions, all of the reactions were carried out with 5 mol% palladium. We found that the Pd–phosphine system can tolerate acidic protons in sulfonamides but cannot accommodate ester groups, as substrates **1p**, **1t**, **1aa**, **1ab**, and **1ac** gave negligible yields (Table 4A). Notably, although the Pd–phosphine system delivered the desired products for chlorpromazine **1ad**, desloratadine **1al**, and perphenazine **1ae**, the yields were significantly lower. Moreover, no product formation was observed for drugs with more complex structures such as amoxapine **1ai**, isoxepac amide **1aq**, and gefitinib **1at**. Overall, the results indicate that the catalytic system developed represents a novel approach for C–N coupling of complex molecules that cannot be replaced by other methods.

**Table 4:**
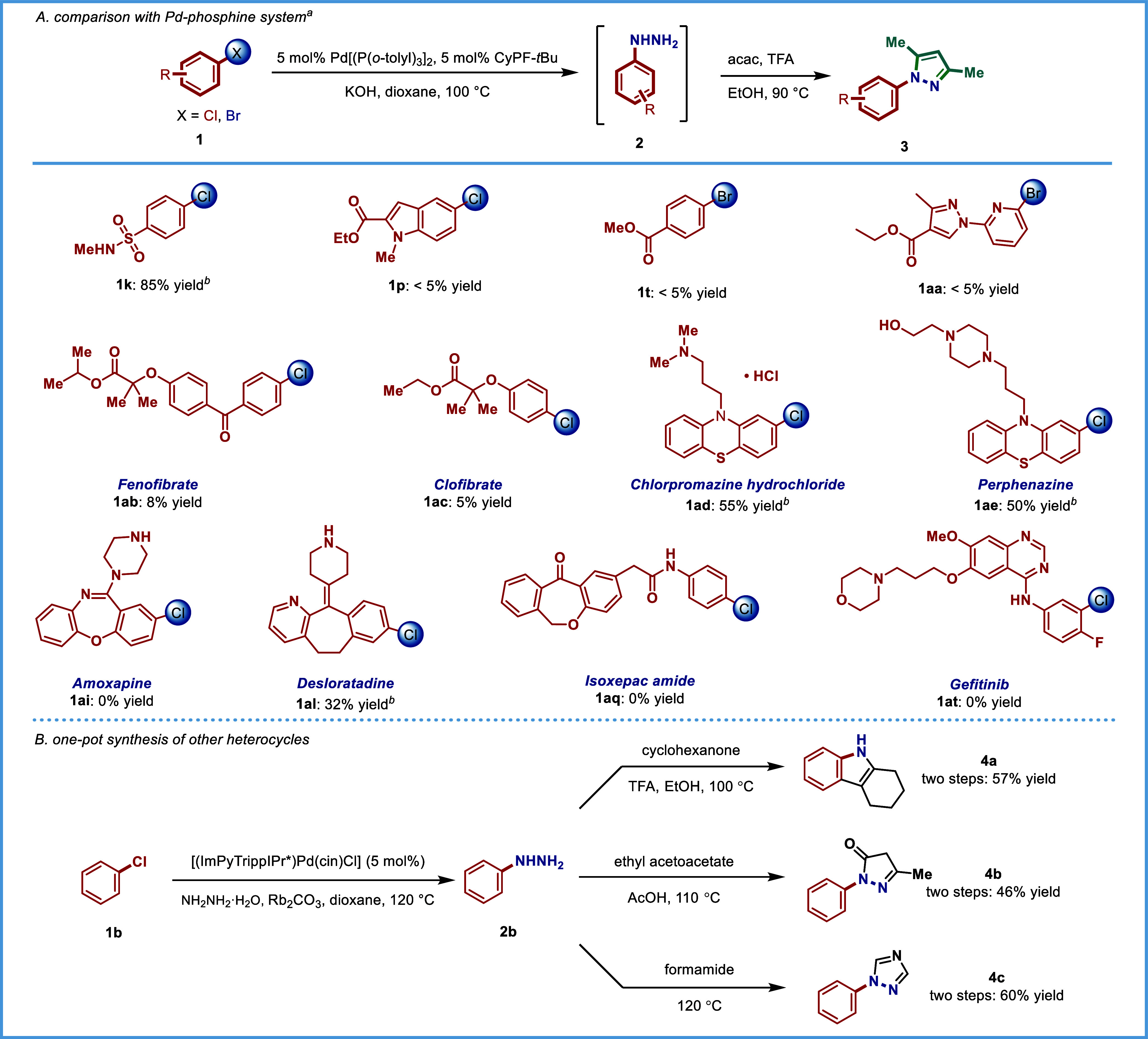
Comparison with the Pd-Phosphine System and Derivation of Arylhydrazines

a Conditions: 1 (0.2 mmol, 1.0 equiv), NH_2_NH_2_·H_2_O (3 equiv), Pd­[(P­(o-tolyl)_3_]_2_ (5 mol %), CyPF-*t*Bu (5 mol %), KOH (4.5 equiv), dioxane (0.60 M), 100 °C, 16 h. ^1^H NMR yields.

b Isolated yields.

To further demonstrate the broad applicability of our method in organic synthesis, we carried out a one-pot synthesis of three additional valuable heterocycles starting from aryl chlorides (Table 4B). Following hydrazination under our standard conditions, the resulting arylhydrazines were subjected directly to cyclization without isolation. To our delight, the heterocycles—indole **4aa**, pyrazolone **4ab**, and triazole **4ac**—were obtained smoothly in good yields. These results highlight the versatility of our protocol in constructing diverse nitrogen-containing heterocycles from simple starting materials.

### Ligand Characterization

The key aspect of sterically demanding, L-shaped, and flexible ImPy ligands is the spatial environment of the catalytic pocket. To better understand the unified impact of rigid biaryl C5-substitution and the flexible N2-wingtip, we synthesized a linear [(ImPyTrippIPr*)­CuCl] complex. It is now well-established that accurate determination of geometry of catalytic pockets of NHC ligands is possible only in linear [NHC–M–X] complexes.^46,52^ Remarkably, the X-ray crystallographic analysis of [(ImPyTrippIPr*)­CuCl] revealed the (%V_bur_) of 62.0% with 66.6, 67.3, 53.6, 60.6% for each quadrant (Figure 2), indicating ImPyTrippIPr* to be the most sterically hindered N-heterocyclic carbene ligand developed to date.^46,52^ The values can be compared with the (%V_bur_) of 48.5% for the linear imidazol-2-ylidene [(IPr)­CuCl] with 47.5, 49.5, 47.5, 49.5% for each quadrant.^54^ It should be further noted that [(ImPyTrippIPr*)­CuCl] features a unique unsymmetrical steric quadrant distribution, which is in contrast with the symmetrical quadrants of [(IPr)­CuCl]. The combination of steric shielding and flexible twisting enforces a sterically controlled arrangement of the catalytic pocket in this class of N-heterocyclic carbenes.

To gain deeper insight into the electronic properties of the ligand, the Rh­(I) complex [(ImPyTrippIPr*)­Rh­(CO)_2_Cl] and selenium adduct [(ImPyTrippIPr*)­Se] were synthesized from the corresponding NHC salt (Figure 3).^55^ [(ImPyTrippIPr*)­Rh­(COD)­Cl] was generated first via the reaction of the carbene precursor and commercially available [Rh­(COD)­Cl]_2_ in the presence of KO*t*Bu. Subsequently, the carbonyl complex [(ImPyTrippIPr*)­Rh­(CO)_2_Cl] was obtained in excellent yield upon treatment with carbon monoxide. [(ImPyTrippIPr*)­Rh­(CO)_2_Cl] exhibited two CO stretching frequencies in DCM at ν_*trans*_ = 1990.6 cm^–1^ and ν_*cis*_ = 2072.5 cm^–1^ respectively, enabling the determination of the Tolman electronic parameter (TEP) as 2045.5 cm^–1^. This result indicates that the σ-donating ability of ImPyTrippIPr* lies between that of classic five-membered ring NHCs (e.g., IMes: TEP = 2051 cm^–1^) and six-membered ring NHCs (e.g., 6-Mes: TEP = 2043 cm^–1^).^55a,55b^

**Figure 3:**
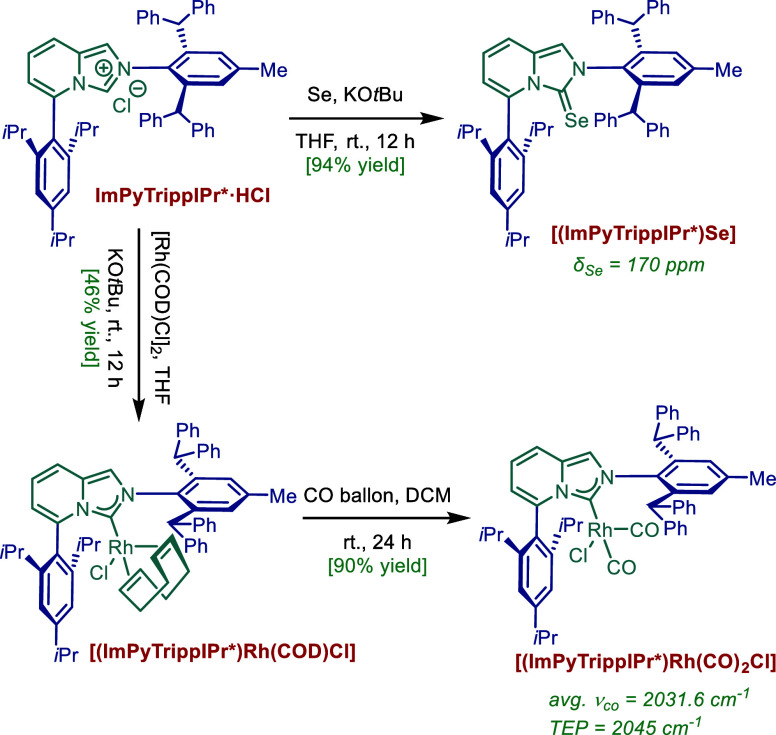
Characterization of the Electronic Properties of ImPyTrippIPr*.

Next, the selenium adduct [(ImPyTrippIPr*)­Se] was prepared to evaluate the ligand’s π-accepting ability. The observed δ_Se_ value of 170 ppm (in CDCl_3_) for [(ImPyTrippIPr*)­Se] indicates a stronger π-accepting character than IPr (δ_Se_ = 90 ppm), and is comparable to that of SIPr (δ_Se_ = 190 ppm).^55c–55e^ Overall, this architecture is likely to open up new design opportunities of sterically defined and strongly σ-donating and π-accepting N-heterocyclic carbene architectures for general applications in catalysis that are not easily accessible in other ligand scaffolds.

### Large Scale Synthesis

In consideration of the reactivity of biaryl, sterically demanding and flexible ImPyTrippIPr* ligands, we developed large-scale, chromatography-free, and modular ligand synthesis on a 10 g scale in three steps from commercially available materials (Figure 4). Synthetic access to reactive ligands that operate under user-friendly benchtop conditions is the key determinant of the adoption of reactive ligand classes in catalysis research.^56^ In this context, another advantage of our hydrazination protocol is the rapid accessibility of the ligands. Thus, protection of 6-bromopicolinaldehyde with ethylene glycol afforded acetal **4** in quantitative yield after aqueous workup. Nickel-catalyzed Kumada cross-coupling gave key acetal intermediate 6 in 85% yield. The cyclization step was performed using paraformaldehyde as the C1 source in the presence of HCl. Trituration of the crude product afforded 13.84 g of ImPyTrippIPr*HCl in 89% yield. It is particularly noteworthy that chromatography is not required for any of the steps, indicating that this class of ligands is readily scalable for a broad range of potential applications.

**Figure 4:**
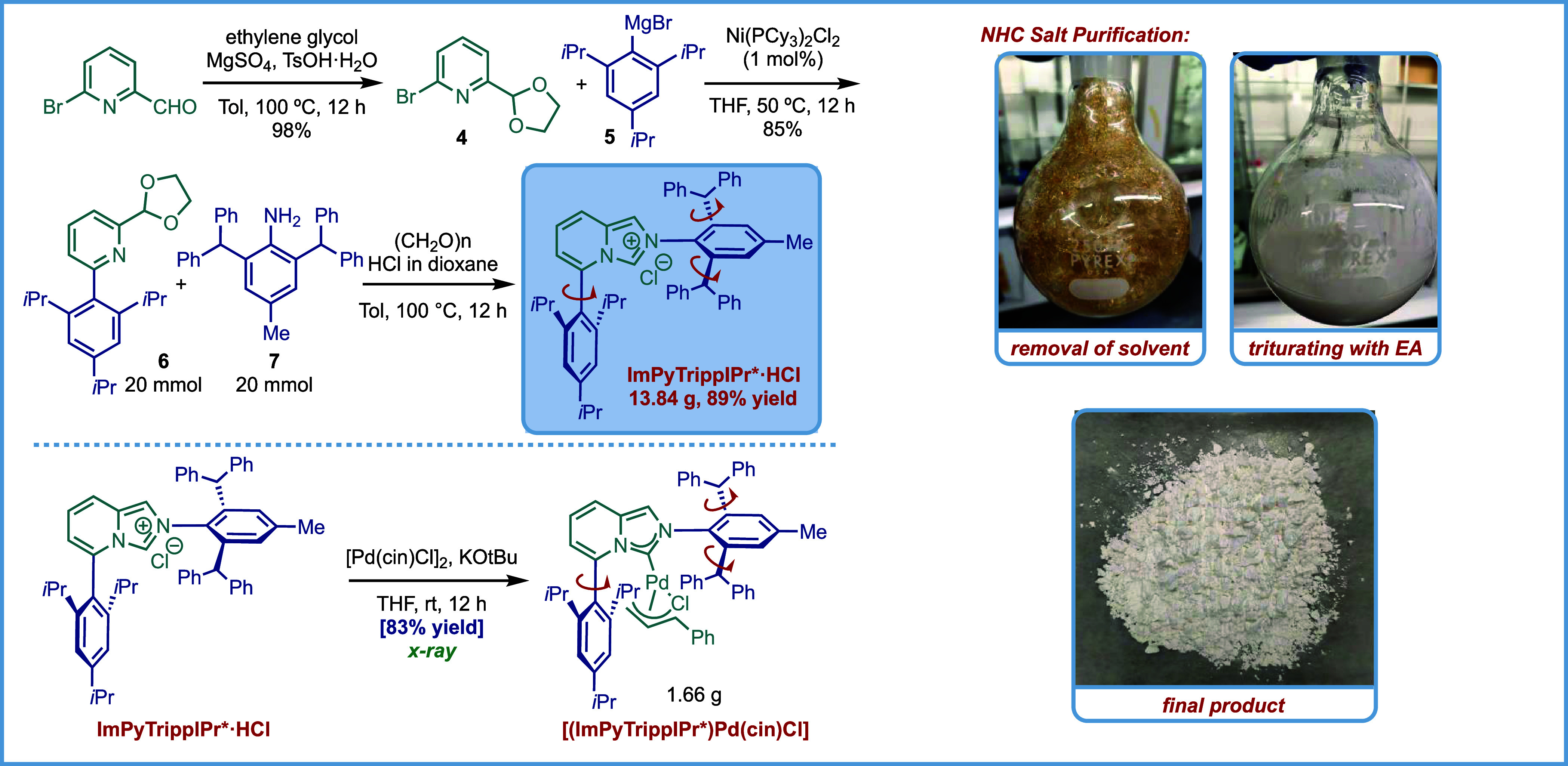
Chromatography-free large scale synthesis of ImPyTrippIPr*HCl and [(ImPyTrippIPr*)­Pd­(cin)­Cl].

### Mechanistic Studies

To gain insight into the reaction mechanism, comprehensive studies were conducted (Figure 5). Specifically, (1) we determined the rate orders, (2) measured the KIE, and (3) compared the kinetic profiles with both strong and weak base. Importantly, these new results show that the rate-limiting step shifted as the base changed.

**Figure 5:**
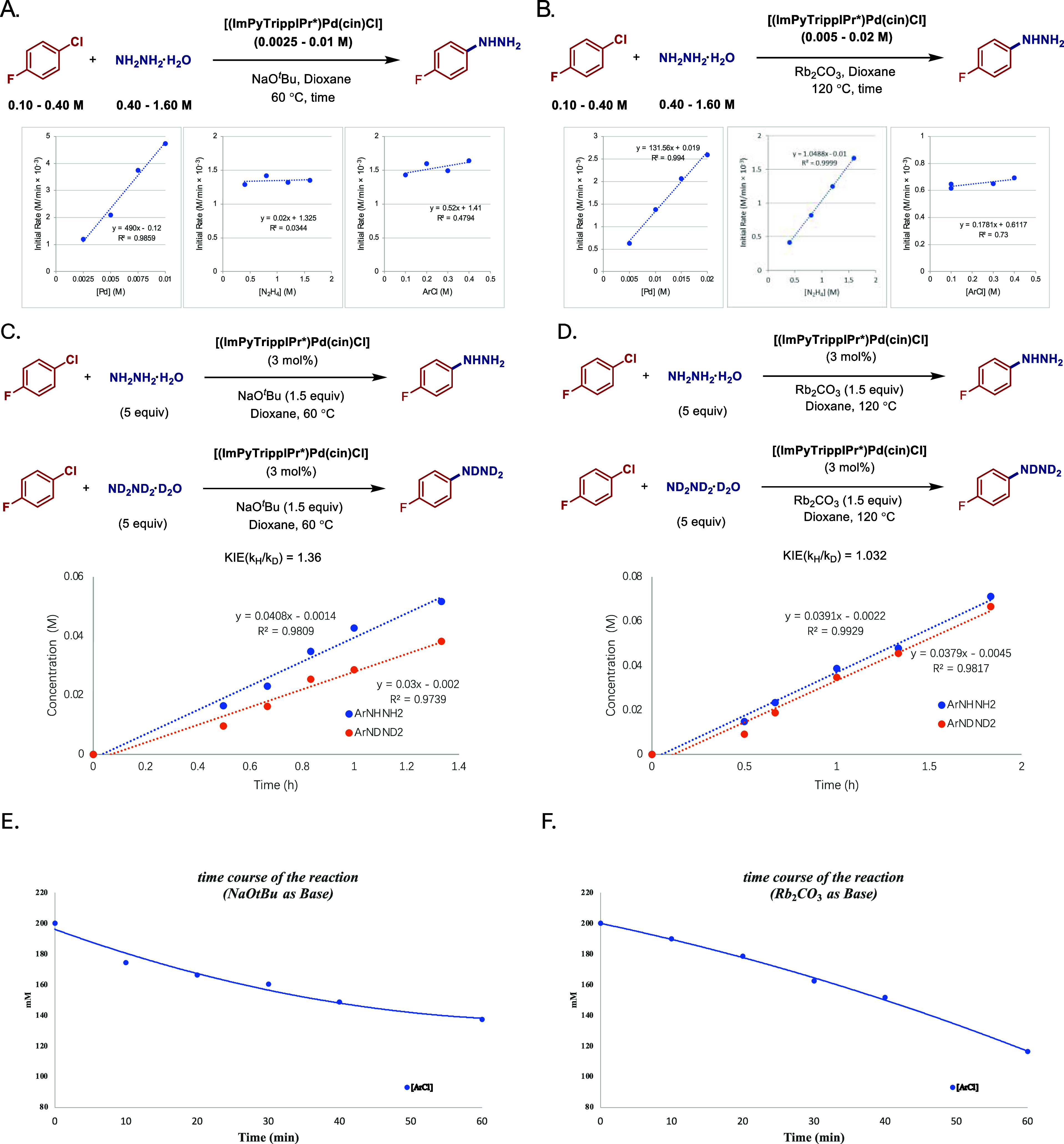
(A) Initial rates in the presence of NaO*t*Bu with various concentration of palladium catalyst, hydrazine and chloroarene. (B) Initial rates in the presence of Rb_2_CO_3_ with various concentration of palladium catalyst, hydrazine and chloroarene. (C) KIE of arylhydrazine with NaO*t*Bu. (D) KIE of arylhydrazine with Rb_2_CO_3_. (E) Time course of the reaction with NaO*t*Bu. (F) Time course of the reaction with Rb_2_CO_3._ See the SI for additional details.

It can be assumed that after activation to Pd(0), the NHC-Pd(0) complex inserts into an aryl halide to form arylpalladium halide,^45^ which then coordinates with hydrazine. Arylpalladium­(II) hydrazido intermediates are generated after deprotonation of hydrazine.^33^ Finally, the arylpalladium­(II) hydrazido species undergoes reductive elimination, yielding the product and regenerating NHC-Pd(0). To elucidate the mechanism, we first measured the initial rates with two different sets of conditions: NaO*t*Bu at 60 °C and Rb_2_CO_3_ at 120 °C. As shown in Figure 5A, the reaction was first-order in palladium, zero-order in hydrazine and aryl chloride in the presence of NaO*t*Bu. However, when NaO*t*Bu was replaced with a weak base, Rb_2_CO_3_, the order of hydrazine became first-order dependence (Figure 5B), indicating a shift of mechanism arising from the change in base. Considering the well-established mechanism for oxidative addition of NHC-Pd(0) complexes,^45^ oxidative addition is not likely the rate-determining step for both bases. Next, we carried out KIE experiments with ND_2_ND_2_·D_2_O to further clarify the mechanism. With NaO*t*Bu, we observed a secondary KIE of 1.36 (Figure 5C), while an equilibrium KIE of 1.03 was obtained with Rb_2_CO_3_ (Figure 5D), suggesting that deprotonation of the hydrazine is not the rate-limiting step in both cases. Time course of the reaction using NaO*t*Bu and Rb_2_CO_3_ as a function of aryl chloride consumption is shown in Figure 5E,F. Moreover, a kinetic comparison between aryl chlorides and aryl bromides with Rb_2_CO_3_ demonstrated that aryl chlorides reacted faster than aryl bromides under the same conditions (see Figure S6); however, no difference between aryl chloride and bromide was observed using NaO*t*Bu (see Figure S5). This reactivity trend likely arises from the formation of an equilibrium between arylpalladium halide complexes and μ-halogen dimers.^45d^ The stability of μ-bromide Pd dimers is higher than that of chloride dimers.

Overall, the above results demonstrate that the resting state of the catalytic cycle is an arylpalladium halide species. When the reaction is conducted with Rb_2_CO_3_, reductive elimination is the rate-limiting-step. The data (1) provide crucial insights into the present process and (2) offer guidance for the design and development of C–N coupling reactions of general synthetic interest.

### DFT Studies

Density functional theory (DFT) studies were conducted to investigate the unique reactivity of sterically demanding, L-shaped, and flexible ligands. The aim was to gain insight into the mechanism of the coupling reaction, as well as to elucidate the key role played by N-heterocyclic carbene (NHC) ligands.^57^ In this study, we selected the palladium-catalyzed cross-coupling of 4-chlorotoluene with hydrazine as the model reaction to investigate the details of the proposed mechanism. The computed Gibbs free energy profile of this model reaction is shown in Figure 6A. The primary coordination of 4-chlorotoluene to palladium forms the active catalyst **1**, entering the catalytic cycle. **1** then underwent oxidative addition via the transition state **TS1** with an energy barrier of 15.8 kcal/mol to give intermediate **2**. Coordination of hydrazine and the CO_3_^2–^ anion generated anionic tetracoordinated species **3**, which was exergonic by 46.5 kcal/mol. Next, the proton of hydrazine transferred to the O atom of the CO_3_^2–^ anion via the transition state **TS2**, leading to the formation of species **4**. Simultaneously, HCO_3_^–^ anion was released, producing the prereductive elimination intermediate **5**. The subsequent reductive elimination step proceeded with a barrier of only 16.5 kcal/mol, generating intermediate **6**. The dissociation of the chlorine anion produced product complex **7**. Subsequently, the release of the *p*-tolylhydrazine product from **7** by exchanging with 4-chlorotoluene regenerated **1** (see Figure S11). Finally, the product is also deprotonated by the CO_3_^2–^ anion, producing the more stable product-**0**, concomitantly releasing the HCO_3_^–^ anion.

**Figure 6:**
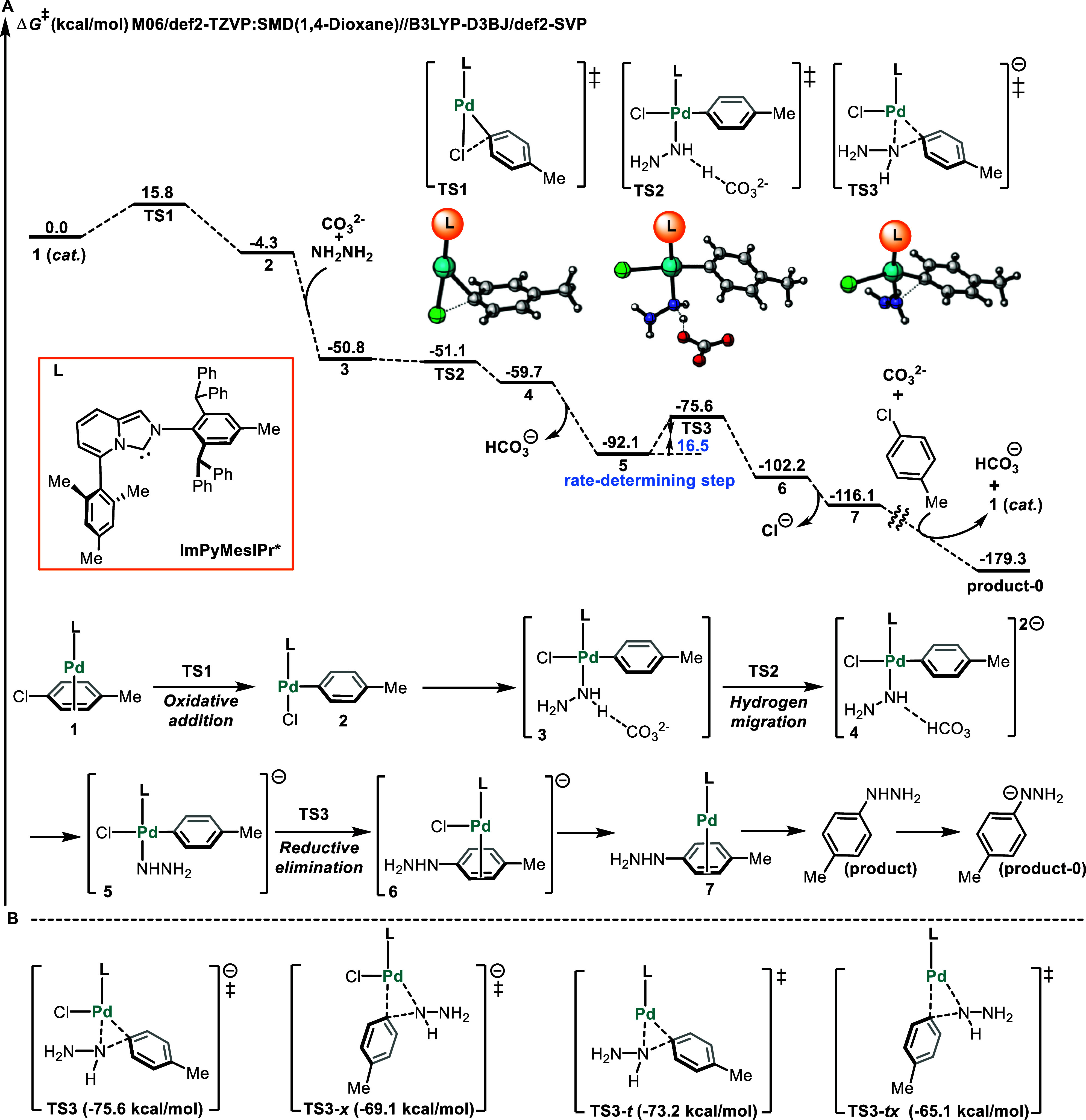
(A) Gibbs free energy profile of the palladium-catalyzed cross-coupling of 4-chlorotoluene with hydrazine. (B) All possible transient state structures and relative free energies for the reductive elimination step.

The pre-reductive elimination intermediate **5** was the on-cycle resting state of the catalytic cycle. The rate-determining step was the reductive elimination process **TS3**, which required a free energy barrier of 16.5 kcal/mol compared with that of the prereductive elimination intermediate **5**. Additionally, Intrinsic Reaction Coordinate (IRC) calculations were performed to verify the connectivity of transition state **TS2** (see Figure S12 in the SI). We also considered other possible transition states in the reductive elimination step (see **TS3-x**, **TS3-t**, and **TS3-tx** in Figure 6B). However, the level of **TS3-x** was higher than that of **TS3** by 6.5 kcal/mol, making it unsuitable. Furthermore, both the neutral transition state **TS3-t** and **TS3-tx** were also higher than the anionic transition state **TS3** by 2.4 and 10.5 kcal/mol, respectively. Therefore, these transition states were ruled out due to their very high energy barriers.

## Conclusions

In conclusion, cross-coupling between aryl halides and hydrazine has been a largely unsolved problem in drug discovery research despite the broad utility of the coupling products as privileged motifs to access an array of molecular targets. In this study, we demonstrated the late-stage hydrazination of complex pharmaceuticals for drug discovery research. The unprecedented mild method for hydrazination of aryl halides hinges on the development of nucleophilic, biaryl, sterically demanding, L-shaped, and simultaneously flexible N-heterocyclic carbene ligands. The most active ligand ImPyTrippIPr* represents one of the most sterically demanding N-heterocyclic carbenes known so far.^58^ The utility has been demonstrated by excellent functional group tolerance and broad substrate scope in the selective cross-coupling of complex pharmaceuticals covering various metabolic diseases to give highly useful aryl hydrazines directly from active pharmaceuticals. Extensive mechanistic and DFT studies were conducted to gain insight into the reaction mechanism and guide the future development of C–N coupling reactions of general synthetic interest. The presented reactivity establishes a powerful entry into cross-coupling with challenging nucleophiles in drug discovery and development and expands the available chemical space for new generations of ligands and therapeutics.

## Supplementary Material



## Notes

Experimental details, characterization data, CIF files, and computational details (PDF)
