# Supplementary material for: Diversification of Pharmaceuticals via Late-Stage Hydrazination
Source: ACS Catal. 2025 Jul 29;15(16):13846–59. doi: 10.1021/acscatal.5c03458 (PMC12362435; doi:10.1021/acscatal.5c03458)
Supplement: Supplementary file 1 [file cs5c03458_si_001.pdf]

Supporting Information

Diversification of Pharmaceuticals via Late-Stage Hydrazination

Tongliang Zhou,*[,a,‖] Chaoyue Zhao,[b,f,‖] Shiyi Yang,[a,‖] Elwira Bisz,[c] Błażej Dziuk,[d] Roger Lalancette,[a] Roman Szostak,[e] Xin Hong,*[,b] and Michal Szostak*[,a]

[a]Department of Chemistry, Rutgers University, 73 Warren Street, Newark, New Jersey 07102, United States

[b]Center of Chemistry for Frontier Technologies, Department of Chemistry, State Key Laboratory of Clean Energy Utilization, Zhejiang University, Hangzhou 310027, P.R. China; School of Chemistry and Chemical Engineering, Henan Normal University, Xinxiang 453007, P.R. China

[c]Department of Chemistry, Opole University, 48 Oleska Street, Opole 45-052, Poland

[d]Department of Chemistry, University of Science and Technology, Norwida 4/6, Wroclaw 50-373, Poland

[e]Department of Chemistry, Wroclaw University, F. Joliot-Curie 14, Wroclaw 50-383, Poland

[f]Ningbo Key Laboratory of Agricultural Germplasm Resources Mining and Environmental Regulation, College of Science and Technology, Ningbo University, Ningbo 315300, China

[‖]These authors contributed equally to the work.

tzhou@scripps.edu; *hxchem@zju.edu.cn*; *michal.szostak@rutgers.edu*

Table of Contents S1

List of Known Compounds/General Methods S2
Experimental Procedures and Characterization Data S3

- Small Scale Synthesis of ImPyTrippIPr*·HCl S3
- Chromatography-Free Large-Scale Synthesis of ImPyTrippIPr*·HCl S4
- Synthesis of [(ImPyTrippIPr*)Pd(cin)Cl] Complex S7
- General Procedures for Hydrazination of Aryl Halides S8
- Additional Optimization Studies S10
- Characterization Data of Hydrazination Products S13
- Late-Stage Hydrazination of Complex Pharmaceuticals S27
- One-pot Synthesis of Heterocycles S42
- Mechanistic Studies S44

Crystallographic Studies S60

- Details of Crystal Structure Analysis S60
- Large ORTEP Structures of [(TrippIPr*)Pd(cin)Cl] and [(TrippIPr*)CuCl] S61
- Crystallographic Comparison of Pd and Cu Complexes S65
- Synthesis of [(ImPyTrippIPr*)CuCl] S66
- Synthesis of [(ImPyTrippIPr*)Rh(CO)$_2$Cl] S67
- Synthesis of [(ImPyTrippIPr*)Se] S69

References S70
Computational Methods S72
The Cartesian Coordinates of the stationary points S79
NMR Spectra S169

List of Known Compounds/General Methods

All experiments were performed using standard Schlenk techniques under nitrogen or argon unless stated otherwise. All solvents were purchased at the highest commercial grade and used as received or after purification by passing through activated alumina columns or distillation from sodium/benzophenone under nitrogen. All solvents were deoxygenated prior to use. All other chemicals were purchased at the highest commercial grade and used as received. Reaction glassware was oven-dried at 140 °C for at least 24 h or flame-dried prior to use, allowed to cool under vacuum and purged with argon (three cycles). All products were identified using ^{1}H NMR analysis and comparison with authentic samples. GC and/or GC/MS analysis was used for volatile products. All yields refer to yields determined by ^{1}H NMR and/or GC or GC/MS using an internal standard (optimization) and isolated yields (preparative runs) unless stated otherwise. ^{1}H NMR and ^{13}C NMR spectra were recorded in $CDCl_3$ on Bruker spectrometers at 500 (^{1}H NMR) and 125 MHz (^{13}C NMR). All shifts are reported in parts per million (ppm) relative to residual $CHCl_3$ peak (7.26 and 77.2 ppm, ^{1}H NMR and ^{13}C NMR, respectively). All coupling constants (*J*) are reported in hertz (Hz). Abbreviations are: s, singlet; d, doublet; t, triplet; q, quartet; brs, broad singlet. GC-MS chromatography was performed using Agilent HP6890 GC System and Agilent 5973A inert XL EI/CI MSD using helium as the carrier gas at a flow rate of 1 mL/min and an initial oven temperature of 50 °C. The injector temperature was 250 °C. The detector temperature was 250 °C. For runs with the initial oven temperature of 50 °C, temperature was increased with a 10 °C/min ramp after 50 °C hold for 3 min to a final temperature of 220 °C, then hold at 220 °C for 15 min (splitless mode of injection, total run time of 22.0 min). High-resolution mass spectra (HRMS) were measured on a 7T Bruker Daltonics FT-MS instrument. All flash chromatography was performed using silica gel, 60 Å, 300 mesh. TLC analysis was carried out on glass plates coated with silica gel 60 F254, 0.2 mm thickness. The plates were visualized using a 254 nm UV lamp or aqueous potassium permanganate. ^{1}H NMR and ^{13}C NMR data are given for all compounds in the Supporting Experimental for characterization purposes. ^{1}H NMR, ^{13}C NMR, and HRMS data are given for all new compounds.

Experimental Procedures and Characterization Data

Small Scale Synthesis of ImPyTripp*·HCl

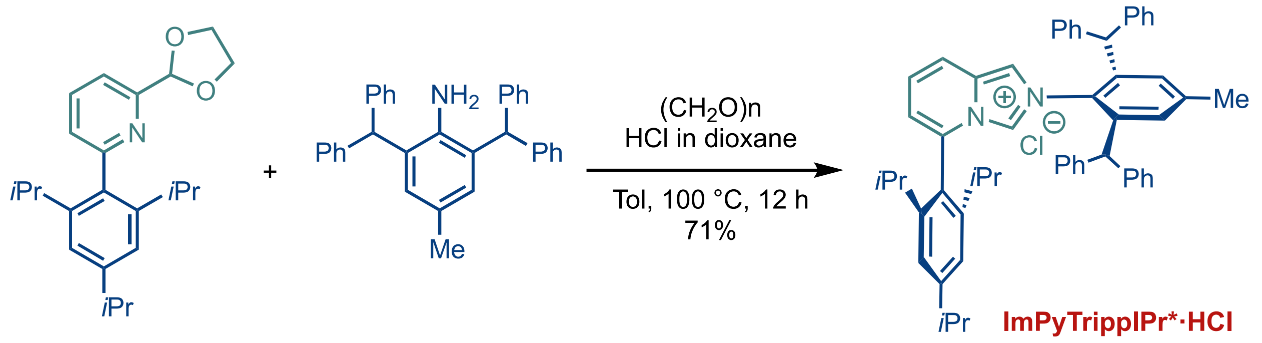

2-(2,6-Dibenzhydryl-4-methoxyphenyl)-5-(2,4,6-triisopropylphenyl)imidazo[1,5-*a*]pyridin-2-ium chloride (ImPyTrippIPr*·HCl). 2-(1,3-Dioxolan-2-yl)-6-(2,4,6-triisopropylphenyl)pyridine was prepared according to the previous report.[1] An oven-dried 100 mL round-bottomed flask equipped with a stir bar was charged with 2-(1,3-dioxolan-2-yl)-6-(2,4,6-triisopropylphenyl)pyridine (450 mg, 2.0 mmol, 1.0 equiv), 2,6-dibenzhydryl-4-methylaniline (879 mg, 2 mmol, 1.0 equiv), paraformaldehyde (90 mg, 3.0 mmol, 1.5 equiv) and toluene (10 mL). The reaction mixture was stirred at 100 °C and 4 M HCl in dioxane (1 mL, 4.0 mmol, 2 equiv) was added. The resulting reaction mixture was stirred at 100 °C for 12 h. The crude product was purified by column chromatography (CH_2Cl_2/MeOH 50/1 to 15/1). The title product was obtained by trituration from diethyl ether/ethyl acetate as white solid in 71 % yield (1.11 g). **^{1}H NMR (500 MHz, $CDCl_3$)** δ 8.71 (s, 1H), 7.76 (s, 2H), 7.26 – 7.17 (m, 14H), 7.14 – 7.09 (m, 1H), 6.90 (d, *J* = 6.7 Hz, 4H), 6.79 – 6.71 (m, 6H), 6.40 (s, 1H), 4.89 (s, 2H), 3.05 – 2.97 (m, 1H), 2.35 – 2.27 (m, 2H), 2.21 (s, 3H), 1.34 (d, *J* = 7.0 Hz, 6H), 1.15 (d, *J* = 6.7 Hz, 6H), 1.08 (d, *J* = 6.9 Hz, 6H). **^{13}C NMR (125 MHz, $CDCl_3$)** δ 153.2, 148.0, 142.5, 142.0, 141.8, 140.8, 140.8, 133.1, 130.8, 130.5, 129.3, 129.2, 128.8, 128.7, 127.9, 127.4, 127.3, 124.2, 122.7, 122.5, 122.0, 119.0, 118.7, 52.2, 34.6, 31.6, 29.8, 25.2, 24.6, 24.0, 22.0. **HRMS** calcd for $C_{55}H_{55}N_2$ (M^+ – Cl) 743.4360, found 743.4390.

Chromatography-Free Large-Scale Synthesis of ImPyTrippIPr*·HCl

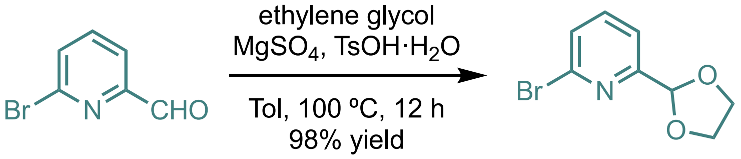

2-Bromo-6-(1,3-dioxolan-2-yl)pyridine. A mixture of 6-bromopyridine-2-carboxaldehyde (18.6 g, 100 mmol, 1 equiv), ethylene glycol (11.2 mL, 12.4 g, 200 mmol, 2 equiv), *p*-toluenesulfonic acid monohydrate (1.72 g, 10 mmol, 10 mol%) and $MgSO_4$ (24 g, 200 mmol, 2 equiv) in toluene (100 mL) was heated at 100 °C until >95% conversion of the starting material as monitored by GC/MS. Upon cooling, aqueous $NaHCO_3$ solution was added to the reaction mixture. The organic layer was separated, and the water layer was extracted with ethyl acetate (100 mL × 2). The combined organic layers were washed with brine and dried over Na_2SO_4. The solvent was removed in vacuo to give the product as colorless oil in 98% yield (22.5 g).

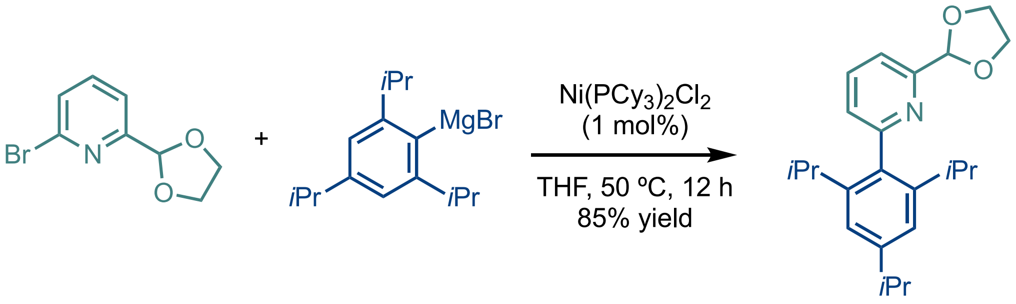

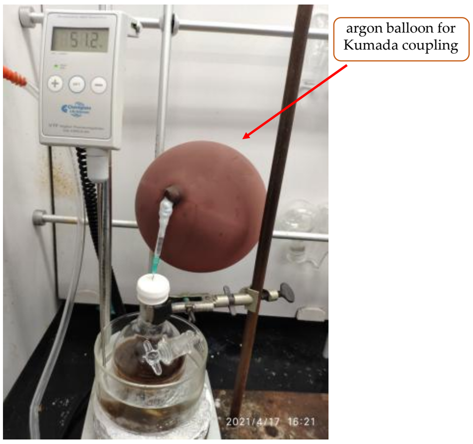

reaction setup

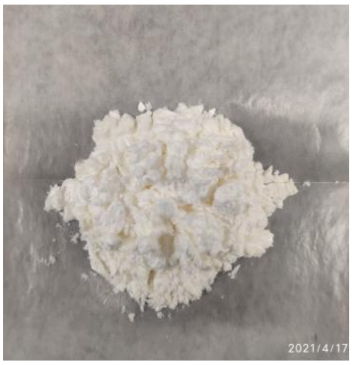

final product

2-(1,3-Dioxolan-2-yl)-6-(2,4,6-triisopropylphenyl)pyridine. Activated magnesium turnings (1.38 g, 57.6 mmol, 1.44 equiv) were suspended in anhydrous THF (40 mL). To the mixture at 60 °C was

slowly added 2-bromo-1,3,5-triisopropylbenzene (2.72 g, 9.6 mmol). The Grignard reaction was then initiated by the addition of catalytic 1,2-dibromoethane (ca. 100 μL). The remaining 2-bromo-1,3,5-triisopropylbenzene (10.88 g, 38.4 mmol, total 1.2 equiv) was added slowly. After complete addition, the reaction mixture was heated at 60 °C for 2 h. To a well-stirred suspension of 2-bromo-6-(1,3-dioxolan-2-yl)pyridine **4** (9.2 g, 40 mmol, 1.0 equiv) and $Ni(PCy_3)Cl_2$ (276 mg, 0.4 mmol, 1 mol%) in anhydrous THF (20 mL) was slowly added the above Grignard solution over 15 minutes. The resultant brown solution was heated at 50 °C for 12 h, after which the mixture was poured over aqueous NH_4Cl solution (100 mL). The aqueous layer was extracted with ethyl acetate (100 mL × 2), and the combined organic extracts were washed with brine (100 mL × 2) and dried over anhydrous Na_2SO_4, filtered and concentrated in vacuo. The residue was loaded onto a short pad of silica gel. After washing with hexane, the residue was eluted with hexane/ethyl acetate (4/1) till fully recovery of the product. The filtrate was concentrated in vacuo to give the desired product **6** as white solid in 85% yield (12.2 g).

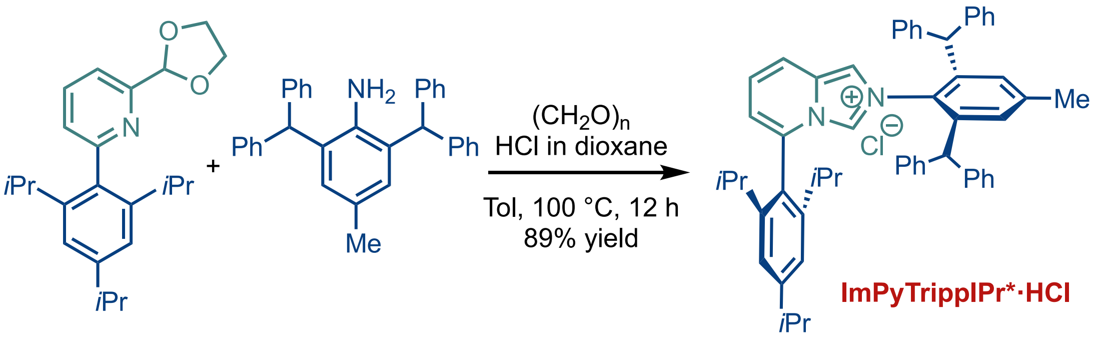

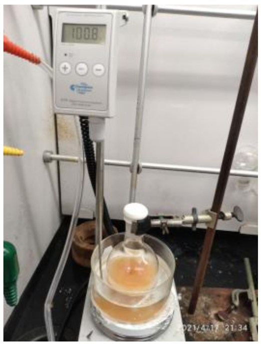
reaction setup

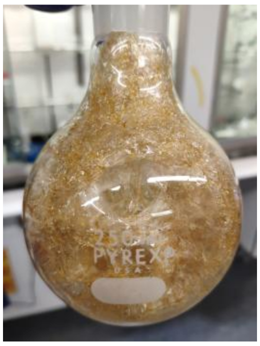
after removal of solvent

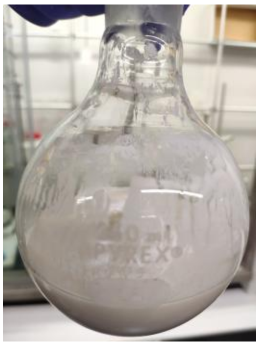
triturate with EA
(EA = ethyl acetate)

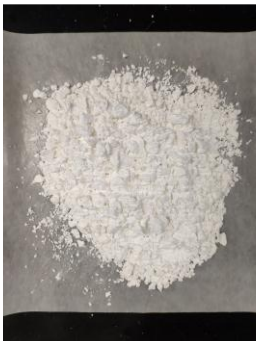
final product

ImPyTrippIPr*·HCl. To a mixture of **6** (7.08 g, 20 mmol, 1.0 equiv), 2,6-dibenzhydryl-4-methylaniline (8.79 g, 20 mmol, 1.0 equiv), paraformaldehyde (0.72 g, 24 mmol, 1.2 equiv) in toluene (40 mL, 0.5 M) was added 4 M HCl in dioxane (20 mL, 80 mmol, 4 equiv). The mixture was then

heated at 100 °C for 12 h. Solvent was removed under reduced pressure. The residue was dried under high vacuum to get foamy solid which was then triturated with ethyl acetate (40 mL) to give the desired product as white solid in 89% yield (13.84 g). NMR spectroscopic data agreed with data from small scale synthesis.

Synthesis of [(ImPyTrippIPr*)Pd(cin)Cl]

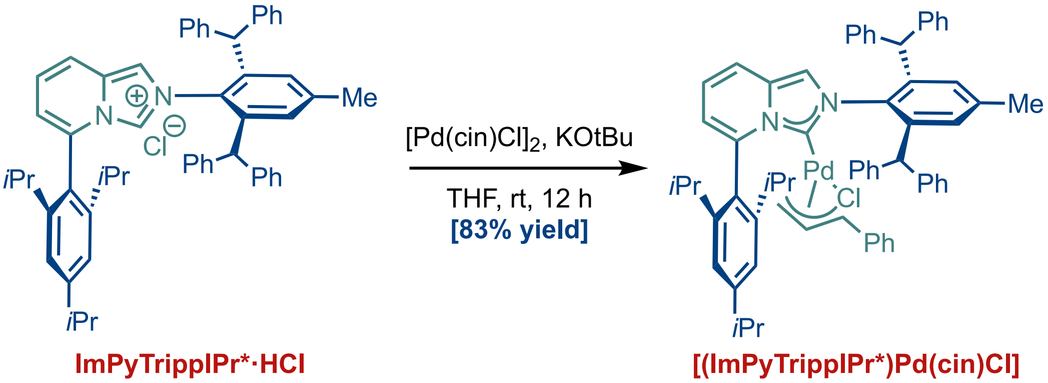

[(ImPyTrippIPr*)Pd(cin)Cl]. An oven-dried flask equipped with a stir bar was charged with ImPyTrippIPr*·HCl (1.64 g, 2.1 mmol, 1.05 equiv), KO*t*Bu (314 mg, 2.8 mmol, 1.4 equiv), and $[Pd(cin)Cl]_2$ (518 mg, 1 mmol, 0.5 equiv), placed under a positive pressure of argon and subjected to three evacuation/backfilling cycles under high vacuum. Anhydrous THF (20 mL) was added, and the resulting reaction mixture was stirred at room temperature for 12 h. The reaction mixture was diluted with CH_2Cl_2 and passed through a short pad of silica gel covered with a pad of Celite eluting with CH_2Cl_2 until the product was completely recovered. The solution was collected and concentrated. After triturating with pentane, the desired product was obtained as yellow solid by recrystallization from dichloromethane/hexane and drying under high vacuum. Yield 83% (1.66 g). NMR data were acquired at 90 °C. Note that [(ImPyTrippIPr*)Pd(cin)Cl] shows peak broadening in NMR spectra due to Pd-allyl isomerization.[1] **^{1}H NMR (500 MHz, DMSO, 363 K)** δ 7.30 – 7.12 (m, 20H), 7.02 (d, *J* = 7.9 Hz, 4H), 6.81 – 6.77 (m, 1H), 6.75 – 6.61 (m, 7H), 6.57 (d, *J* = 6.4 Hz, 1H), 5.17 – 4.88 (m, 2H), 3.98 – 3.82 (m, 1H), 3.02 – 2.97 (m, 2H), 2.87 – 2.73 (m, 1H), 2.18 (s, 3H), 1.36 – 1.32 (m, 1H), 1.31 (d, *J* = 6.9 Hz, 6H), 1.28 – 1.26 (m, 1H), 1.25 – 1.11 (m, 6H), 1.07 – 0.96 (m, 6H). **^{13}C NMR (125 MHz, DMSO, 363 K)** δ 173.0, 149.2, 142.8, 142.6, 137.8, 137.5, 136.6, 135.9, 130.5, 130.3, 129.4, 129.1, 128.9, 128.7, 128.4, 127.6, 127.5, 127.3, 126.6, 126.0, 125.9, 120.6, 120.1, 117.8, 117.4, 116.6, 107.4, 89.2, 49.5, 45.6, 33.2, 30.2, 25.9, 23.4, 22.4, 20.8. **HRMS** calcd for $C_{64}H_{63}N_2Pd$ (M^+ – Cl) 965.4042, found 965.4065. Crystals suitable for X-ray crystallography were obtained from saturated hexane/DCM solution.

General Procedures for Hydrazination of Aryl Halides

General Procedure for *In Situ* Optimization of Hydrazination of Aryl Halides. An oven-dried vial equipped with a stir bar was charged with aryl halide (neat, 1.0 equiv), NaO*t*Bu (typically, 1.5 equiv), $[Pd(cin)Cl]_2$ (typically, 2.5 mol%), ligand (typically, 10 mol%) placed under a positive pressure of argon, and subjected to three evacuation/backfilling cycles under high vacuum. Dioxane (typically, 0.1 M) was added, and the reaction mixture was stirred at room temperature for 15 min. Hydrazine hydrate (50% solution in water, typically, 5 equiv) was added before placing in a preheated oil bath (typically, 120 °C) and stirred for 16 h. After 16 h, the reaction mixture was cooled down to room temperature, diluted with CH_2Cl_2 (10 mL), filtered, and concentrated. A sample was analyzed by ^{1}H NMR ($CDCl_3$, 500 MHz) and GC-MS to obtain conversion, and yield using internal standard and comparison with authentic samples.

General Procedure for Optimization of Hydrazination of Aryl Halides with [(ImPyTrippIPr*)Pd(cin)Cl)]. An oven-dried vial equipped with a stir bar was charged with aryl halide (neat, 1.0 equiv), base (typically, 3 equiv), [(ImPyTrippIPr*)Pd(cin)Cl)] (typically, 5 mol%), placed under a positive pressure of argon, and subjected to three evacuation/backfilling cycles under high vacuum. Dioxane (typically, 0.1 M) and hydrazine hydrate (50% solution in water, typically, 5 equiv) were added with vigorous stirring, the reaction mixture was placed in a preheated oil bath (typically, 120 °C) and stirred for 16 h. After 16 h, the reaction mixture was cooled down to room temperature, diluted with CH_2Cl_2 (10 mL), filtered, and concentrated. A sample was analyzed by ^{1}H NMR ($CDCl_3$, 500 MHz) and GC-MS to obtain conversion, and yield using internal standard and comparison with authentic samples. Purification by chromatography on silica gel afforded the title product.

General Procedure for Hydrazination of Aryl Halides with [(ImPyTrippIPr*)Pd(cin)Cl)]. An oven-dried vial equipped with a stir bar was charged with aryl halide (neat, 1.0 equiv), Rb_2CO_3 (typically, 3 equiv), [(ImPyTrippIPr*)Pd(cin)Cl)] (typically, 5 mol%), placed under a positive pressure of argon, and subjected to three evacuation/backfilling cycles under high vacuum. Dioxane (typically, 0.2 M) and hydrazine hydrate (50% solution in water, typically, 5 equiv) were added with vigorous

stirring, the reaction mixture was placed in a preheated oil bath (typically, 120 °C) and stirred for 16 h. After 16 h, 2 M TFA in ethanol (typically, 4 equiv) and acetylacetone (typically, 9 equiv) was added and the mixture was further stirred for 12 h at 90 °C. Afterward, the reaction mixture was cooled down to down to room temperature, diluted with CH_2Cl_2 (10 mL), filtered, and concentrated. A sample was analyzed by ^1H NMR ($CDCl_3$, 500 MHz) and GC-MS to obtain conversion, and yield using internal standard and comparison with authentic samples. Purification by chromatography on silica gel afforded the title product.

General Procedure for Hydrazination of Aryl Halides with Pd-Phosphine System. An An oven-dried vial equipped with a stir bar was charged with aryl halide (neat, 1.0 equiv), KOH (typically, 4.5 equiv), Pd[(P(*o*-tolyl)$_3$]$_2$ (typically, 5 mol%), CyPF-tBu (typically, 5 mol%), placed under a positive pressure of argon, and subjected to three evacuation/backfilling cycles under high vacuum. Dioxane (typically, 0.6 M) and hydrazine hydrate (50% solution in water, typically, 3 equiv) were added with vigorous stirring, the reaction mixture was placed in a preheated oil bath (typically, 100 °C) and stirred for 16 h. After 16 h, 2 M TFA in ethanol (typically, 4 equiv) and acetylacetone (typically, 9 equiv) was added and the mixture was further stirred for 12 h at 90 °C. Afterward, the reaction mixture was cooled down to down to room temperature, diluted with CH_2Cl_2 (10 mL), filtered, and concentrated. A sample was analyzed by ^1H NMR ($CDCl_3$, 500 MHz) to obtain conversion, and yield using internal standard and comparison with authentic samples.

Additional Optimization Studies

Table S1. *Optimization of the reaction conditions: bases and concentration.*[a]

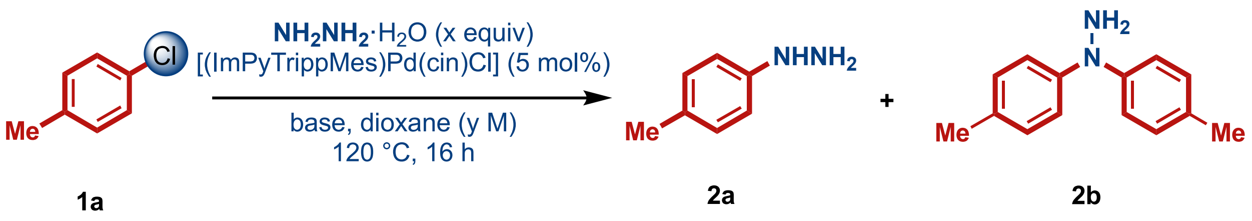

Entry	Base	x	y	Conversion of 1a (%)[b]	Yield of 2a (%)[b]	Ratio (2a/2b)[b]
1	K_3PO_4 (3 equiv)	5	0.1	>99	58	7.7/1
2	K_3PO_4 (3 equiv)	10	0.1	>99	59	32.2/1
3	K_3PO_4 (3 equiv)	5	0.05	>99	47	10.1/1
4	KOH (2 equiv)	5	0.1	>99	67	10.1/1
5	KOH (2 equiv)	10	0.1	>99	36	12.3/1
6	KOH (2 equiv)	5	0.05	>99	78	12.3/1
7	NaO*t*Bu (1.5 equiv)	5	0.1	>99	74	11.5/1

[a]Conditions: 4-chlorotoluene (1.0 equiv), $NH_2NH_2 \cdot H_2O$ (x equiv), [(ImPyTrippMes)Pd(cin)Cl] (5 mol%), K_3PO_4 (3 equiv)/KOH (2 equiv)/NaO*t*Bu (1.5 equiv), dioxane (y M), 120 °C, 16 h. [b]Determined by GC/^{1}H NMR.

Table S2. *Optimization of the reaction conditions: bases.*[a]

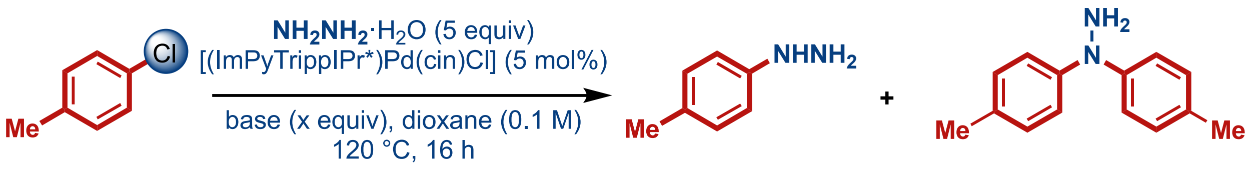

1a 2a 2b

Entry	Base	x	Conversion of 1a (%)[b]	Yield of 2a (%)[b]	Ratio (2a/2b)[b]
1	NaO*t*Bu	1.5	>99	79	65.7/1
2	Na_2CO_3	3	81	<5	--/--
3	Et_3N	3	41	0	--/--
4	DBU	3	41	0	--/--
5	Cs_2CO_3	5	>99	76	49/1
6	K_3PO_4	5	>99	39	13.3/1
7	K_3PO_4	3	>99	81	27.6/1
8[c]	Cs_2CO_3	3	>99	90	32.3/1
9[c,d]	Rb_2CO_3	3	77	66	32.3/1
10[c,e]	Rb_2CO_3	3	72	58	17.2/1
11[c,f]	Rb_2CO_3	3	78	54	8.1/1
12[c,g]	Rb_2CO_3	3	62	42	39/1
13[c]	Rb_2CO_3	1	75	29	8.1/1
13[c]	Rb_2CO_3	2	90	58	32.2/1
12[c,h]	Rb_2CO_3	3	>99	63	12.3/1

[a]Conditions: 4-chlorotoluene (1.0 equiv), $NH_2NH_2\cdot H_2O$ (5 equiv), [(ImPyTrippIPr*)Pd(cin)Cl] (5 mol%), base (x equiv), dioxane (0.1 M), 120 °C, 16 h. [b]Determined by GC/^{1}H NMR. [c]dioxane (0.2 M). [d][(ImPyTrippIPr*)Pd(cin)Cl] (2.5 mol%). [e][(ImPyTrippIPr*)Pd(cin)Cl] (2.5 mol%). [f]100 °C. [g]80 °C. [h]$NH_2NH_2\cdot H_2O$ (2.5 equiv).

Table S3. *Optimization of the reaction conditions: solvents.*[a]

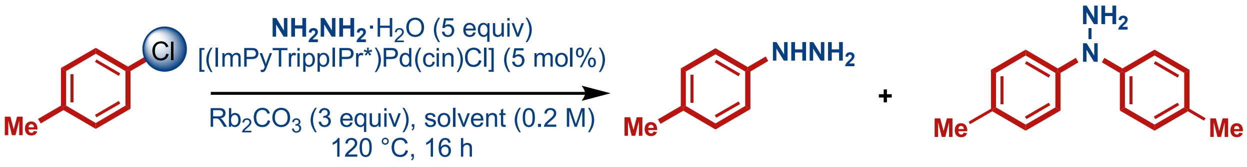

1a 2a 2b

Entry	Solvent	Conversion of 1a (%)[b]	Yield of 2a (%)[b]	Ratio (2a/2b)[b]
1	dioxane	>99	94	32.3/1
2	toluene	10	0	--/--
3	THF	>99	93	27.6/1
4	CPME	81	0	--/--
5	EtOH	82	57	19/1
6	*i*PrOH	88	60	32.2/1
7	*n*BuOH	86	49	24/1
8	*t*BuOH	>99	83	27.6/1
9	*t*AmOH	>99	67	9/1

[a]Conditions: 4-chlorotoluene (1.0 equiv), $NH_2NH_2 \cdot H_2O$ (5 equiv), [(ImPyTrippIPr*)Pd(cin)Cl] (5 mol%), Rb_2CO_3 (3 equiv), solvent (0.2 M), 120 °C, 16 h. [b]Determined by GC/^{1}H NMR.

Characterization Data of Cross-Coupling Products

3,5-Dimethyl-1-(*p*-tolyl)-1*H*-pyrazole (Table 2, 3a)

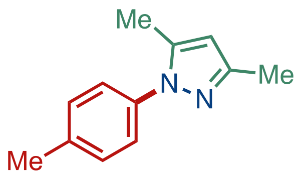

According to the general procedure, the reaction of 4-chlorotoluene (0.2 mmol), hydrazine hydrate (5 equiv), Rb_2CO_3 (3.0 equiv) and [(ImPyTrippIPr*)Pd(cin)Cl] (5 mol%) in dioxane (0.2 M) for 16 h, afforded after work-up and chromatography the title compound in 81 % yield (30.1 mg). Colorless oil. ^{1}H NMR (500 MHz, $CDCl_3$) δ 7.30 (d, J = 8.4 Hz, 2H), 7.24 (d, J = 8.4 Hz, 2H), 5.98 (s, 1H), 2.39 (s, 3H), 2.30 (s, 3H), 2.27 (s, 3H). ^{13}C NMR (125 MHz, $CDCl_3$) δ 148.8, 139.6, 137.5, 137.4, 129.7, 124.9, 106.7, 21.2, 13.6, 12.4. NMR spectroscopic data agreed with literature values.[2]

3,5-Dimethyl-1-phenyl-1*H*-pyrazole (Table 2, 3b)

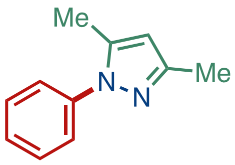

According to the general procedure, the reaction of chlorobenzene (0.2 mmol), hydrazine hydrate (5 equiv), Rb_2CO_3 (3.0 equiv) and [(ImPyTrippIPr*)Pd(cin)Cl] (5 mol%) in dioxane (0.2 M) for 16 h at 120 °C then with acetylacetone (9 equiv) and 2M TFA in ethanol (4 equiv) for 12 h at 90 °C, afforded after work-up and chromatography the title compound in 54 % yield (18.6 mg). Yellow oil. ^{1}H NMR (500 MHz, $CDCl_3$) δ 7.47 – 7.40 (m, 4H), 7.37 – 7.30 (m, 1H), 5.99 (s, 1H), 2.30 (s, 6H). ^{13}C NMR (125 MHz, $CDCl_3$) δ 149.1, 140.1, 139.5, 129.1, 127.3, 124.9, 107.0, 13.6, 12.5. NMR spectroscopic data agreed with literature values.[2]

3,5-Dimethyl-1-(*o*-tolyl)-1*H*-pyrazole (Table 2, 3c)

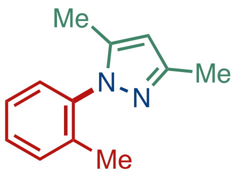

According to the general procedure, the reaction of 2-chlorotoluene (0.2 mmol), hydrazine hydrate (5 equiv), Rb_2CO_3 (3.0 equiv) and [(ImPyTrippIPr*)Pd(cin)Cl] (5 mol%) in dioxane (0.2 M) for 16 h, afforded after work-up and chromatography the title compound in 74 % yield (27.5 mg). Yellow oil. ^{1}H NMR (500 MHz, $CDCl_3$) δ 7.29 – 7.21 (m, 2H), 7.18 (d, *J* = 8.6 Hz, 1H), 7.14 (d, *J* = 7.6 Hz, 1H), 5.89 (s, 1H), 2.22 (s, 3H), 1.98 (s, 6H). ^{13}C NMR (125 MHz, $CDCl_3$) δ 148.6, 140.3, 138.9, 136.4, 130.9, 129.0, 128.1, 126.6, 105.0, 17.4, 13.7, 11.4. NMR spectroscopic data agreed with literature values.[2]

1-(3-Methoxyphenyl)-3,5-dimethyl-1*H*-pyrazole (Table 2, 3d)

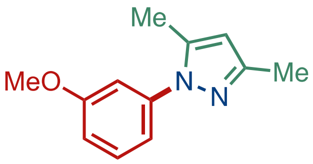

According to the general procedure, the reaction of 3-chloroanisole (0.2 mmol), hydrazine hydrate (5 equiv), Rb_2CO_3 (3.0 equiv) and [(ImPyTrippIPr*)Pd(cin)Cl] (5 mol%) in dioxane (0.2 M) for 16 h at 120 °C then with acetylacetone (9 equiv) and 2M TFA in ethanol (4 equiv) for 12 h at 90 °C, afforded after work-up and chromatography the title compound in 85 % yield (34.4 mg). Colorless oil. ^{1}H NMR (500 MHz, $CDCl_3$) δ 7.33 (t, *J* = 8.4 Hz, 1H), 7.02 – 6.96 (m, 2H), 6.89 (d, *J* = 7.5 Hz, 1H), 5.99 (s, 1H), 3.84 (s, 3H), 2.31 (s, 3H), 2.30 (s, 3H). ^{13}C NMR (125 MHz, $CDCl_3$) δ 160.2, 149.1, 141.1, 139.6, 129.7, 117.1, 113.5, 110.5, 107.1, 55.6, 13.6, 12.6. NMR spectroscopic data agreed with literature values.[3]

1-(4-Fluorophenyl)-3,5-dimethyl-1*H*-pyrazole (Table 2, 3e)

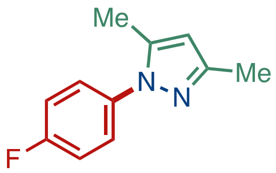

According to the general procedure, the reaction of 1-chloro-4-fluorobenzene (0.2 mmol), hydrazine hydrate (5 equiv), Rb_2CO_3 (3.0 equiv) and [(ImPyTrippIPr*)Pd(cin)Cl] (5 mol%) in dioxane (0.2 M) for 16 h then with acetylacetone (9 equiv) and 2M TFA in ethanol (4 equiv) for 12 h at 90 °C, afforded after work-up and chromatography the title compound in 82 % yield (31.1 mg). Colorless oil. ^{1}H NMR (500 MHz, $CDCl_3$) δ 7.42 – 7.35 (m, 2H), 7.16 – 7.10 (m, 2H), 5.99 (s, 1H), 2.29 (s, 3H), 2.27 (s, 3H). ^{13}C NMR (125 MHz, $CDCl_3$) δ 161.8 (d, *J* = 247.0 Hz), 149.2, 139.6, 136.2 (d, *J* = 3.1 Hz), 126.8 (d, *J* = 8.2 Hz), 116.0 (d, *J* = 23.2 Hz), 107.0, 13.6, 12.4. ^{19}F NMR (471 MHz, $CDCl_3$) δ -114.43. NMR spectroscopic data agreed with literature values.[4]

4-(3,5-Dimethyl-1*H*-pyrazol-1-yl)benzonitrile (Table 2, 3f)

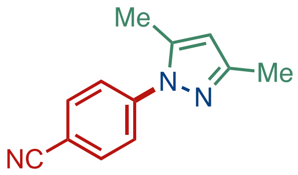

According to the general procedure, the reaction of 4-chlorobenzonitrile (0.2 mmol), hydrazine hydrate (5 equiv), Rb_2CO_3 (3.0 equiv) and [(ImPyTrippIPr*)Pd(cin)Cl] (5 mol%) in dioxane (0.2 M) for 16 h at 120 °C then with acetylacetone (9 equiv) and 2M TFA in ethanol (4 equiv) for 12 h at 90 °C, afforded after work-up and chromatography the title compound in 87 % yield (34.3 mg). Colorless solid. ^{1}H NMR (500 MHz, $CDCl_3$) δ 7.74 (d, *J* = 8.7 Hz, 2H), 7.61 (d, *J* = 8.7 Hz, 2H), 6.05 (s, 1H), 2.39 (s, 3H), 2.29 (s, 3H). ^{13}C NMR (125 MHz, $CDCl_3$) δ 150.7, 143.6, 139.8, 133.3, 124.1, 118.6, 110.2, 109.1, 13.6, 13.1. NMR spectroscopic data agreed with literature values.[5]

3,5-Dimethyl-1-(4-(trifluoromethyl)phenyl)-1*H*-pyrazole (Table 2, 3g)

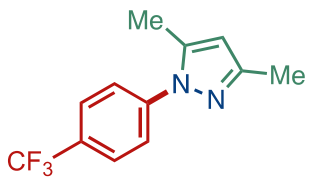

According to the general procedure, the reaction of 4-chlorobenzonitrile (0.2 mmol), hydrazine hydrate (5 equiv), Rb_2CO_3 (3.0 equiv) and [(ImPyTrippIPr*)Pd(cin)Cl] (5 mol%) in dioxane (0.2 M) for 16 h at 120 °C then with acetylacetone (9 equiv) and 2M TFA in ethanol (4 equiv) for 12 h at 90 °C, afforded after work-up and chromatography the title compound in 76 % yield (36.5 mg). Yellow oil. ^{1}H NMR (500 MHz, $CDCl_3$) δ 7.71 (d, *J* = 8.5 Hz, 2H), 7.59 (d, *J* = 8.5 Hz, 2H), 6.04 (s, 1H), 2.36 (s, 3H), 2.30 (s, 3H). ^{13}C NMR (125 MHz, $CDCl_3$) δ 150.1, 142.9, 139.7, 129.0 (q, *J* = 32.9 Hz), 127.3, 126.4 (q, *J* = 3.9 Hz), 124.3, 124.1 (q, *J* = 272.0 Hz), 120.8, 108.3, 13.6, 12.9. ^{19}F NMR (471 MHz, $CDCl_3$) δ -62.37. NMR spectroscopic data agreed with literature values.[4]

4-(3,5-Dimethyl-1*H*-pyrazol-1-yl)-*N*,*N*-dimethylbenzamide (Table 2, 3h)

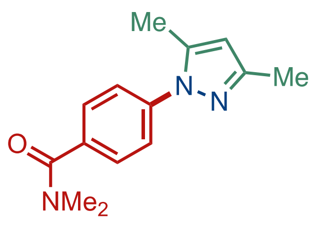

According to the general procedure, the reaction of 4-chloro-*N*,*N*-dimethylbenzamide (0.2 mmol), hydrazine hydrate (5 equiv), Rb_2CO_3 (3.0 equiv) and [(ImPyTrippIPr*)Pd(cin)Cl] (5 mol%) in dioxane (0.2 M) for 16 h at 120 °C then with acetylacetone (9 equiv) and 2M TFA in ethanol (4 equiv) for 12 h at 90 °C, afforded after work-up and chromatography the title compound in 61 % yield (29.7 mg). Colorless solid. ^{1}H NMR (500 MHz, $CDCl_3$) δ 7.53 – 7.44 (m, 4H), 6.01 (s, 1H), 3.12 (s, 3H), 2.99 (s, 3H), 2.32 (s, 3H), 2.29 (s, 3H). ^{13}C NMR (125 MHz, $CDCl_3$) δ 171.0, 149.6, 140.9, 139.7, 135.1, 128.1, 124.5, 107.6, 39.7, 35.6, 13.6, 12.6. NMR spectroscopic data agreed with literature values.[2]

4-(3,5-Dimethyl-1*H*-pyrazol-1-yl)-*N*-methylbenzamide (Table 2, 3i)

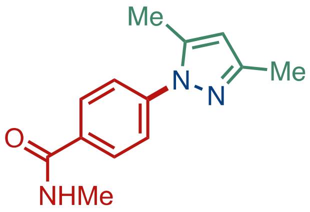

According to the general procedure, the reaction of 4-chloro-*N*-methylbenzamide (0.2 mmol), hydrazine hydrate (5 equiv), Rb_2CO_3 (3.0 equiv) and [(ImPyTrippIPr*)Pd(cin)Cl] (5 mol%) in dioxane (0.2 M) for 16 h at 120 °C then with acetylacetone (9 equiv) and 2M TFA in ethanol (4 equiv) for 12 h at 90 °C, afforded after work-up and chromatography the title compound in 73 % yield (33.4 mg). Colorless solid. ^{1}H NMR (500 MHz, $CDCl_3$) δ 7.83 (d, J = 8.7 Hz, 2H), 7.49 (d, J = 8.5 Hz, 2H), 6.36 (s, 1H), 6.02 (s, 1H), 3.02 (d, J = 4.9 Hz, 3H), 2.33 (s, 3H), 2.29 (s, 3H). ^{13}C NMR (125 MHz, $CDCl_3$) δ 167.5, 149.8, 142.4, 139.7, 133.1, 127.9, 124.2, 108.0, 27.0, 13.6, 12.8. HRMS calcd for $C_{13}H_{16}N_3O$ (M^+ + H) 230.1288, found 268.1271.

4-(3,5-Dimethyl-1*H*-pyrazol-1-yl)-*N*-phenylbenzamide (Table 2, 3j)

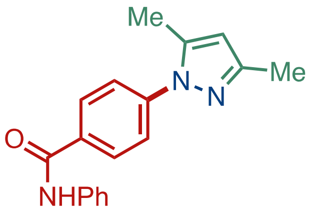

According to the general procedure, the reaction of 4-chloro-*N*-phenylbenzamide (0.2 mmol), hydrazine hydrate (5 equiv), Rb_2CO_3 (3.0 equiv) and [(ImPyTrippIPr*)Pd(cin)Cl] (5 mol%) in dioxane (0.2 M) for 16 h at 120 °C then with acetylacetone (9 equiv) and 2M TFA in ethanol (4 equiv) for 12 h at 90 °C, afforded after work-up and chromatography the title compound in 68 % yield (39.6 mg). Colorless solid. ^{1}H NMR (500 MHz, $CDCl_3$) δ 8.18 (s, 1H), 7.92 (d, J = 8.5 Hz, 2H), 7.65 (d, J = 8.1 Hz, 2H), 7.52 (d, J = 8.5 Hz, 2H), 7.37 (t, J = 7.9 Hz, 2H), 7.16 (t, J = 7.4 Hz, 1H), 6.04 (s, 1H), 2.35 (s, 3H), 2.30 (s, 3H). ^{13}C NMR (125 MHz, $CDCl_3$) δ 165.1, 150.0, 142.7, 139.8, 138.0, 133.3, 129.2,

128.2, 124.8, 124.2, 120.5, 108.2, 13.6, 12.8. HRMS calcd for $C_{18}H_{17}N_3OK$ (M^+ + K) 330.1003, found 330.0980.

4-(3,5-Dimethyl-1*H*-pyrazol-1-yl)-*N*-methylbenzenesulfonamide (Table 2, 3k)

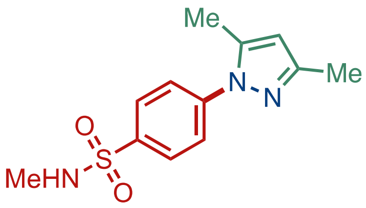

According to the general procedure, the reaction of 4-chloro-*N*-methylbenzenesulfonamide (0.2 mmol), hydrazine hydrate (5 equiv), Rb_2CO_3 (3.0 equiv) and [(ImPyTrippIPr*)Pd(cin)Cl] (5 mol%) in dioxane (0.2 M) for 16 h at 120 °C then with acetylacetone (9 equiv) and 2M TFA in ethanol (4 equiv) for 12 h at 90 °C, afforded after work-up and chromatography the title compound in 88 % yield (46.7 mg). Colorless solid. ^{1}H NMR (500 MHz, Chloroform-*d*) δ 7.92 (d, J = 8.6 Hz, 2H), 7.62 (d, J = 8.7 Hz, 2H), 6.05 (s, 1H), 4.70 (q, J = 5.4 Hz, 1H), 2.66 (d, J = 5.3 Hz, 3H), 2.38 (s, 3H), 2.30 (s, 3H). ^{13}C NMR (125 MHz, $CDCl_3$) δ 150.5, 143.5, 139.9, 137.0, 128.5, 124.3, 108.7, 29.4, 13.6, 13.0. HRMS calcd for $C_{12}H_{15}N_3O_2SK$ (M^+ + K) 304.0517, found 304.0495.

2-(3,5-Dimethyl-1*H*-pyrazol-1-yl)pyridine (Table 2, 3l)

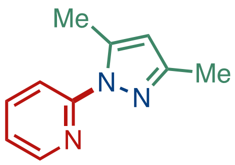

According to the general procedure, the reaction of 2-chloropyridine (0.2 mmol), hydrazine hydrate (5 equiv), Rb_2CO_3 (3.0 equiv) and [(ImPyTrippIPr*)Pd(cin)Cl] (5 mol%) in dioxane (0.2 M) for 16 h, afforded after work-up and chromatography the title compound in 66 % yield (22.8 mg). Yellow oil. ^{1}H NMR (500 MHz, $CDCl_3$) δ 8.41 (d, J = 3.8 Hz, 1H), 7.83 (d, J = 8.2 Hz, 1H), 7.77 (td, J = 7.7, 1.9 Hz, 1H), 7.14 (dd, J = 6.7, 5.3 Hz, 1H), 5.99 (s, 1H), 2.63 (s, 3H), 2.30 (s, 3H). ^{13}C NMR (125 MHz,

$CDCl_3$) δ 153.7, 150.0, 147.6, 141.6, 138.3, 120.8, 116.0, 109.1, 14.5, 13.8. NMR spectroscopic data agreed with literature values.[6]

Methyl 6-(3,5-dimethyl-1*H*-pyrazol-1-yl)nicotinate (Table 2, 3m)

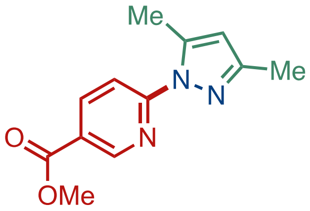

According to the general procedure, the reaction of methyl 6-chloronicotinate (0.2 mmol), hydrazine hydrate (5 equiv), Rb_2CO_3 (3.0 equiv) and [(ImPyTrippIPr*)Pd(cin)Cl] (5 mol%) in dioxane (0.2 M) for 16 h at 120 °C then with acetylacetone (9 equiv) and 2M TFA in ethanol (4 equiv) for 12 h at 90 °C, afforded after work-up and chromatography the title compound in 50 % yield (23.1 mg). White solid. ^{1}H NMR (500 MHz, $CDCl_3$) δ 9.01 (d, J = 2.4 Hz, 1H), 8.33 (dd, J = 8.6, 2.4 Hz, 1H), 7.98 (d, J = 8.7 Hz, 1H), 6.02 (s, 1H), 3.95 (s, 3H), 2.69 (s, 3H), 2.30 (s, 3H). ^{13}C NMR (125 MHz, $CDCl_3$) δ 165.7, 156.4, 151.2, 149.7, 142.8, 139.4, 122.6, 114.4, 110.3, 52.4, 15.3, 13.8. HRMS calcd for $C_{24}H_{27}N_6O_4$ ($2M^+$ + H) 463.2088, found 463.2085.

4-(3,5-Dimethyl-1*H*-pyrazol-1-yl)pyridine (Table 2, 3n)

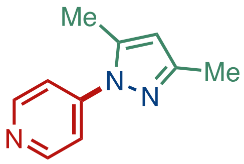

According to the general procedure, the reaction of 4-chloropyridine (0.2 mmol), hydrazine hydrate (5 equiv), Rb_2CO_3 (3.0 equiv) and [(ImPyTrippIPr*)Pd(cin)Cl] (5 mol%) in dioxane (0.2 M) for 16 h at 120 °C then with acetylacetone (9 equiv) and 2M TFA in ethanol (4 equiv) for 12 h at 90 °C, afforded after work-up and chromatography the title compound in 73 % yield (25.3 mg). Yellow oil. ^{1}H NMR (500 MHz, $CDCl_3$) δ 8.66 (d, J = 6.3 Hz, 2H), 7.48 (d, J = 6.4 Hz, 2H), 6.06 (s, 1H), 2.46 (s, 3H), 2.30

(s, 3H). ^{13}C NMR (125 MHz, $CDCl_3$) δ 151.0, 150.9, 146.8, 140.0, 117.0, 109.6, 13.7, 13.5. HRMS calcd for $C_{10}H_{12}N_3$ (M^+ + H) 174.1026, found 174.1003.

2-(3,5-Dimethyl-1*H*-pyrazol-1-yl)quinoline (Table 2, 3o)

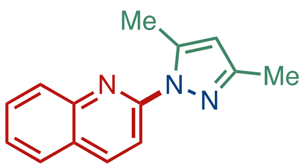

According to the general procedure, the reaction of 2-chloroquinoline (0.2 mmol), hydrazine hydrate (5 equiv), Rb_2CO_3 (3.0 equiv) and [(ImPyTrippIPr*)Pd(cin)Cl] (5 mol%) in dioxane (0.2 M) for 16 h at 120 °C then with acetylacetone (9 equiv) and 2M TFA in ethanol (4 equiv) for 12 h at 90 °C, afforded after work-up and chromatography the title compound in 74 % yield (33 mg). Yellow solid. ^{1}H NMR (500 MHz, $CDCl_3$) δ 8.21 (d, J = 8.9 Hz, 1H), 8.11 (d, J = 9.0 Hz, 1H), 7.98 (d, J = 8.5 Hz, 1H), 7.81 (d, J = 8.1 Hz, 1H), 7.69 (ddd, J = 8.4, 6.9, 1.4 Hz, 1H), 7.49 (ddd, J = 8.1, 6.9, 1.3 Hz, 1H), 6.05 (s, 1H), 2.82 (s, 3H), 2.34 (s, 3H). ^{13}C NMR (125 MHz, $CDCl_3$) δ 152.4, 150.2, 146.5, 142.4, 138.4, 130.0, 128.8, 127.7, 126.4, 125.9, 115.2, 109.7, 15.2, 13.9. HRMS calcd for $C_{28}H_{27}N_6$ ($2M^+$ + H) 447.2292, found 447.2302.

Ethyl 5-(3,5-dimethyl-1*H*-pyrazol-1-yl)-1-methyl-1*H*-indole-2-carboxylate (Table 2, 3p)

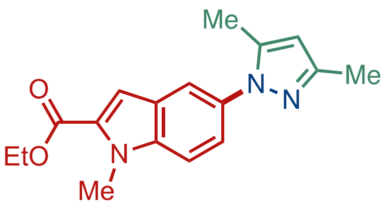

According to the general procedure, the reaction of ethyl 5-chloro-1-methyl-1*H*-indole-2-carboxylate (0.2 mmol), hydrazine hydrate (5 equiv), Rb_2CO_3 (3.0 equiv) and [(ImPyTrippIPr*)Pd(cin)Cl] (5 mol%) in dioxane (0.2 M) for 16 h at 120 °C, afforded after work-up and chromatography the title compound in 77 % yield (45.8 mg). Yellow solid. ^{1}H NMR (500 MHz, $CDCl_3$) δ 7.68 – 7.65 (m, 1H), 7.44 – 7.40 (m, 2H), 7.33 – 7.31 (m, 1H), 5.99 (s, 1H), 4.39 (q, J = 7.1 Hz, 2H), 4.11 (s, 3H), 2.32 (s, 3H), 2.26 (s, 3H), 1.42 (t, J = 7.1 Hz, 3H). ^{13}C NMR (125 MHz, $CDCl_3$) δ 162.1, 148.6, 139.9, 138.7,

133.5, 129.5, 125.7, 123.1, 119.1, 110.7, 110.4, 106.3, 60.8, 32.0, 14.5, 13.7, 12.4. HRMS calcd for $C_{17}H_{20}N_3O_2$ (M^+ + H) 298.1550, found 298.1521.

3-(3,5-Dimethyl-1*H*-pyrazol-1-yl)benzonitrile (Table 2, 3q)

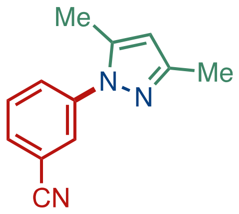

According to the general procedure, the reaction of 3-bromobenzonitrile (0.2 mmol), hydrazine hydrate (5 equiv), Rb_2CO_3 (3.0 equiv) and [(ImPyTrippIPr*)Pd(cin)Cl] (5 mol%) in dioxane (0.2 M) for 16 h at 120 °C then with acetylacetone (9 equiv) and 2M TFA in ethanol (4 equiv) for 12 h at 90 °C, afforded after work-up and chromatography the title compound in 66 % yield (26 mg). White solid. ^{1}H NMR (500 MHz, $CDCl_3$) δ 7.78 (s, 1H), 7.72 (d, *J* = 8.1 Hz, 1H), 7.61 (d, *J* = 7.8 Hz, 1H), 7.56 (t, *J* = 7.9 Hz, 1H), 6.04 (s, 1H), 2.36 (s, 3H), 2.29 (s, 3H). ^{13}C NMR (125 MHz, $CDCl_3$) δ 150.3, 140.9, 139.6, 130.4, 130.2, 128.5, 127.6, 118.1, 113.4, 108.4, 13.6, 12.77. NMR spectroscopic data agreed with literature values.[2]

3,5-Dimethyl-1-(3-(trifluoromethoxy)phenyl)-1*H*-pyrazole (Table 2, 3r)

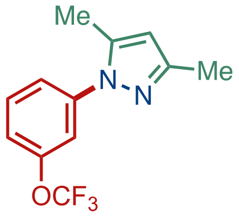

According to the general procedure, the reaction of 1-bromo-3-(trifluoromethoxy)benzene (0.2 mmol), hydrazine hydrate (5 equiv), Rb_2CO_3 (3.0 equiv) and [(ImPyTrippIPr*)Pd(cin)Cl] (5 mol%) in dioxane (0.2 M) for 16 h at 120 °C then with acetylacetone (9 equiv) and 2M TFA in ethanol (4 equiv) for 12 h at 90 °C, afforded after work-up and chromatography the title compound in 54 % yield (27.6 mg). Yellow oil. ^{1}H NMR (500 MHz, $CDCl_3$) δ 7.47 (t, *J* = 8.1 Hz, 1H), 7.40 (d, *J* = 8.9 Hz, 1H), 7.35 (s,

1H), 7.19 (d, J = 7.0 Hz, 1H), 6.02 (s, 1H), 2.34 (s, 3H), 2.29 (s, 3H). ^{13}C NMR (125 MHz, $CDCl_3$) δ 149.8, 149.5 (q, J = 2.0 Hz), 141.4, 139.6, 122.7, 120.6 (q, J = 257.9 Hz), 119.4, 117.4, 107.9, 13.6, 12.7. ^{19}F NMR (471 MHz, $CDCl_3$) δ -57.82. HRMS calcd for $C_{12}H_{12}F_3N_2O$ (M^+ + H) 279.0716, found 279.0688.

1-(4-Methoxyphenyl)-3,5-dimethyl-1*H*-pyrazole (Table 2, 3s)

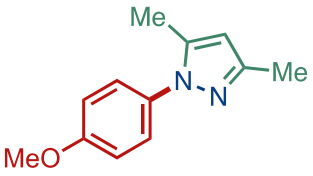

According to the general procedure, the reaction of 4-bromoanisole (0.2 mmol), hydrazine hydrate (5 equiv), Rb_2CO_3 (3.0 equiv) and [(ImPyTrippIPr*)Pd(cin)Cl] (5 mol%) in dioxane (0.2 M) for 16 h at 120 °C then with acetylacetone (9 equiv) and 2M TFA in ethanol (4 equiv) for 12 h at 90 °C, afforded after work-up and chromatography the title compound in 48 % yield (19.4 mg). Yellow oil. ^{1}H NMR (500 MHz, $CDCl_3$) δ 7.32 (d, J = 8.9 Hz, 2H), 6.95 (d, J = 8.9 Hz, 2H), 5.96 (s, 1H), 3.84 (s, 3H), 2.28 (s, 3H), 2.24 (s, 3H). ^{13}C NMR (125 MHz, $CDCl_3$) δ 158.9, 148.7, 139.6, 133.2, 126.5, 114.2, 106.4, 55.7, 13.6, 12.3. NMR spectroscopic data agreed with literature values.[2]

Methyl 4-(3,5-dimethyl-1*H*-pyrazol-1-yl)benzoate (Table 2, 3t)

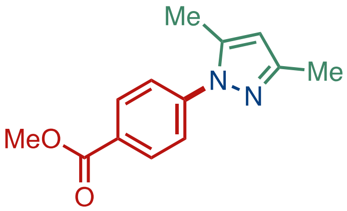

According to the general procedure, the reaction of methyl 4-bromobenzoate (0.2 mmol), hydrazine hydrate (5 equiv), Rb_2CO_3 (3.0 equiv) and [(ImPyTrippIPr*)Pd(cin)Cl] (5 mol%) in dioxane (0.2 M) for 16 h at 120 °C then with acetylacetone (9 equiv) and 2M TFA in ethanol (4 equiv) for 12 h at 90 °C, afforded after work-up and chromatography the title compound in 74 % yield (34.1 mg). White

solid. ^{1}H NMR (500 MHz, $CDCl_3$) δ 8.12 (d, *J* = 8.7 Hz, 2H), 7.54 (d, *J* = 8.7 Hz, 2H), 6.03 (s, 1H), 3.94 (s, 3H), 2.37 (s, 3H), 2.30 (s, 3H). ^{13}C NMR (125 MHz, $CDCl_3$) δ 166.6, 150.1, 143.8, 139.7, 130.7, 128.4, 123.7, 108.4, 52.4, 13.7, 13.0. NMR spectroscopic data agreed with literature values.[7]

Ethyl 4-(3,5-dimethyl-1*H*-pyrazol-1-yl)benzoate (Table 2, 3u)

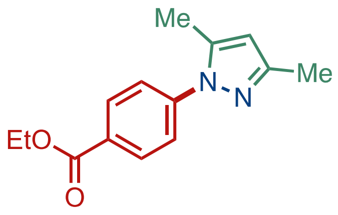

According to the general procedure, the reaction of ethyl 4-bromobenzoate (0.2 mmol), hydrazine hydrate (5 equiv), Rb_2CO_3 (3.0 equiv) and [(ImPyTrippIPr*)Pd(cin)Cl] (5 mol%) in dioxane (0.2 M) for 16 h at 120 °C then with acetylacetone (9 equiv) and 2M TFA in ethanol (4 equiv) for 12 h at 90 °C, afforded after work-up and chromatography the title compound in 60 % yield (29.3 mg). Yellow solid. ^{1}H NMR (500 MHz, $CDCl_3$) δ 8.12 (d, *J* = 8.7 Hz, 2H), 7.54 (d, *J* = 8.5 Hz, 2H), 6.03 (s, 1H), 4.40 (q, *J* = 7.1 Hz, 2H), 2.36 (s, 3H), 2.30 (s, 3H), 1.41 (t, *J* = 7.1 Hz, 3H). ^{13}C NMR (125 MHz, $CDCl_3$) δ 166.1, 150.0, 143.7, 139.7, 130.6, 128.8, 123.7, 108.3, 61.3, 14.5, 13.7, 13.0. HRMS calcd for $C_{28}H_{33}N_4O_4$ ($2M^+$ + H) 489.2496, found 489.2525.

1-(Biphenyl-4-yl)-3,5-dimethyl-1*H*-pyrazole (Table 2, 3v)

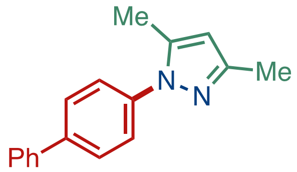

According to the general procedure, the reaction of 4-bromobiphenyl (0.2 mmol), hydrazine hydrate (5 equiv), Rb_2CO_3 (3.0 equiv) and [(ImPyTrippIPr*)Pd(cin)Cl] (5 mol%) in dioxane (0.2 M) for 16 h at 120 °C then with acetylacetone (9 equiv) and 2M TFA in ethanol (4 equiv) for 12 h at 90 °C, afforded after work-up and chromatography the title compound in 57 % yield (28.3 mg). White solid.

^{1}H NMR (500 MHz, $CDCl_3$) δ 7.67 (d, J = 8.5 Hz, 2H), 7.62 (d, J = 7.9 Hz, 2H), 7.51 (d, J = 8.5 Hz, 2H), 7.46 (t, J = 7.6 Hz, 2H), 7.37 (t, J = 7.4 Hz, 1H), 6.02 (s, 1H), 2.36 (s, 3H), 2.32 (s, 3H). ^{13}C NMR (125 MHz, $CDCl_3$) δ 149.2, 140.4, 140.2, 139.6, 139.3, 129.0, 127.8, 127.7 127.2, 125.0, 107.2, 13.7, 12.6. NMR spectroscopic data agreed with literature values.[8]

3,5-Dimethyl-1-(4-(methylthio)phenyl)-1*H*-pyrazole (Table 2, 3w)

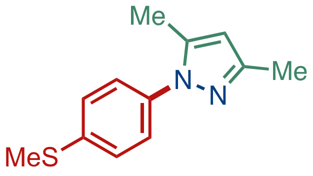

According to the general procedure, the reaction of 4-bromothioanisole (0.2 mmol), hydrazine hydrate (5 equiv), Rb_2CO_3 (3.0 equiv) and [(ImPyTrippIPr*)Pd(cin)Cl] (5 mol%) in dioxane (0.2 M) for 16 h at 120 °C then with acetylacetone (9 equiv) and 2M TFA in ethanol (4 equiv) for 12 h at 90 °C, afforded after work-up and chromatography the title compound in 94 % yield (41 mg). Colorless oil. ^{1}H NMR (500 MHz, $CDCl_3$) δ 7.37 – 7.29 (m, 4H), 5.98 (s, 1H), 2.51 (s, 3H), 2.29 (s, 3H), 2.28 (s, 3H). ^{13}C NMR (125 MHz, $CDCl_3$) δ 149.1, 139.5, 137.8, 137.3, 127.1, 125.3, 107.0, 16.2 13.7, 12.5. NMR spectroscopic data agreed with literature values.[2]

3,5-Dimethyl-1-(4-(methylsulfinyl)phenyl)-1*H*-pyrazole (Table 2, 3x)

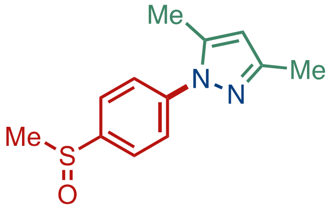

According to the general procedure, the reaction of 1-bromo-4-(methylsulfinyl)benzene (0.2 mmol), hydrazine hydrate (5 equiv), Rb_2CO_3 (3.0 equiv) and [(ImPyTrippIPr*)Pd(cin)Cl] (5 mol%) in dioxane (0.2 M) for 16 h at 120 °C then with acetylacetone (9 equiv) and 2M TFA in ethanol (4 equiv) for 12 h at 90 °C, afforded after work-up and chromatography the title compound in 65 % yield (30.4 mg).

Yellow solid. ^{1}H NMR (500 MHz, $CDCl_3$) δ 7.73 (d, *J* = 8.7 Hz, 2H), 7.63 (d, *J* = 8.5 Hz, 2H), 6.03 (s, 1H), 2.75 (s, 3H), 2.36 (s, 3H), 2.29 (s, 3H). ^{13}C NMR (125 MHz, $CDCl_3$) δ 150.0, 144.2, 142.4, 139.7, 125.1, 124.6, 108.2, 44.2, 13.6, 12.8. HRMS calcd for $C_{24}H_{28}N_4O_2S_2Na$ (M^+ + Na) 491.1546, found 491.1544.

1-(3,5-Dimethylphenyl)-3,5-dimethyl-1*H*-pyrazole (Table 2, 3y)

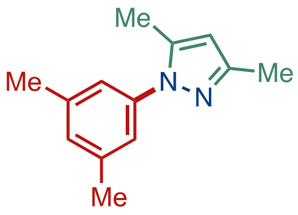

According to the general procedure, the reaction of 1-bromo-3,5-dimethylbenzene (0.2 mmol), hydrazine hydrate (5 equiv), Rb_2CO_3 (3.0 equiv) and [(ImPyTrippIPr*)Pd(cin)Cl] (5 mol%) in dioxane (0.2 M) for 16 h at 120 °C then with acetylacetone (9 equiv) and 2M TFA in ethanol (4 equiv) for 12 h at 90 °C, afforded after work-up and chromatography the title compound in 88 % yield (35.2 mg). Colorless oil. ^{1}H NMR (500 MHz, $CDCl_3$) δ 7.03 (s, 2H), 6.98 (s, 1H), 5.97 (s, 1H), 2.35 (s, 6H), 2.29 (s, 3H), 2.29 (s, 3H). ^{13}C NMR (125 MHz, $CDCl_3$) δ 148.8, 139.9, 139.5, 138.9, 129.1, 122.7, 106.7, 21.4, 13.6, 12.5. NMR spectroscopic data agreed with literature values.[9]

3,5-Dimethyl-1-(naphthalen-2-yl)-1*H*-pyrazole (Table 2, 3z)

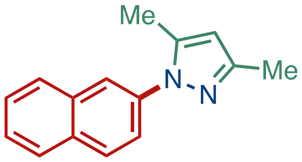

According to the general procedure, the reaction of 2-bromonaphthalene (0.2 mmol), hydrazine hydrate (5 equiv), Rb_2CO_3 (3.0 equiv) and [(ImPyTrippIPr*)Pd(cin)Cl] (5 mol%) in dioxane (0.2 M) for 16 h at 120 °C then with acetylacetone (9 equiv) and 2M TFA in ethanol (4 equiv) for 12 h at 90 °C, afforded after work-up and chromatography the title compound in 88 % yield (39.1 mg). Yellow solid. ^{1}H NMR (500 MHz, $CDCl_3$) δ 7.92 (d, *J* = 8.7 Hz, 1H), 7.90 – 7.84 (m, 3H), 7.61 (dd, *J* = 8.7,

2.1 Hz, 1H), 7.55 – 7.48 (m, 2H), 6.05 (s, 1H), 2.37 (s, 3H), 2.34 (s, 3H). ^{13}C NMR (125 MHz, $CDCl_3$) δ 149.4, 139.8, 137.5, 133.4, 132.3, 129.1, 128.2, 127.9, 126.9, 126.4, 123.5, 122.7, 107.2, 13.7, 12.7. NMR spectroscopic data agreed with literature values.[2]

Ethyl 1-(6-(3,5-dimethyl-1*H*-pyrazol-1-yl)pyridin-2-yl)-3-methyl-1*H*-pyrazole-4-carboxylate (Table 2, 3aa)

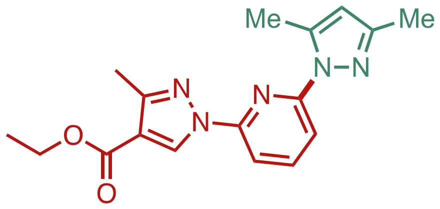

According to the general procedure, the reaction of ethyl 1-(6-bromopyridin-2-yl)-3-methyl-1*H*-pyrazole-4-carboxylate (0.2 mmol), hydrazine hydrate (5 equiv), Rb_2CO_3 (3.0 equiv) and [(ImPyTrippIPr*)Pd(cin)Cl] (5 mol%) in dioxane (0.2 M) for 16 h at 120 °C then with acetylacetone (9 equiv) and 2M TFA in ethanol (4 equiv) for 12 h at 90 °C, afforded after work-up and chromatography the title compound in 84 % yield (54.6 mg). White solid. ^{1}H NMR (500 MHz, $CDCl_3$) δ 8.04 (s, 1H), 7.95 (t, *J* = 7.9 Hz, 1H), 7.90 (d, *J* = 8.0 Hz, 1H), 7.61 (d, *J* = 7.7 Hz, 1H), 6.01 (s, 1H), 4.33 (q, *J* = 7.1 Hz, 2H), 2.86 (s, 3H), 2.61 (s, 3H), 2.30 (s, 3H), 1.38 (t, *J* = 7.1 Hz, 3H). ^{13}C NMR (125 MHz, $CDCl_3$) δ 163.79, 151.99, 150.65, 150.56, 144.88, 142.58, 141.55, 140.89, 115.24, 114.96, 114.20, 109.68, 60.23, 14.64, 14.55, 13.76, 12.95. HRMS calcd for $C_{25}H_{29}N_2O_4$ (M^+ + H) 421.2122, found 421.2102.

Late-Stage Hydrazination of Complex Pharmaceuticals

Isopropyl 2-(4-(4-(3,5-dimethyl-1*H*-pyrazol-1-yl)benzoyl)phenoxy)-2-methylpropanoate (Table 3, 3ab)

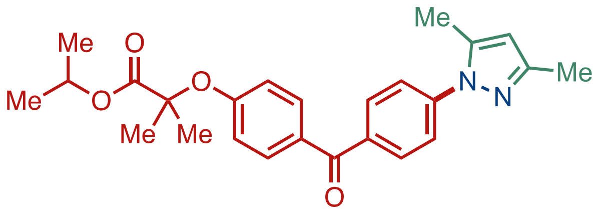

According to the general procedure, the reaction of fenofibrate (0.2 mmol), hydrazine hydrate (5 equiv), Rb_2CO_3 (3.0 equiv) and [(ImPyTrippIPr*)Pd(cin)Cl] (5 mol%) in dioxane (0.2 M) for 16 h at 120 °C then with acetylacetone (9 equiv) and 2M TFA in ethanol (4 equiv) for 12 h at 90 °C, afforded after work-up and chromatography the title compound in 86 % yield (72.3 mg). Colorless solid. ^1H NMR (500 MHz, $CDCl_3$) δ 7.84 (d, J = 8.5 Hz, 2H), 7.76 (d, J = 8.9 Hz, 2H), 7.57 (d, J = 8.5 Hz, 2H), 6.87 (d, J = 8.9 Hz, 2H), 6.04 (s, 1H), 5.13 – 5.05 (m, 1H), 2.39 (s, 3H), 2.31 (s, 3H), 1.66 (s, 6H), 1.21 (d, J = 6.3 Hz, 6H). ^{13}C NMR (125 MHz, $CDCl_3$) δ 194.7, 173.3, 159.8, 150.0, 143.0, 139.7, 136.4, 132.1, 130.9, 130.6, 123.7, 117.4, 108.2, 69.5, 25.5, 21.7, 13.7, 12.9. HRMS calcd for $C_{25}H_{29}N_2O_4$ (M^+ + H) 421.2122, found 421.2102.

Ethyl 2-(4-(3,5-dimethyl-1*H*-pyrazol-1-yl)phenoxy)-2-methylpropanoate (Table, 3ac)

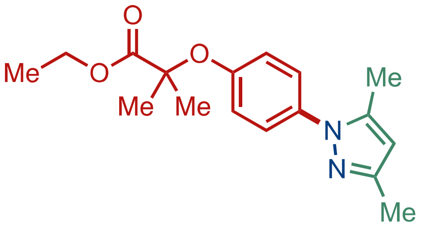

According to the general procedure, the reaction of clofibrate (0.2 mmol), hydrazine hydrate (5 equiv), Rb_2CO_3 (3.0 equiv) and [(ImPyTrippIPr*)Pd(cin)Cl] (5 mol%) in dioxane (0.2 M) for 16 h at 120 °C then with acetylacetone (9 equiv) and 2M TFA in ethanol (4 equiv) for 12 h at 90 °C, afforded after work-up and chromatography the title compound in 64 % yield (38.7 mg). Yellow oil. ^1H NMR (500

MHz, $CDCl_3$) δ 7.28 (d, *J* = 8.9 Hz, 2H), 6.90 (d, *J* = 8.9 Hz, 2H), 5.96 (s, 1H), 4.24 (q, *J* = 7.2 Hz, 2H), 2.28 (s, 3H), 2.25 (s, 3H), 1.61 (s, 6H), 1.25 (t, *J* = 7.1 Hz, 3H). ^{13}C NMR (125 MHz, $CDCl_3$) δ 174.2, 154.8, 148.8, 139.6, 134.4, 126.0, 119.5, 106.6, 79.6, 61.7, 25.5, 14.2, 13.6, 12.3. HRMS calcd for $C_{17}H_{23}N_2O_3$ (M^+ + H) 303.1703, found 303.1675.

3-(2-(3,5-Dimethyl-1*H*-pyrazol-1-yl)-10*H*-phenothiazin-10-yl)-*N*,*N*-dimethylpropan-1-amine (Table 3, 3ad)

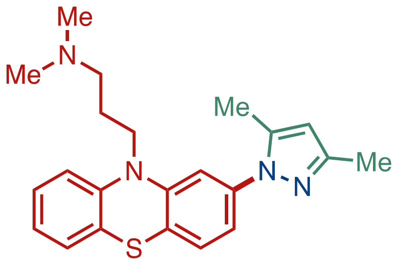

According to the general procedure, the reaction of chlorpromazine hydrochloride (0.2 mmol), hydrazine hydrate (5 equiv), Rb_2CO_3 (4.0 equiv) and [(ImPyTrippIPr*)Pd(cin)Cl] (5 mol%) in dioxane (0.2 M) for 16 h at 120 °C then with acetylacetone (9 equiv) and 2M TFA in ethanol (4 equiv) for 12 h at 90 °C, afforded after work-up and chromatography the title compound in 90 % yield (68.1 mg). Yellow solid. ^{1}H NMR (500 MHz, $CDCl_3$) δ 7.19 – 7.11 (m, 3H), 7.00 (d, *J* = 2.1 Hz, 1H), 6.96 – 6.90 (m, 2H), 6.89 (d, *J* = 8.0 Hz, 1H), 5.98 (s, 1H), 3.93 (t, *J* = 6.9 Hz, 2H), 2.42 (t, *J* = 6.6 Hz, 2H), 2.28 (s, 6H), 2.20 (s, 6H), 1.99 – 1.92 (m, 2H). ^{13}C NMR (125 MHz, $CDCl_3$) δ 149.2, 146.6, 144.8, 139.7, 139.6, 127.8, 127.6, 127.4, 125.1, 124.4, 123.0, 118.7, 116.1, 112.8, 107.2, 57.3, 45.7, 45.7, 25.3, 13.8, 12.7. HRMS calcd for $C_{22}H_{27}N_4S$ (M^+ + H) 379.1951, found 379.1922.

2-(4-(3-(2-(3,5-Dimethyl-1H-pyrazol-1-yl)-10*H*-phenothiazin-10-yl)propyl)piperazin-1-yl)ethan-1-ol (Table 3, 3ae)

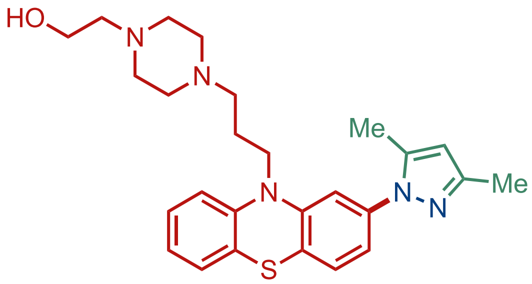

According to the general procedure, the reaction of perphenazine (0.2 mmol), hydrazine hydrate (5 equiv), Rb_2CO_3 (3.0 equiv) and [(ImPyTrippIPr*)Pd(cin)Cl] (5 mol%) in dioxane (0.2 M) for 16 h at 120 °C then with acetylacetone (9 equiv) and 2M TFA in ethanol (4 equiv) for 12 h at 90 °C, afforded after work-up and chromatography the title compound in 76 % yield (70.6 mg). Yellow solid. ^{1}H NMR (500 MHz, $CDCl_3$) δ 7.17 – 7.11 (m, 3H), 6.98 (d, J = 2.0 Hz, 1H), 6.95 – 6.88 (m, 3H), 5.98 (s, 1H), 3.94 (t, J = 6.8 Hz, 2H), 3.59 (t, J = 5.4 Hz, 2H), 2.56 – 2.42 (m, 12H), 2.28 (s, 6H), 1.97 – 1.92 (m, 2H). ^{13}C NMR (125 MHz, $CDCl_3$) δ 149.0, 146.5, 144.7, 139.6, 139.5, 127.6, 127.5, 127.3, 125.0, 124.3, 122.9, 118.6, 116.0, 112.7, 107.1, 59.3, 57.7, 55.6, 53.3, 52.9, 45.4, 24.4, 13.7, 12.6. HRMS calcd for $C_{26}H_{34}N_5SO$ (M^+ + H) 464.2479, found 464.2462. *The final product contains impurities that we could not remove; this may slightly reduce the actual yield.*

4-(4-(4-(3,5-Dimethyl-1*H*-pyrazol-1-yl)phenyl)-4-hydroxypiperidin-1-yl)-1-(4-fluorophenyl) butan-1-one (Table 3, 3af)

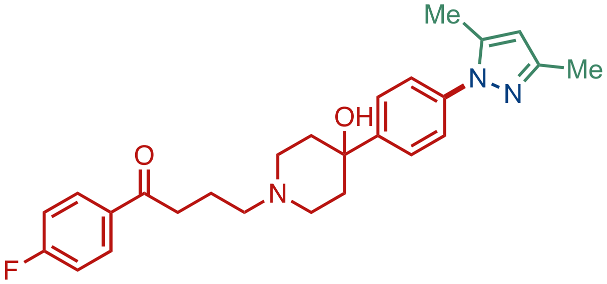

According to the general procedure, the reaction of haloperidol (0.2 mmol), hydrazine hydrate (2 equiv), Rb_2CO_3 (3.0 equiv) and [(ImPyTrippIPr*)Pd(cin)Cl] (5 mol%) in dioxane (0.2 M) for 16 h at 120 °C then with acetylacetone (9 equiv) and 2M TFA in ethanol (4 equiv) for 12 h at 90 °C, afforded

after work-up and chromatography the title compound in 84 % yield (73.1 mg). Yellow solid. ^{1}H NMR (500 MHz, $CDCl_3$) δ 8.05 – 7.97 (m, 2H), 7.50 (d, *J* = 8.7 Hz, 2H), 7.34 (d, *J* = 8.5 Hz, 2H), 7.17 – 7.09 (m, 2H), 5.98 (s, 1H), 3.03 (t, *J* = 6.9 Hz, 2H), 2.95 – 2.84 (m, 2H), 2.66 – 2.52 (m, 4H), 2.28 (s, 3H), 2.28 (s, 3H), 2.22 – 2.12 (m, 2H), 2.09 – 2.02 (m, 2H), 1.77 – 1.69 (m, 2H). ^{13}C NMR (125 MHz, $CDCl_3$) δ 198.3, 165.8 (d, *J* = 254.8 Hz), 149.1, 147.4, 139.6, 138.7, 133.6 (d, *J* = 3.6 Hz), 130.8 (d, *J* = 9.1 Hz), 125.4, 124.7, 115.8 (d, *J* = 21.8 Hz), 107.0, 70.9, 57.7, 49.4, 37.9, 36.3, 13.6, 12.5. ^{19}F NMR (471 MHz, $CDCl_3$) δ -105.43. HRMS calcd for $C_{26}H_{30}FN_3O_2Na$ (M^+ + Na) 458.2214, found 458.2203.

1-(4,4-Bis(4-fluorophenyl)butyl)-4-(4-(3,5-dimethyl-1*H*-pyrazol-1-yl)-3-(trifluoromethyl) phenyl)piperidin-4-ol (Table 3, 3ag)

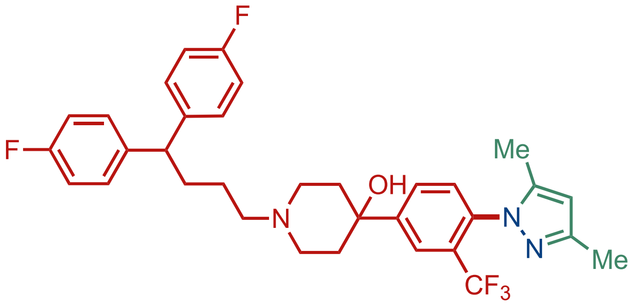

According to the general procedure, the reaction of penfluridol (0.2 mmol), hydrazine hydrate (5 equiv), Rb_2CO_3 (3.0 equiv) and [(ImPyTrippIPr*)Pd(cin)Cl] (5 mol%) in dioxane (0.2 M) for 16 h at 120 °C then with acetylacetone (9 equiv) and 2M TFA in ethanol (4 equiv) for 12 h at 90 °C, afforded after work-up and chromatography the title compound in 87 % yield (98 mg). Yellow solid. ^{1}H NMR (500 MHz, $CDCl_3$) δ 7.88 (s, 1H), 7.66 (d, *J* = 8.2 Hz, 1H), 7.21 – 7.15 (m, 5H), 6.99 – 6.94 (m, 4H), 5.96 (s, 1H), 3.89 (t, *J* = 7.9 Hz, 1H), 2.92 – 2.73 (m, 2H), 2.64 – 2.37 (m, 4H), 2.30 (s, 3H), 2.24 – 2.07 (m, 2H), 2.06 – 2.01 (m, 5H), 1.61 (d, *J* = 12.4 Hz, 2H), 1.57 – 1.46 (m, 2H). ^{13}C NMR (125 MHz, $CDCl_3$) δ 161.6 (d, *J* = 244.3 Hz), 151.0, 149.1, 141.8, 140.5, 135.8, 130.5, 129.2 (d, *J* = 7.7 Hz), 129.0, 128.2 (q, *J* = 31.1 Hz), 123.9 (q, *J* = 5.0 Hz), 123.9 (q, *J* = 272.5 Hz), 115.5 (d, *J* = 21.3 Hz), 105.7, 71.0, 58.5, 49.9, 49.4, 38.2, 33.9, 25.1, 13.5, 11.4. ^{19}F NMR (471 MHz, $CDCl_3$) δ -60.43, -117.00. HRMS calcd for $C_{33}H_{35}F_5N_3O$ (M^+ + H) 584.2695, found 584.2667. *The final product contains impurities that we could not remove; this may slightly reduce the actual yield.*

2-(3,5-Dimethyl-1*H*-pyrazol-1-yl)-11-(4-methylpiperazin-1-yl)dibenzo[*b*,*f*][1,4]oxazepane (Table 3, 3ah)

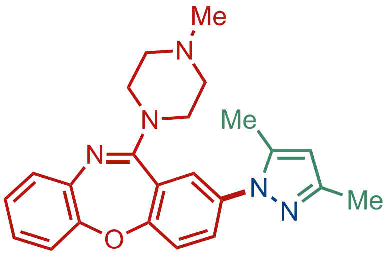

According to the general procedure, the reaction of loxapine succinate (0.2 mmol), hydrazine hydrate (5 equiv), Rb_2CO_3 (5.0 equiv) and [(ImPyTrippIPr*)Pd(cin)Cl] (5 mol%) in dioxane (0.2 M) for 16 h at 120 °C then with acetylacetone (9 equiv) and 2M TFA in ethanol (4 equiv) for 12 h at 90 °C, afforded after work-up and chromatography the title compound in 88 % yield (68.1 mg). White solid. ^{1}H NMR (500 MHz, $CDCl_3$) δ 7.50 (dd, *J* = 8.6, 2.6 Hz, 1H), 7.38 (d, *J* = 2.6 Hz, 1H), 7.31 (d, *J* = 8.6 Hz, 1H), 7.17 – 7.11 (m, 2H), 7.09 (t, *J* = 7.6 Hz, 1H), 6.99 (t, *J* = 7.4 Hz, 1H), 5.98 (s, 1H), 3.72 – 3.50 (m, 4H), 2.64 – 2.44 (m, 4H), 2.35 (s, 3H), 2.27 (s, 6H). ^{13}C NMR (125 MHz, $CDCl_3$) δ 159.7, 159.4, 152.0, 149.5, 140.5, 139.4, 137.0, 129.0, 127.2, 125.9, 125.5, 124.5, 124.3, 122.1, 120.3, 107.3, 55.0, 46.2, 13.6, 12.6. HRMS calcd for $C_{23}H_{25}N_5ONa$ (M^+ + Na) 410.1951, found 410.1939.

2-(3,5-Dimethyl-1*H*-pyrazol-1-yl)-11-(piperazin-1-yl)dibenzo[*b*,*f*][1,4]oxazepane (Table 3, 3ai)

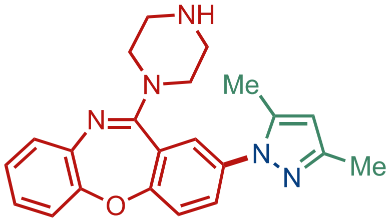

According to the general procedure, the reaction of amoxapine (0.2 mmol), hydrazine hydrate (5 equiv), Rb_2CO_3 (3.0 equiv) and [(ImPyTrippIPr*)Pd(cin)Cl] (5 mol%) in dioxane (0.2 M) for 16 h at 120 °C then with acetylacetone (9 equiv) and 2M TFA in ethanol (4 equiv) for 12 h at 90 °C, afforded after work-up and chromatography the title compound in 93 % yield (69.3 mg). White solid. ^{1}H NMR (500 MHz, $CDCl_3$) δ 7.50 (dd, *J* = 8.6, 2.3 Hz, 1H), 7.39 (d, *J* = 2.3 Hz, 1H), 7.31 (d, *J* = 8.6 Hz, 1H),

7.14 (t, *J* = 8.4 Hz, 2H), 7.08 (t, *J* = 7.5 Hz, 1H), 6.99 (t, *J* = 7.0 Hz, 1H), 5.98 (s, 1H), 3.57 (s, 5H), 3.01 (s, 4H), 2.26 (s, 6H). ^{13}C NMR (125 MHz, $CDCl_3$) δ 159.65, 159.54, 151.97, 149.49, 140.35, 139.42, 137.04, 128.97, 127.19, 125.86, 125.44, 124.64, 124.20, 122.10, 120.27, 107.39, 48.28, 45.62, 13.59, 12.52. HRMS calcd for $C_{23}H_{25}N_5ONa$ (M^+ + Na) 410.1951, found 410.1939.

(*Z*)-3-(2-(3,5-Dimethyl-1*H*-pyrazol-1-yl)-9*H*-thioxanthen-9-ylidene)-*N*,*N*-dimethylpropan-1-amine (Table 3, 3aj)

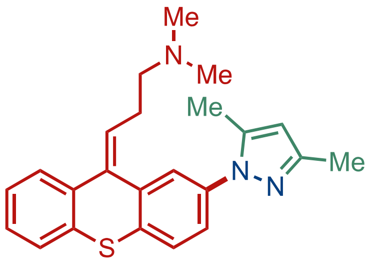

According to the general procedure, the reaction of chlorprothixene (0.2 mmol), hydrazine hydrate (5 equiv), Rb_2CO_3 (3.0 equiv) and [(ImPyTrippIPr*)Pd(cin)Cl] (5 mol%) in dioxane (0.2 M) for 16 h at 120 °C then with acetylacetone (9 equiv) and 2M TFA in ethanol (4 equiv) for 12 h at 90 °C, afforded after work-up and chromatography the title compound in 71 % yield (53.2 mg). Yellow solid. ^{1}H NMR (500 MHz, $CDCl_3$) δ 7.54 – 7.48 (m, 2H), 7.47 (d, *J* = 7.6 Hz, 1H), 7.37 (d, *J* = 6.9 Hz, 1H), 7.31 (dd, *J* = 8.4, 2.3 Hz, 1H), 7.29 – 7.26 (m, 1H), 7.24 – 7.19 (m, 1H), 5.99 (s, 1H), 5.94 (t, *J* = 7.2 Hz, 1H), 2.68 – 2.61 (m, 2H), 2.49 (t, *J* = 7.4 Hz, 2H), 2.33 (s, 3H), 2.29 (s, 3H), 2.23 (s, 6H). ^{13}C NMR (125 MHz, $CDCl_3$) δ 149.3, 139.4, 138.3, 136.2, 134.8, 132.9, 131.6, 130.7, 127.3, 127.3, 127.0, 126.0, 125.9, 124.7, 123.4, 107.3, 59.5, 45.4, 28.1, 13.7, 12.7. HRMS calcd for $C_{23}H_{26}N_3S$ (M^+ + H) 376.1842, found 376.1817. *The final product contains impurities that we could not remove; this may slightly reduce the actual yield.*

4-(3,5-Dimethyl-1*H*-pyrazol-1-yl)-*N*-(propylcarbamoyl)benzenesulfonamide (Table 3, 3ak)

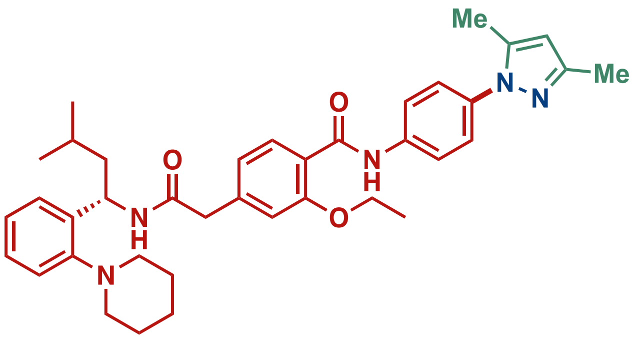

According to the general procedure, the reaction of repaglinide amide (0.2 mmol), hydrazine hydrate (5 equiv), Rb_2CO_3 (3.0 equiv) and [(ImPyTrippIPr*)Pd(cin)Cl] (5 mol%) in dioxane (0.2 M) for 16 h at 120 °C then with acetylacetone (9 equiv) and 2M TFA in ethanol (4 equiv) for 12 h at 90 °C, afforded after work-up and chromatography the title compound in 92 % yield (114.2 mg). White solid. ^{1}H NMR (600 MHz, $CDCl_3$) δ 10.17 (s, 1H), 8.22 (d, *J* = 7.9 Hz, 1H), 7.74 (d, *J* = 8.4 Hz, 2H), 7.40 (d, *J* = 8.4 Hz, 2H), 7.23 – 7.14 (m, 2H), 7.12 – 7.08 (m, 1H), 7.08 – 7.03 (m, 1H), 6.97 (d, *J* = 8.0 Hz, 1H), 6.93 (s, 1H), 6.92 – 6.81 (m, 1H), 5.98 (s, 1H), 5.37 (q, *J* = 8.1 Hz, 1H), 4.24 – 4.16 (m, 1H), 4.15 – 4.07 (m, 1H), 3.56 (s, 2H), 3.05 – 2.81 (m, 2H), 2.71 – 2.55 (m, 2H), 2.30 (s, 3H), 2.29 (s, 3H), 1.75 – 1.70 (m, 2H), 1.67 – 1.56 (m, 7H), 1.56 – 1.48 (m, 2H), 1.45 – 1.39 (m, 1H), 0.92 (d, *J* = 4.6 Hz, 6H). ^{13}C NMR (150 MHz, $CDCl_3$) δ 168.80, 163.17, 156.96, 152.66, 148.96, 141.48, 139.59, 138.77, 137.84, 135.81, 132.94, 128.06, 127.94, 125.60, 125.23, 123.03, 122.41, 120.28, 113.18, 106.84, 65.25, 50.10, 46.80, 44.16, 26.91, 25.47, 24.25, 22.90, 22.68, 15.00, 13.65, 12.45. HRMS calcd for $C_{38}H_{48}N_5O_3$ (M^+ + H) 622.3752, found 622.3724.

8-(3,5-Dimethyl-1*H*-pyrazol-1-yl)-11-(piperidin-4-ylidene)-6,11-dihydro-5*H*-benzo[5,6]cyclo hepta[1,2-*b*]pyridine (Table 3, 3al)

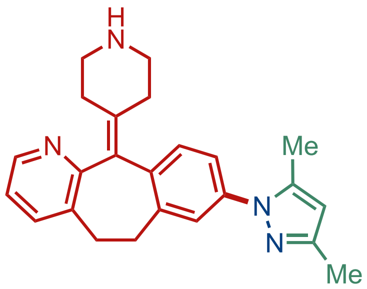

According to the general procedure, the reaction of desloratadine (0.2 mmol), hydrazine hydrate (5 equiv), Rb_2CO_3 (3.0 equiv) and [(ImPyTrippIPr*)Pd(cin)Cl] (5 mol%) in dioxane (0.2 M) for 16 h at 120 °C then with acetylacetone (9 equiv) and 2M TFA in ethanol (4 equiv) for 12 h at 90 °C, afforded after work-up and chromatography the title compound in 86 % yield (63.9 mg). Yellow solid. ^1H NMR (500 MHz, $CDCl_3$) δ 8.41 (d, *J* = 4.8 Hz, 1H), 7.44 (d, *J* = 7.4 Hz, 1H), 7.29 (d, *J* = 2.1 Hz, 1H), 7.26 – 7.22 (m, 1H), 7.16 (dd, *J* = 8.0, 2.1 Hz, 1H), 7.09 (dd, *J* = 7.7, 4.8 Hz, 1H), 5.96 (s, 1H), 3.52 – 3.44 (m, 1H), 3.42 – 3.36 (m, 1H), 3.17 – 3.04 (m, 2H), 2.91 – 2.84 (m, 2H), 2.79 – 2.63 (m, 3H), 2.54 – 2.48 (m, 1H), 2.44 – 2.34 (m, 3H), 2.28 (s, 3H), 2.27 (s, 3H). ^{13}C NMR (500 MHz, $CDCl_3$) δ 157.5, 149.0, 146.8, 139.4, 139.1, 139.1, 138.6, 138.3, 137.6, 133.8, 133.7, 129.9, 125.5, 122.3, 122.1, 107.0, 47.8, 32.0, 31.7, 13.6, 12.5. HRMS calcd for $C_{24}H_{27}N_4$ (M^+ + H) 371.2230, found 371.2209. *The final product contains impurities that we could not remove; this may slightly reduce the actual yield.*

Ethyl 4-(8-(3,5-dimethyl-1*H*-pyrazol-1-yl)-5,6-dihydro-11*H*-benzo[5,6]cyclohepta[1,2-*b*]pyridin-11-ylidene)piperidine-1-carboxylate (Table 3, 3am)

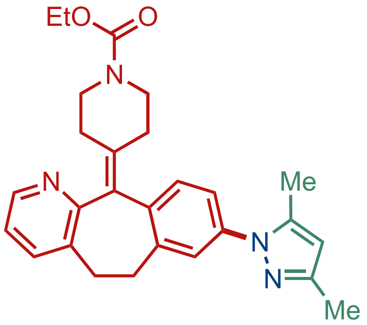

According to the general procedure, the reaction of loratadine (0.2 mmol), hydrazine hydrate (5 equiv), Rb_2CO_3 (3.0 equiv) and [(ImPyTrippIPr*)Pd(cin)Cl] (5 mol%) in dioxane (0.2 M) for 16 h at 120 °C then with acetylacetone (9 equiv) and 2M TFA in ethanol (4 equiv) for 12 h at 90 °C, afforded after work-up and chromatography the title compound in 90 % yield (79.5 mg). Yellow solid. ^1H NMR (500 MHz, $CDCl_3$) δ 8.41 (d, *J* = 3.2 Hz, 1H), 7.45 (d, *J* = 7.0 Hz, 1H), 7.29 (d, *J* = 2.1 Hz, 1H), 7.24 (d, *J* = 8.1 Hz, 1H), 7.16 (dd, *J* = 8.1, 2.2 Hz, 1H), 7.10 (dd, *J* = 7.7, 4.8 Hz, 1H), 5.96 (s, 1H), 4.13 (q, *J* = 7.1 Hz, 2H), 3.91 – 3.73 (m, 2H), 3.49 – 3.42 (m, 1H), 3.40 – 3.32 (m, 1H), 3.17 – 3.09 (m, 2H), 2.92 – 2.82 (m, 2H), 2.53 – 2.46 (m, 1H), 2.41 – 2.31 (m, 3H), 2.28 (s, 3H), 2.27 (s, 3H), 1.25 (t, *J* = 7.1 Hz, 3H). ^{13}C NMR (125 MHz, $CDCl_3$) δ 157.1, 155.5, 148.9, 146.6, 139.3, 139.0, 138.9, 138.3, 137.6,

137.6, 134.5, 133.6, 129.7, 125.4, 122.2, 122.0, 106.9, 61.3, 44.8, 31.8, 31.6, 14.7, 13.5, 12.4. HRMS calcd for $C_{27}H_{31}N_4O_2$ (M^+ + H) 443.2442, found 443.2422.

N-(4-(3,5-Dimethyl-1H-pyrazol-1-yl)phenyl)-4-(N,N-dipropylsulfamoyl)benzamide (Table 3, 3an)

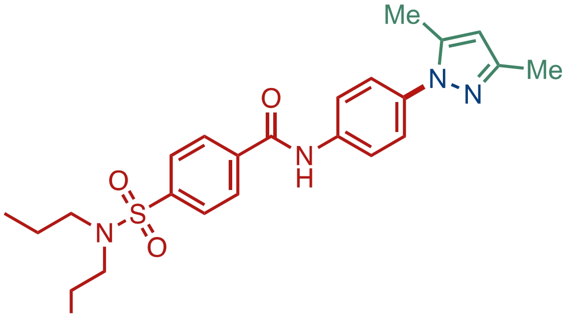

According to the general procedure, the reaction of probenecid amide (0.2 mmol), hydrazine hydrate (5 equiv), Rb_2CO_3 (3.0 equiv) and [(ImPyTrippIPr*)Pd(cin)Cl] (5 mol%) in dioxane (0.2 M) for 16 h at 120 °C then with acetylacetone (9 equiv) and 2M TFA in ethanol (4 equiv) for 12 h at 90 °C, afforded after work-up and chromatography the title compound in 73 % yield (66.6 mg). White solid. ^{1}H NMR (500 MHz, $CDCl_3$) δ 8.40 (s, 1H), 7.98 (d, *J* = 8.3 Hz, 2H), 7.85 (d, *J* = 8.3 Hz, 2H), 7.74 (d, *J* = 8.8 Hz, 2H), 7.42 (d, *J* = 8.7 Hz, 2H), 6.01 (s, 1H), 3.10 (t, *J* = 7.5 Hz, 4H), 2.31 (s, 3H), 2.29 (s, 3H), 1.59 – 1.51 (m, 4H), 0.88 (t, *J* = 7.4 Hz, 6H). ^{13}C NMR (125 MHz, $CDCl_3$) δ 164.8, 149.2, 143.3, 139.8, 138.5, 136.9, 128.1, 127.5, 125.6, 121.0, 107.1, 50.1, 22.1, 13.6, 12.5, 11.3. HRMS calcd for $C_{24}H_{31}N_4O_3S$ (M^+ + H) 455.2111, found 455.2094.

2-(3-Cyano-4-isobutoxyphenyl)-N-(4-(3,5-dimethyl-1H-pyrazol-1-yl)phenyl)-4-methyl thiazole-5-carboxamide (Table 3, 3ao)

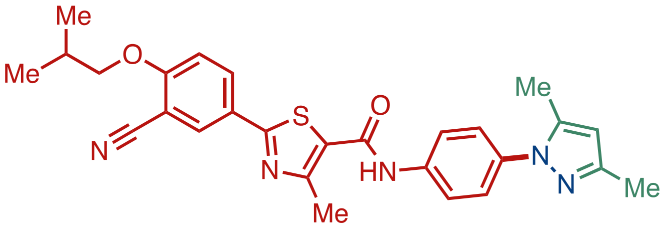

According to the general procedure, the reaction of febuxostat amide (0.2 mmol), hydrazine hydrate (5 equiv), Rb_2CO_3 (3.0 equiv) and [(ImPyTrippIPr*)Pd(cin)Cl] (5 mol%) in dioxane (0.2 M) for 16 h at 120 °C then with acetylacetone (9 equiv) and 2M TFA in ethanol (4 equiv) for 12 h at 90 °C, afforded after work-up and chromatography the title compound in 76 % yield (73.8 mg). Yellow solid. ^{1}H NMR (500 MHz, $CDCl_3$) δ 8.16 (d, J = 2.3 Hz, 1H), 8.08 (dd, J = 8.8, 2.3 Hz, 1H), 7.70 (s, 1H), 7.67 (d, J = 8.7 Hz, 2H), 7.43 (d, J = 8.8 Hz, 2H), 7.03 (d, J = 8.9 Hz, 1H), 6.00 (s, 1H), 3.91 (d, J = 6.5 Hz, 2H), 2.80 (s, 3H), 2.30 (s, 3H), 2.29 (s, 3H), 2.23 – 2.18 (m, 1H), 1.09 (d, J = 6.7 Hz, 6H). ^{13}C NMR (125 MHz, $CDCl_3$) δ 165.2, 162.7, 159.9, 157.6, 149.2, 139.7, 136.7, 136.5, 132.7, 132.2, 125.9, 125.6, 120.9, 115.5, 112.8, 107.2, 103.2, 75.9, 28.3, 19.2, 17.7, 13.6, 12.5. HRMS calcd for $C_{27}H_{28}N_5O_2S$ (M^+ + H) 486.1958, found 486.1944.

_N_-(4-(3,5-Dimethyl-1_H_-pyrazol-1-yl)phenyl)-9-fluoro-5-methyl-1-oxo-6,7-dihydro-1_H_,5_H_-pyrido[3,2,1-_ij_]quinoline-2-carboxamide (Table 3, 3ap)

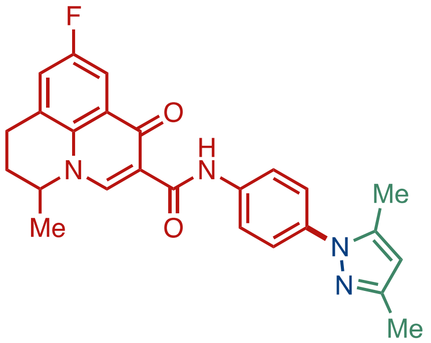

According to the general procedure, the reaction of flumequine amide (0.2 mmol), hydrazine hydrate (5 equiv), Rb_2CO_3 (3.0 equiv) and [(ImPyTrippIPr*)Pd(cin)Cl] (5 mol%) in dioxane (0.2 M) for 16 h at 120 °C then with acetylacetone (9 equiv) and 2M TFA in ethanol (4 equiv) for 12 h at 90 °C, afforded after work-up and chromatography the title compound in 88 % yield (75.5 mg). White solid. ^{1}H NMR (500 MHz, $CDCl_3$) δ 12.31 (s, 1H), 8.81 (s, 1H), 8.03 (dd, J = 8.9, 2.8 Hz, 1H), 7.72 (d, J = 8.8 Hz, 2H), 7.34 – 7.27 (m, 3H), 4.63 – 4.53 (m, 1H), 3.26 – 3.14 (m, 1H), 3.09 – 3.00 (m, 1H), 2.33 – 2.23 (m, 1H), 2.22 – 2.14 (m, 1H), 1.52 (d, J = 6.8 Hz, 3H). ^{13}C NMR (125 MHz, $CDCl_3$) δ 176.0 (d, J = 2.8 Hz), 163.2, 159.7 (d, J = 248.2 Hz), 145.9, 137.5, 132.6, 129.8 (d, J = 7.6 Hz), 129.6 (d, J = 7.6 Hz), 129.0, 128.7, 121.7, 120.9 (d, J = 24.8 Hz), 111.3, 110.1 (d, J = 22.9 Hz), 57.7, 26.4, 22.3,

20.7. ^{19}F NMR (471 MHz, Chloroform-*d*) δ -114.74. HRMS calcd for $C_{25}H_{24}FN_4O_2$ (M^+ + H) 431.1878, found 431.1861.

N-(4-(3,5-Dimethyl-1*H*-pyrazol-1-yl)phenyl)-2-(11-oxo-6,11-dihydrodibenzo[*b*,*e*]oxepin-2-yl)acetamide (Table 3, 3aq)

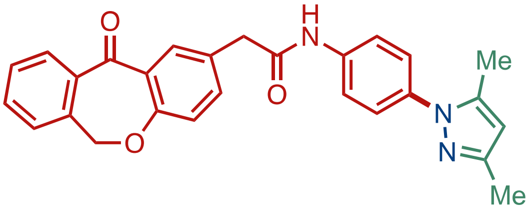

According to the general procedure, the reaction of isoxepac amide (0.2 mmol), hydrazine hydrate (5 equiv), Rb_2CO_3 (3.0 equiv) and [(ImPyTrippIPr*)Pd(cin)Cl] (5 mol%) in dioxane (0.2 M) for 16 h at 120 °C then with acetylacetone (9 equiv) and 2M TFA in ethanol (4 equiv) for 12 h at 90 °C, afforded after work-up and chromatography the title compound in 70 % yield (61.1 mg). Colorless solid. ^{1}H NMR (500 MHz, $CDCl_3$) δ 8.17 (d, *J* = 2.4 Hz, 1H), 7.90 (d, *J* = 7.7 Hz, 1H), 7.58 (t, *J* = 7.4 Hz, 1H), 7.56 – 7.47 (m, 4H), 7.41 (s, 1H), 7.39 (d, *J* = 7.5 Hz, 1H), 7.34 (d, *J* = 8.5 Hz, 2H), 7.10 (d, *J* = 8.4 Hz, 1H), 5.97 (s, 1H), 5.22 (s, 2H), 3.75 (s, 2H), 2.28 (s, 3H), 2.25 (s, 3H). ^{13}C NMR (500 MHz, $CDCl_3$) δ 168.8, 160.8, 148.9, 140.4, 139.6, 136.7, 136.4, 136.0, 135.5, 133.0, 132.6, 129.5, 129.4, 128.0, 127.9, 125.4, 125.3, 121.8, 120.3, 106.8, 73.7, 43.7, 13.5, 12.3. HRMS calcd for $C_{27}H_{24}N_3O_3$ (M^+ + H) 438.1812, found 438.1799.

5-(3,5-Dimethyl-1*H*-pyrazol-1-yl)-6'-methyl-3-(4-(methylsulfonyl)phenyl)-2,3'-bipyridine (Table 3, 3ar)

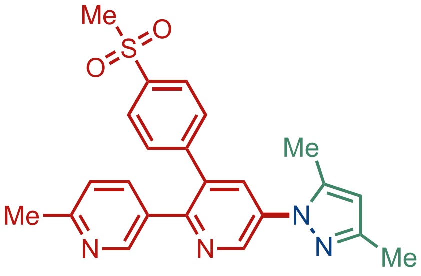

According to the general procedure, the reaction of etoricoxib (0.2 mmol), hydrazine hydrate (5 equiv), Rb_2CO_3 (3.0 equiv) and [(ImPyTrippIPr*)Pd(cin)Cl] (5 mol%) in dioxane (0.2 M) for 16 h at 120 °C then with acetylacetone (9 equiv) and 2M TFA in ethanol (4 equiv) for 12 h at 90 °C, afforded after work-up and chromatography the title compound in 73 % yield (61 mg). White solid. ^{1}H NMR (500 MHz, $CDCl_3$) δ 8.85 (d, *J* = 2.4 Hz, 1H), 8.44 (d, *J* = 2.3 Hz, 1H), 7.93 (d, *J* = 2.4 Hz, 1H), 7.89 (d, *J* = 8.2 Hz, 2H), 7.61 (dd, *J* = 8.0, 2.4 Hz, 1H), 7.45 (d, *J* = 8.2 Hz, 2H), 7.10 (d, *J* = 8.0 Hz, 1H), 6.08 (s, 1H), 3.07 (s, 3H), 2.54 (s, 3H), 2.45 (s, 3H), 2.30 (s, 3H). ^{13}C NMR (125 MHz, $CDCl_3$) δ 158.5, 152.3, 150.8, 150.0, 144.4, 144.1, 140.1, 140.0, 137.6, 135.8, 134.9, 134.0, 131.7, 130.6, 128.0, 122.9, 108.6, 44.6, 24.4, 13.6, 12.8. HRMS calcd for $C_{23}H_{23}N_4O_2S$ (M^+ + H) 419.1536, found 419.1519.

N^4-(7-(3,5-Dimethyl-1*H*-pyrazol-1-yl)quinolin-4-yl)-N^1,N^1-diethylpentane-1,4-diamine (Table 3, 3as)

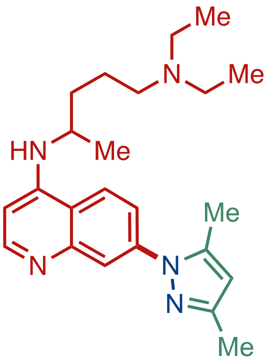

According to the general procedure, the reaction of chloroquine phosphate (0.2 mmol), hydrazine hydrate (5 equiv), Rb_2CO_3 (5.0 equiv) and [(ImPyTrippIPr*)Pd(cin)Cl] (5 mol%) in dioxane (0.2 M) for 16 h at 120 °C then with acetylacetone (9 equiv) and 2M TFA in ethanol (4 equiv) for 12 h at 90 °C, afforded after work-up and chromatography the title compound in 89 % yield (67.5 mg). Yellow solid. ^{1}H NMR (500 MHz, $CDCl_3$) δ 8.54 (d, *J* = 5.5 Hz, 1H), 7.89 (d, *J* = 2.3 Hz, 1H), 7.86 (d, *J* = 9.0 Hz, 1H), 7.69 (dd, *J* = 9.0, 2.3 Hz, 1H), 6.44 (d, *J* = 5.5 Hz, 1H), 6.04 (s, 1H), 5.42 (s, 1H), 3.80 – 3.69 (m, 1H), 2.58 (q, *J* = 7.2 Hz, 4H), 2.50 (t, *J* = 6.9 Hz, 2H), 2.43 (s, 3H), 2.33 (s, 3H), 1.81 – 1.74 (m, 1H), 1.69 – 1.62 (m, 3H), 1.34 (d, *J* = 6.3 Hz, 3H), 1.04 (t, *J* = 7.2 Hz, 6H). ^{13}C NMR (125 MHz, $CDCl_3$) δ 151.9, 149.6, 149.2, 148.8, 140.4, 140.1, 123.0, 121.7, 121.2, 117.5, 107.8, 99.4, 52.6, 48.4, 46.9, 34.6, 23.7, 20.3, 13.7, 13.0, 11.3. HRMS calcd for $C_{23}H_{34}N_5$ (M^+ + H) 380.2809, found 380.2788.

The final product contains impurities that we could not remove; this may slightly reduce the actual yield.

N-(3-(3,5-Dimethyl-1H-pyrazol-1-yl)-4-fluorophenyl)-7-methoxy-6-(3-morpholinopropoxy) quinazolin-4-amine (Table 3, 3at)

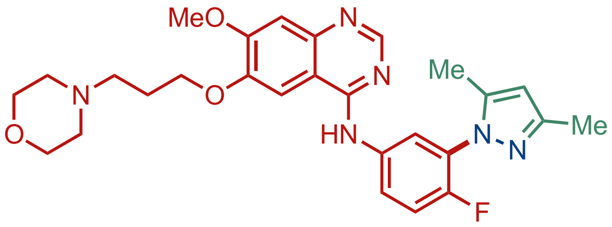

According to the general procedure, the reaction of gefitinib (0.2 mmol), hydrazine hydrate (5 equiv), Rb_2CO_3 (3.0 equiv) and [(ImPyTrippIPr*)Pd(cin)Cl] (5 mol%) in dioxane (0.2 M) for 16 h at 120 °C then with acetylacetone (9 equiv) and 2M TFA in ethanol (4 equiv) for 12 h at 90 °C, afforded after work-up and chromatography the title compound in 86 % yield (87.1 mg). Yellow solid. ^{1}H NMR (500 MHz, $CDCl_3$) δ 9.10 (s, 1H), 8.54 (s, 1H), 7.82 – 7.70 (m, 1H), 7.44 (dd, *J* = 6.7, 2.7 Hz, 1H), 7.39 (s, 1H), 7.20 (s, 1H), 7.07 (t, *J* = 9.3 Hz, 1H), 6.04 (s, 1H), 3.97 (s, 3H), 3.87 (t, *J* = 6.5 Hz, 2H), 3.72 (t, *J* = 4.7 Hz, 4H), 2.52 – 2.41 (m, 6H), 2.25 (s, 3H), 2.22 (s, 3H), 2.00 (t, *J* = 7.1 Hz, 2H). ^{13}C NMR (125 MHz, $CDCl_3$) δ 156.8, 155.1, 153.4,153.3 (d, *J* = 248.1 Hz), 150.1, 149.0, 147.6, 142.3, 135.8 (d, *J* = 3.0 Hz), 126.4 (d, *J* = 13.3 Hz), 124.8 (d, *J* = 7.3 Hz), 123.2, 116.4 (d, *J* = 21.0 Hz), 109.5, 107.7, 106.4, 101.9, 67.2, 67.0, 56.3, 55.5, 53.8, 26.3, 13.8, 11.4 (d, *J* = 2.7 Hz). ^{19}F NMR (471 MHz, Chloroform-*d*) δ -127.85. HRMS calcd for $C_{27}H_{32}N_6FO_3$ (M^+ + H) 371.2230, found 371.2209.

(S)-4-((3-(3,5-Dimethyl-1H-pyrazol-1-yl)-4-methoxybenzyl)amino)-2-(2-(hydroxymethyl) pyrrolidin-1-yl)-N-(pyrimidin-2-ylmethyl)pyrimidine-5-carboxamide (Table 3, 3au)

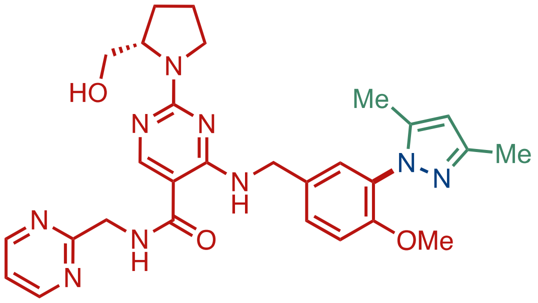

According to the general procedure, the reaction of avanafil (0.2 mmol), hydrazine hydrate (5 equiv), Rb_2CO_3 (3.0 equiv) and [(ImPyTrippIPr*)Pd(cin)Cl] (5 mol%) in dioxane (0.2 M) for 16 h at 120 °C then with acetylacetone (9 equiv) and 2M TFA in ethanol (4 equiv) for 12 h at 90 °C, afforded after work-up and chromatography the title compound in 69 % yield (75.2 mg). Colorless solid. ^1H NMR (600 MHz, $CDCl_3$) δ 9.19 (s, 1H), 8.72 (d, *J* = 4.8 Hz, 2H), 8.36 (d, *J* = 77.6 Hz, 1H), 7.45 – 7.29 (m, 3H), 7.22 (t, *J* = 4.9 Hz, 1H), 6.92 (d, *J* = 8.2 Hz, 1H), 5.93 (s, 1H), 4.83 – 4.49 (m, 4H), 4.28 – 4.16 (m, 1H), 3.75 (s, 3H), 3.72 – 3.30 (m, 4H), 2.26 (s, 3H), 2.07 (s, 3H), 2.04 – 1.62 (m, 4H). ^{13}C NMR (125 MHz, $CDCl_3$) δ 167.2, 165.8, 161.3, 160.0, 157.4, 153.7, 153.5, 149.0, 141.6, 131.8, 129.1, 128.9, 128.6, 128.0, 119.8, 112.1, 105.4, 99.4, 68.2, 61.4, 56.0, 48.7, 45.2, 43.4, 30.0, 23.8, 13.8, 11.4. HRMS calcd for $C_{28}H_{34}N_9O_3$ (M^+ + H) 544.2779, found 544.2759.

(6*R*,12a*R*)-6-(Benzo[*d*][1,3]dioxol-5-yl)-7-(2-(3,5-dimethyl-1*H*-pyrazol-1-yl)benzyl)-2-methyl-2,3,6,7,12,12a-hexahydropyrazino[1',2':1,6]pyrido[3,4-*b*]indole-1,4-dione (Table 3, 3av)

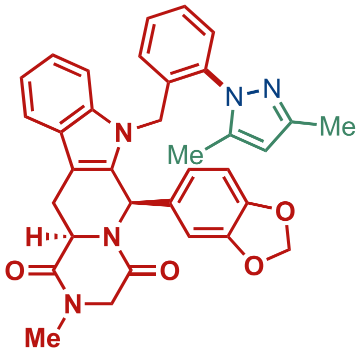

According to the general procedure, the reaction of tadalafil derivative (0.2 mmol), hydrazine hydrate (5 equiv), Rb_2CO_3 (3.0 equiv) and [(ImPyTrippIPr*)Pd(cin)Cl] (5 mol%) in dioxane (0.2 M) for 16 h at 120 °C then with acetylacetone (9 equiv) and 2M TFA in ethanol (4 equiv) for 12 h at 90 °C, afforded after work-up and chromatography the title compound in 92 % yield (105.2 mg). Colorless solid. ^1H NMR (500 MHz, $CDCl_3$) δ 7.55 (d, *J* = 7.3 Hz, 1H), 7.28 (t, *J* = 6.9 Hz, 1H), 7.20 – 7.13 (m, 5H), 6.76 (s, 1H), 6.68 (s, 1H), 6.63 – 6.59 (m, 2H), 6.56 (d, *J* = 8.1 Hz, 1H), 5.90 (dd, *J* = 8.9, 1.4 Hz, 2H), 5.89 (s, 1H), 4.95 (d, *J* = 17.9 Hz, 1H), 4.64 (d, *J* = 17.9 Hz, 1H), 4.33 (dd, *J* = 11.9, 4.4 Hz, 1H), 4.11 (d, *J* = 17.8 Hz, 1H), 3.95 (d, *J* = 17.7 Hz, 1H), 3.58 (dd, *J* = 15.5, 4.3 Hz, 1H), 3.02 – 2.94 (m, 4H), 2.22 (s, 3H), 2.05 (s, 3H). ^{13}C NMR (125 MHz, $CDCl_3$) δ 165.6, 161.3, 149.1, 148.1, 148.0, 140.2,

137.4, 136.8, 134.9, 131.3, 131.1, 129.3, 127.9, 127.6, 127.3, 126.2, 122.7, 122.5, 120.1, 118.5, 110.2, 109.2, 108.7, 108.3, 106.0, 101.4, 52.0, 51.6, 51.0, 43.4, 33.5, 27.7, 13.5, 11.6. HRMS calcd for $C_{34}H_{32}N_5O_4$ (M^+ + H) 574.2449, found 574.2464.

4-(3-(4-(Cyclopropanecarbonyl)piperazine-1-carbonyl)-4-fluorobenzyl)-2-(2-(3,5-dimethyl-1*H*-pyrazol-1-yl)benzyl)phthalazin-1(2*H*)-one (Table 3, 3aw)

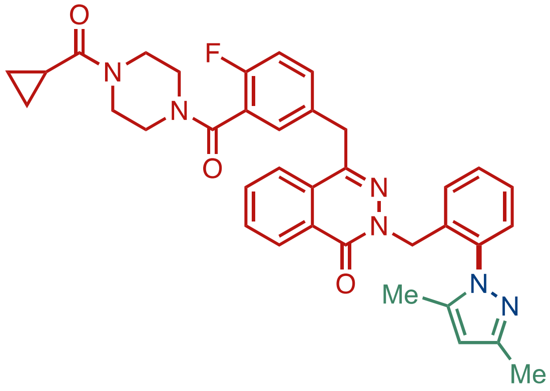

According to the general procedure, the reaction of olaparib derivative (0.2 mmol), hydrazine hydrate (5 equiv), Rb_2CO_3 (3.0 equiv) and [(ImPyTrippIPr*)Pd(cin)Cl] (5 mol%) in dioxane (0.2 M) for 16 h at 120 °C then with acetylacetone (9 equiv) and 2M TFA in ethanol (4 equiv) for 12 h at 90 °C, afforded after work-up and chromatography the title compound in 72 % yield (90 mg). Colorless solid. ^{1}H NMR (600 MHz, $CDCl_3$) δ 8.43 – 8.33 (m, 1H), 7.74 – 7.60 (m, 3H), 7.37 – 7.32 (m, 2H), 7.31 – 7.21 (m, 4H), 6.99 (t, *J* = 8.7 Hz, 1H), 5.99 (s, 1H), 5.21 (s, 2H), 4.21 (s, 2H), 4.01 – 3.63 (m, 4H), 3.62 – 3.47 (m, 2H), 3.46 – 2.68 (m, 2H), 2.23 (s, 3H), 2.17 (s, 3H), 1.77 – 1.59 (m, 1H), 0.99 (s, 2H), 0.84 – 0.70 (m, 2H). ^{13}C NMR (125 MHz, $CDCl_3$) δ 172.41, 165.30, 159.23, 158.04, 156.07, 148.93, 144.60, 141.01, 138.22, 135.71, 134.6 (d, *J* = 6.3 Hz), 133.20, 131.74 (d, *J* = 8.2 Hz), 131.57, 129.25, 129.07, 128.77, 128.42, 128.13, 128.02, 127.64, 127.38, 124.85, 123.7 (d, *J* = 13.5 Hz), 116.24 (d, *J* = 22.7 Hz), 105.63, 50.62, 46.89, 45.24, 42.33, 37.81, 13.65, 11.64, 11.13, 7.82. ^{19}F NMR (470 MHz, $CDCl_3$) δ -117.79. HRMS calcd for $C_{36}H_{36}FN_6O_3$ (M^+ + H) 619.2827, found 619.2843.

One-pot Synthesis of Heterocycles

2,3,4,9-Tetrahydro-1*H*-carbazole (4a)

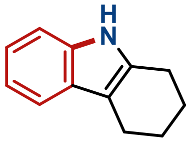

An oven-dried vial equipped with a stir bar was charged with chlorobenzene (45 mg, 0.4 mmol, 1.0 equiv), Rb_2CO_3 (277 mg, 1.2 mmol, 3 equiv), [(ImPyTrippIPr*)Pd(cin)Cl)] (20 mg, 5 mol%), placed under a positive pressure of argon, and subjected to three evacuation/backfilling cycles under high vacuum. Dioxane (2 mL, 0.2 M) and hydrazine hydrate (50% solution in water, 202 mg, 2 mmol, 5 equiv) were added with vigorous stirring, the reaction mixture was placed in a preheated 120 °C oil bath and stirred for 16 h. After 16 h, the reaction mixture was cooled down to room temperature, filtered, washed with DCM and concentrated. Ethanol (2 mL), cyclohexanone (235 mg, 2.4 mmol, 6 equiv), and TFA (228 mg, 2 mmol, 5 equiv) were added and the mixture was further stirred for 12 h at 100 °C. Afterward, the reaction mixture was cooled down to room temperature, diluted with CH_2Cl_2 (10 mL), filtered, and concentrated. Purification by chromatography on silica gel afforded the title product in 57% yield (39.1 mg). White solid. ^1H NMR (600 MHz, $CDCl_3$) δ 7.65 (s, 1H), 7.47 (d, J = 7.7 Hz, 1H), 7.31 – 7.26 (m, 1H), 7.17 – 7.02 (m, 2H), 2.80 – 2.67 (m, 4H), 1.98 – 1.84 (m, 4H). ^{13}C NMR (151 MHz, $CDCl_3$) δ 135.74, 134.20, 127.92, 121.08, 119.20, 117.84, 110.47, 110.26, 23.41, 23.34, 21.03. NMR spectroscopic data agreed with literature values.[10]

5-Methyl-2-phenyl-2,4-dihydro-3*H*-pyrazol-3-one (4b)

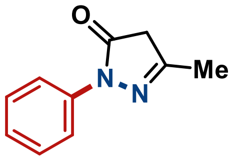

An oven-dried vial equipped with a stir bar was charged with chlorobenzene (45 mg, 0.4 mmol, 1.0 equiv), Rb_2CO_3 (277 mg, 1.2 mmol, 3 equiv), [(ImPyTrippIPr*)Pd(cin)Cl)] (20 mg, 5 mol%), placed under a positive pressure of argon, and subjected to three evacuation/backfilling cycles under high vacuum. Dioxane (2 mL, 0.2 M) and hydrazine hydrate (50% solution in water, 202 mg, 2 mmol, 5 equiv) were added with vigorous stirring, the reaction mixture was placed in a preheated 120 °C oil

bath and stirred for 16 h. After 16 h, the reaction mixture was cooled down to room temperature, filtered, washed with DCM and concentrated. Acetic acid (2 mL) and ethyl acetoacetate (416 mg, 3.2 mmol, 8 equiv) were added and the mixture was further stirred for 12 h at 110 °C. Afterward, the reaction mixture was cooled down to room temperature, diluted with CH_2Cl_2 (10 mL), washed with brine, dried with Na_2SO_4, filtered, and concentrated. Purification by chromatography on silica gel afforded the title product in 46% yield (32 mg). Pale yellow solid. ^{1}H NMR (600 MHz, $CDCl_3$) δ 7.85 (d, *J* = 8.1 Hz, 2H), 7.38 (t, *J* = 7.9 Hz, 2H), 7.17 (t, *J* = 7.4 Hz, 1H), 3.40 (s, 2H), 2.17 (s, 3H). ^{13}C NMR (151 MHz, $CDCl_3$) δ 170.67, 156.42, 138.12, 128.91, 125.12, 118.95, 43.18, 17.09. NMR spectroscopic data agreed with literature values.[11]

1-Phenyl-1*H*-1,2,4-triazole

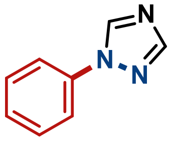

An oven-dried vial equipped with a stir bar was charged with chlorobenzene (45 mg, 0.4 mmol, 1.0 equiv), Rb_2CO_3 (277 mg, 1.2 mmol, 3 equiv), [(ImPyTrippIPr*)Pd(cin)Cl)] (20 mg, 5 mol%), placed under a positive pressure of argon, and subjected to three evacuation/backfilling cycles under high vacuum. Dioxane (2 mL, 0.2 M) and hydrazine hydrate (50% solution in water, 202 mg, 2 mmol, 5 equiv) were added with vigorous stirring, the reaction mixture was placed in a preheated 120 °C oil bath and stirred for 16 h. After 16 h, the reaction mixture was cooled down to room temperature, filtered, washed with DCM and concentrated. Formamide (2 mL) was added and the mixture was further stirred for 12 h at 120 °C. Afterward, the reaction mixture was cooled down to room temperature, diluted with CH_2Cl_2 (10 mL), washed with brine, dried with Na_2SO_4, filtered, and concentrated. Purification by chromatography on silica gel afforded the title product in 60% yield (34.7 mg). Yellow oil. ^{1}H NMR (500 MHz, $CDCl_3$) δ 8.56 (s, 1H), 8.10 (s, 1H), 7.68 (d, *J* = 7.4 Hz, 2H), 7.51 (t, *J* = 8.0 Hz, 2H), 7.40 (t, *J* = 7.5 Hz, 1H). ^{13}C NMR (125 MHz, $CDCl_3$) δ 152.72, 140.98, 137.14, 129.92, 128.38, 120.22. NMR spectroscopic data agreed with literature values.[12]

Mechanistic studies

Initial Rate Measurements with NaOtBu at 60 °C.

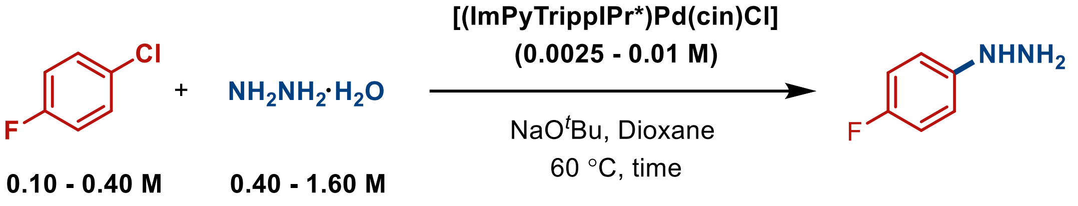

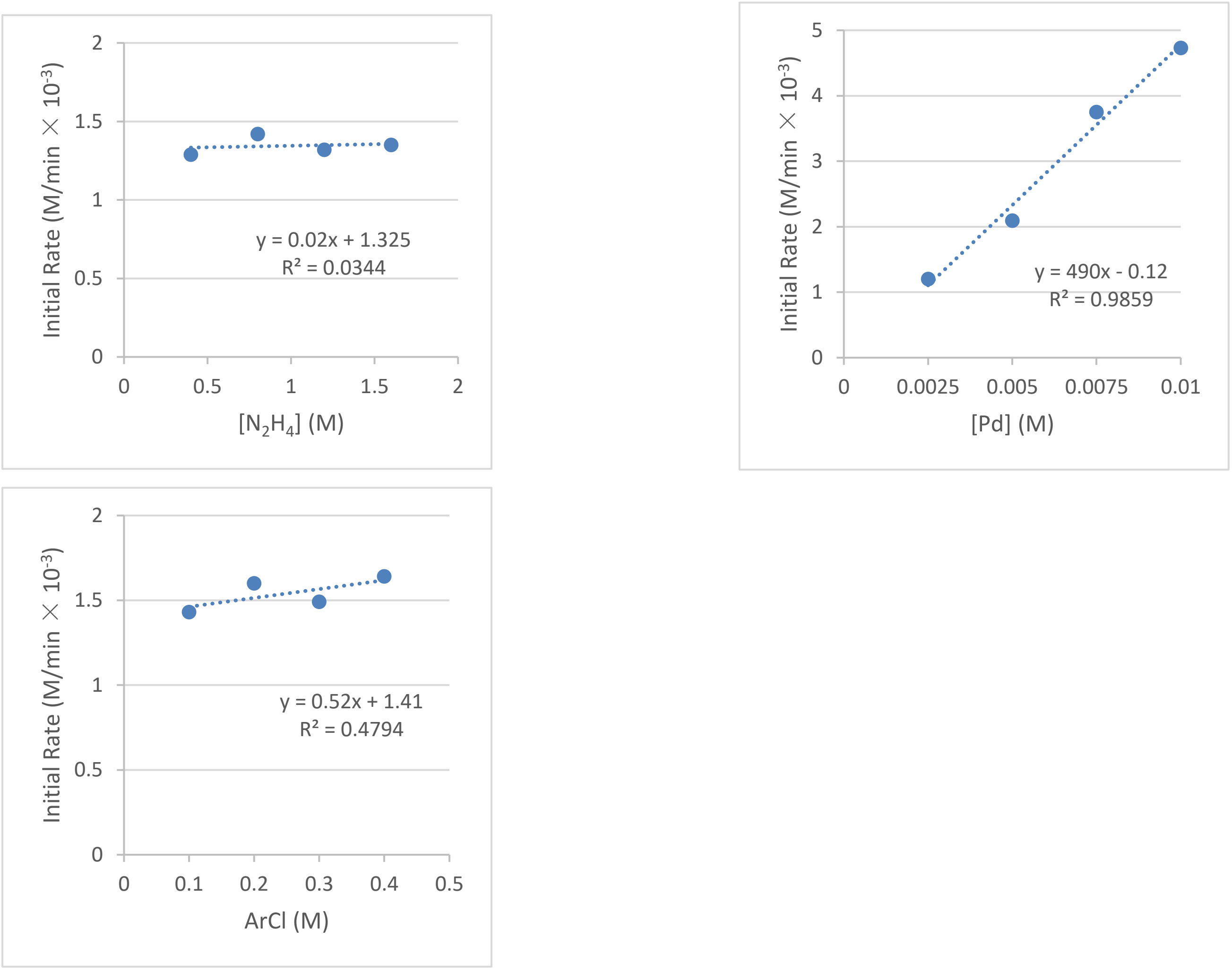

Figure S1: Kinetic studies on the monoarylation of hydrazine with NaOtBu as base. a) Dependence of initial rate on [N_2H_4] (0.40–1.60 M) with [ArCl]=0.20 M and [Pd]=0.006 M. b) Dependence of initial rate on [Pd] (0.0025–0.01 M) with [ArCl]=0.20 M and [N_2H_4] =1.0 M. c) Dependence of initial rate on [ArCl] (0.10–0.40 M) with [N_2H_4]=1.0 M and [Pd] and [Pd]=0.006 M.

order in hydrazine

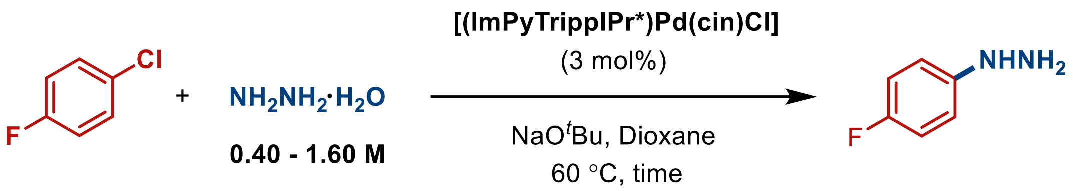

Table S4: Initial rate dependence on hydrazine monohydrate concentration

Entry	$[N_2H_4]$ (M)	Time Point Interval (min)	Concentration of ArCl (M)	Initial Rate (M/min × 10^{-3})
1	0.40	30	0.0388	1.29
2	0.80	30	0.0426	1.42
3	1.20	30	0.0396	1.32
4	1.60	30	0.0404	1.35

An 5 mL oven-dried vial equipped with a stir bar was charged with 4-fluoro-chlorobenzene (0.20 mmol, 26.1 mg), 4-fluorotoluene (0.20 mmol, 22.0 mg), NaO^{t}Bu (0.30 mmol, 1.5 equiv, 28.8 mg) and [(ImPyTrippIPr*)Pd(cin)Cl)] (3 mol%, 6.1 mg), placed under a positive pressure of argon, and subjected to three evacuation/backfilling cycles under high vacuum. An appropriate amount of hydrazine monohydrate was added to the reaction corresponding to the desired amount of hydrazine (19.6-78.4 µL, 0.40-1.60 mmol). Next, the reaction was adjusted by adding an appropriate quantity of 1,4-dioxane to reach a total liquid volume of 1000 µL. This procedure was replicated thrice, resulting in four separate reactions with hydrazine monohydrate concentration of 0.40 mol/L, 0.80 mol/L, 1.20 mol/L, and 1.60 mol/L. Subsequently, these reactions were subjected to heating at 60 °C with continuous stirring for a predefined duration (as indicated below). At each time point, a 15 µL sample was withdrawn from each reaction and placed into an NMR tube, followed by the addition of 600 µL of $CDCl_3$. This sequence was iterated until a total of six time points (including t = 0 minute) were collected. The initial rate was then determined by tracking the degradation of 4-fluoro-chlorobenzene over time via ^{19}F NMR spectroscopy.

$[N_2H_4]$ (M)	Conversion of ArCl (%)				
	10 min	20 min	30 min	40 min	60 min
0.40	11.5	16.0	19.4	24.2	29.1
0.80	9.5	17.6	21.3	22.5	28.9
1.20	12.9	16.8	19.8	25.7	31.4
1.60	11.2	18.8	20.0	25.4	33.2

order in palladium

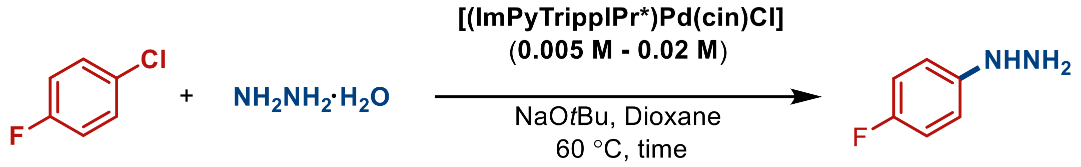

Table S5: Initial rate dependence on palladium catalyst loading

Entry	[Pd] (M)	Time Point Interval (min)	Concentration of ArCl (M)	Initial Rate (M/min × 10^{-3})
1	0.0025	20	0.0240	1.20
2	0.005	20	0.0418	2.09
3	0.0075	15	0.0562	3.75
4	0.01	15	0.0710	4.73

An 5 mL oven-dried vial equipped with a stir bar was charged with 4-fluoro-chlorobenzene (0.20 mmol, 26.1 mg), 4-fluorotoluene (0.20 mmol, 22.0 mg), NaO^{t}Bu (0.30 mmol, 1.5 equiv, 28.8 mg) and the desired amount of [(ImPyTrippIPr*)Pd(cin)Cl)] (1.25-5.0 mol%, 2.6-10.2 mg), placed under a positive pressure of argon, and subjected to three evacuation/backfilling cycles under high vacuum. Next, the reaction was adjusted by adding 49.0 µL of hydrazine (5 equiv, 1.0 mmol) and an appropriate quantity of 1,4-dioxane to reach a total liquid volume of 1000 µL. This procedure was replicated thrice, resulting in four separate reactions with the loading of precatalyst [(ImPyTrippIPr*)Pd(cin)Cl)] 0.0025 mol/L, 0.005 mol/L, 0.0075 mol/L, and 0.010 mol/L. Subsequently, these reactions were subjected to heating at 60 °C with continuous stirring for a predefined duration (as indicated below). At each time point, a 15 µL sample was withdrawn from each reaction and placed into an NMR tube, followed by the addition of 600 µL of $CDCl_3$. This sequence was iterated until a total of six time points (including t = 0 minute) were collected. The initial rate was then determined by tracking the degradation of 4-fluoro-chlorobenzene over time via ^{19}F NMR spectroscopy.

[Pd] (M)	Conversion of ArCl (%)				
	10 min	15 min	20min	25 min	35min
0.0025	9.5	10.6	12.0	14.8	16.3
0.005	17.4	19.4	20.9	22.5	24.8
0.0075	22.0	28.1	35.3	36.6	43.8
0.01	27.5	35.5	37.5	40.7	55.9

order in chloroarene

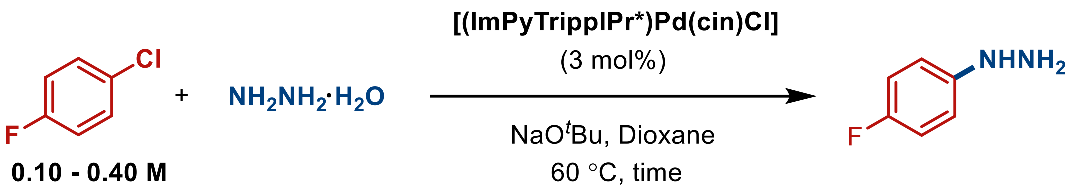

Table S6: Initial rate dependence on aryl chloride concentration

Entry	[Ar-Cl] (M)	Time Point Interval (min)	Concentration of ArCl (M)	Initial Rate (M/min × 10^{-3})
1	0.10	20	0.0286	1.43
2	0.20	20	0.0320	1.60
3	0.30	20	0.0297	1.49
4	0.40	20	0.0328	1.64

An 5 mL oven-dried vial equipped with a stir bar was charged with 4-fluoro-chlorobenzene (0.10-0.40 mmol, 13.1-52.2 mg), 4-fluorotoluene (0.20 mmol, 22.0 mg), NaO^{t}Bu (0.30 mmol, 1.5 equiv, 28.8 mg) and the desired amount of [(ImPyTrippIPr*)Pd(cin)Cl)] (3 mol%, 6.1 mg), placed under a positive pressure of argon, and subjected to three evacuation/backfilling cycles under high vacuum. Next, the reaction was adjusted by adding 49.0 µL of hydrazine (5 equiv, 1.0 mmol) and an appropriate quantity of 1,4-dioxane to reach a total liquid volume of 1000 µL. This procedure was replicated thrice, resulting in four separate reactions with the concentration of 4-fluoro-chlorobenzene 0.10 mol/L, 0.20 mol/L, 0.30 mol/L, and 0.40 mol/L. Subsequently, these reactions were subjected to heating at 60 °C with continuous stirring for a predefined duration (as indicated below). At each time point, a 15 µL sample was withdrawn from each reaction and placed into an NMR tube, followed by the addition of 600 µL of $CDCl_3$. This sequence was iterated until a total of six time points (including t = 0 minute) were collected. The initial rate was then determined by tracking the degradation of 4-fluoro-chlorobenzene over time via ^{19}F NMR spectroscopy.

[Ar-Cl] (M)	Conversion of ArCl (%)				
	10 min	20 min	30min	40 min	60 min
0.10	20.8	28.6	42.8	42.5	50.5
0.20	11.5	16	19.4	24.2	29.1
0.30	7.8	9.9	14.5	20.8	22.4
0.40	6	8.2	12.1	17.5	20.8

Initial Rate Measurements with Rb_2CO_3 at 120 °C.

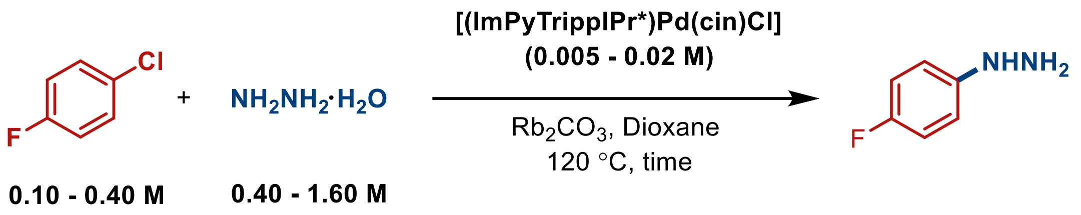

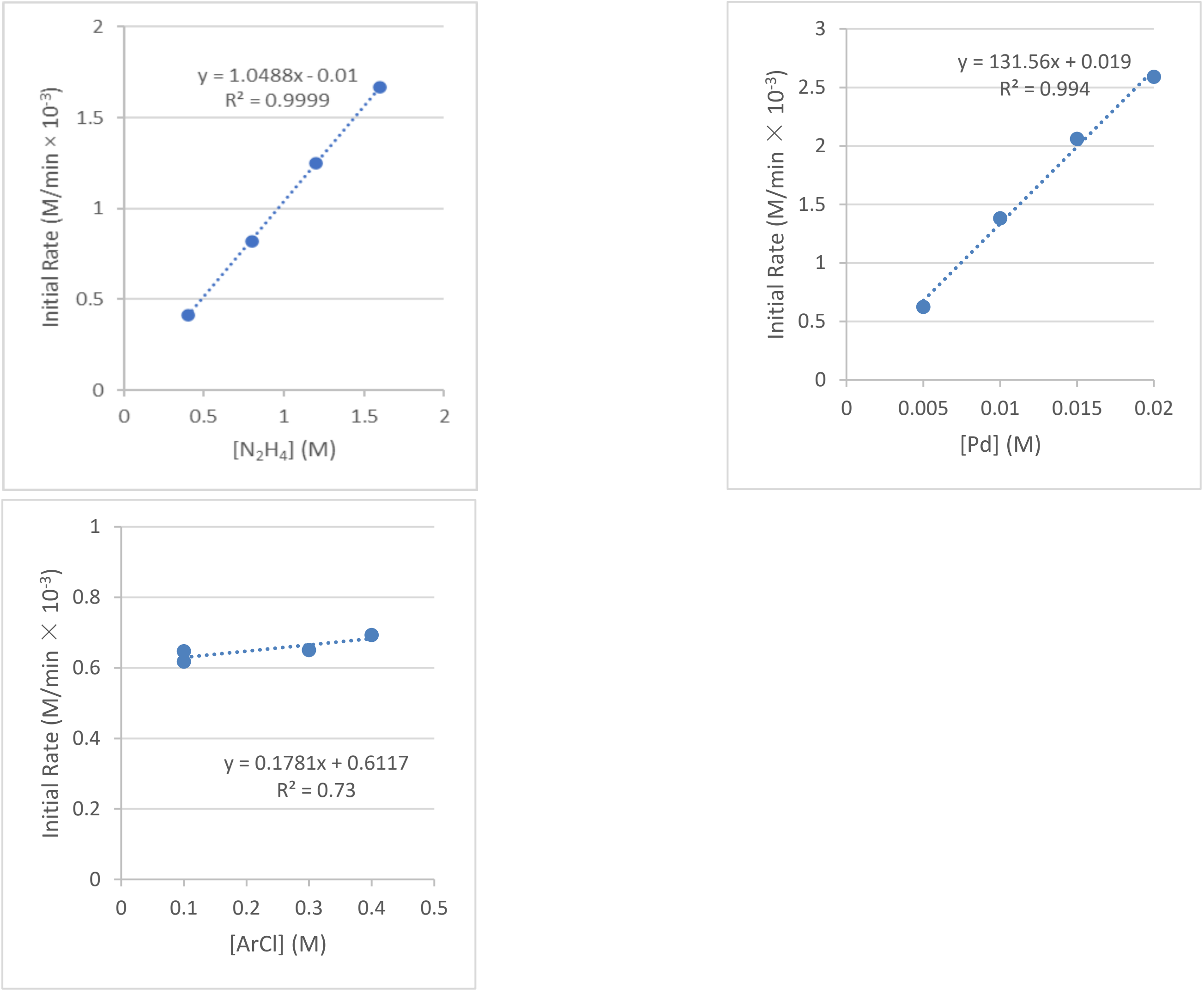

Figure S2: Kinetic studies on the monoarylation of hydrazine with Rb_2CO_3 as base. a) Dependence of initial rate on $[N_2H_4]$ (0.40–1.60 M) with [ArCl]=0.20 M and [Pd]=0.006 M. b) Dependence of initial rate on [Pd] (0.005–0.02 M) with [ArCl]=0.20 M and $[N_2H_4]$ =1.0 M. c) Dependence of initial rate on [ArCl] (0.10–0.40 M) with $[N_2H_4]$ =1.0 M and [Pd] and [Pd]=0.005 M.

order in hydrazine

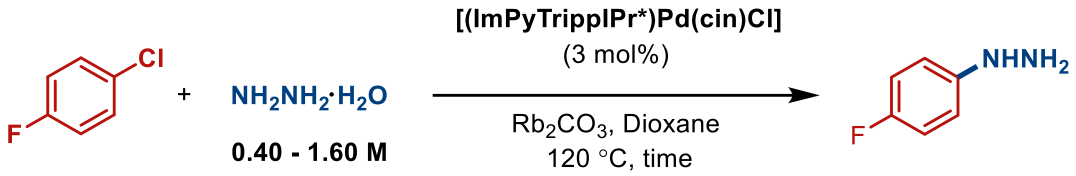

Table S7: Initial rate dependence on hydrazine monohydrate concentration

Entry	$[N_2H_4]$ (M)	Time Point Interval (min)	Concentration of ArCl (M)	Initial Rate (M/min × 10^{-3})
1	0.40	40	0.0166	0.415
2	0.80	40	0.0328	0.820
3	1.20	30	0.0376	1.25
4	1.60	30	0.0502	1.67

An 5 mL oven-dried vial equipped with a stir bar was charged with 4-fluoro-chlorobenzene (0.20 mmol, 26.1 mg), 4-fluorotoluene (0.20 mmol, 22.0 mg), Rb_2CO_3 (0.30 mmol, 1.5 equiv, 69.3 mg) and [(ImPyTrippIPr*)Pd(cin)Cl)] (3 mol%, 6.1 mg), placed under a positive pressure of argon, and subjected to three evacuation/backfilling cycles under high vacuum. An appropriate amount of hydrazine monohydrate was added to the reaction corresponding to the desired amount of hydrazine (19.6-78.4 µL, 0.40-1.60 mmol). Next, the reaction was adjusted by adding an appropriate quantity of 1,4-dioxane to reach a total liquid volume of 1000 µL. This procedure was replicated thrice, resulting in four separate reactions with hydrazine monohydrate concentration of 0.40 mol/L, 0.80 mol/L, 1.20 mol/L, and 1.60 mol/L. Subsequently, these reactions were subjected to heating at 120 °C with continuous stirring for a predefined duration (as indicated in below). At each time point, a 15 µL sample was withdrawn from each reaction and placed into an NMR tube, followed by the addition of 600 µL of $CDCl_3$. This sequence was iterated until a total of six time points (including t = 0 minute) were collected. The initial rate was then determined by tracking the degradation of 4-fluoro-chlorobenzene over time via ^{19}F NMR spectroscopy.

$[N_2H_4]$ (M)	Conversion of ArCl (%)				
	10 min	20 min	30 min	40 min	60 min
0.40	5.2	6.2	6.6	8.3	12.4
0.80	5.2	9.1	12.4	16.4	33.5
1.20	5.1	10.8	18.8	24.2	41.8
1.60	7.6	11.3	25.0	31.7	49.4

order in palladium

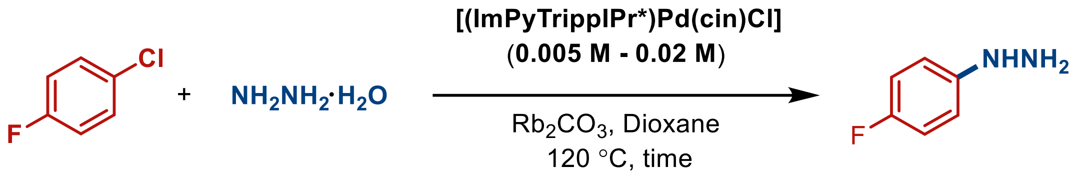

Table S8: Initial rate dependence on palladium catalyst loading

Entry	[Pd] (M)	Time Point Interval (min)	Concentration of ArCl (M)	Initial Rate (M/min × 10^{-3})
1	0.005	25	0.0156	0.624
2	0.01	25	0.0344	1.38
3	0.015	30	0.0412	2.06
4	0.02	30	0.0518	2.59

An 5 mL oven-dried vial equipped with a stir bar was charged with 4-fluoro-chlorobenzene (0.20 mmol, 26.1 mg), 4-fluorotoluene (0.20 mmol, 22.0 mg), Rb_2CO_3 (0.30 mmol, 1.5 equiv, 69.3 mg) and the desired amount of [(ImPyTrippIPr*)Pd(cin)Cl)] (2.5-10.0 mol%, 5.1-20.4 mg), placed under a positive pressure of argon, and subjected to three evacuation/backfilling cycles under high vacuum. Next, the reaction was adjusted by adding 49.0 µL of hydrazine (5 equiv, 1.0 mmol) and an appropriate quantity of 1,4-dioxane to reach a total liquid volume of 1000 µL. This procedure was replicated thrice, resulting in four separate reactions with the loading of precatalyst [(ImPyTrippIPr*)Pd(cin)Cl)] 0.005 mol/L, 0.010 mol/L, 0.015 mol/L, and 0.020 mol/L. Subsequently, these reactions were subjected to heating at 120 °C with continuous stirring for a predefined duration (as indicated below). At each time point, a 15 µL sample was withdrawn from each reaction and placed into an NMR tube, followed by the addition of 600 µL of $CDCl_3$. This sequence was iterated until a total of six time points (including t = 0 minute) were collected. The initial rate was then determined by tracking the degradation of 4-fluoro-chlorobenzene over time via ^{19}F NMR spectroscopy.

[Pd] (M)	Conversion of ArCl (%)				
	15 min	20 min	25min	30 min	40 min
0.005	5.2	6.5	7.8	9.7	15.2
0.01	9.9	16.0	17.2	18.9	26.5
0.015	14.5	20.6	23.1	26.0	35.5
0.02	22.0	25.9	29.5	38.5	54.8

order in chloroarene

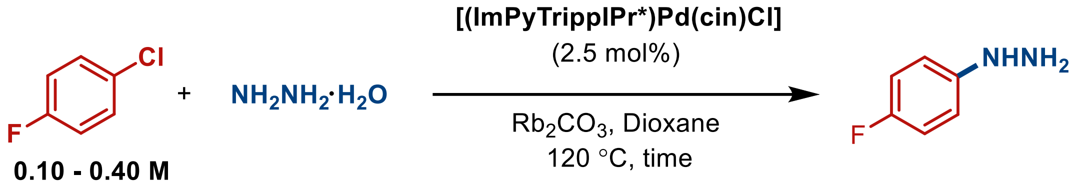

Table S9: Initial rate dependence on aryl chloride concentration

Entry	[Ar-Cl] (M)	Time Point Interval (min)	Concentration of ArCl (M)	Initial Rate (M/min × 10^{-3})
1	0.10	30	0.0185	0.617
2	0.20	30	0.0194	0.647
3	0.30	30	0.0195	0.650
4	0.40	30	0.0208	0.693

An 5 mL oven-dried vial equipped with a stir bar was charged with 4-fluoro-chlorobenzene (0.10-0.40 mmol, 13.1-52.2 mg), 4-fluorotoluene (0.20 mmol, 22.0 mg), Rb_2CO_3 (0.30 mmol, 1.5 equiv, 69.3 mg) and the desired amount of [(ImPyTrippIPr*)Pd(cin)Cl)] (2.5 mol%, 5.1 mg), placed under a positive pressure of argon, and subjected to three evacuation/backfilling cycles under high vacuum. Next, the reaction was adjusted by adding 49.0 µL of hydrazine (5 equiv, 1.0 mmol) and an appropriate quantity of 1,4-dioxane to reach a total liquid volume of 1000 µL. This procedure was replicated thrice, resulting in four separate reactions with the concentration of 4-fluoro-chlorobenzene 0.10 mol/L, 0.20 mol/L, 0.30 mol/L, and 0.40 mol/L. Subsequently, these reactions were subjected to heating at 120 °C with continuous stirring for a predefined duration (as indicated below). At each time point, a 15 µL sample was withdrawn from each reaction and placed into an NMR tube, followed by the addition of 600 µL of $CDCl_3$. This sequence was iterated until a total of six time points (including t = 0 minute) were collected. The initial rate was then determined by tracking the degradation of 4-fluoro-chlorobenzene over time via ^{19}F NMR spectroscopy.

[Ar-Cl] (M)	Conversion of ArCl (%)				
	15 min	20 min	25min	30 min	40 min
0.10	8.2	18.0	17.6	18.5	19.2
0.20	5.2	6.5	7.8	9.7	15.2
0.30	4.3	6.0	6.1	6.5	8.8
0.40	2.9	3.8	4.5	5.2	6.9

<u>*Kinetic Isotope Effect Studies.*</u>

<u>NaOtBu as base at 60 °C</u>

Two 5 mL oven-dried vial each equipped with a stir bar was charged with 4-fluoro-chlorobenzene (0.20 mmol, 26.1 mg), 4-fluorotoluene (0.20 mmol, 22.0 mg), NaOtBu (0.30 mmol, 1.5 equiv, 28.8 mg) and [(ImPyTrippIPr*)Pd(cin)Cl)] (3 mol%, 6.1 mg), placed under a positive pressure of argon, and subjected to three evacuation/backfilling cycles under high vacuum. Subsequently, a vial was charged with hydrazine (5 equivalents, 1.0 mmol, 49.0 µL), while another with Hydrazine-d4 monodeuterate (5 equivalents, 1.0 mmol, 48.5 µL), and each followed by an appropriate quantity of 1,4-dioxane to reach a total liquid volume of 1000 µL. These reactions were subjected to heating at 120 °C with continuous stirring for a predefined duration (as indicated above). At each time point, a 15 µL sample was withdrawn from each reaction and placed into an NMR tube, followed by the addition of 600 µL of CDCl3. This sequence was iterated until a total of six time points (including t = 0 minute) were collected. The yield was then determined by tracking the amount of phenyl hydrazine product over time via ^{19}F NMR spectroscopy.

Time (min)	Ar-NHNH$_2$		Ar-NDND$_2$	
	Yield (%)	Concentration (M)	Yield (%)	Concentration (M)
0	0	0	0	0
30	8.2	0.0164	4.8	0.0096
40	11.5	0.0230	8.1	0.0162
50	17.4	0.0348	12.7	0.0254
60	21.3	0.0426	14.3	0.0286
80	25.8	0.0516	19.1	0.0382

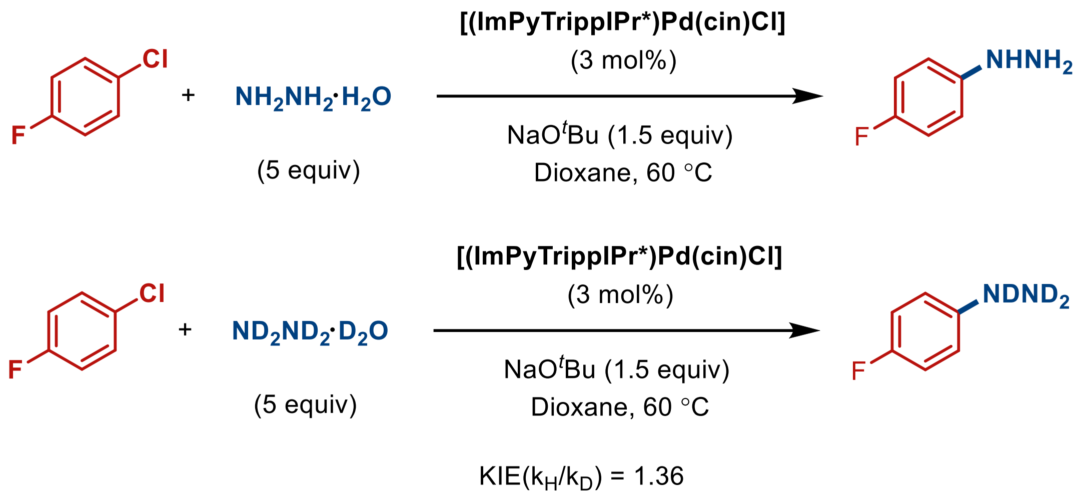

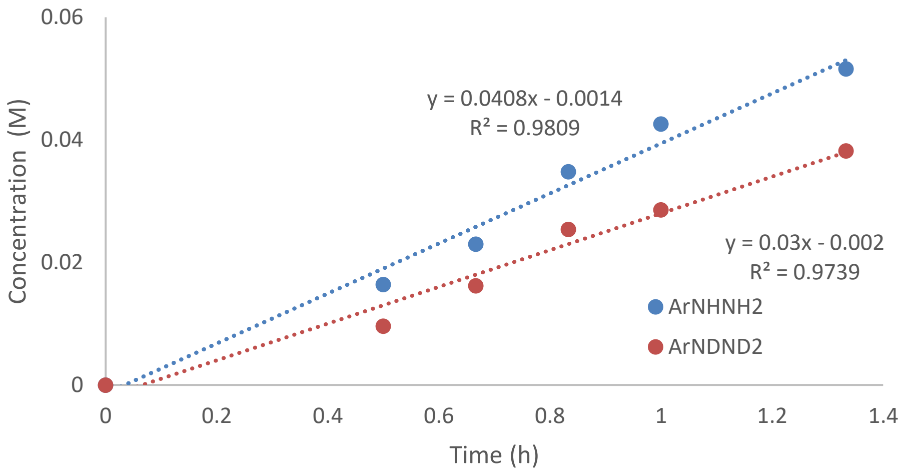

Figure S3: KIE of arylhydrazine with NaO*t*Bu as base.

Rb$_2$CO$_3$ as base at 120 °C

Two 5 mL oven-dried vial each equipped with a stir bar was charged with 4-fluoro-chlorobenzene (0.20 mmol, 26.1 mg), 4-fluorotoluene (0.20 mmol, 22.0 mg), Rb$_2$CO$_3$ (0.30 mmol, 1.5 equiv, 69.3 mg) and [(ImPyTrippIPr*)Pd(cin)Cl)] (3 mol%, 6.1 mg), placed under a positive pressure of argon, and subjected to three evacuation/backfilling cycles under high vacuum. Subsequently, a vial was charged with hydrazine (5 equivalents, 1.0 mmol, 49.0 µL), while another with Hydrazine-d4 monodeuterate (5 equivalents, 1.0 mmol, 48.5 µL), and each followed by an appropriate quantity of 1,4-dioxane to reach a total liquid volume of 1000 µL. These reactions were subjected to heating at 120 °C with continuous stirring for a predefined duration (as indicated above). At each time point, a 15 µL sample was withdrawn from each reaction and placed into an NMR tube, followed by the addition of 600 µL of CDCl$_3$. This sequence was iterated until a total of six time points (including t = 0 minute) were collected. The yield was then determined by tracking the amount of phenyl hydrazine product over time via ^{19}F NMR spectroscopy.

Time (min)	Ar-NHNH$_2$		Ar-NDND$_2$	
	Yield (%)	Concentration (M)	Yield (%)	Concentration (M)
0	0	0	0	0
30	7.4	0.0148	4.6	0.0092
40	11.6	0.0232	9.4	0.0188
60	19.4	0.0388	17.3	0.0346
80	23.9	0.0478	22.8	0.0456
110	35.6	0.0712	33.3	0.0666

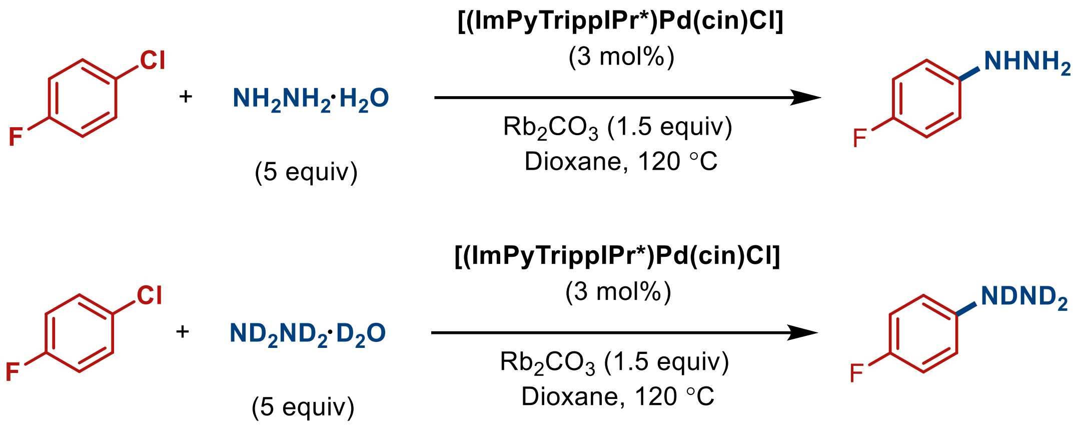

KIE(k_H/k_D) = 1.032

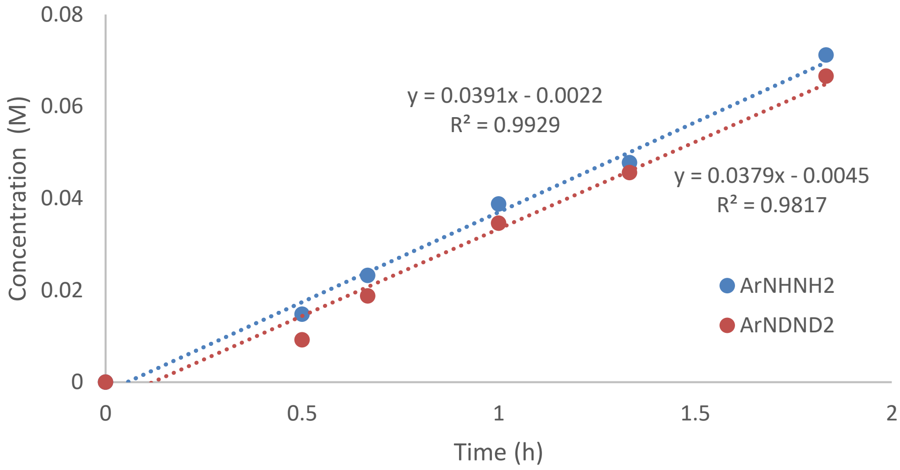

Figure S4: KIE of arylhydrazine with Rb_2CO_3 as base.

<u>*Time course of the reaction with NaOtBu at 60 °C.*</u>

An 5 mL oven-dried vial equipped with a stir bar was charged with 4-fluoro-chlorobenzene (0.20 mmol, 26.1 mg), 4-fluorotoluene (0.20 mmol, 22.0 mg), NaO^{t}Bu (0.30 mmol, 1.5 equiv, 28.8 mg) and [(ImPyTrippIPr*)Pd(cin)Cl)] (3 mol%, 6.1 mg), placed under a positive pressure of argon, and subjected to three evacuation/backfilling cycles under high vacuum. An appropriate amount of hydrazine monohydrate was added to the reaction corresponding to the desired amount of hydrazine (58.5 µL, 1.20 mmol). Next, the reaction was adjusted by adding an appropriate quantity of 1,4-dioxane to reach a total liquid volume of 1000 µL. Subsequently, the reaction was subjected to heating at 60 °C with continuous stirring for a predefined duration (as indicated below). At each time point, a 15 µL sample was withdrawn from each reaction and placed into an NMR tube, followed by the addition of 600 µL of $CDCl_3$. This sequence was iterated until a total of six time points (including t = 0 minute) were collected. The initial rate was then determined by tracking the degradation of 4-fluoro-chlorobenzene over time via ^{19}F NMR spectroscopy.

			t		
	10 min	20 min	30 min	40 min	60 min
[ArCl] (mM)	174.2	166.4	160.4	148.6	137.2

Time course of the reaction with Rb_2CO_3 at 120 °C.

An 5 mL oven-dried vial equipped with a stir bar was charged with 4-fluoro-chlorobenzene (0.20 mmol, 26.1 mg), 4-fluorotoluene (0.20 mmol, 22.0 mg), Rb2CO3 (0.30 mmol, 1.5 equiv, 69.3 mg) and [(ImPyTrippIPr*)Pd(cin)Cl)] (3 mol%, 6.1 mg), placed under a positive pressure of argon, and subjected to three evacuation/backfilling cycles under high vacuum. An appropriate amount of hydrazine monohydrate was added to the reaction corresponding to the desired amount of hydrazine (58.5 µL, 1.20 mmol). Next, the reaction was adjusted by adding an appropriate quantity of 1,4-dioxane to reach a total liquid volume of 1000 µL. Subsequently, these reactions were subjected to heating at 120 °C with continuous stirring for a predefined duration (as indicated in below). At each time point, a 15 µL sample was withdrawn from each reaction and placed into an NMR tube, followed by the addition of 600 µL of CDCl3. This sequence was iterated until a total of six time points (including t = 0 minute) were collected. The initial rate was then determined by tracking the degradation of 4-fluoro-chlorobenzene over time via ^{19}F NMR spectroscopy.

			t		
	10 min	20 min	30 min	40 min	60 min
[ArCl] (mM)	189.8	178.4	162.4	151.6	116.4

Kinetic comparison of aryl chloride and aryl bromide with NaOtBu at 60 °C.

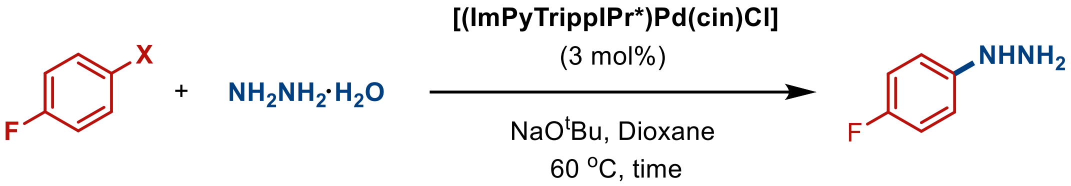

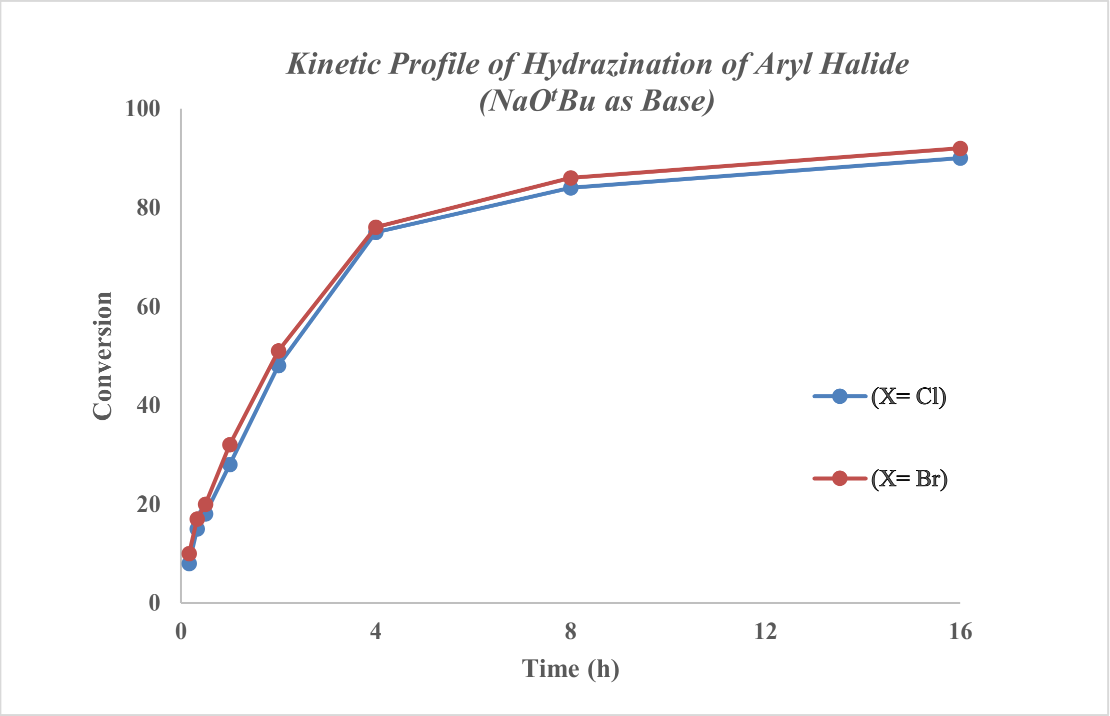

Figure S5: Kinetic comparison of aryl chloride and aryl bromide with NaOtBu at 60 °C. General conditions: phenyl halide (0.2 mmol, 1.0 equiv), $NH_2NH_2 \cdot H_2O$ (5 equiv), [(ImPyTrippIPr*)Pd(cin)Cl] (3 mol%), NaO^{t}Bu (1.5 equiv), dioxane (0.20 M), 60 °C, time (h). Conversions determined from ^{19}F NMR with 4-Fluorotoluene as internal standard.

Kinetic comparison of aryl chloride and aryl bromide with Rb_2CO_3 at 120 °C.

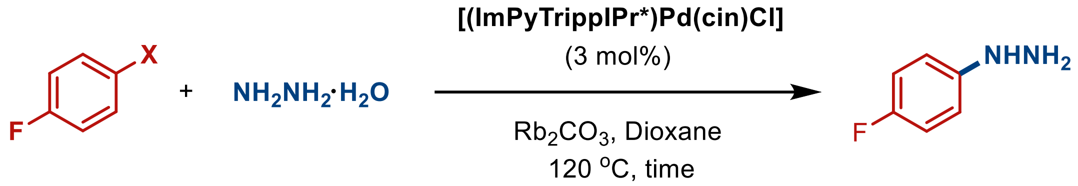

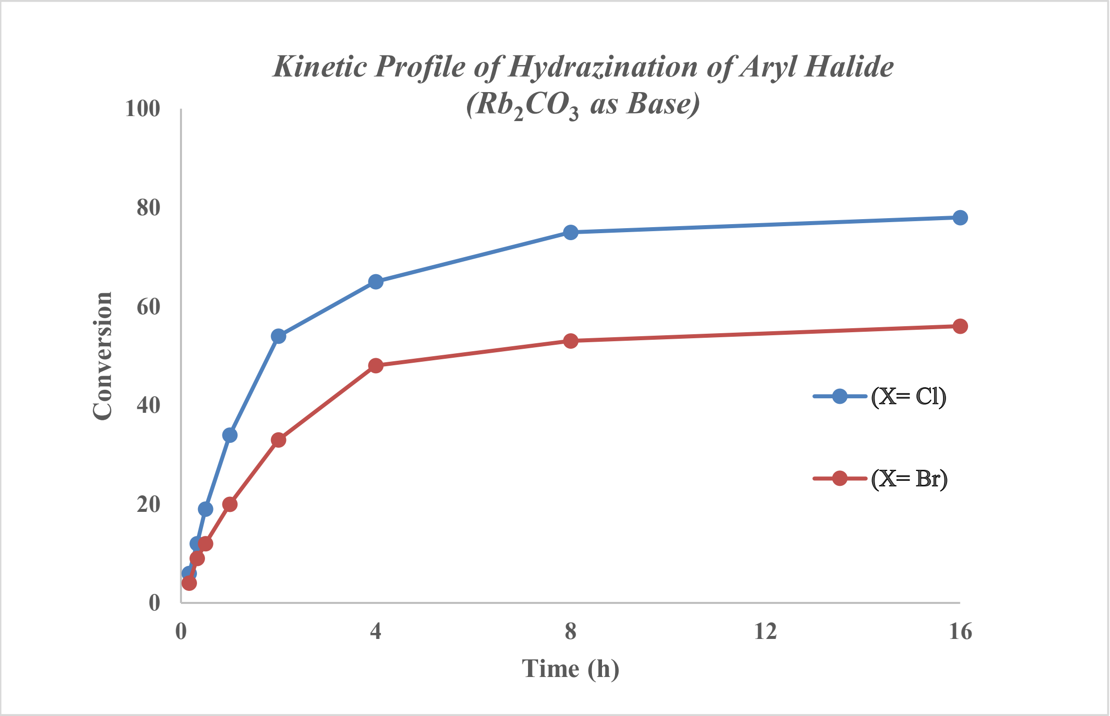

Figure S6: Kinetic comparison of aryl chloride and aryl bromide with Rb_2CO_3 at 120 °C. General conditions: phenyl halide (0.2 mmol, 1.0 equiv), $NH_2NH_2 \cdot H_2O$ (5 equiv), [(ImPyTrippIPr*)Pd(cin)Cl] (3 mol%), Rb_2CO_3 (3 equiv), dioxane (0.20 M), 120 °C, time (h). Conversions determined from ^{19}F NMR with 4-Fluorotoluene as internal standard.

Details of Crystal Structure Analysis of [(TrippIPr*)Pd(cin)Cl] and [(TrippIPr*)CuCl]

Table S10. *Crystal Data and Structure Refinement Summaries for **[(TrippIPr*)Pd(cin)Cl]** and **[(TrippIPr*)CuCl]**.*

Compound	**[(TrippIPr*)Pd(cin)Cl]**	**[(TrippIPr*)CuCl]**
Chemical formula	$C_{64}H_{63}ClN_2Pd \cdot CH_2Cl_2$	$C_{55}H_{54}ClCuN_2$
M_r	1086.94	841.99
Crystal system, space group	Monoclinic, C2/c	Monoclinic, $P2_1/n$
Temperature (K)	100	293
a, *b*, *c* (Å)	32.1683 (4), 13.6846 (2), 25.2892 (3)	14.5586 (5), 24.1614 (9), 15.8762 (6)
β (°)	96.539 (1)	101.365 (3)
V ($Å^3$)	11060.1 (3)	5475.0 (4)
Z	8	4
Radiation type	Cu Kα	Mo *K*α
μ (mm^{-1})	4.35	0.48
Crystal size (mm)	0.22 × 0.18 × 0.12	0.4 × 0.25 × 0.1
Diffractometer	XtaLAB Synergy R, DW system, HyPix-Arc 150	KM4 with Eos CCD
Absorption correction	Multi-scan SCALE3 ABSPACK (Rigaku Oxford Diffraction, 2015).	Multi-scan SCALE3 ABSPACK (Rigaku Oxford Diffraction, 2015).
T_{min}, T_{max}	0.449, 0.593	0.980, 1.000
No. of measured, independent and observed [$I > 2\sigma(I)$] reflections	33220, 9927, 8884	29344, 10717, 4793
R_{int}	0.023	0.083
$(\sin \theta/\lambda)_{max}$ ($Å^{-1}$)	0.599	0.617
$R[F^2 > 2\sigma(F^2)]$, $wR(F^2)$, S	0.060, 0.174, 0.85	0.102, 0.343, 1.06
No. of reflections	9927	10714
No. of parameters	691	539
No. of restraints	369	389
H-atom treatment	H-atom parameters constrained	H-atom parameters constrained
$\Delta\rho_{max}$, $\Delta\rho_{min}$ (e $Å^{-3}$)	2.14, −1.37	1.05, −0.46

Computer programs: CrysAlis PRO 1.171.41.61a (Rigaku OD, 2020), SHELXS(Sheldrick, 2018), SHELXL2018/1 (Sheldrick, 2018).

ORTEP Structure of [(TrippIPr*)Pd(cin)Cl] – Front View

Figure S7. *ORTEP Structure of* ***[(TrippIPr*)Pd(cin)Cl]*** *(30% ellipsoids).* (Crystallographic data has been deposited with the Cambridge Crystallographic Data Center as supplementary publication no. CCDC 2271723).

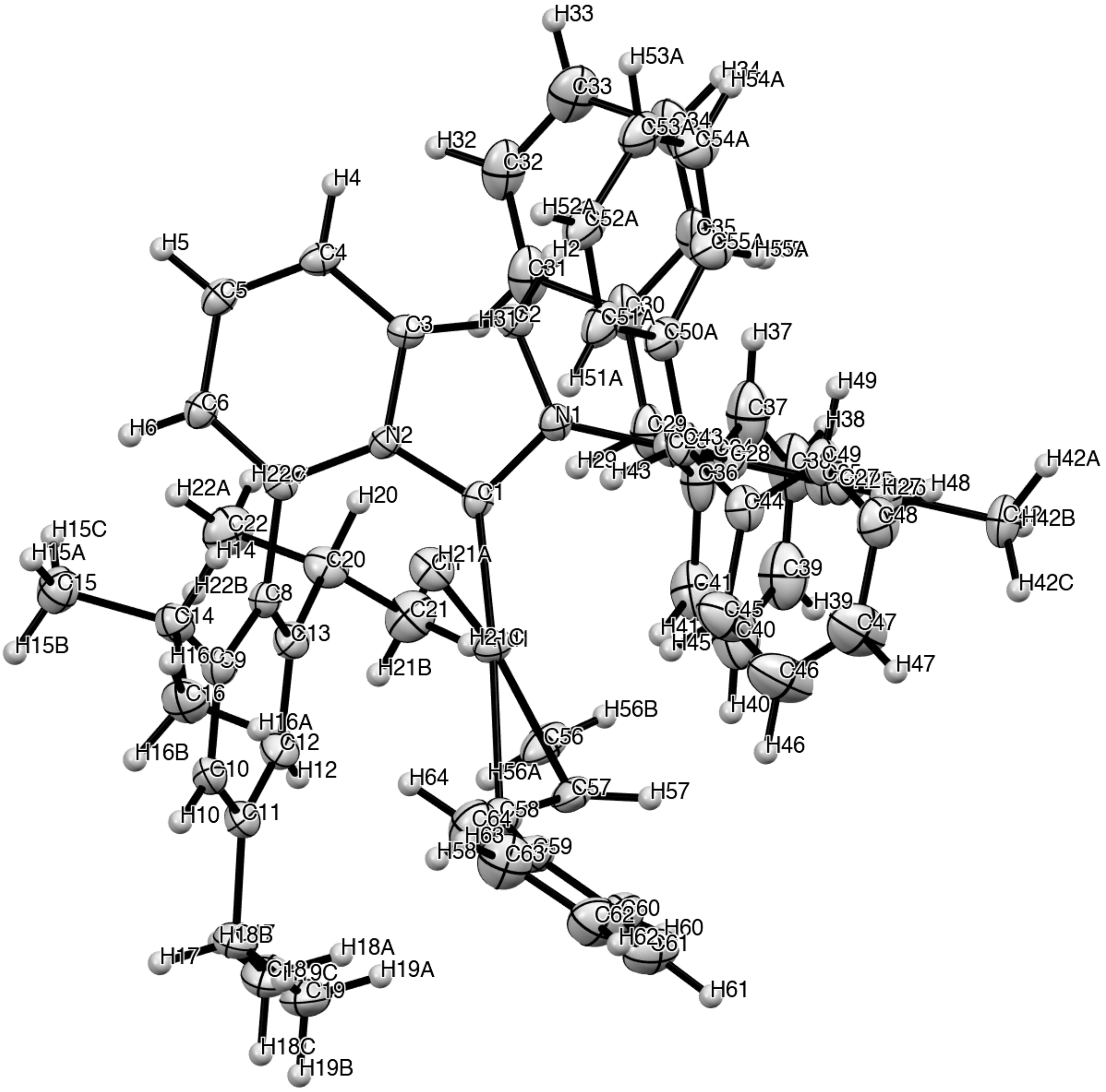

Selected bond lengths [Å] and angles [°]: Pd–C1, 2.024(4); Pd–Cl, 2.378(1); Pd–C56, 2.091(5); Pd–C57, 2.134(4); Pd–C58, 2.241(5); C1–N1, 1.374(5); C1–N2, 1.371(5); C7–N2, 1.413(5); C23–N1, 1.457(5); C7–C8, 1.498(6); C1–Pd–C56, 104.2(2); C1–Pd–C57, 139.4(2); C1–Pd–C58, 171.9(2); C56–Pd–C58, 69.6(2); C1–Pd–Cl, 87.7(1); N1–C1–N2, 103.2(3); C7–N2–C1, 127.6(3); C23–N1–C1, 122.5(3); C8–C7–N2, 119.1(3).

ORTEP Structure of [(TrippIPr*)Pd(cin)Cl] – Side View

Figure S8. *ORTEP Structure of* ***[(TrippIPr*)Pd(cin)Cl]*** *(30% ellipsoids).* (Crystallographic data has been deposited with the Cambridge Crystallographic Data Center as supplementary publication no. CCDC 2271723).

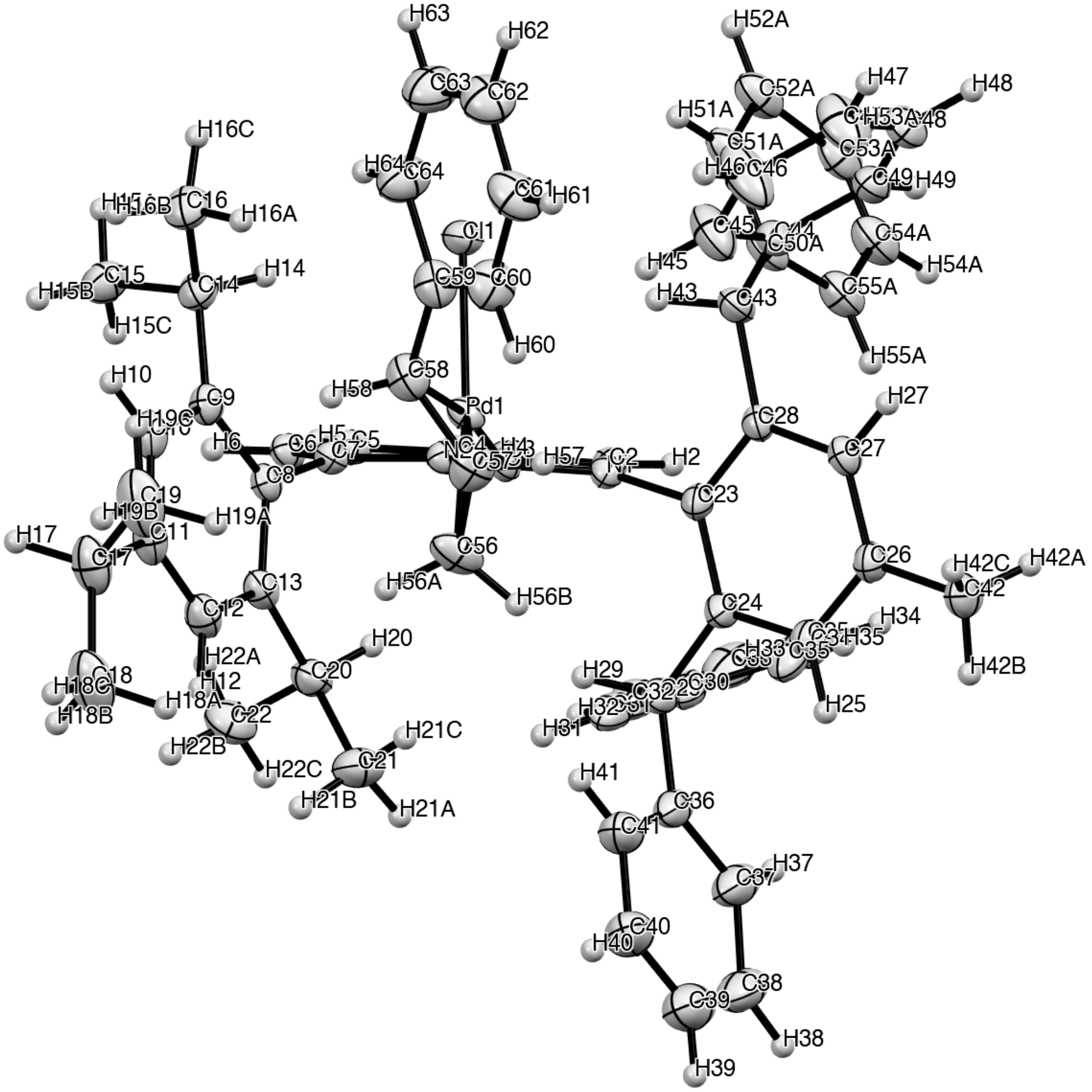

See, Figure S7 for selected bond lengths and angles.

ORTEP Structure of [(TrippIPr*)CuCl] – Side View

Figure S9. *ORTEP Structure of* ***[(TrippIPr*)CuCl]*** *(30% ellipsoids).* (Crystallographic data has been deposited with the Cambridge Crystallographic Data Center as supplementary publication no. CCDC 2271724).

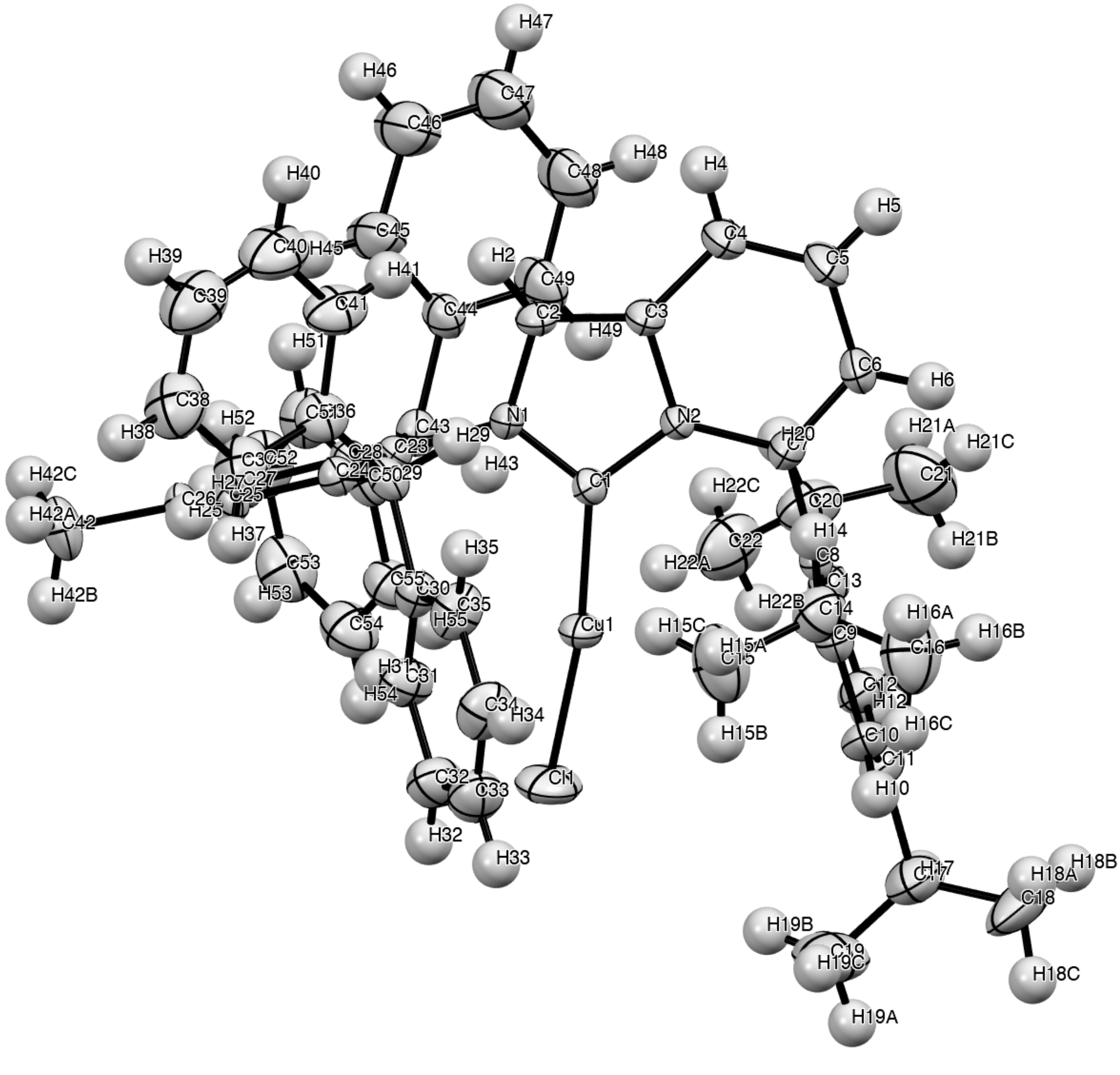

Selected bond lengths [Å] and angles [°]: Cu–C1, 1.886(5); Cu–Cl, 2.098(2); C1–N1, 1.342(7); C1–N2, 1.377(7); N1–C2, 1.380(7); C2–C3, 1.350(1); C3–N2, 1.413(7); C7–N2, 1.404(9); C23–N1, 1.460(7); C7–C8, 1.484(9); C1–Cu–Cl, 171.1(2); Cu–C1–N1, 125.9(4); Cu–C1–N2, 130.7(4); N1–C1–N2, 103.4(5); C1–N1–C2, 113.6(5); N1–C2–C3, 106.1(5); C2–C3–N2, 106.3(5); C3–N2–C1, 110.6(5); C1–N2–C7, 128.1(5); C1–N1–C23, 124.0(5).

ORTEP Structure of [(TrippIPr*)CuCl] – Side View

Figure S10. *ORTEP Structure of* ***[(TrippIPr*)CuCl]*** *(30% ellipsoids).* (Crystallographic data has been deposited with the Cambridge Crystallographic Data Center as supplementary publication no. CCDC 2271724).

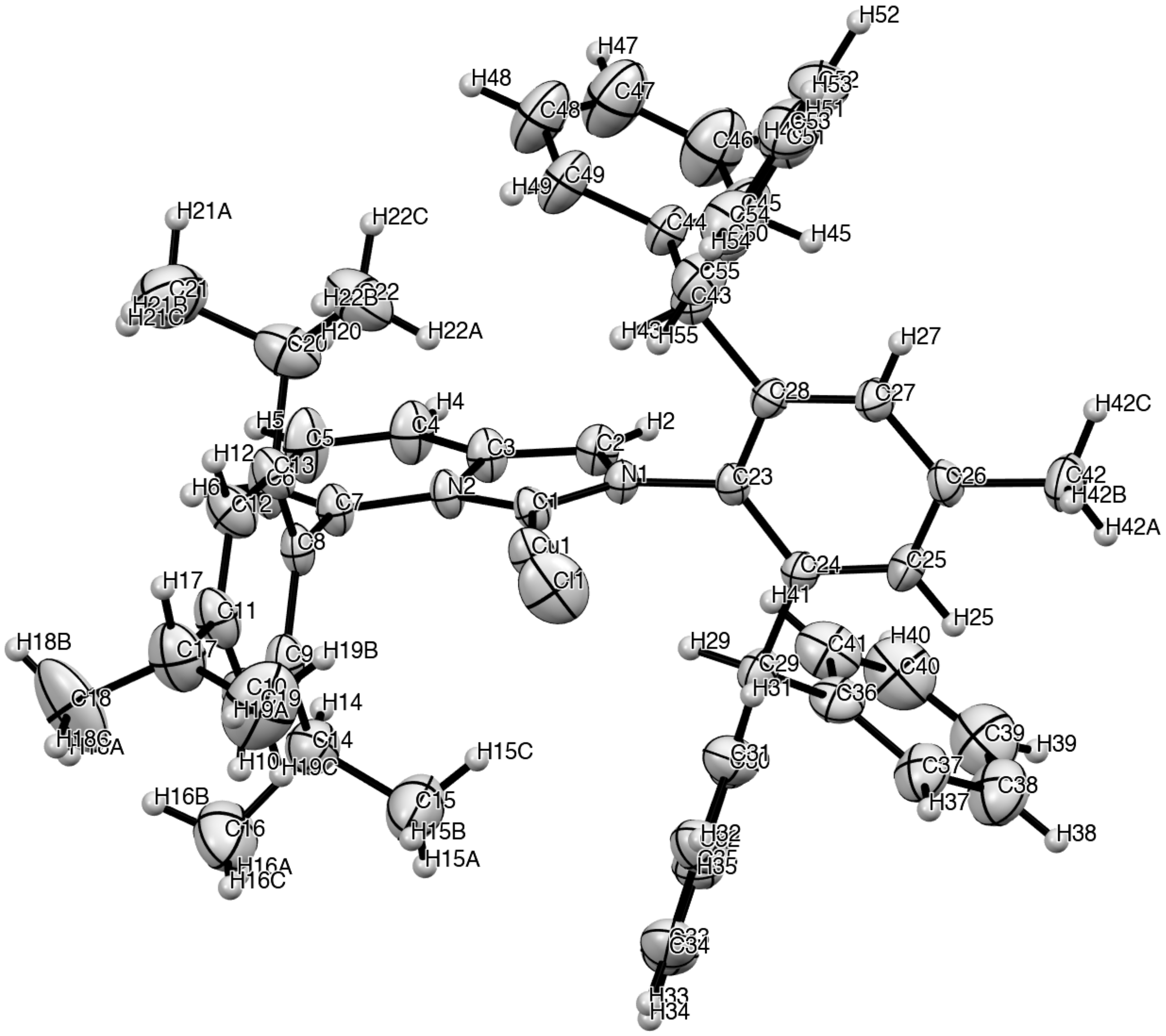

See, Figure S9 for selected bond lengths and angles.

Table S11. *Detailed Comparison of Bond Lengths and Angles of Palladium Complexes Supported by* ***IPr**, **IPr*** and **ImPyTrippIPr****.

Structure	$C_{carbene}$-Pd (Å)	Pd-Cl (Å)	C1^{cin}-Pd (Å)	C2^{cin}-Pd (Å)	C3^{cin}-Pd (Å)	$C_{carbene}$–Pd–Cl (°)	$C_{carbene}$–Pd–C1^{cin} (°)	$C_{carbene}$–Pd–C2^{cin} (°)	$C_{carbene}$–Pd–C3^{cin} (°)
(IPr)Pd(cin)Cl[a]	2.04	2.348	2.082	2.136	2.284	94.90	102.30	132.90	168.90
(IPr)Pd(cin)Cl*[b]	2.038	2.346	2.110	2.270	2.160	91.60	103.00	138.90	167.0
(ImPyTrippIPr)Pd(cin)Cl*	2.024	2.378	2.091	2.134	2.241	87.70	104.20	139.40	171.90

[a]Ref. 13 [b]Ref. 14

Table S12. *Detailed Comparison of Bond Lengths and Angles of Copper Complexes Supported by* ***IPr**, **IPr*** and **ImPyTrippIPr****.

Structure	$C_{carbene}$-Cu (Å)	Cu-Cl (Å)	$C_{carbene}$–Cu–Cl (°)
IPrCuCl[a]	1.953	2.089	180.00
*IPr*CuCl*[b]	1.868	2.094	176.21
*ImPyTrippIPr*CuCl*	1.886	2.098	171.10

[a]Ref. 15. [b]Ref. 16.

Synthesis of [(ImPyTrippIPr*)CuCl]

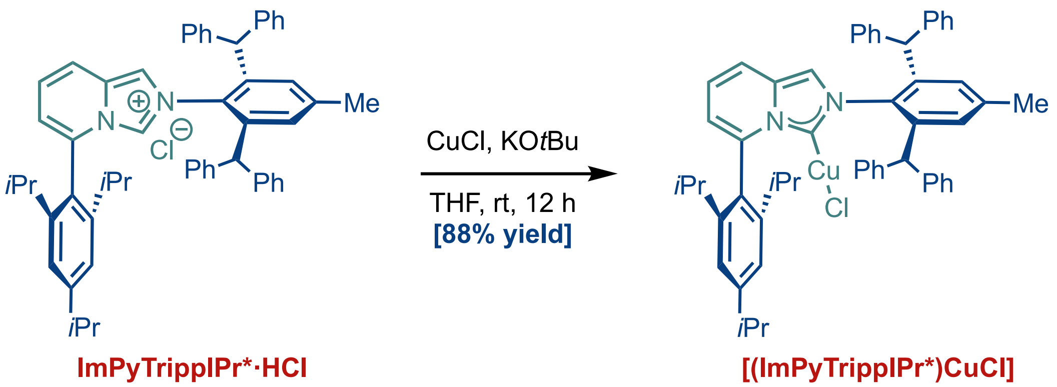

[(ImPyTrippIPr*)CuCl]. An oven-dried flask equipped with a stir bar was charged with ImPyTrippIPr*·HCl (78 mg, 0.10 mmol, 1.0 equiv), copper(I) chloride (19.8 mg, 0.20 mmol, 2.0 equiv) and KO*t*Bu (16.8 mg, 0.15 mmol, 1.5 equiv), placed under a positive pressure of argon and subjected to three evacuation/backfilling cycles under high vacuum. THF (1.0 mL, 0.10 M) were added and the resulting solution was stirred at room temperature for 12 h. The reaction mixture was diluted with CH_2Cl_2 and filtered through Celite. The solution was collected and concentrated. The residue was then loaded on to a short pad of silica gel and eluting with DCM until the product was completely recovered. After triturating with pentane and drying under high vacuum, the desired product was obtained as white solid. Yield 88% (74 mg). **^{1}H NMR (500 MHz, $CDCl_3$)** δ 7.25 – 7.14 (m, 14H), 7.04 – 7.00 (m, 4H), 6.87 – 6.83 (m, 2H), 6.80 – 6.76 (m, 4H), 6.67 (s, 2H), 6.53 – 6.49 (m, 1H), 5.64 (s, 1H), 5.19 (s, 2H), 3.09 – 2.99 (m, 1H), 2.54 – 2.44 (m, 2H), 2.18 (s, 3H), 1.41 (d, J = 6.9 Hz, 6H), 1.23 (d, J = 6.9 Hz, 6H), 1.17 (d, J = 6.8 Hz, 6H). **^{13}C NMR (125 MHz, $CDCl_3$)** δ 169.5, 152.4, 146.7, 142.8, 142.4, 141.0, 139.6, 138.1, 135.8, 130.1, 129.7, 129.6, 129.1, 128.5, 128.4, 128.2, 126.6, 126.5, 122.8, 122.5, 116.6, 116.0, 115.8, 51.6, 34.8, 31.8, 25.2, 24.5, 24.4, 22.0. **HRMS** calcd for $C_{55}H_{54}N_2Cu$ (M^+ – Cl) 805.3578, found 805.3581. Crystals suitable for X-ray crystallography were obtained from saturated hexane/DCM solution.

Synthesis of [(ImPyTrippIPr*)Rh(CO)$_2$Cl]

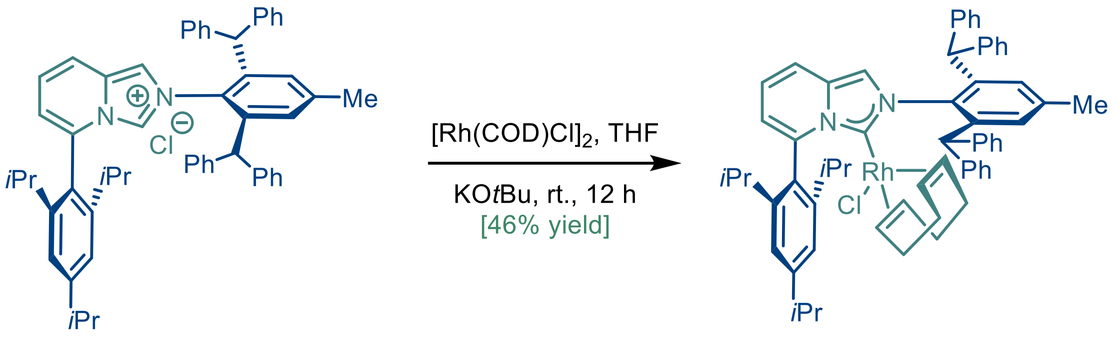

ImPyTrippIPr*·HCl [(ImPyTrippIPr*)Rh(COD)Cl]

[(ImPyTrippIPr*)Rh(COD)Cl]. An oven-dried flask equipped with a stir bar was charged with the corresponding NHC·HCl salt (78 mg, 0.10 mmol, 1.0 equiv), [Rh(COD)Cl]$_2$ (24.6 mg, 0.05 mmol, 1.0 equiv) and KO*t*Bu (16.8 mg, 0.15 mmol, 1.5 equiv), placed under a positive pressure of argon and subjected to three evacuation/backfilling cycles under high vacuum. THF (1.0 mL, 0.10 M) were added and the resulting solution was stirred at room temperature for 12 h. The reaction mixture was diluted with CH_2Cl_2 and filtered through Celite. The solution was collected and concentrated. The product was purified by column chromatography on silica gel to give the desired product as yellow solid. Yield 46% (46 mg). ^{1}H NMR (500 MHz, $CDCl_3$) δ 7.51 – 7.43 (m, 3H), 7.25 – 7.19 (m, 4H), 7.19 – 7.02 (m, 9H), 6.99 (s, 2H), 6.96 – 6.88 (m, 4H), 6.63 (s, 1H), 6.50 (d, J = 6.7 Hz, 2H), 6.46 (d, J = 7.6 Hz, 1H), 6.32 (s, 1H), 6.31 – 6.28 (m, 1H), 4.81 – 4.72 (m, 1H), 4.69 (s, 2H), 4.58 – 4.50 (m, 1H), 3.93 – 3.83 (m, 1H), 3.73 – 3.64 (m, 1H), 3.41 – 3.33 (m, 1H), 3.10 – 3.02 (m, 1H), 2.28 (s, 3H), 2.25 – 2.19 (m, 1H), 1.78 – 1.69 (m, 2H), 1.62 (d, J = 6.6 Hz, 4H), 1.41 (d, J = 7.0 Hz, 6H), 1.39 – 1.34 (m, 2H), 1.29 (d, J = 6.7 Hz, 3H), 1.27 – 1.26 (m, 2H), 1.20 (d, J = 6.7 Hz, 3H), 0.87 – 0.84 (m, 2H), 0.79 (d, J = 6.7 Hz, 3H). ^{13}C NMR (125 MHz, $CDCl_3$) δ 172.07 (d, J = 50.4 Hz), 149.76, 149.73, 146.34, 145.19, 144.52, 143.86, 143.84, 142.42, 142.31, 138.61, 137.55, 137.00, 132.91, 131.52, 130.45, 130.41, 130.04, 129.82, 129.02, 128.60, 128.44, 128.10, 127.97, 127.91, 126.63, 126.59, 125.99, 125.93, 123.05, 120.14, 119.40, 119.05, 117.42, 94.01 (d, J = 6.8 Hz), 93.48 (d, J = 8.2 Hz), 71.46 (d, J = 14.1 Hz), 66.54 (d, J = 14.1 Hz), 51.99, 50.34, 34.82, 34.62, 33.98, 31.74, 31.51, 30.72, 29.86, 28.76, 27.97, 26.94, 25.45, 24.24, 24.17, 24.06, 22.80, 22.62, 21.97, 14.27. HRMS calcd for $C_{63}H_{66}N_2Rh$ (M^+ – Cl) 953.4276, found 953.4283.

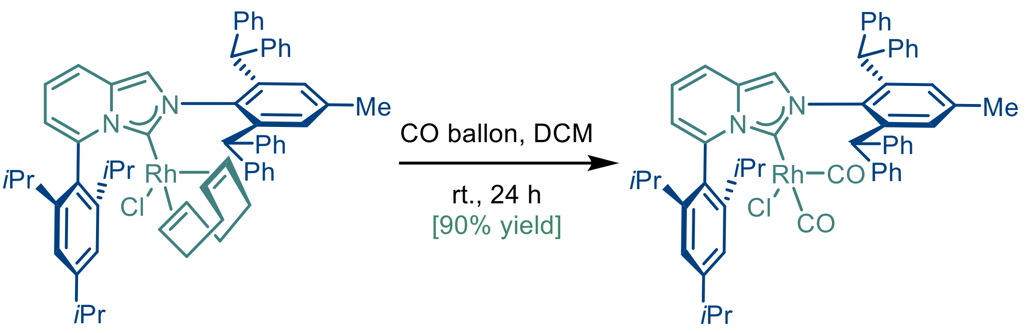

[(ImPyTrippIPr*)Rh(CO)$_2$Cl]. In a 10 mL vial, the corresponding (NHC)Rh(cod)Cl (40 mg, 0.04 mmol) was dissolved in dichloromethane (0.4 mL). Carbon monoxide was bubbled into the solution for 24 h at room temperature. Dichloromethane was then removed under vacuo. The product was obtained by trituration from hexane as yellow solid. Yield 90% (38 mg). ^{1}H NMR (500 MHz, CDCl$_3$) δ 7.32 (s, 1H), 7.26 – 7.05 (m, 15H), 6.99 (d, *J* = 7.5 Hz, 2H), 6.86 – 6.79 (m, 2H), 6.73 (s, 1H), 6.71 – 6.67 (m, 1H), 6.65 (s, 1H), 6.59 (d, *J* = 6.6 Hz, 1H), 6.57 – 6.48 (m, 3H), 6.40 (s, 1H), 5.03 (s, 1H), 4.86 (s, 1H), 3.27 – 3.16 (m, 1H), 3.05 – 2.96 (m, 1H), 2.26 – 2.17 (m, 4H), 1.55 (d, *J* = 6.6 Hz, 3H), 1.34 (dd, *J* = 7.0, 3.8 Hz, 6H), 1.23 – 1.16 (m, 6H), 0.94 (d, *J* = 6.7 Hz, 3H). ^{13}C NMR (125 MHz, CDCl$_3$) δ 185.90 (d, *J* = 55.9 Hz), 183.79 (d, *J* = 75.4 Hz), 167.85 (d, *J* = 44.1 Hz), 151.03, 149.31, 147.07, 144.50, 143.51, 143.25, 142.26, 141.81, 139.07, 136.94, 136.69, 131.29, 130.78, 130.46, 130.32, 129.91, 129.87, 129.80, 129.16, 128.25, 128.12, 128.06, 127.92, 126.78, 126.57, 126.24, 126.04, 122.33, 121.02, 120.75, 119.22, 118.77, 117.19, 51.62, 50.03, 34.86, 31.69, 31.00, 27.57, 26.46, 24.27, 23.89, 21.93, 21.90. HRMS calcd for $C_{57}H_{54}N_2O_2Rh$ (M^+ – Cl) 901.3235, found 901.3260.

Synthesis of [(ImPyTrippIPr*)Se]

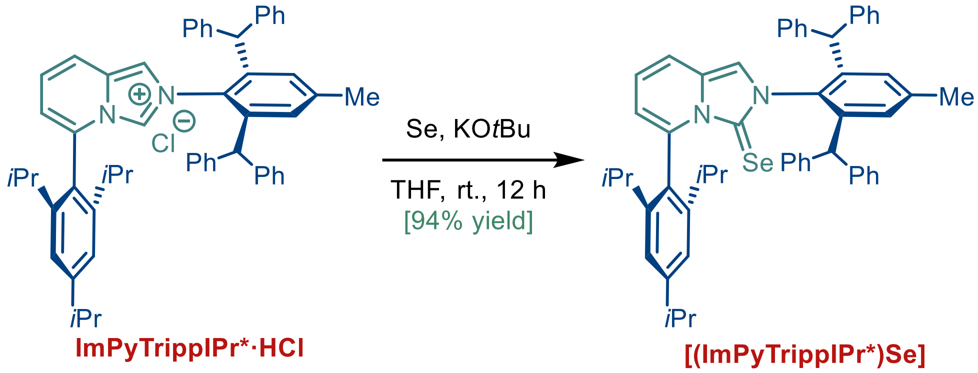

[(ImPyTrippIPr*)Se]. An oven-dried flask equipped with a stir bar was charged with the corresponding NHC·HCl salt (78 mg, 0.10 mmol, 1.0 equiv), selenium (16 mg, 0.20 mmol, 2.0 equiv) and KO*t*Bu (16.8 mg, 0.15 mmol, 1.5 equiv), placed under a positive pressure of argon and subjected to three evacuation/backfilling cycles under high vacuum. THF (1.0 mL, 0.10 M) were added and the resulting solution was stirred at room temperature for 12 h. The reaction mixture was diluted with CH_2Cl_2 and filtered through Celite. The solution was collected and concentrated. The product was obtained by trituration from hexane as yellow solid in 94% yield (77 mg). ^{1}H NMR (500 MHz, $CDCl_3$) δ 7.23 – 7.17 (m, 8H), 7.16 – 7.10 (m, 8H), 7.02 (s, 2H), 6.81 (d, J = 6.5 Hz, 4H), 6.68 (s, 2H), 6.48 (s, 2H), 6.24 – 6.19 (m, 1H), 5.26 (s, 1H), 5.19 (s, 2H), 3.01 (p, J = 7.0 Hz, 1H), 2.73 (p, J = 6.7 Hz, 2H), 2.18 (s, 3H), 1.37 (d, J = 6.9 Hz, 6H), 1.35 (d, J = 6.9 Hz, 6H), 1.16 (d, J = 6.8 Hz, 6H). ^{13}C NMR (125 MHz, $CDCl_3$) δ 150.01, 148.34, 147.55, 143.05, 142.67, 141.93, 138.72, 138.68, 134.59, 130.03, 129.94, 129.39, 129.28, 129.01, 128.27, 128.19, 126.39, 126.34, 120.68, 120.10, 117.31, 116.13, 112.80, 52.22, 34.45, 31.99, 25.20, 24.35, 22.88, 22.01. ^{77}Se NMR (95 MHz, $CDCl_3$) δ 170.40. HRMS calcd for $C_{55}H_{55}N_2Se$ (M^+ + H) 823.3525, found 822.3549.

References

1. Zhou, T.; Gao, P.; Bisz, E.; Dziuk, B.; Lalancette, R.; Szostak, R.; Szostak, M. Well-Defined, Air- and Moisture-Stable Palladium–Imidazo[1,5-a]pyridin-3-ylidene Complexes: A Versatile Catalyst Platform for Cross-Coupling Reactions by L-Shaped NHC Ligands. *Catl. Sci. Technol.* **2022**,12, 6581–6589.

2. Wang, J. Y.; Choi, K.; Zuend, S. J.; Borate, K.; Hartwig, J. F. Cross-Coupling between Hydrazine and Aryl Halides with Hydroxide Base at Low Loadings of Palladium by Rate-Determining Deprotonation of Bound Hydrazine. *Angew. Chem. Int. Ed.* **2020**, *60*, 399–408.

3. Wang, H.; Sun, X.; Zhang, S.; Liu, G.; Wang, C.; Zhu, L.; Zhang, H. Efficient Copper-Catalyzed Synthesis of Substituted Pyrazoles at Room Temperature. *Synlett* **2018**, *29*, 2689–2692.

4. Groves, L. M.; Schotten, C.; Beames, J.; Platts, J. A.; Coles, S. J.; Horton, P. N.; Browne, D. L.; Pope, S. J. A. From Ligand to Phosphor: Rapid, Machine-Assisted Synthesis of Substituted Iridium(III) Pyrazolate Complexes with Tuneable Luminescence. *Chem. - Eur. J.* **2017**, *23*, 9407–9418.

5. Sokolovs, I.; Lubriks, D.; Suna, E. Copper-Catalyzed Intermolecular C-H Amination of (Hetero)arenes via Transient Unsymmetrical λ^3-Iodanes. *J. Am. Chem. Soc.* **2014**, *136*, 6920–6928.

6. Yu, H.; Xiao, L.; Yang, X.; Shao, L. Controllable Access to Multi-Substituted Imidazoles via Palladium(II)-Catalyzed C–C Coupling and C–N Condensation Cascade Reactions. *Chem. Commun.* **2017**, *53*, 9745–9748.

7. Rodríguez-Villar, K.; Hernández-Campos, A.; Yépez-Mulia, L.; Sainz-Espuñes, T.d.R.; Soria-Arteche, O.; Palacios-Espinosa, J.F.; Cortés-Benítez, F.; Leyte-Lugo, M.; Varela-Petrissans, B.; Quintana-Salazar, E. A.; Pérez-Villanueva, J. Design, Synthesis and Anticandidal Evaluation of Indazole and Pyrazole Derivatives. *Pharmaceuticals* **2021**, *14*, 176.

8. Landstrom, E. B.; Akporji, N.; Lee, N. R.; Gabriel, C. M.; Braga, F. C.; Lipshutz, B. H. One-Pot Synthesis of Indoles and Pyrazoles via Pd-Catalyzed Couplings/Cyclizations Enabled by Aqueous Micellar Catalysis. *Org. Lett.* **2020**, *22*, 6543–6546.

9. Zhang, J.; Jia, R.-P.; Wang, D.-H. Copper-catalyzed C–N cross-coupling of arylboronic acids with N-acylpyrazoles. *Tetrahedron Lett.* **2016**, *57*, 3604–3607.

10. Nykaza, T. V.; Ramirez, A.; Harrison, T. S.; Luzung, M. R.; Radosevich, A. T. Biphilic Organophosphorus-Catalyzed Intramolecular Csp2-H Amination: Evidence for a Nitrenoid in Catalytic Cadogan Cyclizations. *J. Am. Chem. Soc.* **2018**, *140*, 3103– 3113.

11. Liao, W.; Du, H.; Chen, M.; Xiong, Y.; Zhou, H.; Qin, T.; Liu, B. Sequential Regioselective Arylation of Pyrazolones with Diaryliodonium Salts. *Org. Biomol. Chem.* **2024**, *22*, 708– 713.

12. Yang, N.; Yuan, G. A Multicomponent Electrosynthesis of 1,5-Disubstituted and 1-Aryl 1,2,4-Triazoles. *J. Org. Chem.* **2018**, *83*, 11963– 11969.

13. Marion, N.; Navarro, O.; Mei, J.; Stevens, E. D.; Scott, N. M.; Nolan, S. P. Modified (NHC)Pd(allyl)Cl (NHC = *N*-heterocyclic carbene) complexes for room-temperature Suzuki–Miyaura and Buchwald–Hartwig reactions. *J. Am. Chem. Soc.* **2006**, *128*, 4101– 4111.

14. Chartoire, A.; Lesieur, M.; Falivene, L.; Slawin, A. M. Z.; Cavallo, L.; Cazin, C. S. J.; Nolan, S. P. [Pd(IPr*)(cinnamyl)Cl]: An Efficient Pre-catalyst for the Preparation of Tetra-*ortho*-substituted Biaryls by Suzuki-Miyaura Cross-Coupling. *Chem. Eur. J.* **2012**, *18*, 4517– 4521.

15. Kaur, H.; Zinn, F. K.; Stevens, E. D.; Nolan, S. P. (NHC)Cu^{I} (NHC = N-Heterocyclic Carbene) Complexes as Efficient Catalysts for the Reduction of Carbonyl Compounds. *Organometallics* **2004**, *23*, 1157–1160.

16. Gómez-Suárez, A.; Ramón, R. S.; Songis, O.; Slawin, A. M. Z.; Cazin, C. S. J.; Nolan, S. P. Influence of a Very Bulky N-Heterocyclic Carbene in Gold-Mediated Catalysis. *Organometallics* **2011**, *30*, 5463– 5470.

Computational Methods

Geometry optimizations were performed with the B3LYP[1] functional with D3 version of Grimme's dispersion corrections[2] with Becke-Johnson damping.[3] The def2-SVP[4] basis set was employed for all atoms. Frequency calculations were performed at 298.15 K to verify that the stationary points were the local minima or first-order saddle points, i.e., transition states, and to obtain thermal corrections. Intrinsic Reaction Coordinate (IRC)[5] calculations were performed to determine the connectivity of the minima and transition states. Single point energy calculations were performed with the M06 functional,[6] and the def2-TZVP[7] for all atoms, with the inclusion of solvation energy corrections based on SMD implicit solvent model[8] with dioxane as solvent (ε = 2.2099). All the reported Gibbs free energy values are the sum of the electronic energy from the single point calculations and the thermal corrections obtained by the frequency calculations. In addition, to correct the Gibbs free energies under pressure of 1 atm to the standard state in solution (1 mol/L), a correction of RT $\ln(c_s/c_g)$ (1.89 kcal/mol) is added to energies of all species. c_s is the standard molar concentration in solution (1 mol/L), c_g is the standard molar concentration in the gas phase (0.0409 mol/L), and R is the gas constant. The 3D diagrams of molecules were generated using CYLView[9]. All of the calculations were performed with Gaussian 16.[10]

References

(1) (a) Lee, C.; Yang, W.; Parr, R. G. Development of the Colle-Salvetti Correlation-Energy Formula into a Functional of the Electron Density. Phys. Rev. B: Condens. *Matter Mater. Phys.* **1988**, *37*, 785−789. (b) Becke, A. D. Density-Functional Thermochemistry. III. The Role of Exact Exchange. *J. Chem. Phys.* **1993**, *98*, 5648−5652.

(2) Grimme, S.; Antony, J.; Ehrlich, S.; Krieg, H. A Consistent and Accurate Ab Initio Parametrization of Density Functional Dispersion correction (DFT-D) for the 94 Elements H-Pu. *J. Chem. Phys.* **2010**, *132*, 154104.

(3) Grimme, S.; Ehrlich, S.; Goerigk, L. Effect of the Damping Function in Dispersion Corrected Density Functional Theory. *J. Comp. Chem.* **2011**, *32*, 1456-1465.

(4) Matsuo, Y.; Nakamura, E. Ruthenium(II) Complexes of Pentamethylated [60]Fullerene. Alkyl, Alkynyl, Chloro, Isocyanide, and Phosphine Complexes. *Organometallics*, **2003**, *22*, 2554−2563.

(5) Fukui, K. The Path of Chemical Reactions - the IRC Approach. *Acc. Chem. Res.* **1981**, *14*, 363-368.

(6) Zhao, Y.; Truhlar, D. G. The M06 Suite of Density Functionals for Main Group Thermochemistry, Thermochemical Kinetics, Noncovalent Interactions, Excited States, and Transition Elements: Two New Functionals and Systematic Testing of Four M06-Class Functionals and 12 Other Functionals. *Theor. Chem. Acc.* **2008**, *120*, 215−241.

(7) Weigend, F.; Ahlrichs, R. Balanced Basis Sets of Split Valence, Triple Zeta Valence and Quadruple Zeta Valence Quality for H to Rn: Design and Assessment of Accuracy. *Phys. Chem. Chem. Phys.* **2005**, *7*, 3297−3305.

(8) Marenich, A. V.; Cramer, C. J.; Truhlar, D. G. Universal Solvation Model Based on Solute Electron Density and on a Continuum Model of the Solvent Defined by the Bulk Dielectric Constant and Atomic Surface Tensions. *J. Phys. Chem. B* **2009**, *113*, 6378−6396.

(9) Legault, C. Y. *CYLView, 1.0b*; Universitéde Sherbrooke: Québec, Montreal, Canada, 2009; (http://www.cylview.org).

(10) *Gaussian 16*, Revision A.03, Frisch, M. J., Trucks, G. W., Schlegel, H. B., Scuseria, G. E., Robb, M.A., Cheeseman, J. R., Scalmani, G., Barone, V., Petersson, G. A., Nakatsuji, H., Li, X., Caricato, M., Marenich, A. V., Bloino, J., Janesko, B. G., Gomperts, R., Mennucci, B., Hratchian, H. P., Ortiz, J.V., Izmaylov, A. F., Sonnenberg, J. L., Williams-Young, D., Ding, F., Lipparini, F., Egidi, F., Goings, J., Peng, B., Petrone, A., Henderson, T., Ranasinghe, D., Zakrzewski, V. G., Gao, J., Rega, N., Zheng, G., Liang, W., Hada, M., Ehara, M., Toyota, K., Fukuda, R., Hasegawa, J., Ishida, M., Nakajima, T.,

Honda, Y., Kitao, O., Nakai, H., Vreven, T., Throssell, K., Montgomery, J. A. Jr., Peralta, J. E., Ogliaro, F., Bearpark, M. J., Heyd, J. J., Brothers, E. N., Kudin, K. N., Staroverov, V. N., Keith, T. A., Kobayashi, R., Normand, J., Raghavachari, K., Rendell, A. P., Burant, J. C., Iyengar, S. S., Tomasi, J., Cossi, M., Millam, J. M., Klene, M., Adamo, C., Cammi, R., Ochterski, J. W., Martin, R. L., Morokuma, K., Farkas, O., Foresman, J. B. & Fox, D. J., Gaussian, Inc., Wallingford CT, 2016.

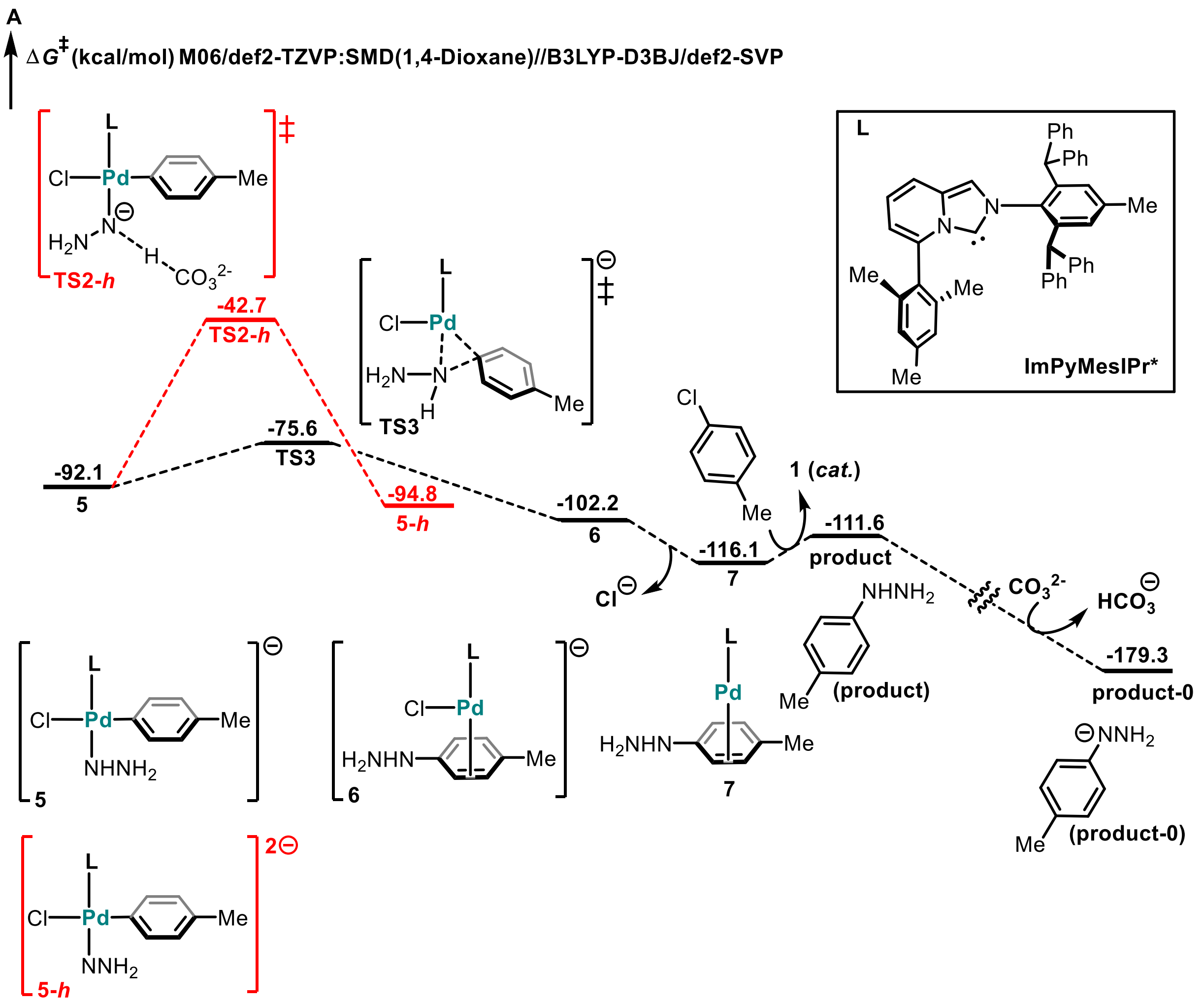

Figure S11. Potential pathways for the formation of more stable **product-0**.

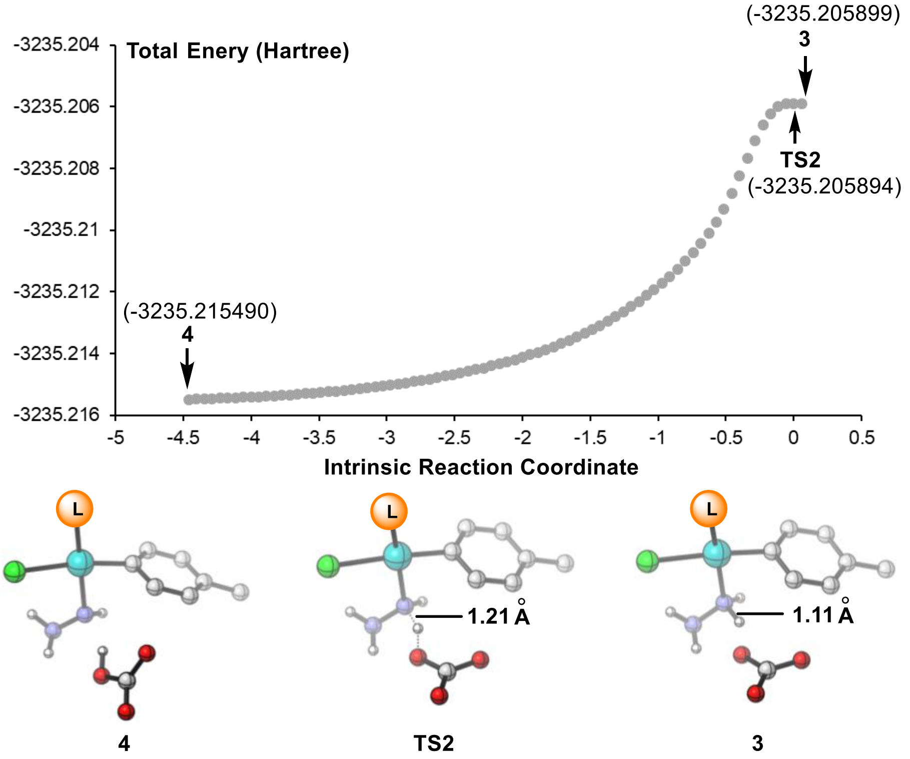

Figure S12. The structure of **TS2** IRC analysis.

Table S13: Zero-point correction (ZPE), thermal correction to enthalpy (TCH), thermal correction to Gibbs free energy (TCG), energies (E), enthalpies (H), and Gibbs free energies (G) (in Hartree) of the structures calculated at the M06/def2-TZVP: SMD(1,4-Dioxane)//B3LYP-D3BJ/def2-SVP level of theory.

species	ZPE	tcH	tcG	E	H	G	Imaginary Frequency
**1**	0.890173	0.945895	0.793368	-2860.221481	-2859.275586	-2859.428113	7.6
**TS1**	0.888965	0.944279	0.793981	-2860.196874	-2859.252595	-2859.402893	-155.8
**2**	0.891275	0.946734	0.795756	-2860.23071	-2859.283976	-2859.434954	5.2
**3**	0.960928	1.022864	0.863034	-3236.026334	-3235.00347	-3235.1633	15.9
**TS2**	0.958072	1.019688	0.860526	-3236.024318	-3235.00463	-3235.163792	-391.8
**4**	0.96237	1.024446	0.864985	-3236.042457	-3235.018011	-3235.177472	12
**5**	0.933152	0.991765	0.837044	-2971.570103	-2970.578338	-2970.733059	14.4
**TS2-h**	0.938032	1.000003	0.840062	-3235.344323	-3234.34432	-3234.504261	-1066.3
**5-h**	0.912133	0.970857	0.815851	-2970.906909	-2969.936052	-2970.091058	10.8
**TS3**	0.931843	0.989759	0.836893	-2971.543596	-2970.553837	-2970.706703	-420.7

**TS3-t**	0.931927	0.988443	0.835601	-2511.212583	-2510.22414	-2510.376982	-399.5
**TS3-tx**	0.931291	0.988158	0.834166	-2511.198309	-2510.210151	-2510.364143	-452.2
**TS3-x**	0.931195	0.989539	0.833637	-2971.530026	-2970.540487	-2970.696389	-448.5
**6**	0.933914	0.992024	0.839116	-2971.588234	-2970.59621	-2970.749118	11
**7**	0.933848	0.990858	0.833004	-2511.278407	-2510.287549	-2510.445403	2.7
**product**	0.161271	0.171161	0.126017	-382.091514	-381.920353	-381.965497	11.3
**product-0**	0.146174	0.155697	0.112797	-381.539793	-381.384096	-381.426996	59
**4-Chlorotoluene**	0.118123	0.126481	0.083668	-731.039028	-730.912547	-730.95536	6.5
**NH_2NH_2**	0.052636	0.057201	0.029435	-111.833854	-111.776653	-111.804419	137.2
**CO_3^{2-}**	0.014254	0.018368	-0.011304	-263.838546	-263.820178	-263.84985	683.2
**HCO_3^-**	0.026545	0.030996	0.000784	-264.496876	-264.46588	-264.496092	549
**Cl^-**	0	0.00236	-0.015023	-460.310936	-460.308576	-460.325959	

The Cartesian Coordinates of the stationary points discussed in the text

1

N	-1.06181000	0.51353200	0.91019100
C	0.00467600	-0.29234700	1.19285100
N	-0.00380800	-0.34135300	2.56544000
C	-1.74812200	0.93932800	2.03263600
H	-2.61332300	1.58931800	1.98094100
C	-1.08362100	0.40679300	3.10955200
C	-1.24280700	0.46596000	4.52296600
H	-2.07077000	1.04447700	4.93457900
C	-0.35118400	-0.19778300	5.31693400
H	-0.44996400	-0.16720100	6.40370300
C	0.73663800	-0.93240000	4.73227200
H	1.45870500	-1.44320900	5.36982200
C	2.05818300	-1.70990300	2.73752300
C	0.91642700	-1.00215600	3.38047300
C	1.96138800	-3.08512800	2.45040100
C	3.06304000	-3.73302200	1.87809900
H	2.98421800	-4.79768200	1.64074000
C	4.24860100	-3.05030500	1.58506500
C	4.31586400	-1.68259900	1.87645400
H	5.22225800	-1.12679100	1.62728100
C	3.23793900	-0.99528600	2.43760700
C	-1.36437100	0.84539500	-0.45142700

C	-2.34981500	0.11228000	-1.11921900
C	-2.58192200	0.39655900	-2.47021800
H	-3.33775900	-0.17700800	-3.00996100
C	-1.83584700	1.36570100	-3.14765100
C	-0.85074800	2.07791200	-2.44430800
H	-0.25082000	2.82202400	-2.97011100
C	-0.60207800	1.84011000	-1.09373100
C	-3.11268800	-0.96440000	-0.36134100
H	-2.41333300	-1.35590900	0.39006200
C	-3.46442200	-2.15321100	-1.24297700
C	-4.71554700	-2.29646800	-1.85490500
H	-5.49445500	-1.55415100	-1.66790100
C	-4.97432300	-3.38602700	-2.69250000
H	-5.95503100	-3.48595200	-3.16434900
C	-3.98690700	-4.34773900	-2.92138400
H	-4.19157600	-5.20104100	-3.57249100
C	-2.73749700	-4.21483000	-2.30711100
H	-1.95995500	-4.96354700	-2.47822300
C	-2.47884400	-3.12428800	-1.47573700
H	-1.49741900	-3.00500100	-1.00578300
C	-4.29979700	-0.40684300	0.41437000
C	-4.93812800	0.78782200	0.05951400
H	-4.58022100	1.35371000	-0.80234900
C	-6.01870900	1.26928700	0.80718900
H	-6.50276300	2.20519400	0.51697200

C	-6.47466100	0.56179500	1.92088000
H	-7.31517000	0.93986500	2.50777200
C	-5.84506900	-0.63503900	2.28119500
H	-6.19173900	-1.19548200	3.15300000
C	-4.76927500	-1.11190600	1.53312000
H	-4.27594500	-2.04428500	1.82020000
C	-2.04103000	1.61490800	-4.61947400
H	-2.95574200	1.13013600	-4.99005700
H	-1.19275800	1.21592100	-5.20176100
H	-2.10508100	2.69165300	-4.84234100
C	0.46652300	2.56708300	-0.28620400
H	1.05360100	1.76589500	0.18577300
C	-0.14414800	3.38548500	0.84507500
C	-1.36848500	4.05096600	0.69946500
H	-1.90562300	3.98618100	-0.24941200
C	-1.92476600	4.76297000	1.76582300
H	-2.88364000	5.27150200	1.63704300
C	-1.26391600	4.81603600	2.99599600
H	-1.70292800	5.36358000	3.83349900
C	-0.03736100	4.16180500	3.14744600
H	0.48500800	4.19096500	4.10679200
C	0.51713000	3.45770500	2.07801800
H	1.46669100	2.93338600	2.20533400
C	1.46097400	3.36955900	-1.10605300
C	1.14628700	4.61926400	-1.65930400

H	0.15794300	5.05213300	-1.48974100
C	2.09014500	5.32032900	-2.41339900
H	1.83008400	6.29261600	-2.83951800
C	3.36762500	4.78675200	-2.61562100
H	4.10650300	5.33891300	-3.20158500
C	3.69481100	3.55026900	-2.05385000
H	4.69029100	3.12105900	-2.19213800
C	2.74579500	2.84939300	-1.30569900
H	3.00400200	1.88280400	-0.87060700
C	0.66947700	-3.82313200	2.68587000
H	0.32941600	-3.73614600	3.72910600
H	0.76884700	-4.89040500	2.44208700
H	-0.12547700	-3.40109700	2.04810200
C	3.30900900	0.49590500	2.63264200
H	2.90930800	0.80855000	3.60890600
H	2.70666500	0.99547700	1.85541900
H	4.34047900	0.86284000	2.54035700
C	5.42661100	-3.74605600	0.95166800
H	6.26542900	-3.83963400	1.66230200
H	5.79857200	-3.17245700	0.08735500
H	5.16474900	-4.75758300	0.60804400
Pd	1.16582700	-1.15408000	-0.23761200
C	1.01206000	-1.46507400	-3.27436300
C	1.71672100	-0.35031400	-3.71446900
C	2.92353300	0.04700600	-3.10797900

C	3.42637100	-0.68962300	-2.04987700
C	2.76912900	-1.85407900	-1.57301500
C	1.54672900	-2.22325300	-2.19091000
H	1.32832200	0.23732000	-4.54975900
H	3.45502300	0.92940600	-3.46208500
H	3.29349900	-2.53284400	-0.89879100
H	1.11482900	-3.20434900	-1.97477600
C	-0.27031400	-1.89298300	-3.93141400
H	-1.12933300	-1.70571800	-3.27195100
H	-0.26907700	-2.97203100	-4.15507000
H	-0.44188000	-1.34701600	-4.87034800
Cl	4.93981900	-0.20088500	-1.29912600

10

N	1.40387400	0.80755000	-0.67005800
C	0.72225500	-0.28456500	-1.13277400
N	1.35735000	-0.56597300	-2.32436200
C	2.38002100	1.25350400	-1.54184800
H	2.98066700	2.13263800	-1.34842200
C	2.36502400	0.40041800	-2.60781500
C	3.09696200	0.34309200	-3.82465200
H	3.84809200	1.11083800	-4.01415200
C	2.82983700	-0.66364500	-4.70550400
H	3.37103600	-0.73903200	-5.65091100

C	1.84685100	-1.65293000	-4.37454700
H	1.67707200	-2.49247100	-5.04798300
C	0.20774700	-2.73765300	-2.86249400
C	1.12353900	-1.62071200	-3.21371900
C	0.55862700	-3.61535800	-1.81363400
C	-0.25149700	-4.72896700	-1.56284500
H	0.02609100	-5.40847400	-0.75264000
C	-1.40753100	-4.98327300	-2.30544600
C	-1.71390900	-4.11777600	-3.36145400
H	-2.61233100	-4.30117400	-3.95767000
C	-0.92085100	-3.00867900	-3.67143700
C	1.17482500	1.39966700	0.61732000
C	1.94342900	0.94617000	1.70199600
C	1.72232800	1.52373300	2.95509200
H	2.28931700	1.16134000	3.81369300
C	0.74544300	2.50908200	3.13797400
C	-0.00033400	2.93913100	2.03156700
H	-0.77174800	3.69914100	2.16410500
C	0.20565900	2.40761600	0.75489400
C	2.96001100	-0.16772900	1.47365500
H	2.45899100	-0.89232800	0.81873300
C	0.45131900	3.05337800	4.51269500
H	-0.38114400	2.49660900	4.97698000
H	0.15101800	4.11179500	4.47359600
H	1.32159300	2.96128400	5.18013600

C	-0.55122600	2.88379900	-0.47924900
C	1.76903600	-3.37172400	-0.95257700
H	1.52870200	-2.61640700	-0.18797300
C	-2.32778000	-6.12374600	-1.95350100
H	-1.82270200	-6.87438400	-1.32702000
H	-2.71521900	-6.62641800	-2.85406900
H	-3.20054500	-5.75243700	-1.38842800
Pd	-1.13648200	-0.84269300	-0.54370100
C	-0.71908600	-1.66077600	1.25165800
C	-0.73239200	-3.04936800	1.45981900
C	-0.61329900	-3.60898900	2.73943300
C	-0.49083400	-2.80274200	3.87809800
C	-0.47559900	-1.41502000	3.68021700
C	-0.58360400	-0.86266300	2.40272900
H	-0.87056800	-3.71813500	0.61023100
H	-0.63530000	-4.69884400	2.85344200
H	-0.37601500	-0.74808400	4.54342000
H	-0.58947500	0.22151000	2.31403200
C	-0.39241800	-3.38875700	5.26443400
H	-0.27187100	-4.48331900	5.23152900
H	-1.29736300	-3.17434300	5.85999900
H	0.46472500	-2.97157600	5.81974900
N	-3.08699200	-1.39874600	-0.21668900
N	-3.56114200	-2.22454600	-1.26349600
H	-2.95979500	-3.04727700	-1.29381900

H	-3.39410200	-1.73451200	-2.15103000
H	-0.88393800	1.98067400	-1.01016400
C	-1.29470800	-2.13485700	-4.84116400
H	-2.32412500	-2.34959900	-5.16329000
H	-0.63146300	-2.30530000	-5.70542400
H	-1.24246400	-1.07322400	-4.56078300
H	2.62360500	-3.00034700	-1.53893800
H	2.07391900	-4.28901800	-0.42949200
C	4.18970200	0.32121600	0.71824100
C	4.69390300	1.61757900	0.88120100
C	4.83429800	-0.54286800	-0.17706200
C	5.80613500	2.04734300	0.15127800
H	4.18695100	2.30673500	1.55993500
C	5.94131100	-0.11748800	-0.91087200
H	4.43963000	-1.55202500	-0.31822000
C	6.43067000	1.18355000	-0.75196400
H	6.17837300	3.06700100	0.28093100
H	6.41549100	-0.79897900	-1.62167500
H	7.29200900	1.52277500	-1.33303900
C	3.34359900	-0.94087200	2.72677400
C	4.35893900	-0.50462100	3.59056700
C	2.66670000	-2.13020700	3.02640600
C	4.68382300	-1.23595100	4.73560400
H	4.90813300	0.41026100	3.35837100
C	2.99866000	-2.86897600	4.16416700

H	1.86628000	-2.47603200	2.37086200
C	4.00504900	-2.42394500	5.02495900
H	5.47631700	-0.88060300	5.39983300
H	2.45622600	-3.79234800	4.37656000
H	4.26233100	-3.00123300	5.91703100
C	0.37457700	3.61740000	-1.43986400
C	0.24219200	3.38755200	-2.81727500
C	1.38246500	4.48204100	-0.99127000
C	1.10756000	4.00303800	-3.72302100
H	-0.51689600	2.67798900	-3.15832600
C	2.24833300	5.10108500	-1.89807800
H	1.51116100	4.64597500	0.08134500
C	2.11559300	4.86051700	-3.26864900
H	1.00499800	3.79678200	-4.79176400
H	3.03479500	5.76674400	-1.53142200
H	2.79795700	5.33523600	-3.97906600
C	-1.82188700	3.65783600	-0.16553900
C	-3.03712700	2.96104600	-0.14591700
C	-1.82098000	5.02940000	0.13029100
C	-4.22622200	3.61181300	0.19240500
H	-3.05069900	1.90039100	-0.39642600
C	-3.01000800	5.68458700	0.46148900
H	-0.88459200	5.59091900	0.09458000
C	-4.21572700	4.97501700	0.49843300
H	-5.15289800	3.03523800	0.22713200

H	-2.99513800	6.75419100	0.69019000
H	-5.14538100	5.48664600	0.76300400
C	-4.04552700	-0.73795300	0.48578100
C	-5.44338000	-0.91735200	0.27215900
C	-3.68891900	0.19598600	1.50180700
C	-6.38555300	-0.21885400	1.02374900
H	-5.75158200	-1.61754800	-0.50274500
C	-4.64739900	0.87402300	2.24013900
H	-2.63435000	0.39192900	1.67750800
C	-6.02685900	0.69547800	2.02694200
H	-7.44958100	-0.38709200	0.81624600
H	-4.31079700	1.59069600	2.99805100
C	-7.05226400	1.45217800	2.83364400
H	-6.88130600	2.54435100	2.80457400
H	-7.04767600	1.16414400	3.90247500
H	-8.07126000	1.27042800	2.45428900
Cl	-1.88609500	0.37815500	-2.61211100

2

N	0.45167600	1.02287500	-0.56622200
C	-0.62352400	0.27459200	-0.95282300
N	-1.06069600	0.90538300	-2.09441800
C	0.67858400	2.09936000	-1.40515200
H	1.46561000	2.81842900	-1.22601900
C	-0.27102200	2.04367200	-2.38955900

C	-0.58696300	2.84577300	-3.52137600
H	0.03729400	3.71368700	-3.73548700
C	-1.66308400	2.50741900	-4.28990600
H	-1.93310100	3.10594100	-5.16171500
C	-2.45451200	1.35708700	-3.95457000
H	-3.31730900	1.08840100	-4.56447400
C	-2.94847900	-0.63980100	-2.50261300
C	-2.16637700	0.56529700	-2.88090800
C	-2.46039200	-1.91795800	-2.87803800
C	-3.20160300	-3.04605100	-2.53195200
H	-2.81544700	-4.03396000	-2.79685400
C	-4.40717700	-2.94997000	-1.82038600
C	-4.88361700	-1.68116600	-1.49383700
H	-5.81204900	-1.59020600	-0.92509200
C	-4.17592900	-0.51504700	-1.81661400
C	1.21877300	0.81722500	0.63630000
C	2.52041800	0.29976800	0.53158200
C	3.25990300	0.13373300	1.70580500
H	4.26090100	-0.29497900	1.64073100
C	2.74081500	0.48572000	2.95591600
C	1.44558500	1.01313700	3.02027900
H	1.02157700	1.27557700	3.99009600
C	0.66021100	1.18040000	1.87679100
C	3.10734600	-0.05061100	-0.83371300
H	2.28762500	-0.49948900	-1.41499300

C	4.20291900	-1.10745600	-0.75059100
C	3.82719700	-2.45294200	-0.64850500
H	2.76868700	-2.71336400	-0.64428300
C	4.78996300	-3.45620000	-0.53422000
H	4.47449800	-4.49913400	-0.45804000
C	6.14900100	-3.12653700	-0.51758200
H	6.90529900	-3.91026300	-0.42913200
C	6.53284600	-1.78741000	-0.62091000
H	7.59226700	-1.51914300	-0.61439500
C	5.56590800	-0.78392900	-0.74047900
H	5.87753500	0.25793300	-0.83203900
C	3.55934500	1.18735700	-1.59803600
C	4.07683400	2.31377500	-0.94535200
H	4.15982700	2.31284600	0.14331500
C	4.45657400	3.44698900	-1.67164400
H	4.85098600	4.32026500	-1.14617600
C	4.32677100	3.46667600	-3.06271200
H	4.61601200	4.35440200	-3.63014300
C	3.82686900	2.33950300	-3.72373700
H	3.72306000	2.34340600	-4.81155800
C	3.45151400	1.21051900	-2.99502600
H	3.04850100	0.33645200	-3.51313100
C	3.53667000	0.24738700	4.21267800
H	3.25118200	0.94724100	5.01192100
H	4.61785100	0.34401300	4.03259600

H	3.35908500	-0.77404300	4.59182600
C	-0.78617800	1.66119000	1.95240000
H	-1.36852900	0.86655000	1.45503500
C	-1.02187300	2.92621200	1.14525900
C	-0.09504700	3.97656800	1.11801600
H	0.82780300	3.89280700	1.69704100
C	-0.32430300	5.10904600	0.33172600
H	0.40957100	5.91893000	0.31939600
C	-1.48009300	5.20045300	-0.44842400
H	-1.65356200	6.07885600	-1.07456300
C	-2.41279900	4.15893100	-0.42329900
H	-3.31796000	4.21847600	-1.03269800
C	-2.18600300	3.03670800	0.37432500
H	-2.90917100	2.21914900	0.38989000
C	-1.34047900	1.72761700	3.36583400
C	-1.17585800	2.85343600	4.18406600
H	-0.66818500	3.73761600	3.79327100
C	-1.66345200	2.85692400	5.49348100
H	-1.52853500	3.74220500	6.12018900
C	-2.32778500	1.73521900	5.99863100
H	-2.71227100	1.73964800	7.02159700
C	-2.50707800	0.61390800	5.18387300
H	-3.03723800	-0.26347000	5.56201400
C	-2.01836400	0.60924600	3.87495400
H	-2.18098600	-0.26561500	3.23800100

C	-1.14794300	-2.04778400	-3.60652800
H	-0.95849900	-3.08988600	-3.89890300
H	-0.31238800	-1.72066400	-2.96626900
H	-1.12361600	-1.42393500	-4.51370500
C	-4.73478200	0.81932100	-1.38585100
H	-5.69136500	1.02760600	-1.89237900
H	-4.05354600	1.65005300	-1.60729800
H	-4.93691800	0.81432600	-0.30289300
C	-5.11698200	-4.18201100	-1.33255400
H	-4.86662700	-5.06738800	-1.93557100
H	-6.20982500	-4.05168700	-1.33599700
H	-4.80917500	-4.37701300	-0.29107400
Pd	-1.72832300	-1.06123400	0.06474500
C	-0.22682500	-2.20937000	0.63170000
C	0.11775800	-3.27908000	-0.20007200
C	1.03307800	-4.24365900	0.24318500
C	1.64266600	-4.14768800	1.50009700
C	1.28572300	-3.06312300	2.31576400
C	0.35209700	-2.11244300	1.89953200
H	-0.34447800	-3.39074800	-1.18156400
H	1.28135300	-5.08340600	-0.41313500
H	1.73337100	-2.96585200	3.30964400
H	0.06769500	-1.31301200	2.58045400
C	2.66066000	-5.15765400	1.96537500
H	3.66644200	-4.70828600	2.02672400

H	2.72079900	-6.01539700	1.27862100
H	2.41590100	-5.54448000	2.96800300
Cl	-3.22464500	-2.21553500	1.44598900

3

N	0.44058800	-0.56179800	1.23016100
C	-0.46497800	-1.18947100	0.41438600
N	-0.82785600	-2.30479500	1.13307700
C	0.67742700	-1.25878900	2.39742500
H	1.39428200	-0.93199900	3.13792000
C	-0.09848700	-2.38147600	2.35612100
C	-0.23065100	-3.51495500	3.20160400
H	0.37239400	-3.55287600	4.10968600
C	-1.08510200	-4.51690200	2.83577000
H	-1.20306100	-5.40834100	3.45611800
C	-1.86565500	-4.37765900	1.64118600
H	-2.61584700	-5.12873500	1.39174000
C	-2.73525400	-3.09618500	-0.29615900
C	-1.76056500	-3.29462900	0.80971400
C	-3.75693700	-2.13669400	-0.12028600
C	-4.76749600	-2.03638000	-1.08148600
H	-5.55611100	-1.29148700	-0.93978300
C	-4.77417900	-2.83548800	-2.22839900
C	-3.76357300	-3.79057800	-2.37066200

H	-3.75385100	-4.43097500	-3.25763700
C	-2.75309600	-3.95735700	-1.41553800
C	1.05930800	0.70673500	0.90963300
C	0.39244000	1.87704600	1.31372100
C	0.93434900	3.11115500	0.95187800
H	0.39892000	4.02476800	1.21352000
C	2.10810800	3.20320300	0.19140100
C	2.78013000	2.01612000	-0.14384700
H	3.65677500	2.09386400	-0.78666400
C	2.28616900	0.75804000	0.21217100
C	-0.89239300	1.75650400	2.12844800
H	-1.51672700	1.01691200	1.60799900
C	2.57019300	4.51456900	-0.36072500
H	2.17420500	4.54850900	-1.41027000
H	3.66615800	4.57098600	-0.42828000
H	2.18223000	5.36356800	0.22807800
C	3.02635100	-0.53789900	-0.13458200
C	-3.75309500	-1.20178900	1.05932200
H	-2.99622200	-0.41639000	0.90305200
C	-5.81556300	-2.63356700	-3.29981100
H	-6.79711300	-2.37646900	-2.86924200
H	-5.93663800	-3.53272000	-3.92414000
H	-5.52754800	-1.80371500	-3.96879000
Pd	-0.52155900	-0.76180800	-1.54922000
C	-1.63264300	0.91520300	-1.50492600

C	-3.03262300	0.91872200	-1.61190000
C	-3.74047000	2.10107800	-1.88696000
C	-3.07006200	3.31584600	-2.08265600
C	-1.67024300	3.30958200	-1.98306200
C	-0.96925400	2.14142400	-1.68483800
H	-3.58710200	-0.01371000	-1.52927300
H	-4.83395400	2.06562300	-1.97214500
H	-1.06486800	4.19436300	-2.19775300
H	0.11703400	2.21910900	-1.71120000
C	-3.81203500	4.58745000	-2.41621300
H	-4.90242800	4.42451800	-2.45215900
H	-3.49911200	4.99134400	-3.39504800
H	-3.61370800	5.37866600	-1.67208700
N	0.58117900	-0.92176400	-4.44401000
H	2.31551500	-1.15945200	-0.70182100
C	-1.70811200	-5.02292500	-1.61122200
H	-1.85355800	-5.53332800	-2.57514900
H	-1.74067900	-5.78176400	-0.81326700
H	-0.70300100	-4.56749300	-1.60595200
H	-3.50444100	-1.72214000	1.99728500
H	-4.72705700	-0.70519500	1.17607800
C	-0.63600000	1.21011500	3.52803300
C	0.54862100	1.49245100	4.22155000
C	-1.59472000	0.39557900	4.14531100
C	0.77761800	0.95629100	5.49163900

H	1.31092100	2.10443000	3.73478100
C	-1.36845700	-0.14764400	5.41040600
H	-2.51530900	0.16015800	3.60574700
C	-0.17681900	0.12811100	6.08936200
H	1.71621500	1.17299900	6.00925200
H	-2.11765100	-0.80425600	5.86137000
H	0.00962600	-0.30678900	7.07529200
C	-1.72444300	3.02931100	2.19020200
C	-1.50002800	4.02466200	3.15264100
C	-2.74959700	3.21733300	1.25516500
C	-2.27933900	5.18390400	3.17663800
H	-0.70796900	3.88983800	3.89171700
C	-3.53539100	4.37140400	1.28203400
H	-2.92576800	2.45949500	0.49178400
C	-3.30392400	5.36059100	2.24142500
H	-2.08477500	5.95247200	3.93049600
H	-4.32336200	4.49411600	0.53593200
H	-3.91523000	6.26763400	2.25848800
C	3.37739400	-1.31893800	1.11900200
C	3.15667700	-2.70234700	1.16845300
C	3.91841900	-0.68257800	2.24620400
C	3.44453800	-3.42754900	2.32723500
H	2.70324900	-3.18709900	0.29964600
C	4.20739800	-1.40488000	3.40661400
H	4.08645000	0.39638200	2.21044900

C	3.96528200	-2.78276300	3.45406800
H	3.23751600	-4.50117400	2.35770400
H	4.61619700	-0.88990300	4.28128100
H	4.17910400	-3.34891500	4.36572800
C	4.23707800	-0.36465900	-1.05243600
C	4.03015200	0.16074700	-2.33591700
C	5.54036700	-0.72361500	-0.66701400
C	5.11089700	0.35109700	-3.19943600
H	3.03712700	0.49593300	-2.68184800
C	6.61529200	-0.56069200	-1.54616100
H	5.72454800	-1.13932000	0.32511200
C	6.40281900	-0.01712500	-2.81847700
H	4.91009000	0.84461200	-4.15201900
H	7.62297800	-0.84988300	-1.22857900
H	7.24786300	0.13492400	-3.49886300
Cl	0.91122300	-2.81163700	-1.79503300
N	-0.19236900	-0.11249100	-3.52747000
C	2.13735800	2.76868600	-3.16054000
H	1.41649900	-0.34120800	-4.59189400
H	0.91131000	-1.72115200	-3.89537200
H	-1.07706900	0.12444300	-3.97689300
O	1.28580800	3.69449600	-2.90573200
O	1.69331700	1.56251000	-3.50365500
O	3.39188300	2.94218500	-3.04685900
H	0.41578600	0.80568400	-3.42585300

4

N	0.79338500	1.32215600	0.19278300
C	-0.06474000	1.15041900	-0.86392400
N	-0.01061200	2.37639700	-1.50766300
C	1.33844200	2.59036400	0.25500500
H	1.98552500	2.91180800	1.05983100
C	0.84438000	3.28232400	-0.81315100
C	1.00149600	4.62000100	-1.26938800
H	1.65018900	5.28675400	-0.69916100
C	0.33147900	5.02009700	-2.38871700
H	0.42785000	6.04197200	-2.76349400
C	-0.50508100	4.08577700	-3.08662000
H	-1.02662100	4.39160400	-3.99361600
C	-1.53615500	1.82618800	-3.39289100
C	-0.68130300	2.79553500	-2.66813800
C	-0.93576600	0.73020200	-4.04368700
C	-1.75282900	-0.29371500	-4.53188800
H	-1.28981500	-1.17499400	-4.98252800
C	-3.14188900	-0.26003300	-4.36757000
C	-3.71461300	0.90986800	-3.85788700
H	-4.80167100	0.96825800	-3.75317700
C	-2.94008200	1.96822900	-3.37907100
C	1.06756400	0.28006100	1.15150200

C	2.22265200	-0.50052400	0.99264600
C	2.41743600	-1.58575800	1.85513000
H	3.27650200	-2.23983400	1.69965700
C	1.48881900	-1.89855900	2.85177400
C	0.39119300	-1.04680500	3.03531700
H	-0.34354200	-1.29835800	3.79777700
C	0.16095700	0.04897000	2.20562000
C	3.22246400	-0.15173900	-0.10637000
H	2.62876100	0.03442700	-1.01477100
C	1.55533800	-3.17015200	3.64760700
H	0.62450500	-3.72874500	3.41630700
H	1.56387300	-2.97039900	4.73322900
H	2.44282000	-3.77032900	3.39212700
C	-1.04232800	0.96690300	2.39144800
C	0.56511000	0.65227000	-4.15943000
H	1.00575300	0.32743700	-3.20334500
C	-3.99698900	-1.47391600	-4.60411500
H	-3.45979000	-2.25406700	-5.16527000
H	-4.92754300	-1.22708100	-5.14360800
H	-4.27639000	-1.87892500	-3.60879300
Pd	-1.37323700	-0.41983600	-0.98443700
C	-0.09976400	-1.94889900	-1.34700200
C	0.35395700	-2.37257600	-2.60695900
C	0.97330100	-3.62091000	-2.79562500
C	1.17539300	-4.49572000	-1.72283600

C	0.73337100	-4.07467900	-0.45770700
C	0.11192300	-2.83965600	-0.27241700
H	0.18967200	-1.75241000	-3.48473300
H	1.29443900	-3.91599700	-3.80248900
H	0.82423000	-4.73466700	0.41090800
H	-0.29048500	-2.64189400	0.72326600
C	1.82088700	-5.84768100	-1.90666200
H	2.10711600	-6.01952800	-2.95791000
H	1.14190800	-6.66549900	-1.60688800
H	2.73155000	-5.95158400	-1.29018000
N	-4.07548500	-1.72835200	-1.19502100
H	-4.17590600	-0.70994200	-1.23825800
H	-4.57953300	-1.97758700	-0.33623400
H	-1.58254900	0.97293500	1.43255600
C	-3.61155100	3.20203400	-2.83221600
H	-4.67540600	3.00362800	-2.64140900
H	-3.53897500	4.04577700	-3.54283900
H	-3.16005300	3.50601400	-1.88021700
H	0.99930500	1.63238100	-4.41307600
H	0.86895400	-0.07332600	-4.92758300
C	3.97996300	1.13384900	0.19886300
C	4.29539100	1.50644100	1.51242000
C	4.37161100	1.97895400	-0.84808900
C	4.96477600	2.70530800	1.77398400
H	3.97517900	0.86321000	2.33453100

C	5.03737600	3.17821900	-0.59280600
H	4.11779400	1.70206400	-1.87456200
C	5.33364000	3.54988200	0.72308100
H	5.18515700	2.98770000	2.80712100
H	5.31076000	3.83509600	-1.42318900
H	5.84243100	4.49589700	0.92790100
C	4.17381000	-1.29212400	-0.44692900
C	5.47235200	-1.37714200	0.07406100
C	3.72043800	-2.30933200	-1.29700600
C	6.30096300	-2.45687500	-0.24803000
H	5.84106700	-0.59250900	0.73716800
C	4.54783400	-3.38380500	-1.62465300
H	2.70638100	-2.26485600	-1.69498900
C	5.84250400	-3.46362100	-1.10172000
H	7.31023100	-2.50858300	0.17120900
H	4.16506300	-4.16345700	-2.28608200
H	6.48938200	-4.30865700	-1.35506100
C	-0.61359400	2.40025600	2.64630400
C	-1.28743400	3.44645900	1.99882400
C	0.46589200	2.71235200	3.48566100
C	-0.87497800	4.76955700	2.16908200
H	-2.10792900	3.19110100	1.32209500
C	0.87814600	4.03640000	3.66070000
H	1.00230900	1.89949600	3.98104200
C	0.21165200	5.07173100	2.99702500

H	-1.39333400	5.56973300	1.63288900
H	1.73028400	4.26006700	4.30978500
H	0.54135000	6.10778800	3.12043800
C	-2.05272000	0.44200700	3.40038000
C	-2.81480700	-0.67946900	3.03806400
C	-2.23024300	1.00175000	4.67223000
C	-3.70725500	-1.25954600	3.94065100
H	-2.68854700	-1.13358800	2.05498600
C	-3.14468800	0.43995500	5.57125800
H	-1.65315100	1.88148100	4.96507900
C	-3.87500100	-0.69515600	5.21046200
H	-4.21431100	-2.18302900	3.65457400
H	-3.27429600	0.88782900	6.56236100
H	-4.56752100	-1.15235900	5.92411400
Cl	-3.13559500	1.26112600	-0.28916500
N	-2.68005000	-2.00468000	-0.90161600
H	-2.38259900	-2.66907300	-1.61727100
C	-2.45273600	-3.82552000	2.52367800
O	-1.21493500	-3.60057100	2.38247900
O	-3.04086600	-4.34459100	3.48366600
O	-3.28026400	-3.40726800	1.48081000
H	-2.77429200	-2.97977000	0.73899400

5-h

N	-0.68388800	0.72378800	0.85474500
C	0.60260800	0.22164300	0.87692900
N	0.96791500	0.26984800	2.19520400
C	-1.10743000	1.09839700	2.14232700
H	-2.03125800	1.63923000	2.30027600
C	-0.05105200	0.87489400	2.99307100
C	0.20923700	1.19559500	4.35126500
H	-0.56168100	1.71404000	4.92247500
C	1.46787100	0.89125200	4.87981600
H	1.70281000	1.15214700	5.91682200
C	2.40785800	0.18947700	4.11570100
H	3.32158700	-0.18025800	4.58166600
C	2.96845200	-1.23020600	2.12033300
C	2.15566800	-0.20809200	2.78879100
C	2.38814800	-2.49381800	1.77352000
C	3.18379100	-3.48738800	1.20631300
H	2.72300300	-4.45050400	0.96123300
C	4.54704800	-3.29632200	0.93770300
C	5.11852600	-2.08157400	1.34475800
H	6.18748900	-1.90992400	1.17067600
C	4.37752200	-1.07046900	1.95958900
C	-1.50631800	0.81763200	-0.29105400
C	-2.66272300	0.00829800	-0.38624900
C	-3.47977600	0.13855400	-1.51667800
H	-4.34223600	-0.52496400	-1.62008700

C	-3.19261600	1.04912400	-2.53586100
C	-2.04491900	1.86514400	-2.40367900
H	-1.81210300	2.59795900	-3.17946100
C	-1.20389500	1.76042500	-1.30038100
C	-2.93843900	-1.02552000	0.69850700
H	-1.96066700	-1.26797400	1.13328700
C	-4.06172600	1.16040700	-3.76126400
H	-3.51288300	0.87066900	-4.67630400
H	-4.41392900	2.19649200	-3.92238300
H	-4.94804800	0.51076400	-3.68632200
C	-0.02231100	2.69202900	-1.07094500
C	0.93238200	-2.75918800	2.02547300
H	0.31069900	-2.21377600	1.29383500
C	5.35979100	-4.32590100	0.19903400
H	4.85112400	-5.30389400	0.17846300
H	6.35837100	-4.47336300	0.65018700
H	5.52799000	-4.02775700	-0.85460600
Pd	1.88224300	-0.24980700	-0.64609200
C	0.73698800	-1.65836600	-1.52234000
C	1.06509900	-3.02162800	-1.44362600
C	0.40947200	-3.98552300	-2.22204500
C	-0.59669000	-3.62812700	-3.12907700
C	-0.92288300	-2.26800600	-3.21987900
C	-0.27281200	-1.30881500	-2.43763200
H	1.87132800	-3.33641600	-0.78312400

H	0.70094600	-5.03928600	-2.13087100
H	-1.70775800	-1.94451800	-3.91296400
H	-0.56012700	-0.26766500	-2.56487900
C	-1.31782100	-4.65434500	-3.96822700
H	-0.88922800	-5.66046400	-3.82811900
H	-1.26288700	-4.41473000	-5.04534000
H	-2.38924000	-4.71069600	-3.70553800
N	3.25471300	-0.84644900	-2.01472900
N	4.44554200	-0.48769200	-1.71016900
H	4.62628500	0.19028300	-0.94786000
H	5.24135200	-0.84477000	-2.24996500
H	0.81776900	2.06129000	-0.74870900
C	5.08065200	0.18228500	2.41625000
H	6.06827100	0.26856300	1.93427400
H	5.23953100	0.19152300	3.50980700
H	4.48811700	1.06919400	2.15458000
H	0.62710200	-2.40394600	3.02427500
H	0.69832800	-3.83043100	1.93413300
C	-3.78227200	-0.47711700	1.84389200
C	-4.87754400	0.36652600	1.61857600
C	-3.45054700	-0.81696500	3.16221000
C	-5.63310300	0.86019000	2.69807800
H	-5.11472100	0.67075900	0.59727200
C	-4.19489100	-0.32347000	4.24205400
H	-2.58265800	-1.45511900	3.34176500

C	-5.29011400	0.51948000	4.00660500
H	-6.48498500	1.52009900	2.50765000
H	-3.89853000	-0.57149700	5.26485200
H	-5.86762700	0.91707000	4.84731100
C	-3.47749300	-2.33742400	0.14469700
C	-4.84915700	-2.64216000	0.10024500
C	-2.56693500	-3.28706300	-0.34923700
C	-5.29636100	-3.86013700	-0.41972800
H	-5.56938600	-1.92244300	0.49430800
C	-3.01146000	-4.50691100	-0.85664100
H	-1.49850400	-3.06198900	-0.34529000
C	-4.38109900	-4.80278600	-0.89850300
H	-6.36946800	-4.07595600	-0.44315000
H	-2.27612400	-5.22368500	-1.22742200
H	-4.72905600	-5.75782100	-1.30342000
C	-0.31844400	3.62556100	0.09370800
C	0.65080900	3.81576800	1.09136900
C	-1.56647600	4.24845500	0.23924800
C	0.37418200	4.61372600	2.20396400
H	1.60780200	3.29434100	1.00335300
C	-1.84055100	5.05406900	1.34799300
H	-2.34158200	4.06751900	-0.50953600
C	-0.86781600	5.24036700	2.33671600
H	1.12898500	4.71795900	2.98789400
H	-2.82487700	5.52145200	1.45023600

H	-1.08704000	5.85267100	3.21683700
C	0.46375000	3.35285200	-2.34609100
C	1.27084300	2.59241500	-3.21014900
C	0.12491700	4.65964900	-2.71648900
C	1.71259600	3.12617700	-4.42146500
H	1.56508500	1.58111300	-2.90957300
C	0.57057100	5.19761400	-3.93115600
H	-0.48927500	5.26177600	-2.04377600
C	1.36269300	4.43177100	-4.78916400
H	2.33990800	2.51797100	-5.07937800
H	0.29775800	6.22213500	-4.20359800
H	1.71298500	4.85094600	-5.73773300
Cl	3.32372300	1.73739900	-0.00825100

5

N	-0.04365400	-0.97922200	0.94960400
C	-0.53589900	0.29725800	0.94070800
N	-1.01111700	0.45253300	2.22359600
C	-0.26297000	-1.64448300	2.14423800
H	0.02957000	-2.67475200	2.30333400
C	-0.88039500	-0.75016400	2.97420700
C	-1.36202900	-0.80842900	4.31090900
H	-1.25444100	-1.74257500	4.86382900
C	-1.93935000	0.30390100	4.85182200

H	-2.31782200	0.29268000	5.87627500
C	-2.03873100	1.50823300	4.07841900
H	-2.46820900	2.40511900	4.52381400
C	-1.63982300	2.86199000	2.01817500
C	-1.59064100	1.59164200	2.78911300
C	-0.43934300	3.53141400	1.69607900
C	-0.50408300	4.74157400	0.99702800
H	0.42722600	5.26307700	0.75972000
C	-1.71973400	5.28469700	0.57454600
C	-2.89681300	4.62409800	0.94121300
H	-3.86095400	5.04299700	0.63889500
C	-2.88724700	3.43815700	1.68300000
C	0.66781500	-1.55149800	-0.15867900
C	2.06343500	-1.39459900	-0.21206600
C	2.75587700	-1.97022900	-1.27945900
H	3.83606700	-1.83180400	-1.34607100
C	2.08646700	-2.67732300	-2.28528600
C	0.69463400	-2.80149300	-2.20899100
H	0.15402100	-3.32277800	-3.00095000
C	-0.03700100	-2.24393100	-1.15436700
C	2.76387000	-0.57833100	0.86942700
H	2.14875100	0.32313100	0.99754900
C	2.84509500	-3.22356700	-3.46806300
H	2.96984700	-2.44360900	-4.23970300
H	2.31386000	-4.06582800	-3.93657400

H	3.85330700	-3.56210500	-3.18242400
C	-1.54978700	-2.36223800	-1.05306000
C	0.90630300	2.96168700	2.05696300
H	1.21350900	2.23788000	1.28513100
C	-1.76232000	6.51874700	-0.28912600
H	-0.85595800	7.13166600	-0.16713400
H	-2.63911200	7.14573200	-0.06211800
H	-1.82937000	6.23814200	-1.35499100
Pd	-1.09871200	1.23900600	-0.79963400
C	0.66167800	1.74089200	-1.64846400
C	1.29092200	2.99211200	-1.51107900
C	2.42851000	3.34281700	-2.25415000
C	3.00475500	2.45083300	-3.16646900
C	2.39100200	1.19604300	-3.30931900
C	1.25024400	0.85664300	-2.57967000
H	0.87070300	3.72984400	-0.82687500
H	2.87591100	4.33456500	-2.11885100
H	2.81327500	0.46936400	-4.01305900
H	0.79532500	-0.11976000	-2.75276000
C	4.23453200	2.80699900	-3.96473500
H	4.58900000	3.82223000	-3.72527500
H	4.04166600	2.76994100	-5.05133900
H	5.06470100	2.10746700	-3.76365300
N	-1.91160700	1.86322900	-2.55172500
N	-2.97551200	2.80371300	-2.35530000

H	-2.71364600	3.50165100	-1.64480500
H	-3.71741800	2.26824700	-1.89800000
H	-1.92196500	-1.38388000	-0.71708400
H	-1.22411700	2.33981600	-3.13661200
C	-4.19587900	2.81567900	2.09652800
H	-5.02387600	3.25313800	1.51992200
H	-4.40316300	2.98852500	3.16640200
H	-4.18779400	1.73476200	1.90209200
H	0.88651100	2.43641100	3.02378200
H	1.67143300	3.75026500	2.10111000
C	2.77785100	-1.28911200	2.21453000
C	2.92452600	-2.67874400	2.31315000
C	2.62851300	-0.54432200	3.39150900
C	2.90455200	-3.31160400	3.55905400
H	3.01994200	-3.27073400	1.40028100
C	2.60156300	-1.17173600	4.63715500
H	2.49899100	0.53814100	3.32196900
C	2.73685700	-2.56088200	4.72587600
H	3.00740000	-4.39847200	3.61704600
H	2.45679400	-0.57555100	5.54160200
H	2.70581800	-3.05615800	5.69979800
C	4.14399700	-0.08734400	0.45598800
C	5.32051600	-0.77931000	0.77383900
C	4.23538700	1.09375700	-0.29297600
C	6.56378600	-0.30325900	0.34610700

H	5.26590100	-1.69433400	1.36662800
C	5.47605500	1.57396800	-0.71447000
H	3.32625800	1.63628000	-0.55458700
C	6.64614300	0.87652300	-0.39861800
H	7.47235600	-0.85536000	0.60204800
H	5.51976100	2.49727500	-1.29571100
H	7.61840400	1.25196500	-0.72917500
C	-1.95281700	-3.35593600	0.02584000
C	-3.04136300	-3.05005100	0.85724600
C	-1.24515600	-4.54536600	0.24906600
C	-3.40751100	-3.91532900	1.88949100
H	-3.56106300	-2.09944100	0.70582200
C	-1.61302500	-5.41383900	1.28181600
H	-0.38164100	-4.78098900	-0.37754100
C	-2.69589900	-5.10019200	2.10792500
H	-4.24675800	-3.65341300	2.53969400
H	-1.04656500	-6.33513600	1.44544200
H	-2.97984400	-5.77287600	2.92213800
C	-2.21253800	-2.58517900	-2.40348100
C	-2.45285300	-1.46330100	-3.21614300
C	-2.55966800	-3.85774000	-2.87506800
C	-3.01592000	-1.62883000	-4.48286500
H	-2.21624800	-0.44931500	-2.86718400
C	-3.12390500	-4.01831800	-4.14554100
H	-2.39465200	-4.73147200	-2.24048100

C	-3.35076300	-2.90269500	-4.95543300
H	-3.19867800	-0.74250200	-5.09619600
H	-3.39118700	-5.01861900	-4.49890700
H	-3.79511800	-3.02508500	-5.94759400
Cl	-3.42536700	0.41637900	-0.09406300

6

N	-0.89109800	0.03677300	1.03969500
C	0.38479500	-0.47015500	0.94569400
N	0.61660700	-0.92153300	2.23155500
C	-1.42819000	-0.03977600	2.31574400
H	-2.41470900	0.33053800	2.56384200
C	-0.48728600	-0.65159100	3.09386200
C	-0.43428500	-1.05618000	4.45723300
H	-1.28587200	-0.83235000	5.10141400
C	0.67045300	-1.72057300	4.90583800
H	0.73606200	-2.05385000	5.94402500
C	1.76070800	-1.98935800	4.00975700
H	2.64070300	-2.52560200	4.36529200
C	2.88274100	-1.87166800	1.78311100
C	1.74585900	-1.58823400	2.70169300
C	2.75742700	-2.85327600	0.77961600
C	3.85966100	-3.13236200	-0.03887300
H	3.75148700	-3.87783300	-0.83230000

C	5.07635800	-2.46345200	0.11425500
C	5.18614900	-1.51820100	1.14157800
H	6.12438200	-0.96974300	1.26114000
C	4.11462200	-1.20303900	1.98046900
C	-1.61885300	0.45436700	-0.12467200
C	-2.32627500	-0.52411500	-0.84428000
C	-3.02966700	-0.13645800	-1.98806300
H	-3.57309500	-0.89162700	-2.55857400
C	-3.01941000	1.19038200	-2.43054100
C	-2.30743100	2.14102700	-1.68978800
H	-2.27757300	3.17435200	-2.03706200
C	-1.60608300	1.80096800	-0.52959500
C	-2.28556500	-1.97227600	-0.36675100
H	-1.23257800	-2.16383000	-0.11415200
C	-3.70015800	1.57885400	-3.71779100
H	-2.98244400	1.54362900	-4.55598300
H	-4.10000400	2.60375000	-3.67265500
H	-4.52489800	0.89273900	-3.96480700
C	-0.81531900	2.81379300	0.28593100
C	1.45226900	-3.55992200	0.53300600
H	0.80492100	-2.90184400	-0.07030600
C	6.22764400	-2.68186400	-0.83367700
H	6.02477000	-3.50013100	-1.54095700
H	7.16102700	-2.91483100	-0.29449900
H	6.40714500	-1.75966200	-1.41273400

Pd	1.63332300	0.13716900	-0.57384600
C	2.56361300	0.62118400	-2.43840500
C	2.22570400	-0.79483800	-2.47702500
C	1.17235500	-1.24362200	-3.32763100
C	0.32561000	-0.36463600	-3.98398700
C	0.55133500	1.02993800	-3.80790300
C	1.63507700	1.50025000	-3.09231500
H	2.98485200	-1.53090800	-2.19270100
H	1.06393800	-2.32145800	-3.49239000
H	-0.12967900	1.74488500	-4.28046900
H	1.84879200	2.56959700	-3.04495800
C	-0.81507400	-0.84608300	-4.84269700
H	-0.67202800	-1.89059000	-5.16025000
H	-0.92752100	-0.22920100	-5.75088000
H	-1.77672300	-0.80542700	-4.30200100
N	3.90511400	1.12499700	-2.29375800
N	4.69479500	0.62172800	-1.22603900
H	4.42876700	-0.34633100	-1.02414400
H	4.39098100	1.13527500	-0.39158600
H	0.18459900	2.37555400	0.43211100
H	4.42121400	0.99454000	-3.16267500
C	4.26835300	-0.11143700	3.00638600
H	5.30971100	0.24122100	3.04039100
H	3.97817000	-0.43717400	4.01562300
H	3.62979900	0.74343900	2.72048100

H	0.92055300	-3.78329800	1.47088300
H	1.60245700	-4.50033300	-0.01730700
C	-3.08164000	-2.18869800	0.91068500
C	-4.27462800	-1.49970900	1.16306200
C	-2.60923900	-3.08980700	1.87373700
C	-4.97052100	-1.69377500	2.35960500
H	-4.64050600	-0.78073200	0.42688100
C	-3.29745500	-3.28400700	3.07125200
H	-1.66880100	-3.61564500	1.69269400
C	-4.48172800	-2.58295600	3.32068900
H	-5.89295200	-1.13763000	2.54696400
H	-2.89647100	-3.96980700	3.82171900
H	-5.01725500	-2.72420200	4.26309400
C	-2.64122200	-2.97109700	-1.45842000
C	-3.89556900	-3.58676600	-1.55242000
C	-1.67015500	-3.26973400	-2.42501600
C	-4.17489700	-4.47827200	-2.59389200
H	-4.65910900	-3.37180000	-0.80273900
C	-1.94524200	-4.15958500	-3.46284800
H	-0.69950400	-2.77420000	-2.36591400
C	-3.20137700	-4.76894200	-3.55232900
H	-5.15970700	-4.94986500	-2.65259400
H	-1.17483300	-4.37449500	-4.20780200
H	-3.41900900	-5.46724700	-4.36482200
C	-1.39836200	3.00560700	1.67783400

C	-0.52056500	3.12715100	2.76544400
C	-2.77969600	3.03444300	1.91291800
C	-1.01980700	3.26300700	4.06174500
H	0.55559900	3.06584400	2.57448000
C	-3.28000500	3.17418900	3.21116300
H	-3.46920000	2.91008100	1.07408500
C	-2.40002100	3.28562100	4.29155700
H	-0.32443800	3.33445900	4.90250400
H	-4.36065200	3.18587400	3.37995100
H	-2.78831700	3.38330400	5.30923600
C	-0.58084400	4.13480300	-0.43050800
C	0.65085100	4.34633100	-1.06737600
C	-1.55406300	5.14298600	-0.48957500
C	0.89071400	5.53047400	-1.76899200
H	1.42535500	3.58213600	-0.97567700
C	-1.31566400	6.32675100	-1.19336000
H	-2.50706900	5.00336200	0.02546400
C	-0.09211700	6.52211400	-1.84129500
H	1.85834100	5.67848800	-2.25591200
H	-2.08700900	7.10142500	-1.23115800
H	0.09751700	7.44870000	-2.39057900
Cl	2.51980800	2.12166200	0.97070900

N	-0.86566400	0.37390000	1.10679300
C	0.12446000	-0.56829900	1.03422600
N	0.11604400	-1.11318800	2.29942300
C	-1.49018300	0.43058700	2.33900100
H	-2.28789300	1.12817600	2.56120900
C	-0.86688800	-0.50230600	3.12683400
C	-0.98997100	-0.91534800	4.48385300
H	-1.73167000	-0.42510000	5.11560400
C	-0.17376700	-1.90505100	4.95100100
H	-0.24494600	-2.23828400	5.98811200
C	0.77945100	-2.53032700	4.07602000
H	1.40676500	-3.34394300	4.44112000
C	1.86217100	-2.81883300	1.83259700
C	0.91954600	-2.15177300	2.77159600
C	1.37948000	-3.80127900	0.94023800
C	2.28786300	-4.44751000	0.09844300
H	1.91385500	-5.19884100	-0.60303000
C	3.65790600	-4.15357900	0.12405300
C	4.10928200	-3.18148600	1.02224300
H	5.17334700	-2.93058600	1.05346900
C	3.23120900	-2.49759700	1.87313800
C	-1.23287400	1.13244100	-0.05293500
C	-2.31943900	0.68057500	-0.81399500
C	-2.64451500	1.37323600	-1.98369700
H	-3.48018600	1.02162700	-2.59253000

C	-1.89342800	2.47495300	-2.40709100
C	-0.80383000	2.88906000	-1.62809000
H	-0.19509200	3.72496800	-1.97264000
C	-0.45455800	2.23868000	-0.44245200
C	-3.08170600	-0.55774400	-0.36555300
H	-2.36647900	-1.16388000	0.20549900
C	-2.20491300	3.15548000	-3.71475700
H	-1.75334500	2.59859900	-4.55410300
H	-1.80570000	4.17991300	-3.74527600
H	-3.28867400	3.19904500	-3.90213300
C	0.79015400	2.58648600	0.36769600
C	-0.09133800	-4.11371900	0.86830800
H	-0.63448400	-3.25831700	0.43373300
C	4.61488500	-4.87951800	-0.78783100
H	4.22593300	-4.93288300	-1.81714100
H	4.77616800	-5.91770800	-0.45028400
H	5.59758100	-4.38589300	-0.81941600
Pd	1.18043100	-0.90885000	-0.64028500
C	3.05700400	0.09729600	-2.44556000
C	2.41472400	-1.17705700	-2.44650800
C	1.18646000	-1.32950800	-3.13615400
C	0.59828100	-0.26629100	-3.85031000
C	1.27797500	0.95865500	-3.86554700
C	2.47165900	1.15020000	-3.16977400
H	2.95838500	-2.05811000	-2.09519700

H	0.73948700	-2.32494400	-3.19738100
H	0.84975300	1.79940100	-4.41785400
H	2.95689800	2.12848300	-3.17840800
C	-0.71869600	-0.43376300	-4.56019300
H	-0.84646700	-1.45471900	-4.95039700
H	-0.80879900	0.26730600	-5.40428300
H	-1.56343700	-0.24738800	-3.87797000
N	4.26842300	0.27064700	-1.78541400
N	4.51250500	-0.43663800	-0.59776500
H	4.87398500	-1.36193200	-0.82655300
H	3.60833300	-0.60249500	-0.11953700
H	1.41115400	1.67661800	0.30234200
H	4.56538600	1.23827600	-1.71704100
C	3.74201300	-1.40637400	2.78000200
H	4.74872500	-1.08472100	2.47927100
H	3.78047000	-1.73264800	3.83212900
H	3.08065400	-0.52751200	2.74177400
H	-0.51660800	-4.30061400	1.86646900
H	-0.28422200	-4.99248000	0.23709200
C	-4.22287200	-0.24364600	0.59251000
C	-4.85908100	1.00300400	0.62814800
C	-4.65386800	-1.24208400	1.47979600
C	-5.90143000	1.24720500	1.52965400
H	-4.53035500	1.79434600	-0.04765000
C	-5.69141600	-1.00263400	2.37989200

H	-4.16046600	-2.21748000	1.46303300
C	-6.32046000	0.24727000	2.40889800
H	-6.38477000	2.22734900	1.54452400
H	-6.00846400	-1.79167300	3.06637400
H	-7.13137400	0.43906800	3.11566400
C	-3.49023700	-1.43378000	-1.54195100
C	-4.78874500	-1.44939300	-2.06384700
C	-2.50809500	-2.24162100	-2.13566300
C	-5.10074800	-2.25652000	-3.16315200
H	-5.56284400	-0.83318500	-1.60174300
C	-2.82078500	-3.05337800	-3.22585800
H	-1.48721500	-2.20991200	-1.74182800
C	-4.11954600	-3.06221300	-3.74563600
H	-6.11846000	-2.25935700	-3.56156200
H	-2.04622200	-3.67956100	-3.67576300
H	-4.36585700	-3.69708200	-4.60019700
C	0.49830100	2.80777000	1.84609000
C	1.34297500	2.23946300	2.80753700
C	-0.60006800	3.56270900	2.27754200
C	1.08857400	2.40383700	4.17008600
H	2.19566700	1.64132000	2.47743800
C	-0.85201500	3.74027400	3.64008400
H	-1.28332900	3.98908100	1.53923300
C	-0.01150700	3.15613600	4.59198000
H	1.74677200	1.93464100	4.90533100

H	-1.71570200	4.32880500	3.96015700
H	-0.21450600	3.28393900	5.65797200
C	1.63507100	3.72289400	-0.18212900
C	2.94440400	3.45804000	-0.60104800
C	1.17006400	5.04539200	-0.24267000
C	3.76718800	4.47811300	-1.08665500
H	3.31502100	2.43400600	-0.53825400
C	1.98495500	6.06737300	-0.73285900
H	0.16053100	5.28034900	0.09971300
C	3.28706100	5.78735300	-1.16034100
H	4.78764400	4.24988900	-1.40560400
H	1.60370900	7.09066600	-0.77630700
H	3.92552900	6.58829500	-1.54061600

8

N	-1.96667200	-0.39430500	-0.47798700
C	-0.85883000	-1.19579500	-0.40119100
N	-1.26485300	-2.36919800	-0.99571600
C	-3.05280700	-1.03768100	-1.05289900
H	-4.01826700	-0.56406800	-1.16566300
C	-2.63437900	-2.29561700	-1.38332000
C	-3.28473400	-3.41033100	-1.97724500
H	-4.33407200	-3.30956000	-2.25575000
C	-2.56302000	-4.56420300	-2.17021100

H	-3.02727200	-5.44288100	-2.62490000
C	-1.18768200	-4.60517900	-1.80832200
H	-0.59803100	-5.49833200	-2.01413200
C	0.93296800	-3.60860200	-0.97418800
C	-0.52039400	-3.54102300	-1.24264100
C	1.83608700	-2.85016300	-1.76082000
C	3.21208200	-2.98551300	-1.56020400
H	3.89919500	-2.34109700	-2.12816300
C	3.72407600	-3.87249700	-0.59879800
C	2.81442800	-4.64759700	0.13367700
H	3.19616600	-5.34228600	0.88947100
C	1.42872900	-4.54030500	-0.03441900
C	-2.00659000	0.98340300	-0.08697600
C	-1.79758600	1.96407200	-1.07270200
C	-1.84552300	3.30745500	-0.69157700
H	-1.64395000	4.07537500	-1.43951000
C	-2.07379600	3.67973000	0.63611300
C	-2.28574000	2.67867500	1.59285300
H	-2.44650800	2.96260200	2.63318000
C	-2.26727700	1.32230600	1.25329100
C	-1.47155800	1.53553700	-2.50163300
H	-0.74383200	0.71851000	-2.39949800
C	-2.01047000	5.12920400	1.04612500
H	-0.97385600	5.40948300	1.30286700
H	-2.63338400	5.32899500	1.93202200

H	-2.33607700	5.79556300	0.23215000
C	-2.49921000	0.20397700	2.26384500
C	1.33387300	-1.92294000	-2.84045000
H	0.79188800	-1.06963400	-2.40475600
C	5.19571500	-3.91020300	-0.30631000
H	5.48340400	-4.83231100	0.22640800
H	5.46871900	-3.02072600	0.29292600
H	5.80516400	-3.78156800	-1.21226100
Pd	0.75295100	-0.87048000	0.74954300
C	1.49610800	0.71245500	-0.24190000
C	2.61339800	0.59041400	-1.07146500
C	3.35177700	1.71770100	-1.46068500
C	2.98674100	3.00055100	-1.04401000
C	1.85044100	3.12615800	-0.22784100
C	1.12344500	2.00392500	0.17283900
H	3.03675000	-0.35994000	-1.39087600
H	4.24417700	1.50683700	-2.05914900
H	1.54368700	4.11640700	0.12896600
H	0.29350900	2.14355400	0.86412700
C	3.80647800	4.21378400	-1.40735400
H	4.60139100	3.95336700	-2.12290600
H	4.29337000	4.64846300	-0.51526100
H	3.18606700	5.00982900	-1.85705500
N	3.50832800	-1.66173300	1.77959100
H	4.05886900	-1.31791300	0.94904500

H	3.01465900	-2.48881700	1.44051500
H	-1.65950300	-0.49538400	2.14529600
C	0.50760600	-5.39900700	0.79808900
H	1.05685400	-5.83173000	1.64786700
H	0.07716000	-6.23345600	0.21677600
H	-0.32049900	-4.80400600	1.20672000
H	0.64099100	-2.44437500	-3.52447800
H	2.17661600	-1.51510500	-3.41378200
C	-2.68031800	0.96556900	-3.22975000
C	-3.96278000	1.50851000	-3.07167400
C	-2.51623300	-0.13892900	-4.07561600
C	-5.06104700	0.94439400	-3.72903800
H	-4.10512400	2.35224400	-2.39269300
C	-3.60939500	-0.70841400	-4.72947600
H	-1.52297600	-0.58136200	-4.18367700
C	-4.88949500	-0.16876300	-4.55576400
H	-6.05811300	1.36983200	-3.58313300
H	-3.46435400	-1.59083500	-5.35743800
H	-5.75011000	-0.62035500	-5.05693500
C	-0.77655700	2.61388300	-3.32283600
C	-1.48421400	3.57642600	-4.05865400
C	0.62311600	2.65208900	-3.32958300
C	-0.80473300	4.56476200	-4.77592600
H	-2.57554200	3.54789900	-4.07933000
C	1.30381400	3.63445900	-4.05173400

H	1.18887500	1.91234500	-2.76218400
C	0.59384400	4.59662800	-4.77462300
H	-1.37127600	5.30863800	-5.34408100
H	2.39547500	3.63877300	-4.03464300
H	1.12727700	5.36705700	-5.33879700
C	-3.75883200	-0.59138500	1.94533200
C	-3.72298600	-1.98940900	2.04972000
C	-4.94046600	0.02811100	1.51697100
C	-4.84570300	-2.74984800	1.71902300
H	-2.78495500	-2.46852700	2.34611900
C	-6.06744200	-0.73266800	1.19344200
H	-4.96315600	1.11385600	1.39416800
C	-6.02217200	-2.12666900	1.29013800
H	-4.79188500	-3.84028000	1.77383200
H	-6.97857400	-0.23551700	0.84787200
H	-6.89635500	-2.72563300	1.01947400
C	-2.46201000	0.64549800	3.71849800
C	-1.35065400	0.30253700	4.50179700
C	-3.50250200	1.37589800	4.31307000
C	-1.26440700	0.71170500	5.83532600
H	-0.57497400	-0.32541700	4.05716900
C	-3.41832000	1.78637000	5.64567400
H	-4.39083700	1.62177600	3.72752900
C	-2.29313900	1.46224300	6.41085600
H	-0.38644300	0.43659200	6.42570100

H	-4.23710600	2.36009900	6.08982200
H	-2.22477400	1.78477200	7.45369800
C	2.92352700	0.60071800	2.39575000
C	4.06990800	1.13183400	1.79308600
C	2.15133100	1.39059800	3.25611800
C	4.39161800	2.46752600	2.03754600
H	4.67895800	0.52796400	1.10076600
C	2.49569700	2.72078900	3.48823300
H	1.25185700	0.97140400	3.71167100
C	3.61595800	3.28998800	2.86632800
H	5.26975200	2.88622900	1.53622300
H	1.86762500	3.32975500	4.14669800
C	3.93551000	4.75595200	3.02235600
H	3.52647500	5.16671400	3.96037500
H	3.50509600	5.34557500	2.19161000
H	5.02267600	4.93894400	3.01495600
Cl	-0.11349100	-2.46322400	2.52519900
C	5.75932100	-0.97429100	-1.37901800
O	5.02345500	-0.67029600	-2.39845700
O	5.25043000	-0.82497800	-0.17326800
O	6.92303100	-1.44595200	-1.51566500
N	2.45821100	-0.71792800	2.08932900
H	1.97471600	-1.09232900	2.90881500

9

N	1.60612300	-1.18138100	-0.34050000
C	0.48800900	-0.88604500	-1.07376100
N	0.57069400	-1.75990100	-2.13759200
C	2.40204300	-2.15156500	-0.92781300
H	3.33692100	-2.48421500	-0.49765700
C	1.76805600	-2.52933800	-2.07578400
C	2.08209300	-3.45302300	-3.11051000
H	3.01148100	-4.01928400	-3.03628100
C	1.21138300	-3.59051000	-4.15196400
H	1.41967800	-4.29081300	-4.96455200
C	-0.00303700	-2.82933700	-4.17247300
H	-0.72325300	-2.97499600	-4.97710000
C	-1.64806500	-1.23887300	-3.20759100
C	-0.33343500	-1.93106000	-3.19298000
C	-2.65528200	-1.64483900	-2.30139200
C	-3.93493900	-1.09031200	-2.40334800
H	-4.74124200	-1.40473600	-1.73287700
C	-4.23389000	-0.12220800	-3.37104500
C	-3.22618400	0.24846400	-4.27001600
H	-3.44610200	0.99539900	-5.03977700
C	-1.94202500	-0.30639000	-4.22744500
C	1.88210500	-0.59139400	0.94030100
C	1.35978800	-1.21833700	2.08561400
C	1.59867400	-0.63146300	3.33066500

H	1.16281300	-1.08528800	4.22128400
C	2.31755100	0.56204800	3.44451600
C	2.83854600	1.15199100	2.28599100
H	3.38784300	2.09043800	2.36598600
C	2.64765700	0.58731100	1.02091700
C	0.51409500	-2.47751300	1.92297900
H	-0.20277200	-2.24294200	1.12430400
C	2.45929300	1.24788900	4.77935600
H	1.63683200	1.96967300	4.92562800
H	3.40322800	1.81086600	4.84939100
H	2.41715800	0.52859600	5.61190200
C	3.21744300	1.19557900	-0.25541900
C	-2.37293800	-2.66534100	-1.22887400
H	-1.77597000	-2.20813100	-0.42391600
C	-5.59996200	0.50886100	-3.41124500
H	-5.90574500	0.73891100	-4.44652100
H	-5.59415700	1.44852400	-2.83446200
H	-6.33600500	-0.13669900	-2.90914500
Pd	-0.58932200	0.80821700	-0.89794800
C	-1.61329500	0.47309200	0.80585300
C	-2.94776500	0.03852100	0.82857200
C	-3.70949800	0.03717300	2.00880600
C	-3.15399100	0.47002100	3.21928400
C	-1.81536500	0.89034000	3.20993900
C	-1.06493800	0.89372400	2.03271400

H	-3.44700800	-0.26255500	-0.09144800
H	-4.75021900	-0.30647800	1.97167300
H	-1.35019500	1.23848800	4.13986300
H	-0.04587800	1.27412100	2.07190000
C	-3.96767400	0.48511800	4.49041600
H	-4.99530200	0.13893400	4.30106300
H	-4.02699500	1.50033600	4.92184000
H	-3.52500000	-0.16597400	5.26597500
N	-2.94162200	2.53501300	-1.43919000
H	-3.54850200	1.91988800	-0.88242900
H	-2.86400500	2.08637900	-2.35179200
H	2.38034900	1.26338500	-0.96838100
C	-0.91667300	0.10045600	-5.25615400
H	-1.27492200	0.97148200	-5.82519700
H	-0.71282500	-0.71061000	-5.97632100
H	0.02886700	0.38387300	-4.77080100
H	-1.80723200	-3.52729200	-1.62067200
H	-3.30871400	-3.02675800	-0.78154100
C	1.33287900	-3.67035500	1.44834800
C	2.65037100	-3.88037800	1.87591500
C	0.76225300	-4.58668800	0.55519200
C	3.38826300	-4.97054000	1.40670000
H	3.11243500	-3.15706200	2.55153400
C	1.49684500	-5.67251500	0.07909600
H	-0.25815900	-4.41960300	0.20344200

C	2.81614600	-5.86813200	0.50102700
H	4.42072100	-5.11044300	1.73917000
H	1.04294100	-6.35825700	-0.64084200
H	3.39718000	-6.71236000	0.11990800
C	-0.32506000	-2.84482800	3.13879700
C	0.20373500	-3.55362200	4.22837900
C	-1.67446200	-2.47404800	3.16493300
C	-0.59921600	-3.87487700	5.32537700
H	1.25012200	-3.86616500	4.21415200
C	-2.48304600	-2.80595000	4.25460000
H	-2.09811900	-1.91458400	2.33020400
C	-1.94830200	-3.50377900	5.34009400
H	-0.17141100	-4.42562700	6.16833700
H	-3.53267900	-2.50583000	4.24450400
H	-2.58051100	-3.76134600	6.19455000
C	4.24929400	0.28408600	-0.90534900
C	4.23804800	0.13812800	-2.30056300
C	5.19067200	-0.43436600	-0.15574200
C	5.14506400	-0.71706800	-2.92795700
H	3.46568600	0.66298300	-2.87116600
C	6.10155900	-1.28908000	-0.78387400
H	5.18178200	-0.35368100	0.93406500
C	6.08002500	-1.43537100	-2.17409500
H	5.10589600	-0.84130500	-4.01361800
H	6.82214600	-1.85248000	-0.18372000

H	6.78257300	-2.11361000	-2.66697300
C	3.74858600	2.61476000	-0.10687000
C	2.99768900	3.67812300	-0.62678600
C	4.96673300	2.89756700	0.53196000
C	3.43426600	4.99693400	-0.47158700
H	2.07008900	3.46191900	-1.16017200
C	5.40773300	4.21431900	0.68075000
H	5.57886300	2.07789100	0.91473600
C	4.63592100	5.27126300	0.18520500
H	2.81604500	5.80990400	-0.85930300
H	6.35669100	4.41626800	1.18669900
H	4.97358800	6.30431300	0.30962500
C	-1.36249700	3.63609000	-0.09702500
C	-2.23167200	4.75395300	0.07917700
C	-0.11408600	3.70388400	0.59166000
C	-1.85607300	5.84027400	0.86371600
H	-3.19164400	4.73224800	-0.43553700
C	0.23543000	4.79854000	1.36826100
H	0.57140000	2.86388900	0.48277700
C	-0.62080400	5.90419100	1.53218200
H	-2.55567300	6.68042600	0.96394600
H	1.21459900	4.79737900	1.86153200
C	-0.24804200	7.06735700	2.41692600
H	0.82749000	7.30988400	2.34395600
H	-0.45337900	6.87759100	3.49060200

H	-0.80998500	7.97785900	2.14514000
Cl	0.91473300	1.63135200	-2.79785500
N	-1.64714000	2.56855000	-0.87771100
C	-6.59867300	-0.61497100	0.02486900
O	-5.98459300	0.63138100	-0.30367400
H	-5.83491400	1.05897800	0.55058700
O	-6.81192100	-1.34316900	-0.96004900
O	-6.81211900	-0.78999700	1.23965800

TS1

N	-0.43697700	0.88621600	0.95911300
C	0.59585200	0.00449200	1.10531900
N	0.85874300	0.05846800	2.45484800
C	-0.80374300	1.50366800	2.14263000
H	-1.58733700	2.24823600	2.19639800
C	0.01700800	0.99354200	3.11431700
C	0.17469600	1.21072900	4.51238800
H	-0.46631500	1.94313900	5.00449300
C	1.12084100	0.49567600	5.18912700
H	1.26343600	0.64202200	6.26148900
C	1.92751500	-0.47286000	4.49942300
H	2.65656000	-1.06913400	5.04878400
C	2.57637700	-1.72711100	2.41612500
C	1.80038100	-0.69804900	3.15883100

C	1.96981900	-2.96220300	2.10130900
C	2.70779600	-3.90910000	1.38504100
H	2.23551600	-4.86213200	1.12948700
C	4.01978200	-3.65937000	0.96601500
C	4.60406600	-2.43547100	1.30983600
H	5.62487300	-2.21940000	0.98315300
C	3.90434900	-1.46000600	2.02698300
C	-1.09561300	1.09701600	-0.29876400
C	-2.32620300	0.45826000	-0.52125200
C	-2.97222200	0.67679900	-1.74027800
H	-3.91657300	0.16755000	-1.93916600
C	-2.42133300	1.51206200	-2.71902700
C	-1.17917200	2.11035500	-2.47367200
H	-0.72011800	2.72738000	-3.24722800
C	-0.49176800	1.91043500	-1.27327500
C	-2.91314700	-0.42726300	0.57104600
H	-2.05615400	-0.88257600	1.08561400
C	-3.73953700	-1.59063400	0.04251400
C	-3.11611900	-2.82779300	-0.16724600
H	-2.04760000	-2.92655700	0.03516500
C	-3.84322900	-3.92163600	-0.64039200
H	-3.34199700	-4.88033500	-0.79362000
C	-5.20533200	-3.79070700	-0.92237100
H	-5.77518800	-4.64559600	-1.29408700
C	-5.83535600	-2.55926800	-0.72115400

H	-6.90169200	-2.44861300	-0.93307600
C	-5.10848300	-1.46950400	-0.23479100
H	-5.61300000	-0.51840500	-0.05177300
C	-3.66580700	0.37614400	1.62753800
C	-4.28005800	1.60163600	1.34309200
H	-4.21903700	2.01286500	0.33375500
C	-4.94745000	2.31453100	2.34533800
H	-5.41685300	3.27227200	2.10691700
C	-5.00879200	1.80981200	3.64571000
H	-5.52395600	2.36929200	4.43016000
C	-4.40428200	0.58152200	3.93673600
H	-4.44417300	0.17801900	4.95154800
C	-3.74231100	-0.12668900	2.93458600
H	-3.26195700	-1.08068100	3.16718700
C	-3.14680400	1.73653000	-4.02112700
H	-3.99579800	2.42923400	-3.89017600
H	-3.55907700	0.79365500	-4.41412100
H	-2.48249800	2.16653700	-4.78479100
C	0.90258300	2.46695200	-1.00737900
H	1.50149600	1.57407800	-0.74941900
C	0.93789400	3.37110400	0.21395400
C	-0.08108700	4.29423800	0.48417700
H	-0.92800000	4.37505600	-0.20156500
C	-0.04294300	5.08147100	1.63820100
H	-0.84712100	5.79497400	1.83569300

C	1.01184300	4.94717300	2.54507200
H	1.03593300	5.55217600	3.45466400
C	2.03382500	4.02966500	2.28274000
H	2.85772800	3.90937600	2.99042000
C	1.99774400	3.25456700	1.12275000
H	2.78608900	2.52466000	0.92433300
C	1.57195500	3.07306300	-2.22816900
C	1.32457000	4.38747900	-2.64792400
H	0.65587900	5.02353800	-2.06464400
C	1.93183000	4.89385700	-3.79983200
H	1.72792100	5.92091800	-4.11319100
C	2.80175500	4.09399100	-4.54704000
H	3.27797500	4.49148100	-5.44664600
C	3.06605100	2.78703500	-4.12903100
H	3.75350500	2.15593400	-4.69767800
C	2.45694100	2.28299400	-2.97703100
H	2.67496400	1.26536600	-2.64320100
C	0.53454400	-3.22990700	2.46953000
H	0.27456700	-4.28785200	2.32338300
H	-0.13313600	-2.62598200	1.83169800
H	0.32131900	-2.95970400	3.51507800
C	4.52830200	-0.11663300	2.30451100
H	4.60778500	0.08929700	3.38299500
H	3.91176200	0.68634700	1.86871000
H	5.53354300	-0.04858800	1.86551700

C	4.75731500	-4.65511100	0.10939200
H	5.84678700	-4.58198300	0.24589600
H	4.54469500	-4.46532100	-0.95685900
H	4.44934000	-5.68872800	0.32910200
Pd	1.62680100	-0.91907400	-0.38209900
C	1.10337700	-1.86794000	-2.29248800
C	0.52434800	-3.11679300	-2.00825600
C	-0.75380600	-3.39213600	-2.49648800
C	-1.45157300	-2.47842900	-3.30131100
C	-0.80569200	-1.27625600	-3.63421200
C	0.46647300	-0.96864200	-3.16440100
H	1.06251700	-3.83919600	-1.39393800
H	-1.21873600	-4.34909900	-2.24587000
H	-1.31077200	-0.55879900	-4.28373700
H	0.94926900	-0.03272100	-3.44068100
C	-2.85561400	-2.74816200	-3.77245000
H	-3.02113600	-2.36044200	-4.79022000
H	-3.59158600	-2.26272400	-3.10832700
H	-3.08764300	-3.82314100	-3.77223000
Cl	3.01968600	-1.76740000	-2.18798700

TS2-h

N	0.17892700	0.77717000	-1.23768500
C	0.73799000	-0.47471200	-1.06720200

N	1.45971100	-0.67277800	-2.21316100
C	0.53450800	1.33974500	-2.46959900
H	0.10651600	2.26987200	-2.81965600
C	1.29313000	0.40863800	-3.13032200
C	1.78058600	0.28989000	-4.46433800
H	1.54386800	1.07840500	-5.17993400
C	2.45517700	-0.87745100	-4.81831500
H	2.82710200	-1.00549600	-5.84108600
C	2.71789600	-1.87257200	-3.86125100
H	3.37830900	-2.70431500	-4.10928100
C	2.88173200	-2.59510900	-1.47101100
C	2.29157800	-1.76503700	-2.52622900
C	3.70223000	-1.99578600	-0.46409700
C	4.41682300	-2.80252500	0.41963900
H	5.04153300	-2.31723400	1.17848900
C	4.36730100	-4.20586900	0.36693600
C	3.58656300	-4.78196500	-0.64418200
H	3.51950000	-5.87483900	-0.70595700
C	2.86028300	-4.02087600	-1.56343800
C	-0.63857700	1.42172700	-0.25876100
C	-0.06280800	2.39256000	0.57314800
C	-0.83025300	2.93859500	1.61105000
H	-0.36284800	3.64264000	2.30366500
C	-2.15214200	2.53845200	1.81968500
C	-2.73133300	1.64147400	0.90878600

H	-3.75744500	1.32360600	1.08159300
C	-2.00231000	1.07403000	-0.13388700
C	1.37049800	2.84026400	0.30320900
H	1.91822100	1.94167800	-0.01450200
C	-2.94392600	2.93307100	3.03482500
H	-3.22951200	1.98471200	3.54277400
H	-3.87463600	3.46245600	2.75970200
H	-2.36147800	3.58050100	3.71098200
C	-2.62317800	0.09695000	-1.13562600
C	3.82585800	-0.50209500	-0.35831200
H	2.89724700	-0.07278900	0.05571800
C	5.09258000	-5.04480300	1.38690300
H	6.11798200	-4.67332000	1.57350000
H	5.17126000	-6.09654400	1.06252300
H	4.57649300	-5.04489700	2.36637700
Pd	-0.03347600	-1.80627600	0.28028800
C	0.65487600	-0.93940800	1.95716100
C	1.94682200	-1.20920100	2.43640400
C	2.34360200	-0.82387000	3.72747600
C	1.46134500	-0.15498900	4.58549900
C	0.17065200	0.11456000	4.10325800
C	-0.22724600	-0.26399200	2.82023000
H	2.64916700	-1.76750400	1.81921500
H	3.35782100	-1.06474100	4.07225100
H	-0.58318800	0.58596700	4.74205100

H	-1.28069800	-0.09166600	2.58114200
C	1.86289800	0.24490900	5.98550500
H	2.89745800	-0.06580200	6.21269800
H	1.20216900	-0.21153400	6.74476700
H	1.80399800	1.33890500	6.13347100
N	-2.00006100	-3.80103300	1.16763300
H	-2.00856500	-0.81597200	-1.09869400
C	2.00979700	-4.71579200	-2.59465400
H	1.92905100	-5.79185400	-2.36715200
H	2.41054600	-4.61637100	-3.61947300
H	0.99802000	-4.27654400	-2.59916100
H	3.97613500	-0.03866300	-1.34737100
H	4.65623800	-0.21633300	0.30504800
C	1.44842300	3.82324400	-0.85804300
C	0.47564600	4.81444400	-1.04842800
C	2.52138900	3.74781800	-1.75602500
C	0.58056800	5.71905500	-2.12087700
H	-0.38064200	4.85304600	-0.37257400
C	2.63489900	4.65042000	-2.82296200
H	3.26299400	2.95650500	-1.62604500
C	1.65600800	5.63802700	-3.00595800
H	-0.19278000	6.47996600	-2.26531800
H	3.46753100	4.56039200	-3.52622000
H	1.72838500	6.33418500	-3.84833900
C	2.09556300	3.34871700	1.54304200

C	2.44253100	4.69323200	1.73259900
C	2.44102800	2.41797800	2.53703800
C	3.12113200	5.10301800	2.88698600
H	2.18058300	5.42576800	0.96710300
C	3.12492700	2.82356700	3.68355300
H	2.16558100	1.36996100	2.41265400
C	3.46827500	4.16763400	3.86673000
H	3.38099400	6.15896900	3.01612500
H	3.38023200	2.07457500	4.43529600
H	3.99972100	4.48396000	4.77024500
C	-2.51183000	0.63449600	-2.55152100
C	-2.03972600	-0.19293100	-3.58099000
C	-2.83598400	1.96554100	-2.85227800
C	-1.88509900	0.30523300	-4.87656000
H	-1.74277100	-1.21665900	-3.33382600
C	-2.68670100	2.46433700	-4.14884100
H	-3.17737000	2.62037200	-2.04686400
C	-2.20717500	1.63401300	-5.16837100
H	-1.46801300	-0.34197700	-5.65288300
H	-2.92723700	3.51133900	-4.36029400
H	-2.06452700	2.02876000	-6.17972000
C	-4.03158000	-0.35202200	-0.76402200
C	-4.20073500	-1.07573500	0.42645900
C	-5.16247900	-0.06951300	-1.54373800
C	-5.46831800	-1.46723900	0.86514000

H	-3.34375800	-1.30840800	1.05839300
C	-6.43328700	-0.48751100	-1.12917400
H	-5.05176300	0.47935700	-2.48094600
C	-6.58942700	-1.17359000	0.07899100
H	-5.54893900	-1.92695000	1.85719100
H	-7.30622500	-0.25991900	-1.75241500
H	-7.58919500	-1.46886300	0.41695300
Cl	-1.00594300	-3.08987200	-1.69583300
N	-1.24394800	-2.78198300	1.57774300
C	-3.76237200	-1.18304800	3.54951600
H	-2.91452100	-3.77149800	1.64066800
H	-1.98580100	-3.97521400	0.15366800
O	-3.16924700	-0.04720100	3.49587700
O	-3.09400700	-2.26268500	3.10658500
O	-4.94357500	-1.35525200	3.95604800
H	-1.95137000	-2.30999300	2.45506700

TS2

N	0.50701600	-0.55206700	1.20433900
C	-0.41800100	-1.18442600	0.41298200
N	-0.73357000	-2.31519500	1.13261000
C	0.79294200	-1.25340100	2.35739300
H	1.52433900	-0.92032900	3.08011100
C	0.03753100	-2.38942200	2.33030400
C	-0.04226700	-3.53382100	3.16776900
H	0.59213100	-3.56762400	4.05449800

C	-0.88498100	-4.54960100	2.82016200
H	-0.96212800	-5.45046600	3.43327800
C	-1.70883700	-4.41427600	1.65233100
H	-2.45061400	-5.17869700	1.41880700
C	-2.67285400	-3.13854600	-0.23843800
C	-1.65375800	-3.32158900	0.83073400
C	-3.72661900	-2.22974600	0.00089000
C	-4.78048300	-2.15528500	-0.91508200
H	-5.59290100	-1.44738000	-0.72609600
C	-4.79953000	-2.93011000	-2.07834000
C	-3.75475900	-3.83566500	-2.28335600
H	-3.75380400	-4.45618100	-3.18433700
C	-2.69903500	-3.97705000	-1.37418400
C	1.09574200	0.72642800	0.86458200
C	0.40369600	1.88478500	1.26251300
C	0.89783400	3.12677900	0.86207900
H	0.33796200	4.02847000	1.11304800
C	2.05210300	3.23769200	0.07596300
C	2.75960700	2.06706100	-0.23889400
H	3.62625900	2.15739700	-0.89269700
C	2.31147600	0.80081400	0.14857600
C	-0.85865200	1.74183400	2.10836100
H	-1.48925900	1.00232800	1.59521100
C	2.45378700	4.54948700	-0.52363200
H	2.02590200	4.54301900	-1.55718400

H	3.54458800	4.64073000	-0.62580800
H	2.05500800	5.40052100	0.05481500
C	3.08801400	-0.47425700	-0.20139900
C	-3.70802700	-1.31541700	1.19648000
H	-2.97883200	-0.50669200	1.02764600
C	-5.89118900	-2.75163600	-3.10274200
H	-6.86332000	-2.54000300	-2.62828500
H	-6.00317000	-3.64439600	-3.73786700
H	-5.66190400	-1.90046700	-3.76755300
Pd	-0.56537400	-0.71809000	-1.54421600
C	-1.75112600	0.90563000	-1.45255100
C	-3.15344200	0.83991800	-1.47997400
C	-3.93863500	1.98287200	-1.70682600
C	-3.34645800	3.23281600	-1.92892700
C	-1.94469800	3.29721800	-1.90742700
C	-1.16385700	2.16662700	-1.66521700
H	-3.65255500	-0.12035100	-1.37395200
H	-5.03171800	1.88871800	-1.73151400
H	-1.41320200	4.22629800	-2.13241200
H	-0.08568900	2.30836500	-1.75931900
C	-4.17226200	4.46502500	-2.20924800
H	-5.25273600	4.25182500	-2.14762400
H	-3.96752400	4.86910100	-3.21664100
H	-3.94730900	5.27518400	-1.49371300
N	0.24913600	-0.92786400	-4.50052200

H	2.39663600	-1.10401600	-0.78451500
C	-1.61769000	-4.99079000	-1.63404700
H	-1.78990200	-5.50215800	-2.59301800
H	-1.57159500	-5.75403900	-0.84100200
H	-0.63544400	-4.48834100	-1.67629600
H	-3.41247000	-1.84380200	2.11620800
H	-4.69072100	-0.84863800	1.35552500
C	-0.55958800	1.18554700	3.49604900
C	0.63785400	1.47901700	4.16266300
C	-1.49147300	0.35340700	4.13042100
C	0.90581500	0.93653200	5.42219400
H	1.38012200	2.10420100	3.66192600
C	-1.22598600	-0.19669300	5.38492000
H	-2.42208000	0.10989700	3.61239600
C	-0.02183900	0.09012900	6.03643800
H	1.85403300	1.16217000	5.91801000
H	-1.95459600	-0.86728700	5.84887200
H	0.19508700	-0.35000300	7.01377000
C	-1.70251700	3.00367300	2.21624100
C	-1.41946300	4.01347500	3.14825800
C	-2.80324400	3.16358400	1.36639300
C	-2.21512000	5.15871700	3.22506600
H	-0.56791000	3.90053900	3.82195600
C	-3.60630900	4.30364600	1.44734900
H	-3.02613300	2.39499400	0.62629300

C	-3.31617000	5.30685900	2.37520500
H	-1.97409500	5.93866400	3.95336600
H	-4.45498100	4.40434900	0.76744600
H	-3.94121000	6.20273500	2.43419700
C	3.45212000	-1.26202600	1.04332400
C	3.26001300	-2.65004300	1.07604700
C	3.98503600	-0.62902800	2.17656200
C	3.56871300	-3.38380600	2.22401700
H	2.80982500	-3.13223800	0.20413800
C	4.29529000	-1.35982000	3.32601500
H	4.12926900	0.45376100	2.15453400
C	4.08237100	-2.74303700	3.35642400
H	3.38349500	-4.46167000	2.24199200
H	4.69744500	-0.84776800	4.20544400
H	4.31306300	-3.31648800	4.25934900
C	4.30841900	-0.25854800	-1.10014000
C	4.09901100	0.20810800	-2.40584500
C	5.62342100	-0.51469800	-0.67452000
C	5.18562200	0.44519300	-3.25023500
H	3.09201500	0.46039600	-2.77866200
C	6.70666800	-0.30581500	-1.53371000
H	5.81232900	-0.88633900	0.33381900
C	6.49005200	0.18110100	-2.82769100
H	4.97778500	0.88917700	-4.22553600
H	7.72323400	-0.51537900	-1.18366000

H	7.33941500	0.36907000	-3.49354100
Cl	0.94053300	-2.71655800	-1.84892900
N	-0.32554300	-0.04346300	-3.50742600
C	2.01895400	2.68113600	-3.24989700
H	1.13623800	-0.46259800	-4.72975300
H	0.53835500	-1.76825900	-3.99202200
H	-1.21256900	0.30064500	-3.87487000
O	1.09850200	3.53135000	-2.99853700
O	1.66356500	1.41913300	-3.55142900
O	3.25628500	2.93854000	-3.18551200
H	0.48851100	0.85126500	-3.44656200

TS3-*t*

N	0.40829100	1.12509800	-0.77579300
C	-0.65753400	0.29762500	-1.00061800
N	-0.99628500	0.57962700	-2.30398700
C	0.71717400	1.92707600	-1.86047600
H	1.51470500	2.65837200	-1.83900800
C	-0.17031000	1.59833800	-2.85079100
C	-0.40366800	2.04411000	-4.18231100
H	0.22907400	2.83432400	-4.58800600
C	-1.40799300	1.46770700	-4.90608900
H	-1.60867800	1.79001200	-5.92951600
C	-2.19941400	0.41299400	-4.33424300

H	-2.97487700	-0.07280100	-4.92712500
C	-2.74995200	-1.17001400	-2.46474400
C	-1.99578900	-0.03281700	-3.05979700
C	-2.16374800	-2.45293900	-2.43256500
C	-2.91965200	-3.52421300	-1.94329900
H	-2.46899400	-4.52033100	-1.92255100
C	-4.22566900	-3.35309100	-1.47119700
C	-4.77266900	-2.06263700	-1.48552500
H	-5.78043600	-1.90292300	-1.09212800
C	-4.05731000	-0.96542600	-1.97576400
C	1.15377700	1.09668900	0.45089000
C	2.35190500	0.36891500	0.48349400
C	3.07363800	0.33470200	1.68073800
H	3.99454300	-0.24924600	1.72464000
C	2.62488300	1.00709500	2.82194900
C	1.41446800	1.71271800	2.75694600
H	1.03902400	2.21593900	3.64931600
C	0.65519000	1.76024400	1.58567200
C	2.82341800	-0.35285300	-0.77282200
H	1.91469200	-0.72955400	-1.26374300
C	3.67141600	-1.58208000	-0.47833400
C	3.02295000	-2.78673300	-0.17410200
H	1.93174700	-2.81836600	-0.13648100
C	3.75985800	-3.94093600	0.09278700
H	3.23578400	-4.87127600	0.31783900

C	5.15737900	-3.90353200	0.07717400
H	5.73425700	-4.80768300	0.28677600
C	5.81141800	-2.70303900	-0.21159700
H	6.90352000	-2.66367400	-0.22782100
C	5.07200100	-1.55061200	-0.49413000
H	5.58880600	-0.62114800	-0.74085200
C	3.48521300	0.59195700	-1.76838800
C	4.18250300	1.73612400	-1.36179700
H	4.25860500	1.96929500	-0.29796100
C	4.75674600	2.59441200	-2.30560000
H	5.29209600	3.48661900	-1.97119300
C	4.64213800	2.31738300	-3.66970900
H	5.08410800	2.99119900	-4.40759700
C	3.95757000	1.16935100	-4.08417700
H	3.86095700	0.94332000	-5.14899500
C	3.38878800	0.31558200	-3.13957100
H	2.84309100	-0.57346600	-3.46626400
C	3.38711400	0.91889000	4.11899200
H	3.31914700	1.85545500	4.69306500
H	4.44972200	0.69020000	3.95029400
H	2.97534600	0.11694200	4.75643000
C	-0.71106600	2.43334000	1.50806400
H	-1.37944200	1.64143700	1.12563000
C	-0.74166500	3.55293100	0.48241600
C	0.32750100	4.44604100	0.33053200

H	1.20568400	4.34488400	0.97271600
C	0.29484500	5.43701800	-0.65423700
H	1.13831200	6.12382000	-0.76177600
C	-0.80657900	5.54218900	-1.50811600
H	-0.82789000	6.30847000	-2.28677300
C	-1.87952600	4.65771700	-1.36158900
H	-2.74153500	4.72643500	-2.02962700
C	-1.84681800	3.67614800	-0.36989900
H	-2.67802900	2.97429900	-0.26508800
C	-1.27305900	2.81514900	2.86614200
C	-1.05060400	4.06645100	3.45471000
H	-0.48911700	4.82759600	2.90962500
C	-1.54893800	4.35097100	4.72957700
H	-1.36813700	5.33247100	5.17519000
C	-2.28033800	3.38852700	5.43069400
H	-2.67191700	3.61294100	6.42587800
C	-2.51642200	2.14042100	4.84604700
H	-3.09787700	1.38514600	5.38076400
C	-2.01727900	1.85814800	3.57324500
H	-2.20951800	0.88828600	3.10625800
C	-0.73351900	-2.66037600	-2.85369400
H	-0.50871200	-3.72618900	-2.99902900
H	-0.05966700	-2.27799500	-2.06838000
H	-0.49650200	-2.12277700	-3.78428600
C	-4.65159400	0.41953900	-1.93494100

H	-4.80356500	0.83113000	-2.94480300
H	-3.97940400	1.11299900	-1.40423100
H	-5.61898000	0.41945000	-1.41368400
C	-5.02901400	-4.52134800	-0.95646300
H	-4.39155700	-5.39833000	-0.77129600
H	-5.80484400	-4.81975100	-1.68194600
H	-5.54720600	-4.27058100	-0.01682600
Pd	-1.64329600	-0.75858500	0.40065400
C	-0.68831900	-2.19331200	1.57704400
C	-0.55251400	-3.50950300	1.09796400
C	0.45080700	-4.35049100	1.59978100
C	1.34548900	-3.92050500	2.58676000
C	1.21161000	-2.59681300	3.04642800
C	0.21775700	-1.75049300	2.56476900
H	-1.23512300	-3.88128000	0.33034500
H	0.53618100	-5.36871300	1.20605800
H	1.90530700	-2.22076400	3.80516000
H	0.14284800	-0.73398100	2.95546200
C	2.41973800	-4.82017200	3.14170800
H	3.42642100	-4.41381800	2.94607900
H	2.37461000	-5.82469900	2.69396600
H	2.32394500	-4.93860100	4.23454500
N	-3.39394100	-2.82139800	1.80493600
H	-2.86457300	-3.69819500	1.92042900
H	-3.77944100	-2.82586500	0.86337100

N	-2.56227000	-1.71622000	1.93029400
H	-2.39347600	-1.51929100	2.91129700

TS3-*tx*

N	1.51375200	-0.08335900	0.74536600
C	0.21525400	-0.18843900	1.14767300
N	0.31934300	-0.18442500	2.51369700
C	2.41448100	-0.00696100	1.79294900
H	3.48481500	0.06177600	1.64045500
C	1.66867100	-0.08417400	2.94307500
C	1.95058200	-0.10538000	4.33857000
H	2.98919000	-0.05373200	4.66723200
C	0.91079300	-0.19451400	5.22043400
H	1.09999200	-0.21577700	6.29542000
C	-0.44534400	-0.24510600	4.74717600
H	-1.27306800	-0.27998000	5.45597900
C	-2.13115500	-0.22575400	2.87810000
C	-0.74206100	-0.23308700	3.41444000
C	-2.75788500	1.01001000	2.60226200
C	-4.10486200	1.01081600	2.23021700
H	-4.59114400	1.96435400	2.00935600
C	-4.84272300	-0.17357700	2.12790100
C	-4.18544800	-1.38714400	2.35910800
H	-4.73638300	-2.32448800	2.24357600

C	-2.83783600	-1.43700500	2.72841500
C	1.85485700	-0.00481500	-0.64444100
C	2.03429700	1.26166200	-1.21260500
C	2.32561300	1.33938600	-2.58035800
H	2.43979100	2.32201700	-3.04245000
C	2.42750700	0.18860000	-3.36846600
C	2.22681000	-1.06210600	-2.76534100
H	2.26569600	-1.96192700	-3.38024900
C	1.92128500	-1.18360600	-1.41011600
C	1.86783200	2.50554100	-0.35237900
H	1.21728800	2.21686900	0.48367700
C	1.10150000	3.58793200	-1.10069500
C	-0.28990000	3.44618900	-1.22246300
H	-0.77819300	2.58634100	-0.75072200
C	-1.03898900	4.38302000	-1.93465000
H	-2.12267900	4.26572300	-2.01426100
C	-0.40574900	5.47460500	-2.53955800
H	-0.99108300	6.20952000	-3.09732800
C	0.97768700	5.62308500	-2.41931400
H	1.47869100	6.47592700	-2.88411900
C	1.72775300	4.68573300	-1.70053600
H	2.80780400	4.81141700	-1.59935600
C	3.16826800	2.98705400	0.27647800
C	4.42868900	2.64532100	-0.22807400
H	4.50146700	2.00246500	-1.10674800

C	5.59505600	3.11253500	0.38875800
H	6.57017900	2.83412000	-0.01874400
C	5.51604900	3.92671600	1.51956900
H	6.42662300	4.28898600	2.00273700
C	4.26051800	4.27329900	2.03177100
H	4.18661000	4.90701000	2.91905000
C	3.10090400	3.80614500	1.41470000
H	2.12151900	4.07666500	1.81837200
C	2.65087600	0.28565200	-4.85458300
H	3.27607100	-0.54043300	-5.22602800
H	3.12563100	1.23729900	-5.13548600
H	1.68145900	0.22617300	-5.37864000
C	1.56291900	-2.51499800	-0.75501700
H	0.56150400	-2.34191300	-0.32616100
C	2.48280800	-2.84692000	0.40705300
C	3.86727800	-2.64486200	0.32593600
H	4.29650100	-2.24775900	-0.59707600
C	4.69371100	-2.91882600	1.41877600
H	5.77120900	-2.75286800	1.34026700
C	4.14385600	-3.39419500	2.61290300
H	4.78733100	-3.59767100	3.47227900
C	2.76479300	-3.60545000	2.70052000
H	2.32369100	-3.96857700	3.63189300
C	1.94494700	-3.33920900	1.60258000
H	0.86539000	-3.48812400	1.67835000

C	1.39597000	-3.65290400	-1.75263600
C	2.34582000	-4.66629200	-1.92428200
H	3.24720100	-4.67518000	-1.30955100
C	2.14478400	-5.67656800	-2.87172400
H	2.89527600	-6.46214800	-2.99115200
C	0.99096600	-5.68602800	-3.65736900
H	0.83388000	-6.47667500	-4.39525600
C	0.03566200	-4.67700000	-3.49030700
H	-0.87117500	-4.67512700	-4.10063600
C	0.23637700	-3.67122000	-2.54528700
H	-0.48901100	-2.86337500	-2.42148900
C	-1.98178900	2.29871500	2.68792400
H	-1.50727200	2.42425500	3.67374700
H	-2.62991600	3.16617500	2.50141100
H	-1.17682400	2.31282000	1.93488000
C	-2.13603200	-2.75722300	2.91621700
H	-2.83126700	-3.59968000	2.79517000
H	-1.66331600	-2.83704000	3.90701400
H	-1.33569700	-2.86739000	2.16576500
C	-6.31456000	-0.13239600	1.81507100
H	-6.55111600	0.69451400	1.13170000
H	-6.90337900	0.01438300	2.73772600
H	-6.65468500	-1.06738900	1.34796900
Pd	-1.42814700	-0.24937600	-0.13878700
C	-3.09361900	-0.04050500	-1.27994000

C	-4.08724800	-1.04186300	-1.20185500
C	-5.43101300	-0.71870400	-1.35919900
C	-5.84802500	0.60634900	-1.59387900
C	-4.85885300	1.59470200	-1.66929500
C	-3.50057800	1.28021600	-1.53915200
H	-3.79520500	-2.08051000	-1.02080400
H	-6.18325700	-1.51127900	-1.29362300
H	-5.15298600	2.63438800	-1.84544100
H	-2.75182500	2.06820400	-1.63909900
C	-7.30842000	0.93153700	-1.78149900
H	-7.92844700	0.49072600	-0.98290800
H	-7.69324400	0.53585900	-2.73778200
H	-7.48246000	2.01822400	-1.78121400
N	-1.07079000	0.36295000	-3.14286900
H	-0.25802100	0.84919900	-2.76788800
H	-1.79912700	1.06605800	-3.32919700
N	-1.47889800	-0.61137200	-2.24838800
H	-2.07157900	-1.27564400	-2.73239600

TS3-*x*

N	-1.48256500	0.30073300	0.66070100
C	-0.44342400	-0.52136300	1.00497200
N	-0.82650500	-0.98350800	2.24279600
C	-2.47279900	0.38385600	1.62774900

H	-3.34229100	1.02224900	1.52437000
C	-2.06396500	-0.41742000	2.66101400
C	-2.57499600	-0.73219500	3.95235500
H	-3.50357100	-0.26151800	4.27905100
C	-1.88210800	-1.60638300	4.74047300
H	-2.24899600	-1.86425100	5.73648000
C	-0.67455200	-2.21605100	4.25752300
H	-0.15777900	-2.96025700	4.86369600
C	1.03249200	-2.61555300	2.46133500
C	-0.15325700	-1.91791500	3.02933300
C	0.83812100	-3.56768100	1.43678900
C	1.94844200	-4.23864600	0.91395100
H	1.79151200	-4.98114400	0.12535700
C	3.24660400	-3.97324900	1.36343200
C	3.40780100	-3.05328700	2.40322400
H	4.41324400	-2.83637100	2.77390400
C	2.32448700	-2.37818400	2.97891300
C	-1.56833800	0.88093200	-0.64900300
C	-2.10117400	0.09666500	-1.68052700
C	-2.14077500	0.62829300	-2.97556100
H	-2.51993100	0.00605400	-3.78948500
C	-1.67199800	1.91609800	-3.24487800
C	-1.14924300	2.67668500	-2.18794800
H	-0.75756300	3.67496900	-2.38989300
C	-1.08068600	2.18125700	-0.88407600

C	-2.60648000	-1.30723700	-1.38418200
H	-2.06459100	-1.64031700	-0.49149000
C	-2.20642700	-2.29360700	-2.47474800
C	-0.83406700	-2.54910100	-2.63081500
H	-0.10300700	-2.02771300	-2.00286900
C	-0.38126500	-3.44078000	-3.60176600
H	0.69598700	-3.60967800	-3.68332300
C	-1.29952300	-4.09147600	-4.43573900
H	-0.95067800	-4.79279400	-5.19898900
C	-2.66581700	-3.84098600	-4.28743000
H	-3.38905000	-4.34592000	-4.93421800
C	-3.11966200	-2.94640700	-3.30891600
H	-4.18979200	-2.76209000	-3.19276100
C	-4.07918500	-1.34401400	-1.00939800
C	-5.02967400	-0.49524400	-1.59147000
H	-4.70773200	0.24459700	-2.32632400
C	-6.37771000	-0.57557700	-1.22842000
H	-7.10352500	0.09843300	-1.69134800
C	-6.79626200	-1.50500300	-0.27327100
H	-7.84898300	-1.56325000	0.01537200
C	-5.85484200	-2.35400200	0.31773300
H	-6.16747200	-3.07835800	1.07442700
C	-4.51106900	-2.27090500	-0.04849600
H	-3.77475900	-2.92749800	0.42180300
C	-1.68149600	2.47333000	-4.64632800

H	-2.26374600	1.83835800	-5.33061900
H	-0.65578400	2.54498700	-5.04655300
H	-2.10724400	3.48966600	-4.67212400
C	-0.47532400	2.97009500	0.26768800
H	0.22100800	2.28279000	0.77391900
C	-1.51331600	3.35171800	1.30970300
C	-2.80820600	3.76141200	0.96439500
H	-3.09048200	3.81386400	-0.09008500
C	-3.74955500	4.06172400	1.95420900
H	-4.75873800	4.37240600	1.66926800
C	-3.40407900	3.95392200	3.30443600
H	-4.14140600	4.17844500	4.08016100
C	-2.11120200	3.54909600	3.65540000
H	-1.83697400	3.44726100	4.70890200
C	-1.17192100	3.25176400	2.66697800
H	-0.17240000	2.89017800	2.92591000
C	0.38109000	4.14019100	-0.19042200
C	-0.14449800	5.41493800	-0.44266800
H	-1.20376100	5.60841200	-0.25906500
C	0.67565800	6.44208200	-0.92048000
H	0.25038800	7.43138700	-1.11280900
C	2.03491400	6.20654600	-1.14807900
H	2.67647400	7.00940600	-1.52223900
C	2.56908200	4.94144000	-0.88307500
H	3.63104300	4.73978200	-1.04526900

C	1.75037100	3.91778100	-0.40251900
H	2.17305200	2.93908000	-0.16903500
C	-0.52973600	-3.85046300	0.87616900
H	-1.30510300	-3.85052900	1.65767300
H	-0.55528400	-4.81784100	0.35458600
H	-0.79660700	-3.07393700	0.14404000
C	2.56028000	-1.39123900	4.08979900
H	3.62724900	-1.35388800	4.35323400
H	1.98892000	-1.64609400	4.99553300
H	2.25603600	-0.38311100	3.75966400
C	4.43985300	-4.61037100	0.70016800
H	4.19350600	-5.59604700	0.27563500
H	5.27963300	-4.73234900	1.40152100
H	4.79665000	-3.97283200	-0.12841900
Pd	1.50722700	-0.29353100	0.09637800
C	3.23096600	-0.06877300	-0.94933500
C	3.44268100	1.09323100	-1.75717500
C	4.71671000	1.53883000	-2.07278100
C	5.87834600	0.87464100	-1.61654200
C	5.68237200	-0.25716500	-0.81718500
C	4.40715000	-0.73311800	-0.49024100
H	2.57462400	1.64501800	-2.13083000
H	4.82380200	2.44047400	-2.68852500
H	6.55818900	-0.77895900	-0.41192300
H	4.31036300	-1.56665600	0.20856700

C	7.25442600	1.37376200	-1.97807500
H	7.47866600	1.25892100	-3.05640800
H	8.03509900	0.82519900	-1.42604300
H	7.38094500	2.44732300	-1.74601000
N	2.53628200	-2.48896600	-1.93721500
H	2.23271900	-3.09314500	-1.17424800
H	3.56762600	-2.42451000	-1.83830800
N	1.98654100	-1.22247900	-1.73030300
H	1.83403400	-0.75241500	-2.61592600
Cl	1.94932300	1.30616500	2.03527300

TS3

N	1.03723400	0.37127900	0.90984200
C	0.10607200	-0.63054900	0.96071600
N	0.22435300	-1.09975700	2.24977800
C	1.77681700	0.49145500	2.07543900
H	2.57600000	1.21306900	2.19044200
C	1.27584900	-0.43775400	2.94556400
C	1.58099200	-0.81543600	4.28265200
H	2.39470400	-0.30242800	4.79713200
C	0.84454900	-1.80110100	4.87404800
H	1.05293200	-2.11042600	5.90061100
C	-0.22752300	-2.42902600	4.15485200
H	-0.83983900	-3.18692500	4.64313900

C	-1.69977200	-2.68984300	2.15047300
C	-0.53727200	-2.09438600	2.86579600
C	-2.84673800	-1.90046500	1.90688400
C	-3.95456900	-2.48504800	1.28795700
H	-4.84211300	-1.87106200	1.11180200
C	-3.94249100	-3.81589500	0.85991900
C	-2.80750700	-4.58399700	1.13447400
H	-2.78276800	-5.63219700	0.82317700
C	-1.69127100	-4.05873900	1.79929400
C	1.14630000	1.25083000	-0.21934700
C	0.32783400	2.39279900	-0.25679200
C	0.42478900	3.24414600	-1.35873500
H	-0.22821300	4.11677100	-1.41161800
C	1.30624700	2.97340700	-2.41204400
C	2.09971200	1.82218000	-2.34753500
H	2.77016400	1.58335500	-3.17491000
C	2.03271300	0.94235000	-1.26098000
C	-0.64605800	2.64409900	0.88789900
H	-1.11738600	1.67221000	1.08687400
C	1.33688500	3.86607400	-3.62638400
H	0.50472400	3.61695900	-4.30798200
H	2.27342900	3.74971700	-4.19229800
H	1.22427400	4.92705000	-3.35292100
C	2.86223100	-0.32884700	-1.17399600
C	-2.89259300	-0.43618000	2.25011000

H	-2.48880500	0.14103800	1.40291700
C	-5.11230000	-4.38398900	0.09760300
H	-6.07193300	-4.11972500	0.57122700
H	-5.05497600	-5.48021400	0.02186000
H	-5.13868100	-3.97961000	-0.92926800
Pd	-0.63299100	-1.53340500	-0.68124400
C	-1.98267600	-0.49070100	-1.78855300
C	-3.37870900	-0.41841200	-1.48711100
C	-4.21382300	0.51677300	-2.11337200
C	-3.72982800	1.47103200	-3.01504800
C	-2.33317700	1.47415600	-3.23673500
C	-1.48695600	0.55271900	-2.64265800
H	-3.80496500	-1.10821700	-0.75974200
H	-5.28276700	0.51419700	-1.86503200
H	-1.89989000	2.24127400	-3.89006700
H	-0.41517200	0.60627100	-2.84708400
C	-4.62875900	2.46733800	-3.70093000
H	-5.66468400	2.39110300	-3.33068400
H	-4.66608200	2.31717300	-4.79669200
H	-4.29690500	3.50910400	-3.53703200
N	-1.48366900	-2.18432000	-2.42873000
N	-2.55276100	-3.07835400	-2.49996500
H	-3.43541400	-2.54244600	-2.41540500
H	-2.51658000	-3.68782000	-1.68262500
H	2.19520000	-1.11862700	-0.79340600

H	-1.11231700	-1.97236900	-3.34912200
C	-0.51105500	-4.94728500	2.08903700
H	-0.66824400	-5.94603000	1.65592500
H	-0.34387300	-5.06781900	3.17108200
H	0.39928600	-4.51954300	1.63654300
H	-2.29374800	-0.19750500	3.14112900
H	-3.92574500	-0.10019800	2.42045700
C	0.06571600	3.04885600	2.17079300
C	1.23014300	3.82719600	2.15678100
C	-0.44329800	2.62544200	3.40557400
C	1.88192100	4.15832300	3.34816800
H	1.64530200	4.14780800	1.19892800
C	0.20635900	2.94802200	4.59699200
H	-1.34489800	2.00923800	3.42459100
C	1.37611900	3.71447100	4.57286800
H	2.79677100	4.75615200	3.31767000
H	-0.19287900	2.58550600	5.54762700
H	1.89305200	3.96020700	5.50412600
C	-1.78177200	3.58945000	0.52520100
C	-1.74969000	4.95774300	0.82683000
C	-2.89476800	3.06885100	-0.14995300
C	-2.81016700	5.79208000	0.46043700
H	-0.89230800	5.37305800	1.36002200
C	-3.95813800	3.89938300	-0.50656000
H	-2.92627300	2.00861600	-0.40912300

C	-3.92033300	5.26406900	-0.20437700
H	-2.77006200	6.85780000	0.70252300
H	-4.81360400	3.46948600	-1.02975800
H	-4.75354900	5.91374100	-0.48601200
C	3.97913700	-0.20558200	-0.15000200
C	4.27069000	-1.31090900	0.66469600
C	4.69536000	0.98431500	0.03807200
C	5.25735200	-1.22054800	1.64778200
H	3.67452800	-2.22064900	0.53969300
C	5.68527800	1.07395700	1.02224200
H	4.45652500	1.85646400	-0.57549000
C	5.96847500	-0.02935300	1.83213000
H	5.46153100	-2.08375500	2.28721500
H	6.23128700	2.01147100	1.16113600
H	6.73540600	0.04100000	2.60861100
C	3.31047700	-0.82595600	-2.53728000
C	2.40898900	-1.60320700	-3.28332600
C	4.55891300	-0.51765000	-3.09110400
C	2.74735700	-2.04377000	-4.56305800
H	1.44824100	-1.87336300	-2.83761100
C	4.89843100	-0.95988600	-4.37458700
H	5.27460100	0.06835900	-2.51020800
C	3.99188200	-1.72116100	-5.11616100
H	2.03629600	-2.65269200	-5.12758800
H	5.87793000	-0.71133100	-4.79320100

H	4.25688700	-2.07051700	-6.11801300
Cl	1.35958900	-3.28724000	-0.21357200

4-Chlorotoluene

C	0.31189400	1.21407700	-0.00035500
C	-1.08537200	1.20545600	-0.00104800
C	-1.81018200	0.00593300	-0.00114200
C	-1.08767600	-1.19847600	-0.00106500
C	0.30657600	-1.21134900	-0.00035500
H	0.86610700	2.15376000	-0.00052100
H	-1.62176700	2.15804500	-0.00173200
H	-1.62724000	-2.14976000	-0.00176700
H	0.85915200	-2.15205800	-0.00052100
C	-3.31777400	-0.00332000	0.00147100
H	-3.71570500	-0.55547200	-0.86583100
H	-3.72776100	1.01645100	-0.03100300
H	-3.71225700	-0.49633300	0.90557700
C	1.00160300	0.00143800	0.00009400
Cl	2.75088600	-0.00336400	0.00060000

NH_2NH_2

N	0.71911400	-0.00018800	-0.15956600
H	1.09253400	-0.79501600	0.36872000

H	1.09219400	0.79627600	0.36648600
N	-0.71904100	-0.00018600	0.15954800
H	-1.09237300	0.79644700	-0.36640100
H	-1.09286500	-0.79508700	-0.36867900

CO_3^{2-}

C	-0.00004800	0.00015900	0.00003000
O	0.11737500	1.30094000	-0.00000700
O	-1.18559200	-0.54891200	-0.00000700
O	1.06825300	-0.75214700	-0.00000700

HCO_3^-

C	-0.15807500	0.06879400	0.00005600
O	1.01395000	-0.77904800	-0.00004800
H	1.71700100	-0.11477700	0.00027200
O	-1.22852400	-0.54549600	0.00000000
O	0.11850500	1.28729500	-0.00002800

Cl^-

Cl	0.00000000	0.00000000	0.00000000

product

N	-3.20347800	-0.58979000	0.11950200
H	-4.00071300	-0.57051900	-0.51292800
H	-3.53935600	-0.59129100	1.08605400
C	-0.96607900	0.25817000	-0.06689800
C	-0.40630500	-1.02948400	-0.07598200
C	-0.09740100	1.36514000	-0.00748600
C	0.97961200	-1.19107000	-0.03715300
H	-1.07485300	-1.88905200	-0.11158800
C	1.28220900	1.18267900	0.02948500
H	-0.51385400	2.37712600	0.00690700
C	1.85607800	-0.09894500	0.01403900
H	1.39117700	-2.20482300	-0.04491500
H	1.93162700	2.06217900	0.07561300
C	3.35320900	-0.28063300	0.04525000
H	3.81055700	0.25620000	0.89326500
H	3.82869900	0.10637000	-0.87271200
H	3.62628300	-1.34267200	0.13580800
N	-2.34309300	0.47030600	-0.14938300
H	-2.64151200	1.39773100	0.14613500

product-0

N	-3.20257700	-0.57041000	-0.02147300
H	-4.13108800	-0.23000100	0.23525000
H	-2.93529800	-1.18377200	0.77049000

C	-1.08150800	0.31973900	-0.04315100
C	-0.48214400	-1.00142200	-0.07786600
C	-0.12212700	1.40684400	-0.02240000
C	0.89984700	-1.17572100	-0.05146800
H	-1.13228500	-1.87204200	-0.18139900
C	1.24050800	1.19282100	0.00537700
H	-0.53145900	2.42251400	-0.01985200
C	1.81088100	-0.10807200	0.00211600
H	1.29790000	-2.20062300	-0.08454200
H	1.91148100	2.06310700	0.02923600
C	3.30097600	-0.31643900	0.06294800
H	3.75410000	-0.00544900	1.02927700
H	3.84797800	0.24839900	-0.71973000
H	3.55745900	-1.38152000	-0.07453600
N	-2.37419100	0.60368000	-0.01246000

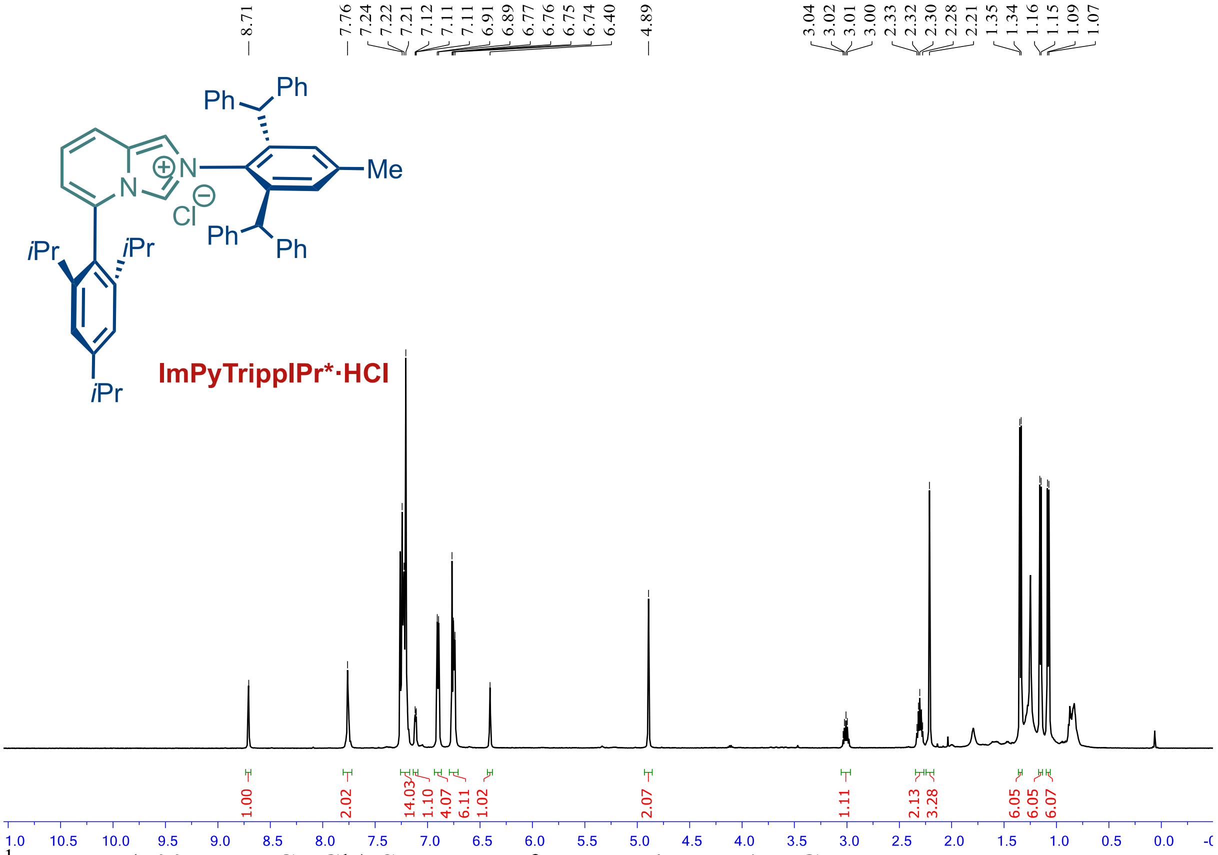

^{1}H NMR (500 MHz, CDCl3) Spectrum of **ImPyTrippIPr*·HCl**

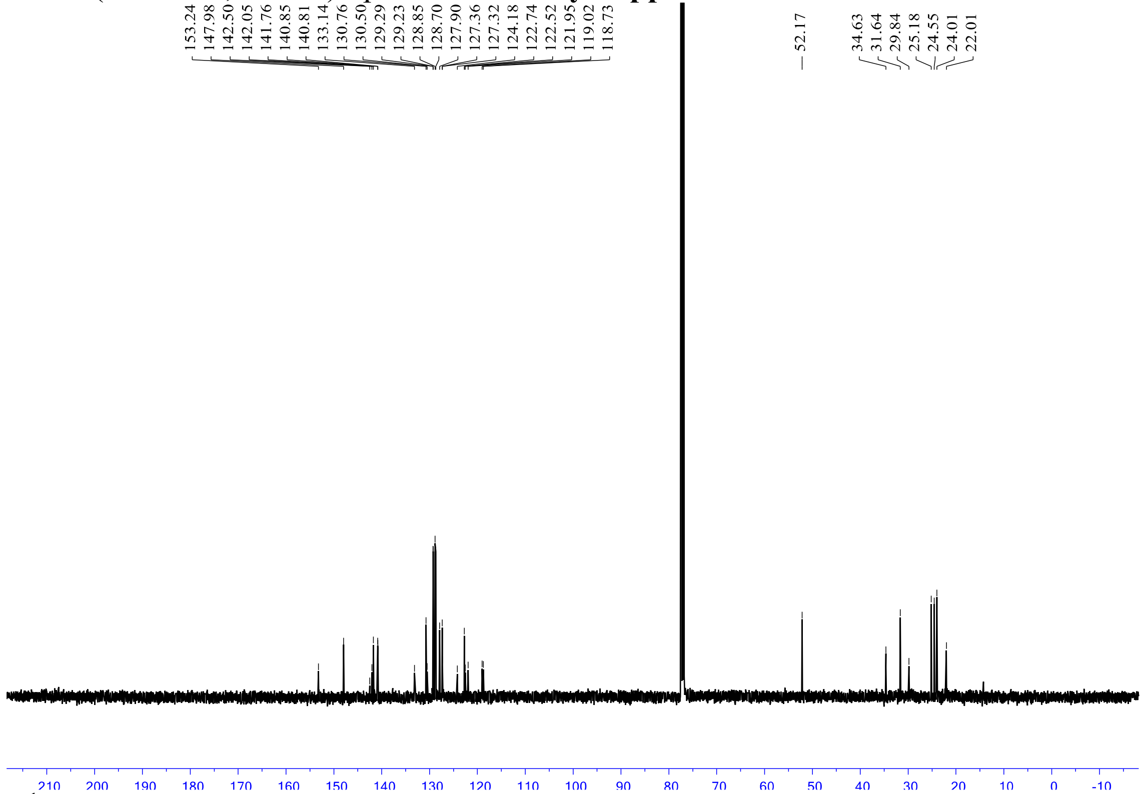

^{13}C{^{1}H} NMR (125 MHz, $CDCl_3$) Spectrum of **ImPyTrippIPr*·HCl**

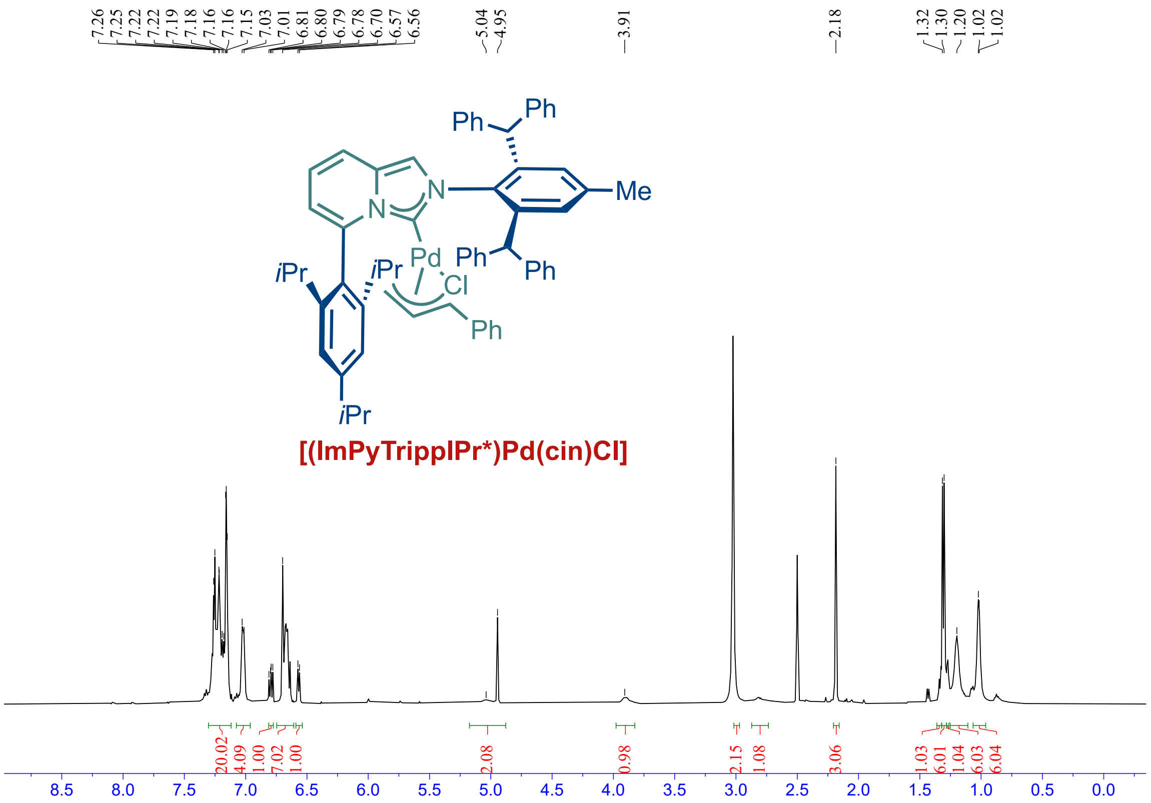

^{1}H NMR (500 MHz, $CDCl_3$) Spectrum of **[(ImPyTrippIPr*)Pd(cin)Cl]**

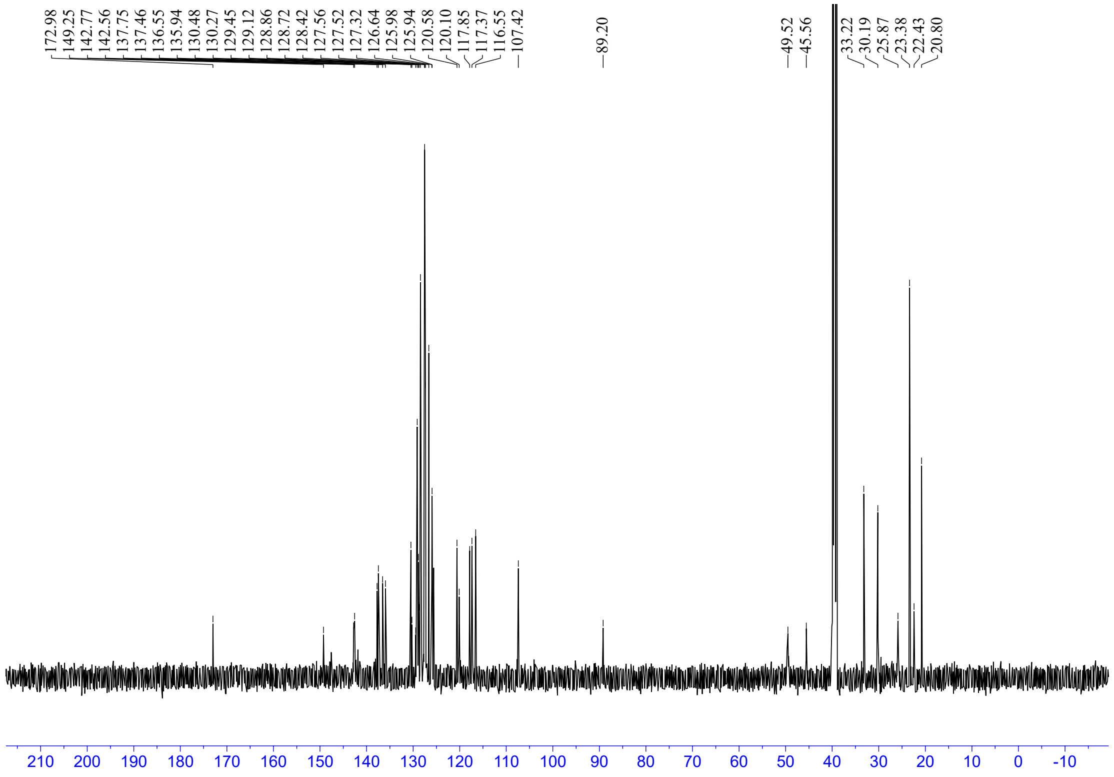

$^{13}C\{^{1}H\}$ NMR (125 MHz, $CDCl_3$) Spectrum of **[(ImPyTrippIPr*)Pd(cin)Cl]**

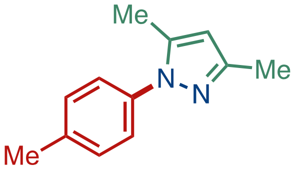

3a

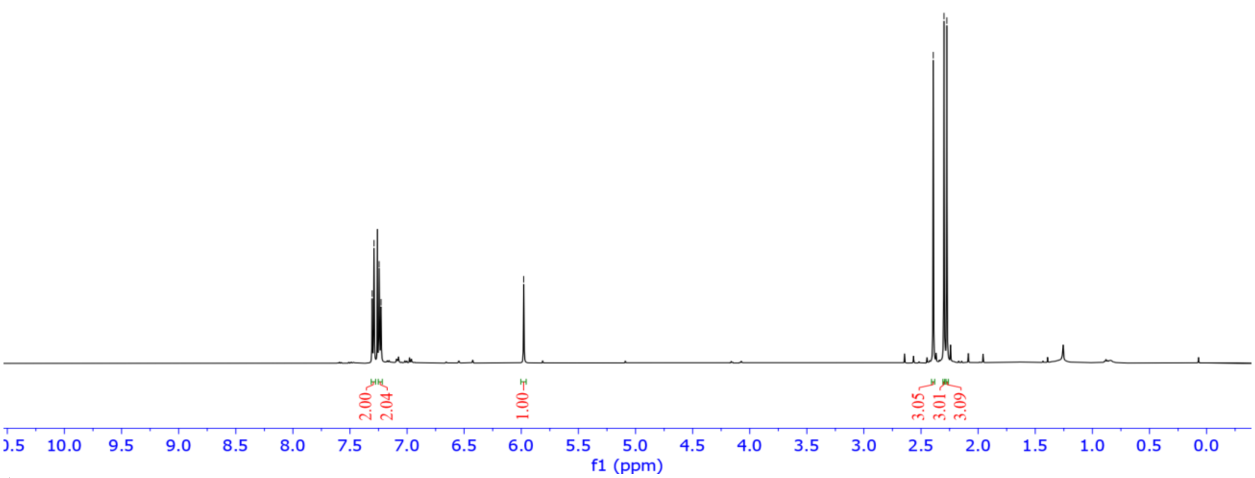

^{1}H NMR (500 MHz, $CDCl_3$) Spectrum of 3,5-Dimethyl-1-(*p*-tolyl)-1*H*-pyrazole (**3a**)

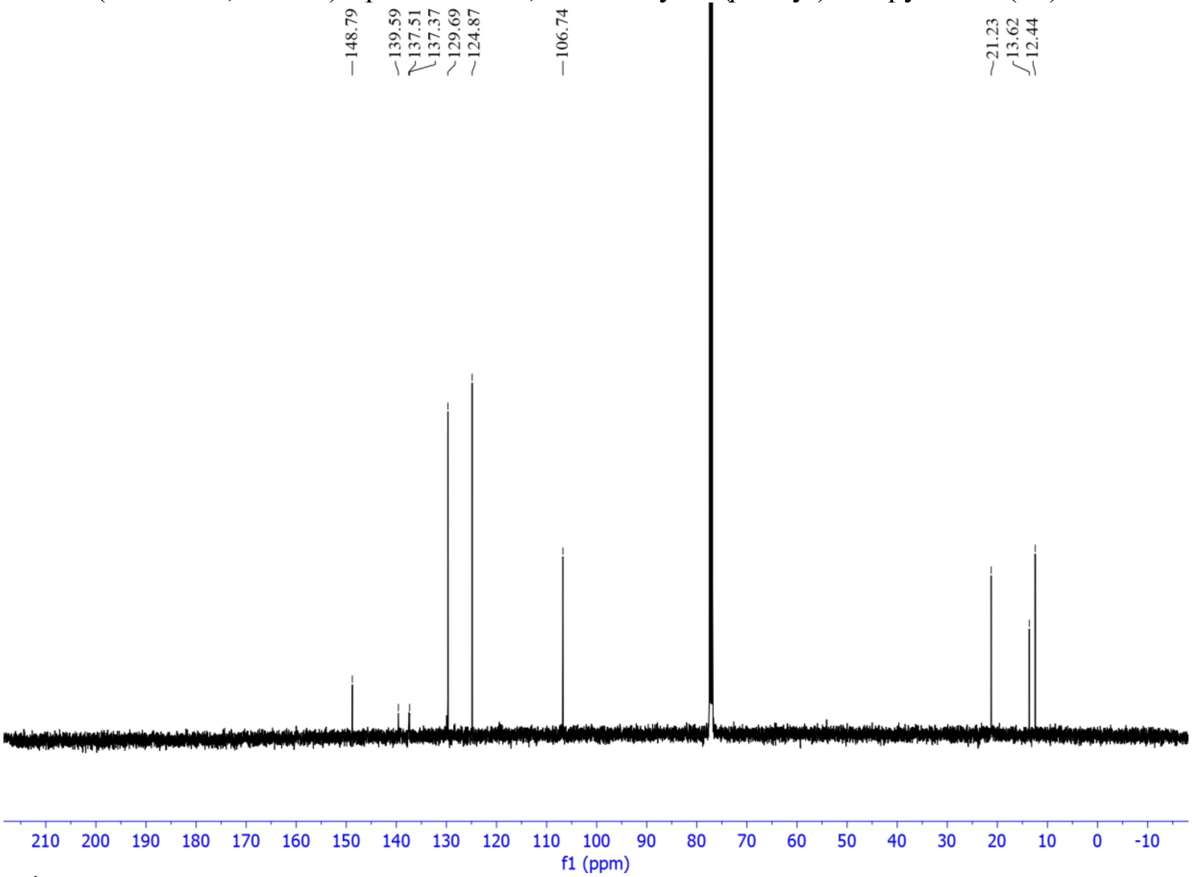

^{13}C{^{1}H} (125 MHz, $CDCl_3$) Spectrum of 3,5-Dimethyl-1-(*p*-tolyl)-1*H*-pyrazole (**3a**)

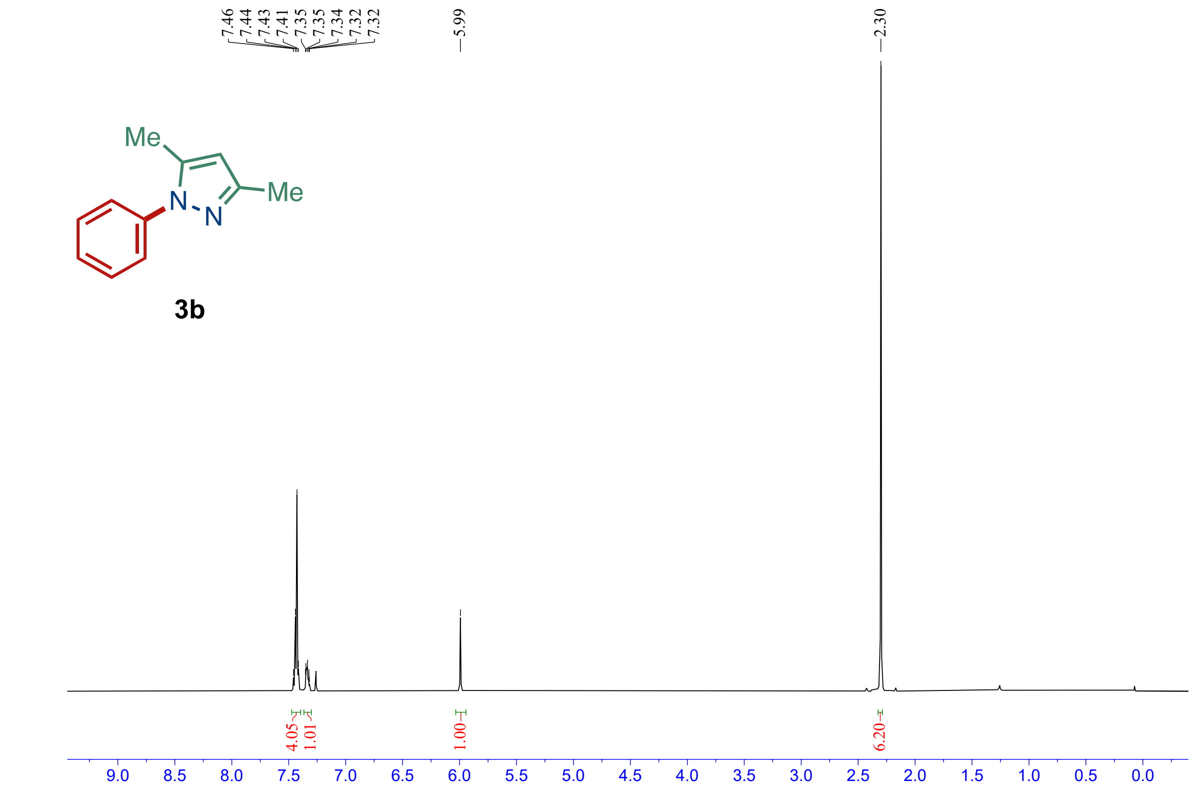

^{1}H NMR (500 MHz, $CDCl_3$) Spectrum of 3,5-Dimethyl-1-phenyl-1*H*-pyrazole (**3b**)

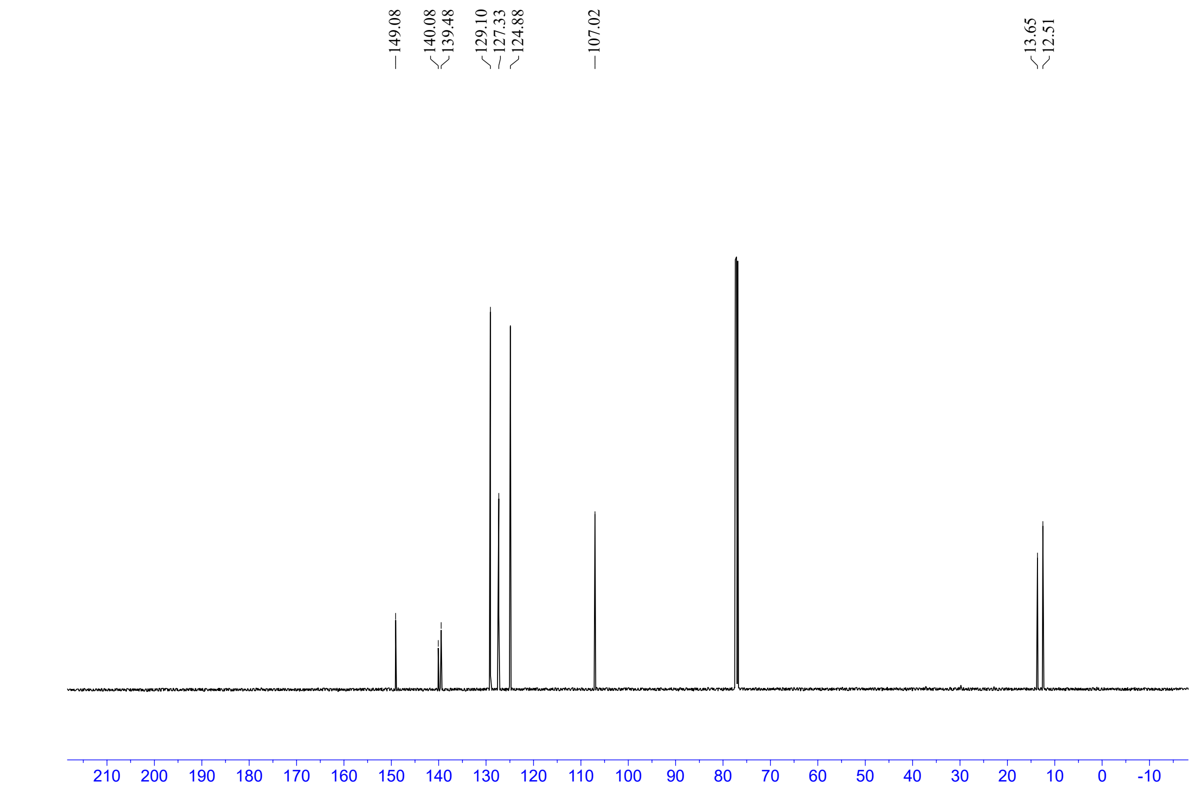

^{13}C{^{1}H} (125 MHz, $CDCl_3$) Spectrum of 3,5-Dimethyl-1-pheny-1*H*-pyrazole (**3b**)

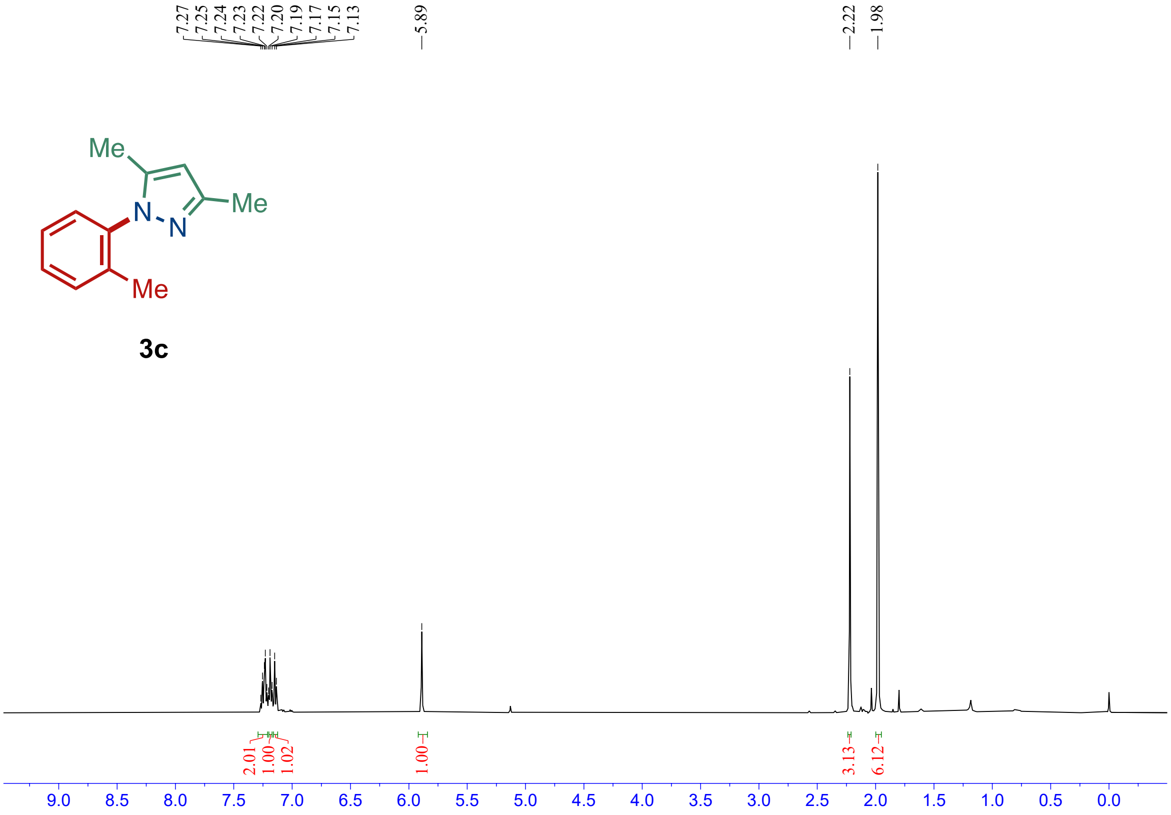

^{1}H NMR (500 MHz, $CDCl_3$) Spectrum of 3,5-Dimethyl-1-(*o*-toyl)-1*H*-pyrazole (**3c**)

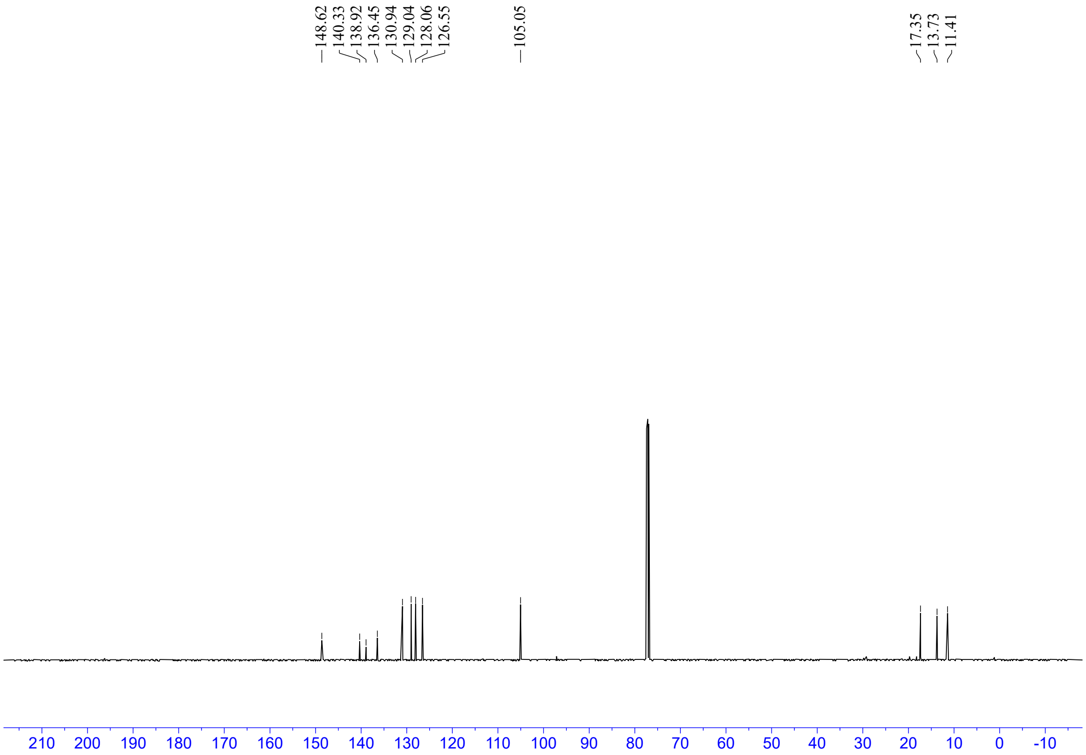

$^{13}C\{^1H\}$ (125 MHz, $CDCl_3$) Spectrum of 3,5-Dimethyl-1-(*o*-toyl)-1*H*-pyrazole (**3c**)

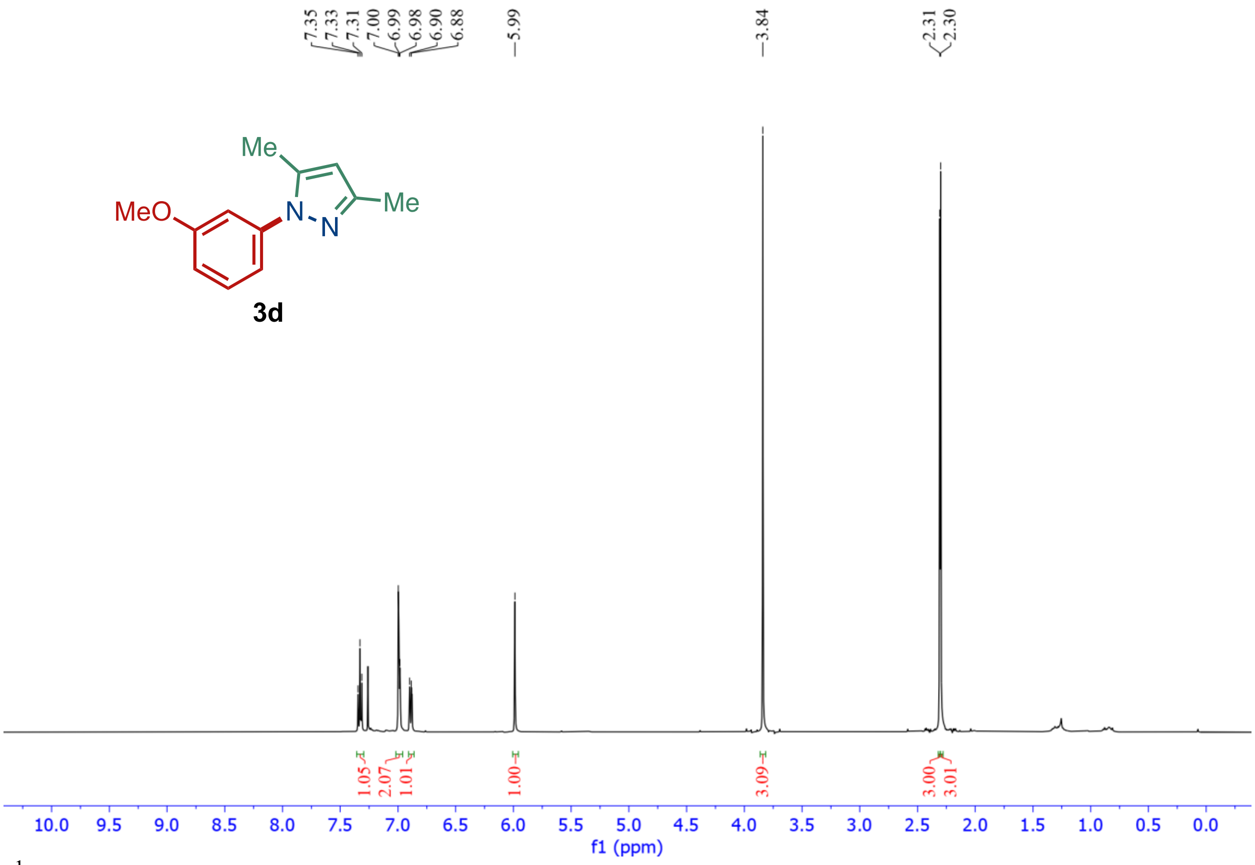

^{1}H NMR (500 MHz, $CDCl_3$) Spectrum of 1-(3-Methoxyphenyl)-3,5-dimethyl-1H-pyrazole (**3d**)

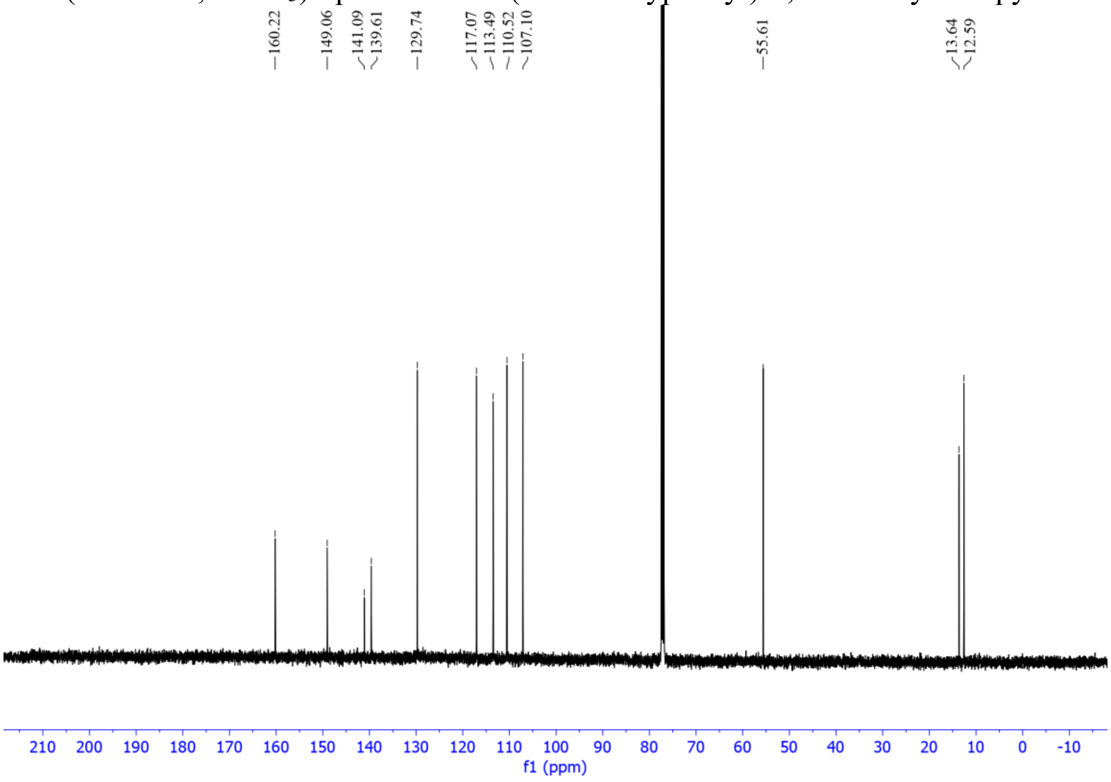

$^{13}C\{^{1}H\}$ (125 MHz, $CDCl_3$) Spectrum of 1-(3-Methoxyphenyl)-3,5-dimethyl-1H-pyrazole (**3d**)

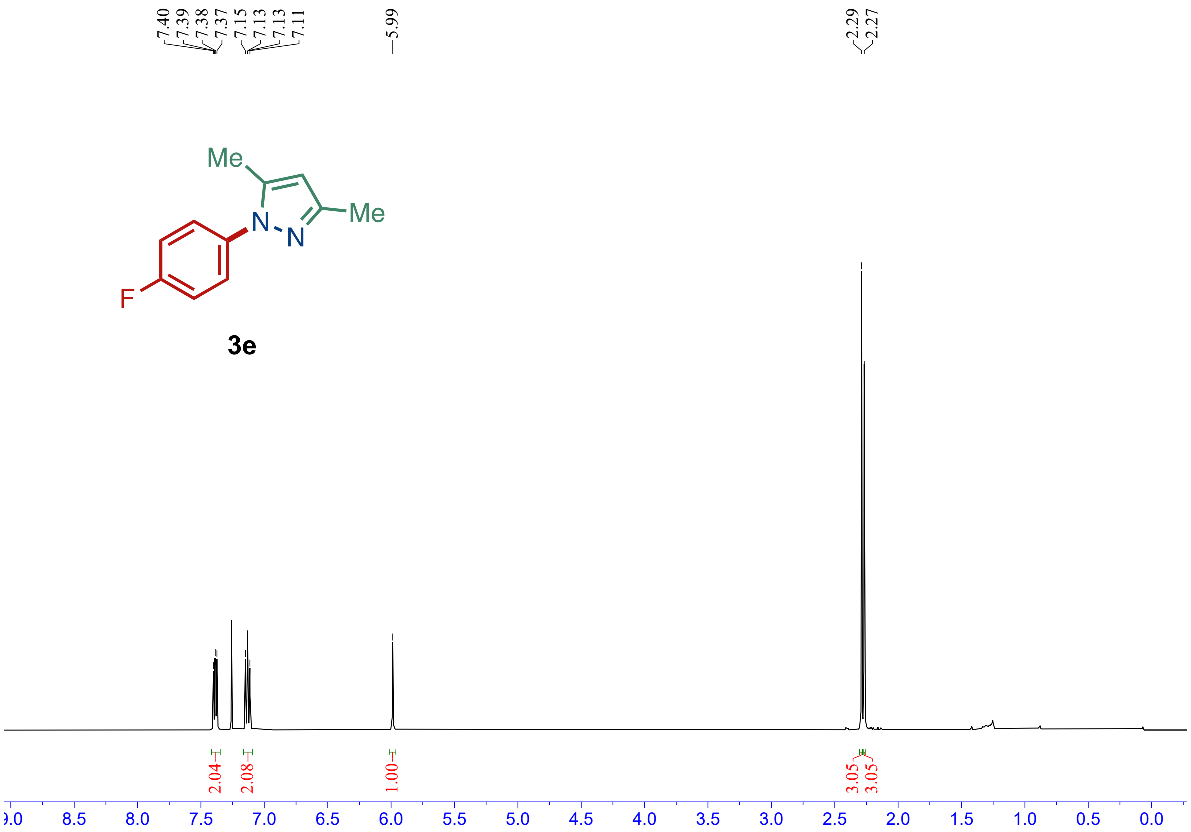

3e

^{1}H NMR (500 MHz, $CDCl_3$) Spectrum of 1-(4-Fluorophenyl)-3,5-dimethyl-1*H*-pyrazole (**3e**)

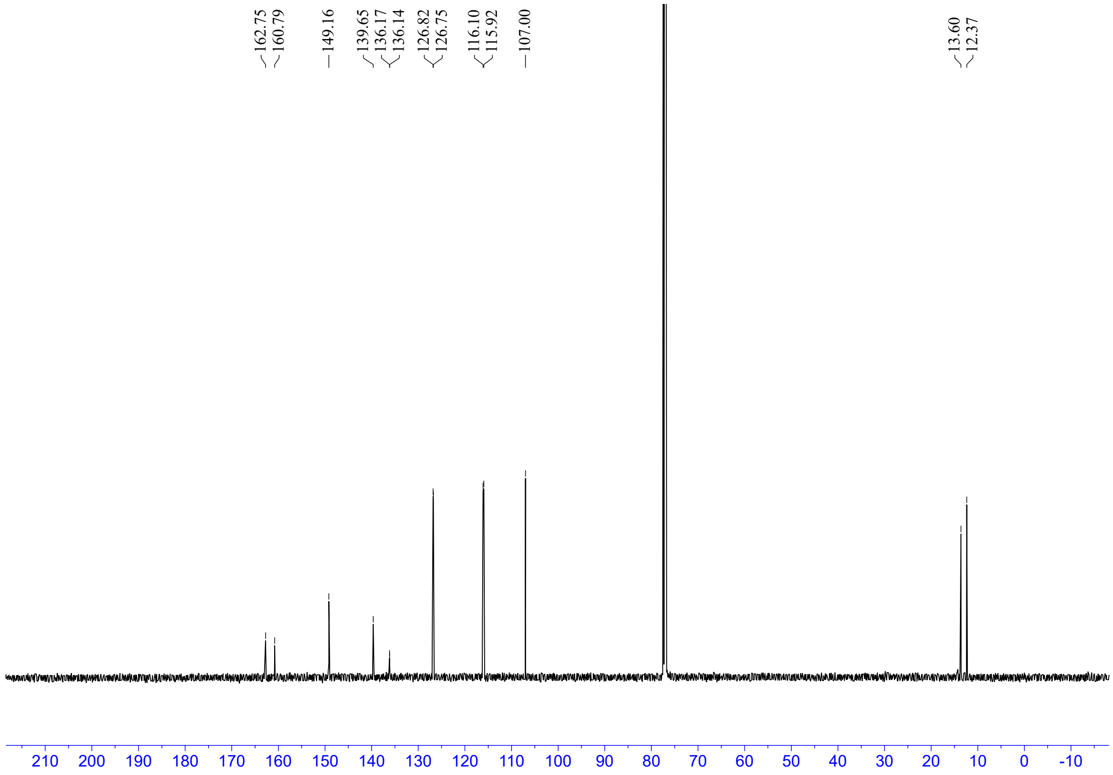

^{13}C{^{1}H} (125 MHz, $CDCl_3$) Spectrum of 1-(4-Fluorophenyl)-3,5-dimethyl-1*H*-pyrazole (**3e**)

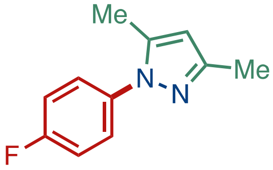

3e

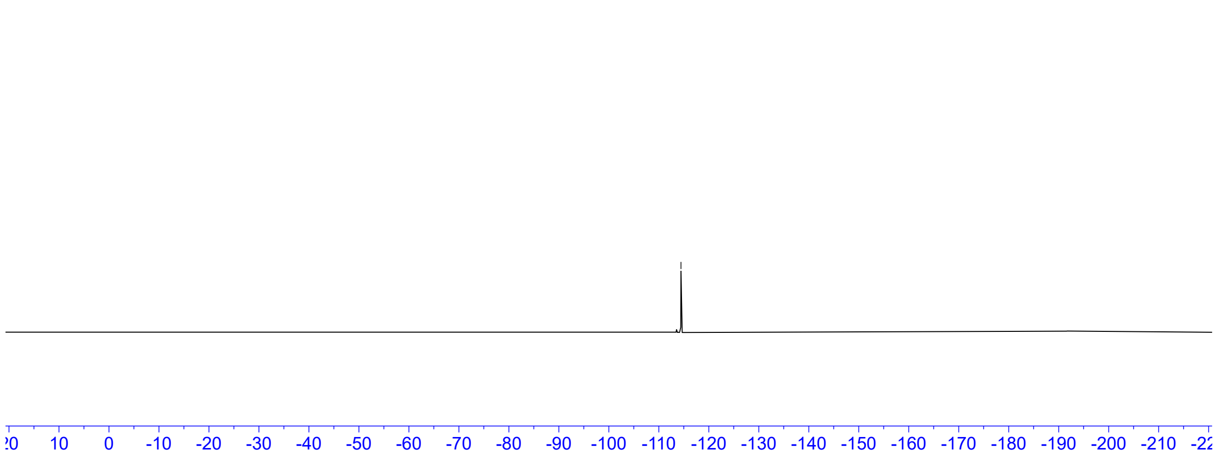

^{19}F NMR (471 MHz, $CDCl_3$) Spectrum of 1-(4-Fluorophenyl)-3,5-dimethyl-1*H*-pyrazole (**3e**)

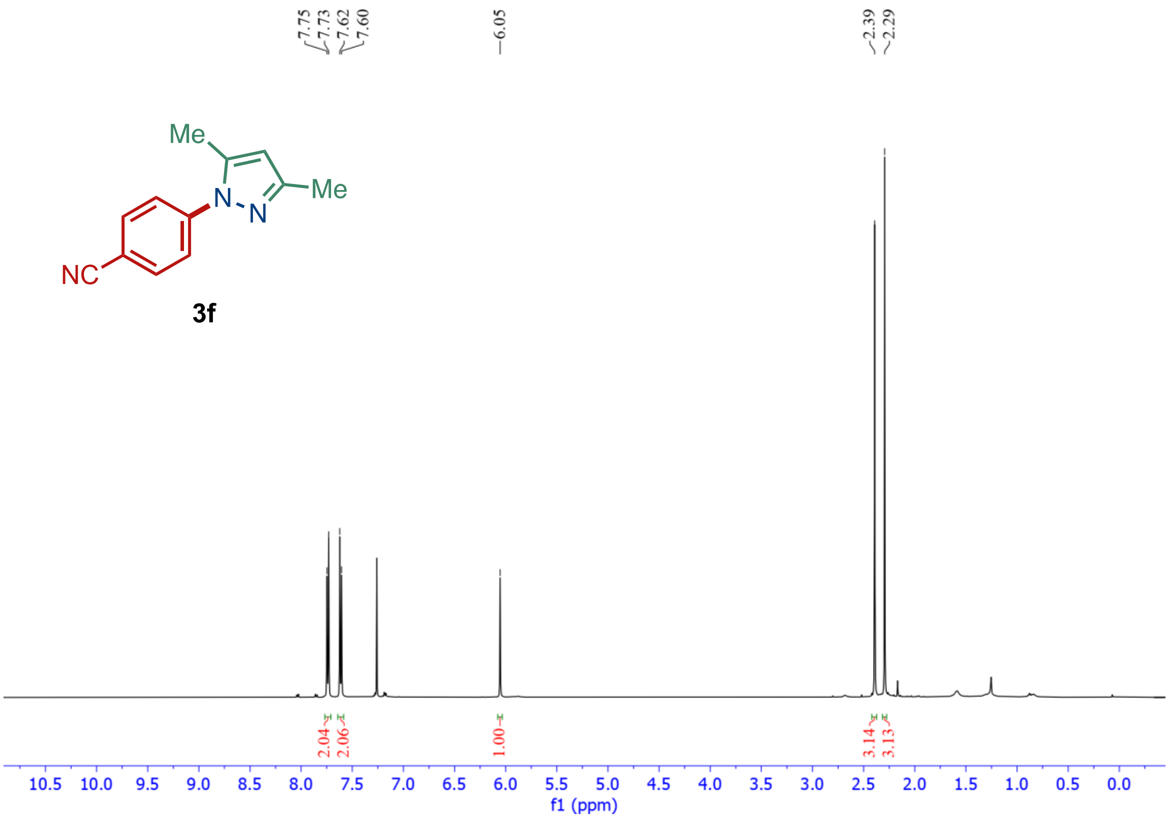

^{1}H NMR (500 MHz, $CDCl_3$) Spectrum of 4-(3,5-Dimethyl-1H-pyrazol-1-yl)benzonitrile (**3f**)

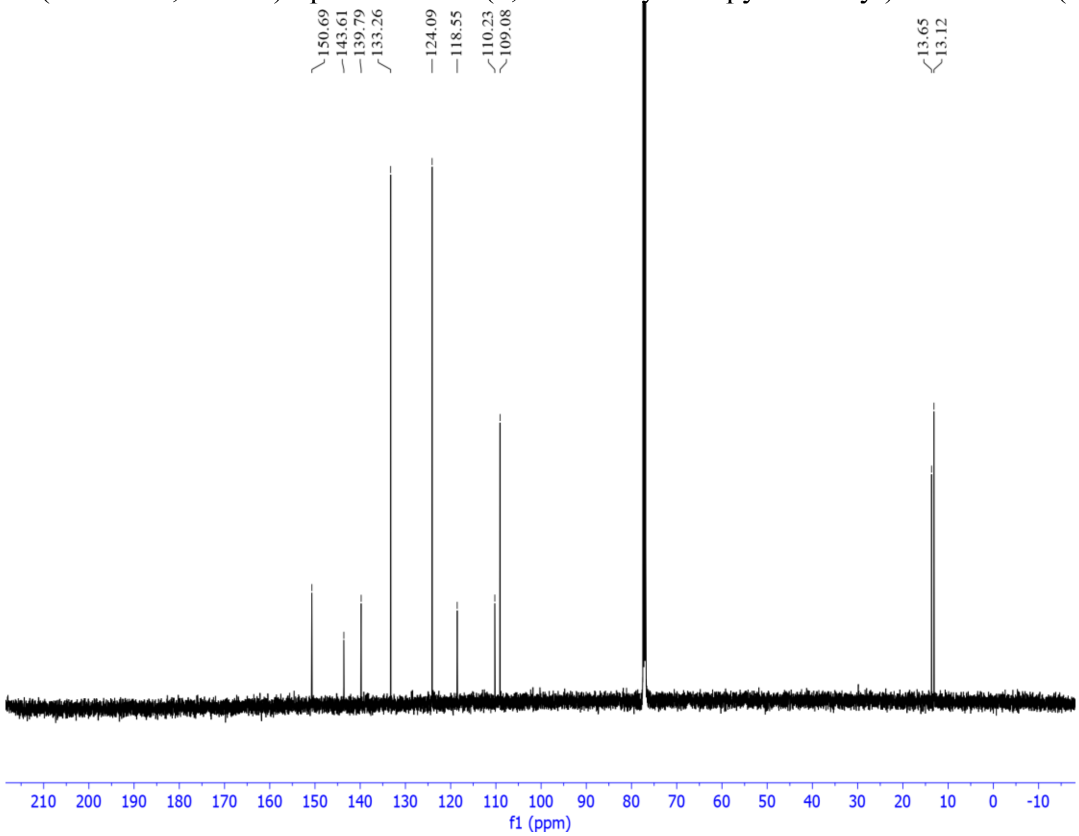

$^{13}C\{^{1}H\}$ (125 MHz, $CDCl_3$) Spectrum of 4-(3,5-Dimethyl-1H-pyrazol-1-yl)benzonitrile (**3f**)

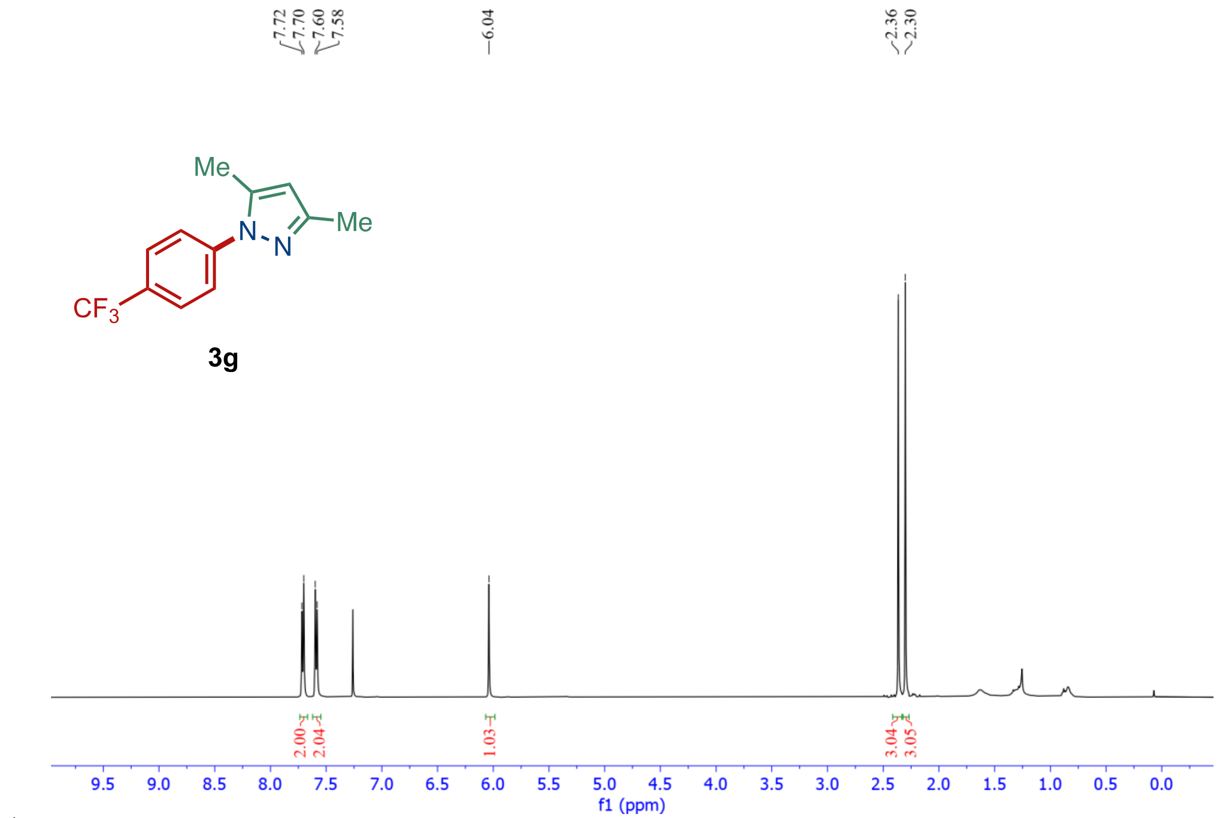

^{1}H NMR (500 MHz, $CDCl_3$) Spectrum of 3,5-Dimethyl-1-(4-(trifluoromethyl)phenyl)-1H-pyrazole (**3g**)

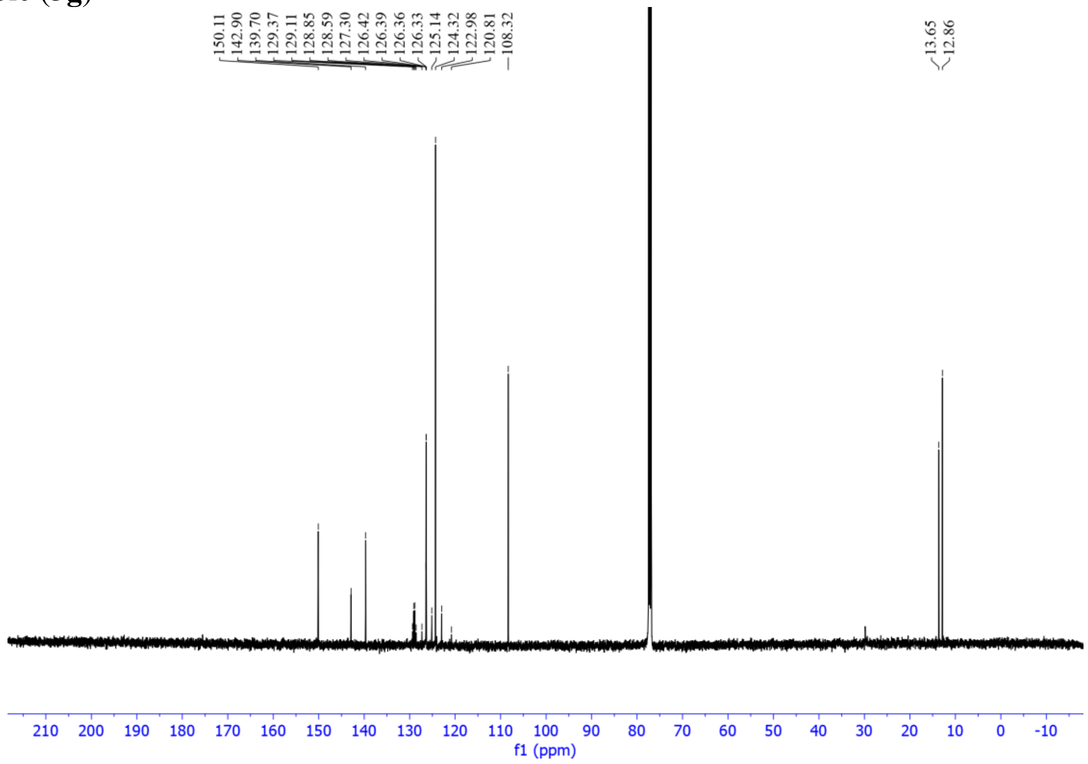

^{13}C{^{1}H} (125 MHz, $CDCl_3$) Spectrum of 3,5-Dimethyl-1-(4-(trifluoromethyl)phenyl)-1H-pyrazole (**3g**)

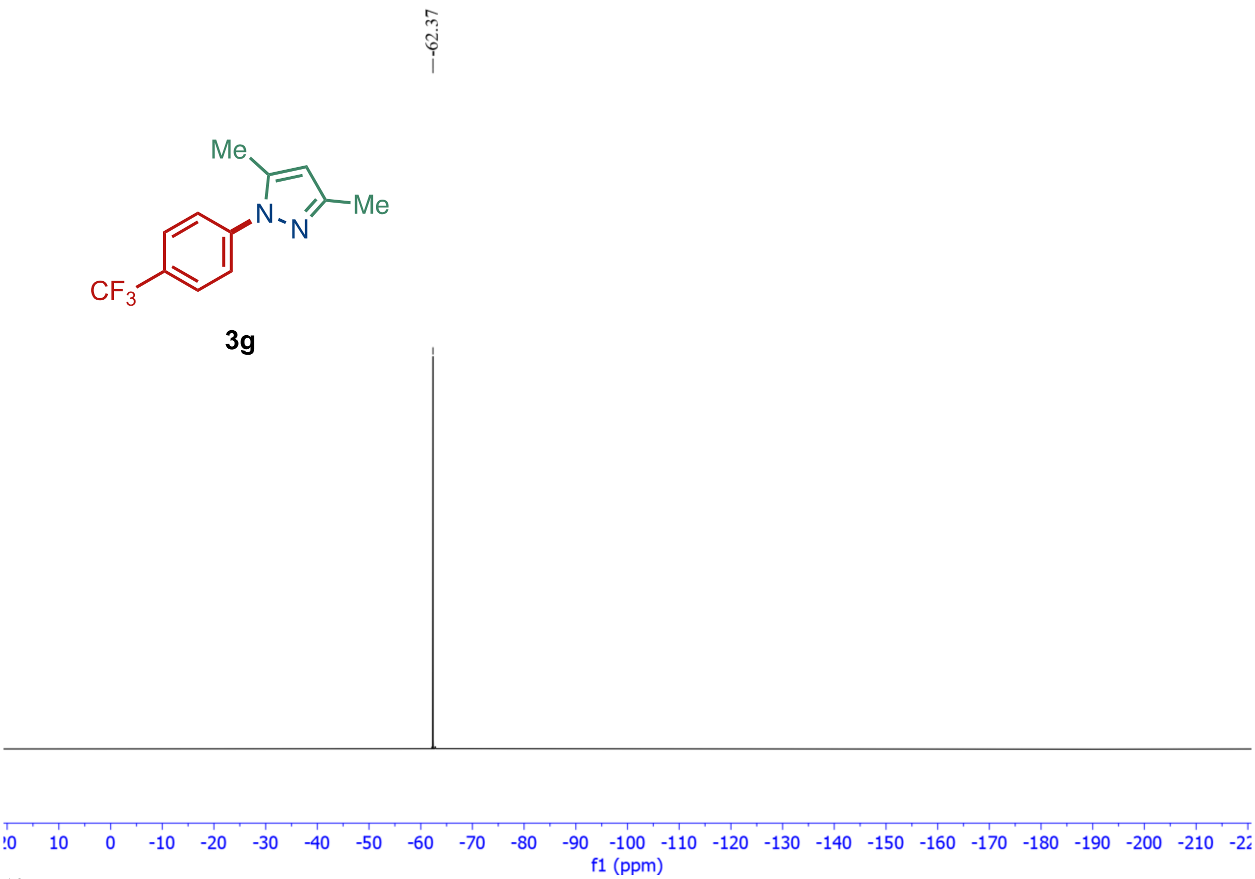

^{19}F NMR (471 MHz, $CDCl_3$) Spectrum of 3,5-Dimethyl-1-(4-(trifluoromethyl)phenyl)-1H-pyrazole (**3g**)

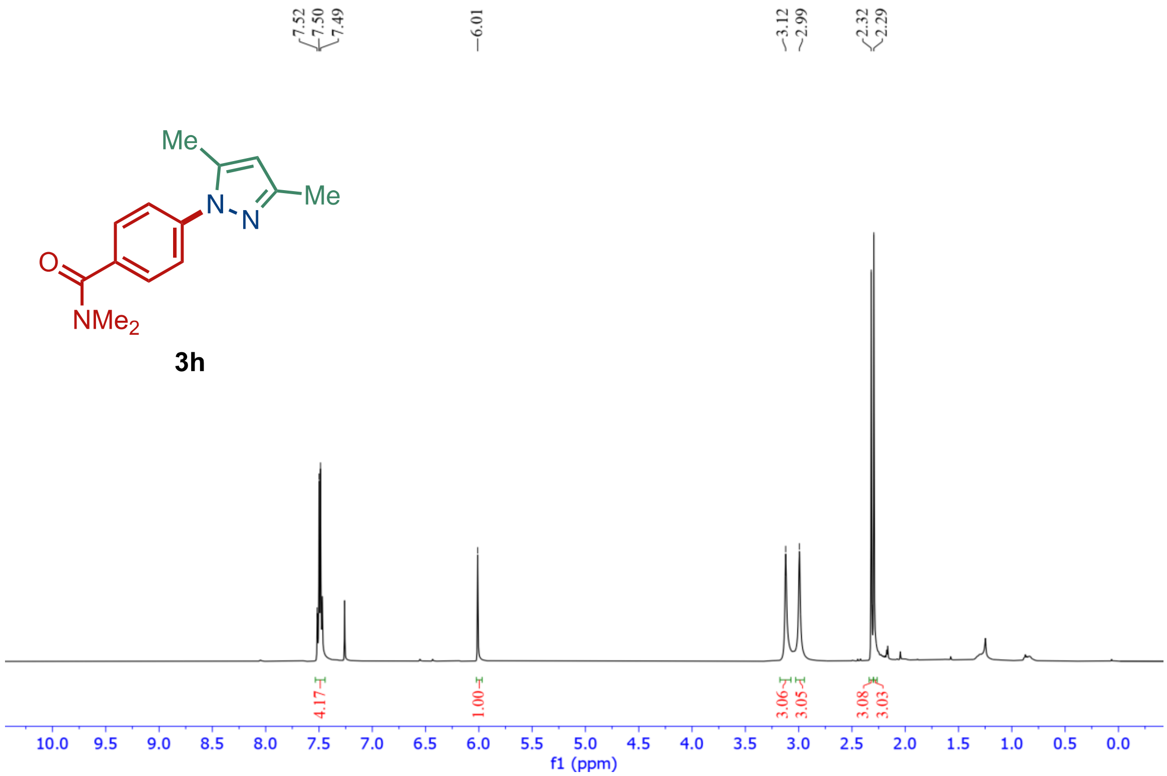

^{1}H NMR (500 MHz, $CDCl_3$) Spectrum of 4-(3,5-Dimethyl-1*H*-pyrazol-1-yl)-*N*,*N*-dimethylbenzamide (**3h**)

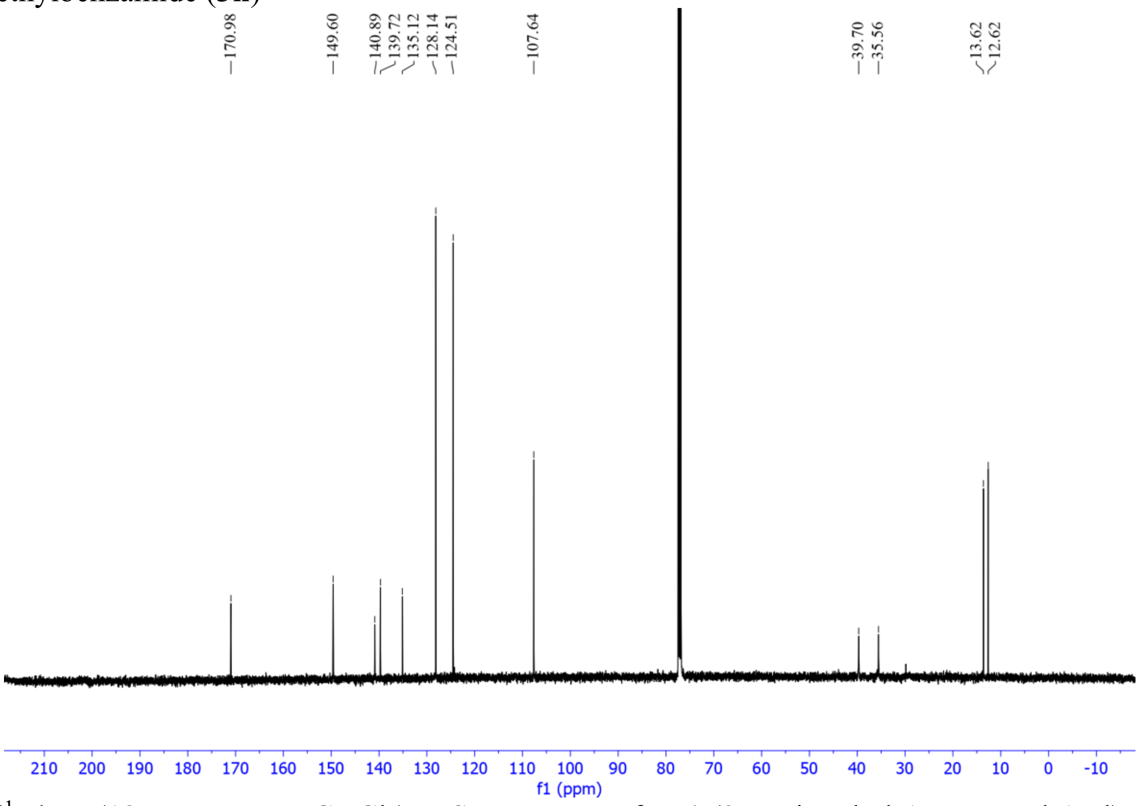

^{13}C{^{1}H} (125 MHz, $CDCl_3$) Spectrum of 4-(3,5-Dimethyl-1*H*-pyrazol-1-yl)-*N*,*N*-dimethylbenzamide (**3h**)

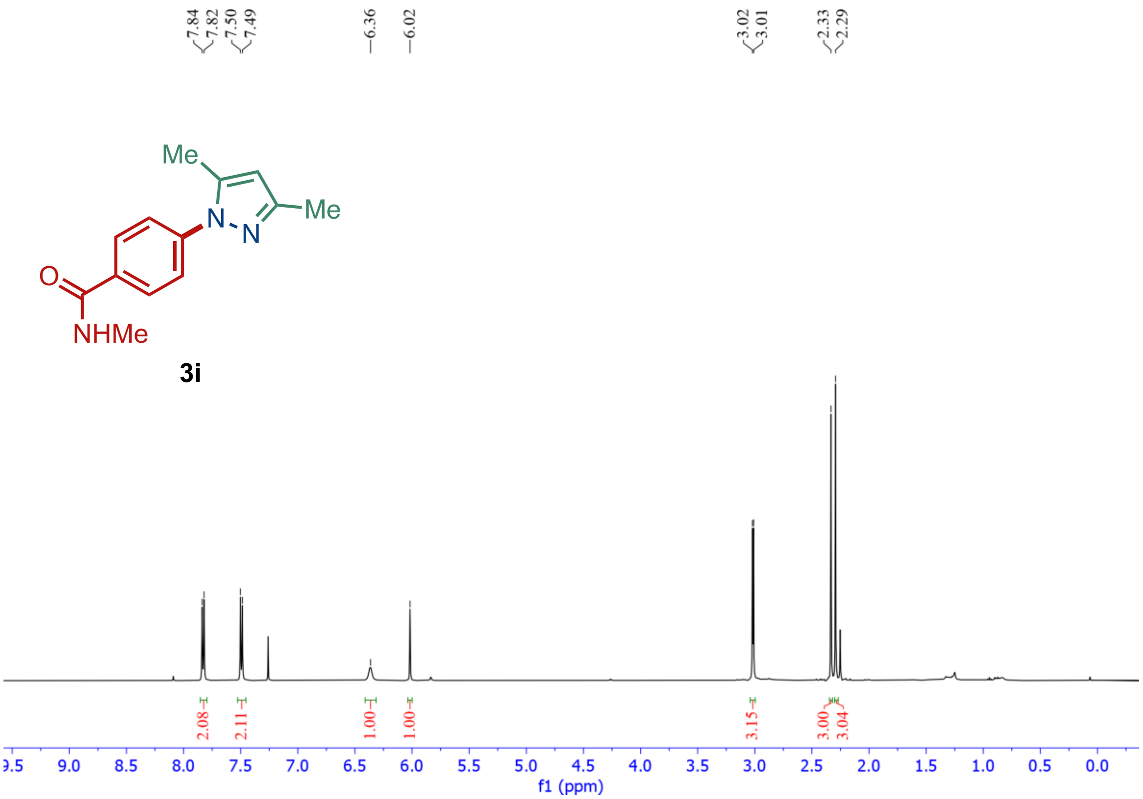

^{1}H NMR (500 MHz, $CDCl_3$) Spectrum of 4-(3,5-Dimethyl-1*H*-pyrazol-1-yl)-*N*-methylbenzamide (**3i**)

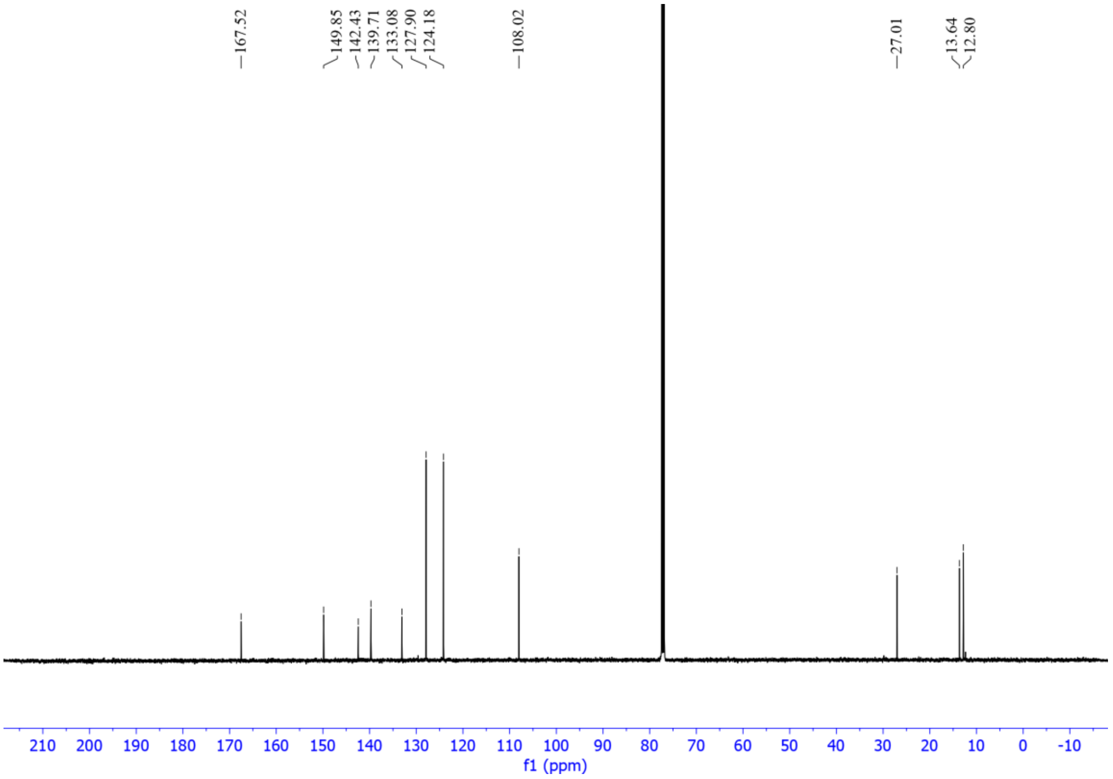

^{13}C{^{1}H} (125 MHz, $CDCl_3$) Spectrum of 4-(3,5-Dimethyl-1*H*-pyrazol-1-yl)-*N*-methylbenzamide (**3i**)

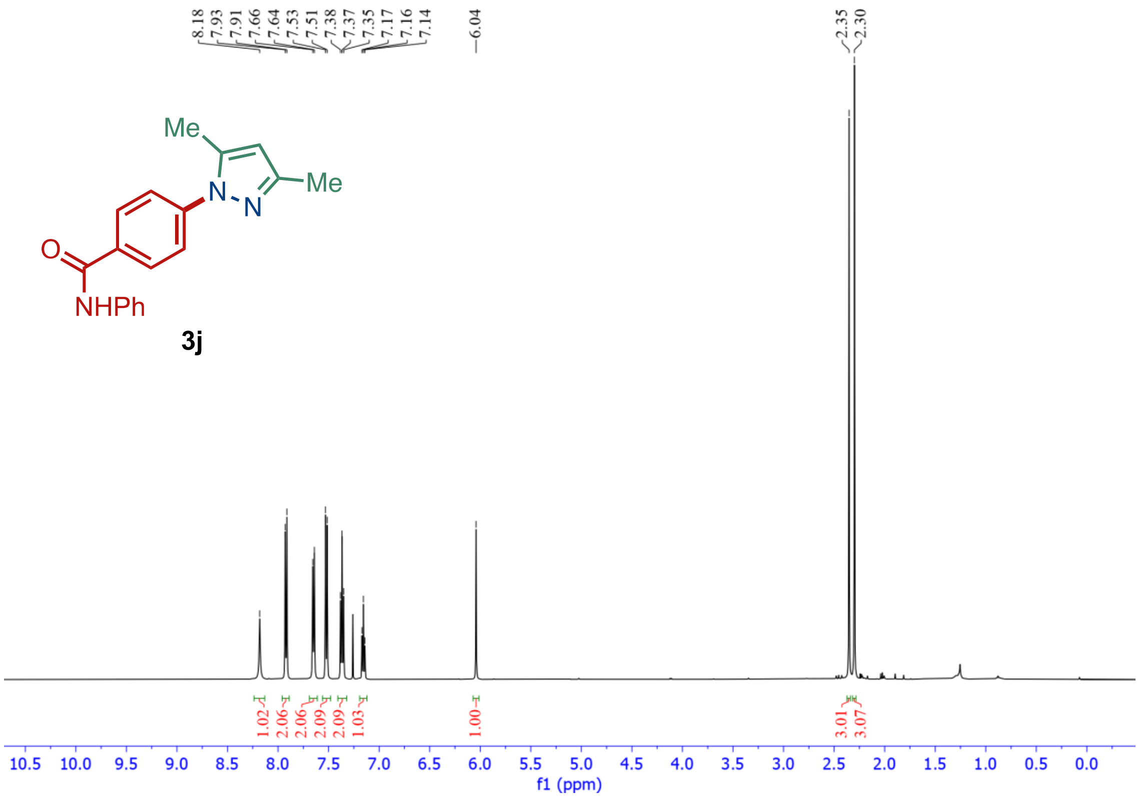

^{1}H NMR (500 MHz, $CDCl_3$) Spectrum of 4-(3,5-Dimethyl-1*H*-pyrazol-1-yl)-*N*-phenylbenzamide (**3j**)

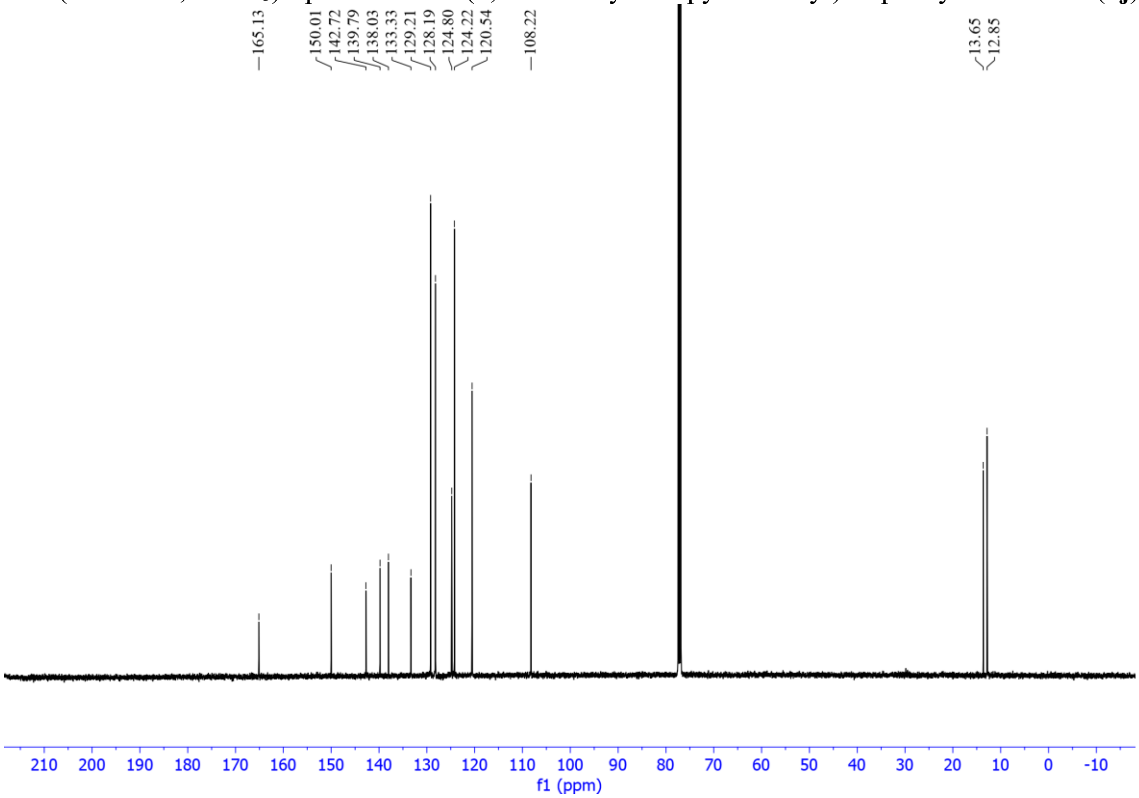

$^{13}C\{^{1}H\}$ (125 MHz, $CDCl_3$) Spectrum of 4-(3,5-Dimethyl-1*H*-pyrazol-1-yl)-*N*-phenylbenzamide (**3j**)

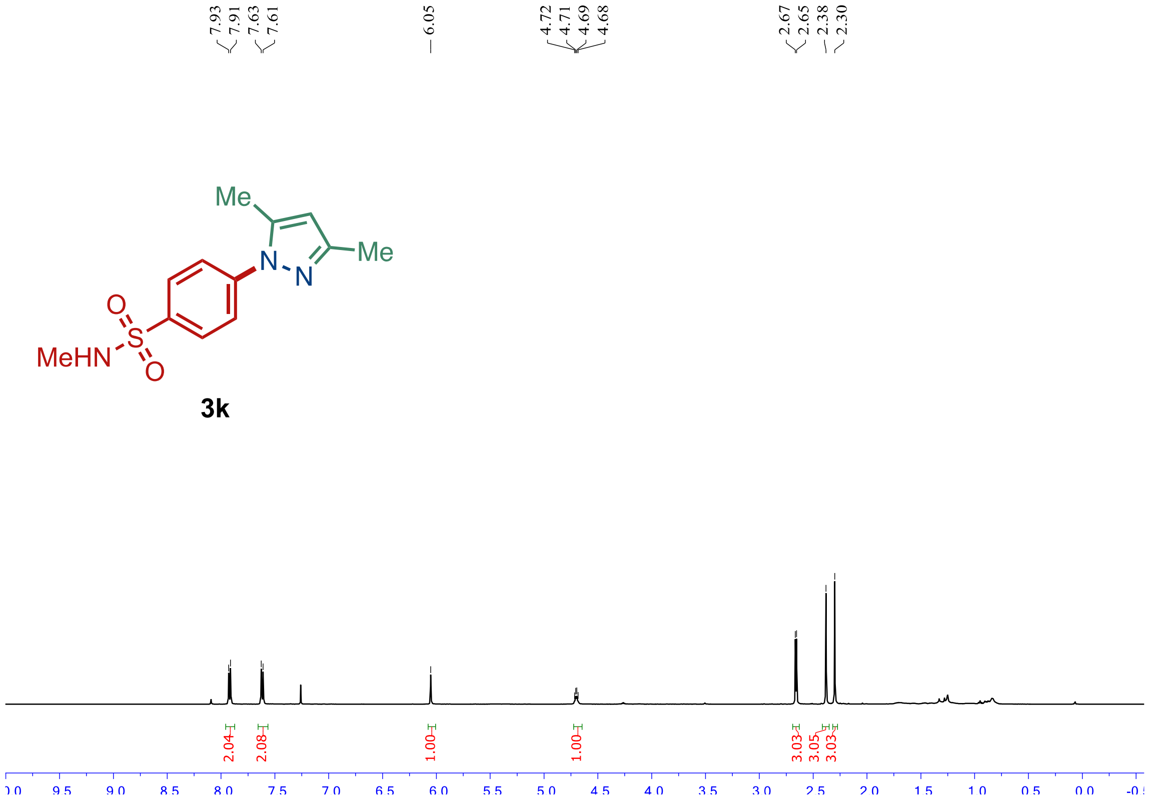

^{1}H NMR (500 MHz, $CDCl_3$) Spectrum of 4-(3,5-Dimethyl-1*H*-pyrazol-1-yl)-*N*-methylbenzenesulfonamide (**3k**)

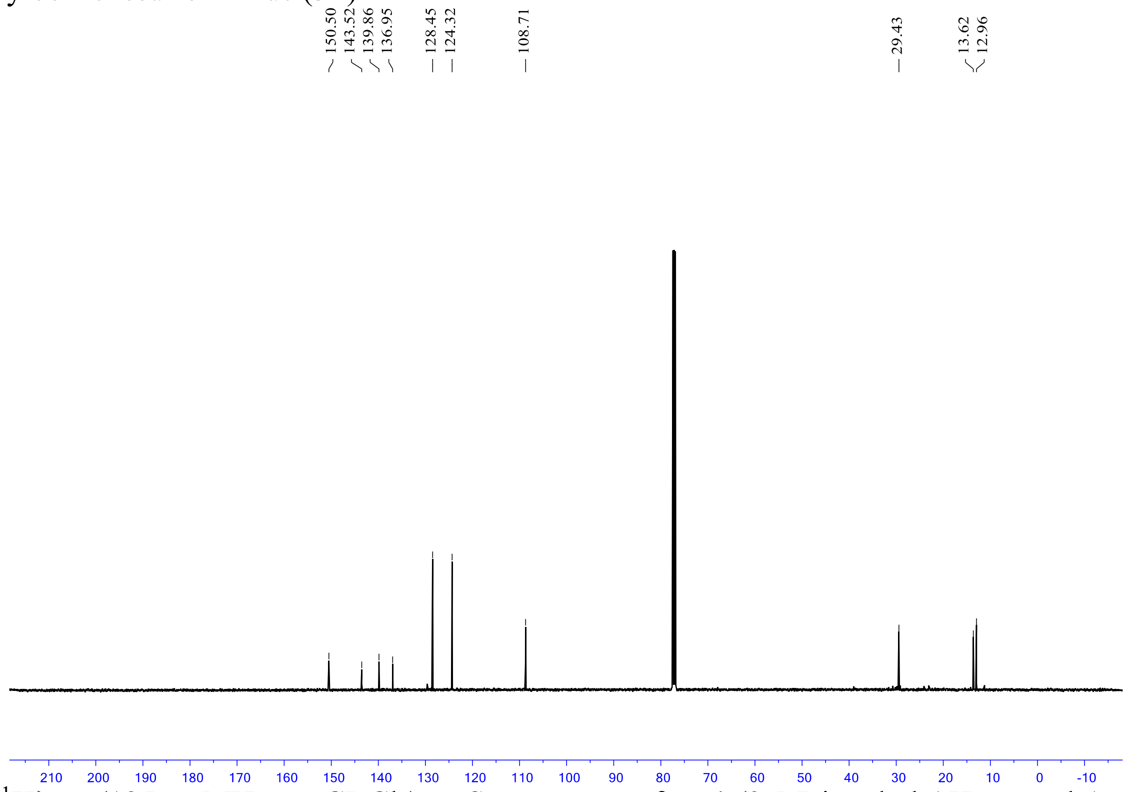

^{13}C{^{1}H} (125 MHz, $CDCl_3$) Spectrum of 4-(3,5-Dimethyl-1*H*-pyrazol-1-yl)-*N*-methylbenzenesulfonamide (**3k**)

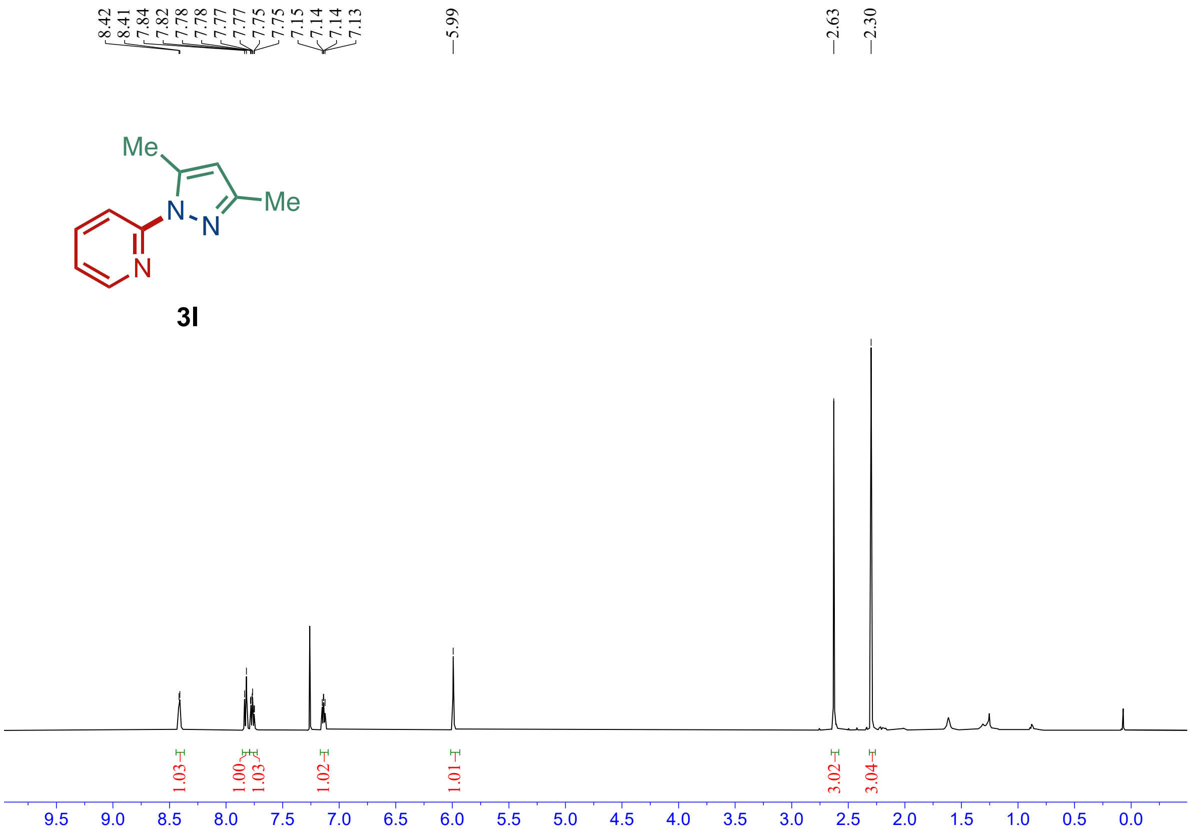

^{1}H NMR (500 MHz, $CDCl_3$) Spectrum of 2-(3,5-Dimethyl-1*H*-pyrazol-1-yl)pyridine (**3l**)

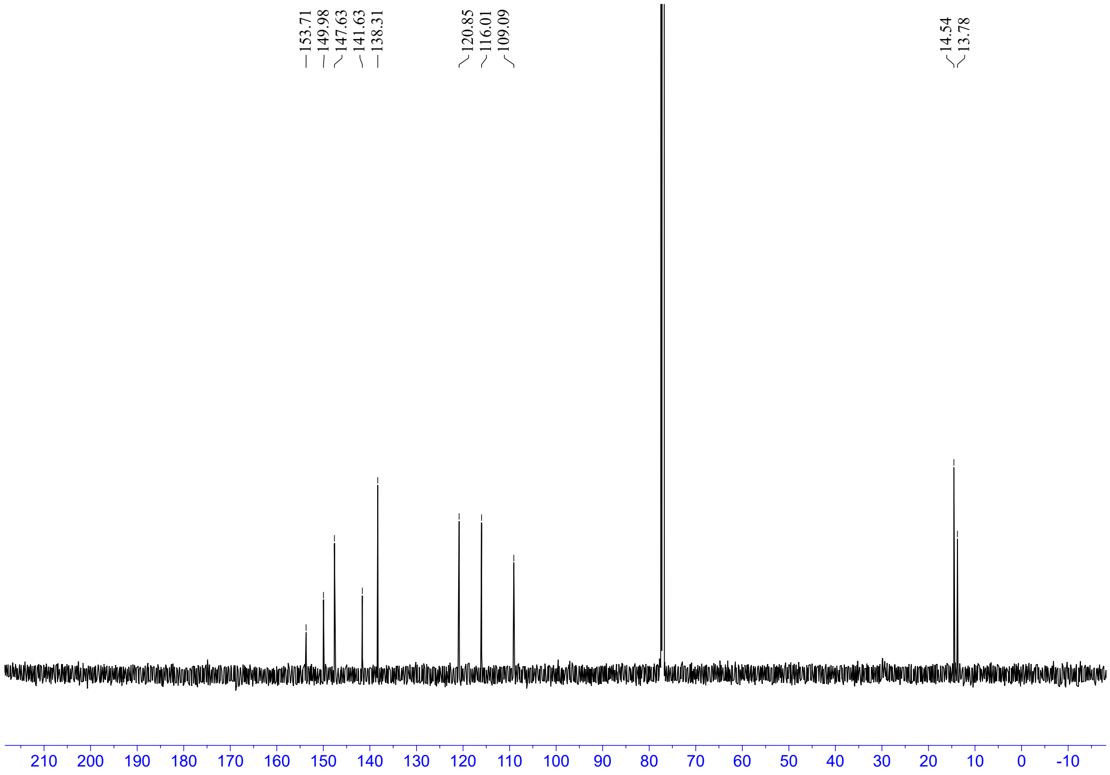

^{13}C{^{1}H} (125 MHz, $CDCl_3$) Spectrum of 2-(3,5-Dimethyl-1*H*-pyrazol-1-yl)pyridine (**3l**)

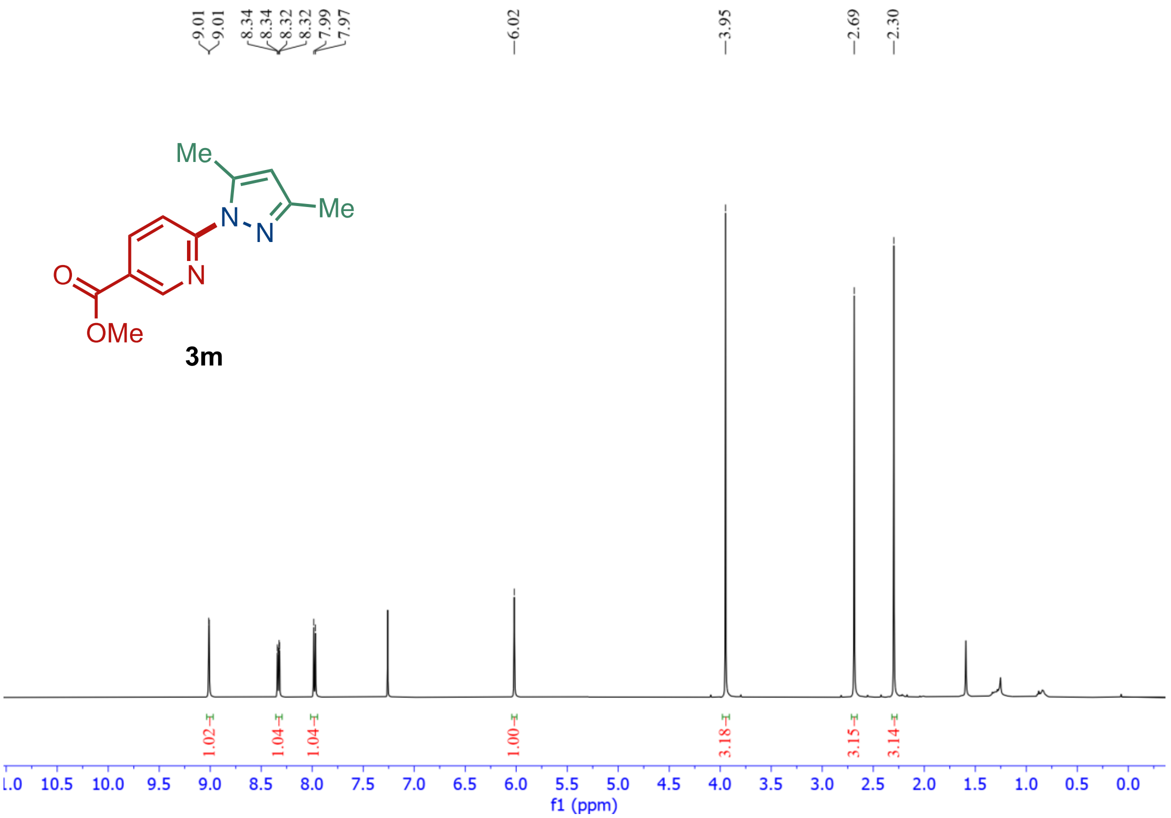

^{1}H NMR (500 MHz, $CDCl_3$) Spectrum of Methyl 6-(3,5-dimethyl-1*H*-pyrazol-1-yl)nicotinate (**3m**)

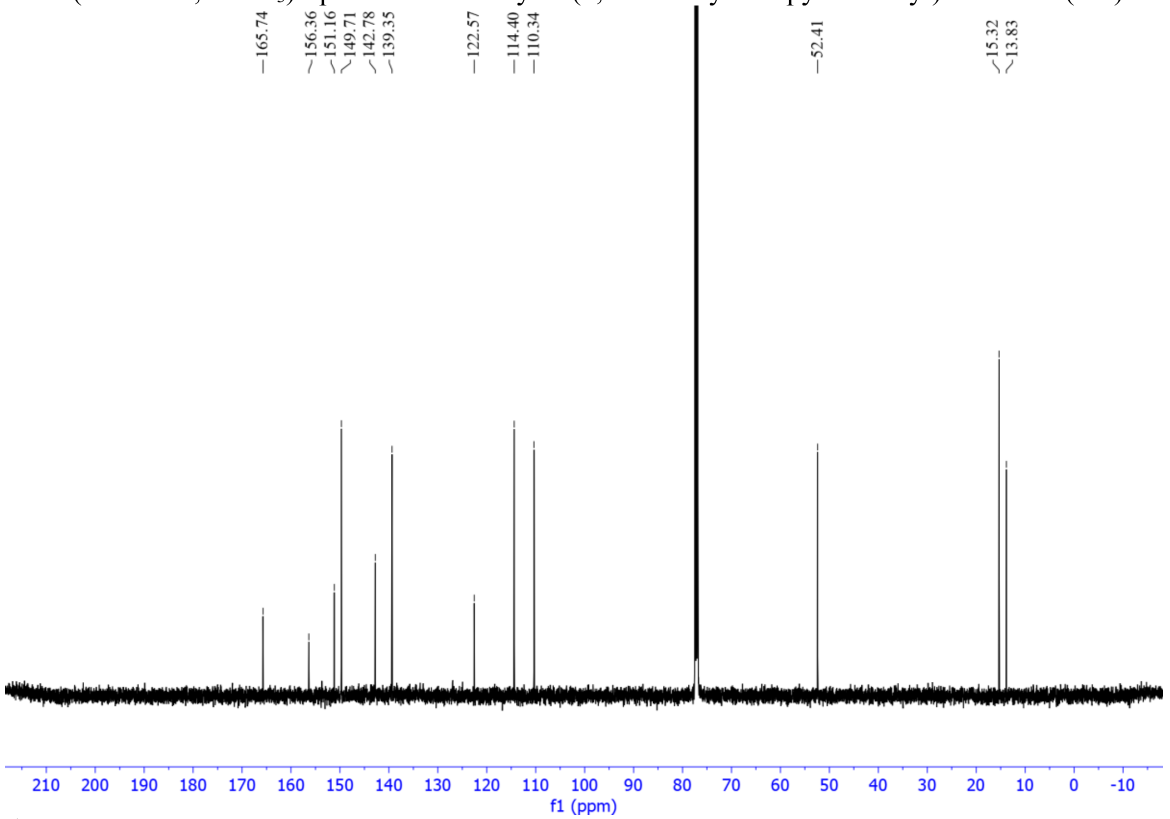

^{13}C{^{1}H} (125 MHz, $CDCl_3$) Spectrum of Methyl 6-(3,5-dimethyl-1*H*-pyrazol-1-yl)nicotinate (**3m**)

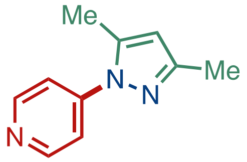

3n

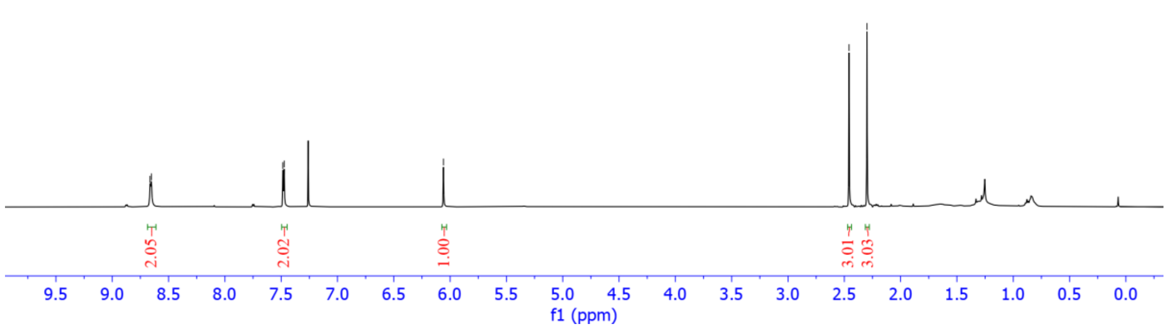

^{1}H NMR (500 MHz, $CDCl_3$) Spectrum of 4-(3,5-Dimethyl-1*H*-pyrazol-1-yl)pyridine (**3n**)

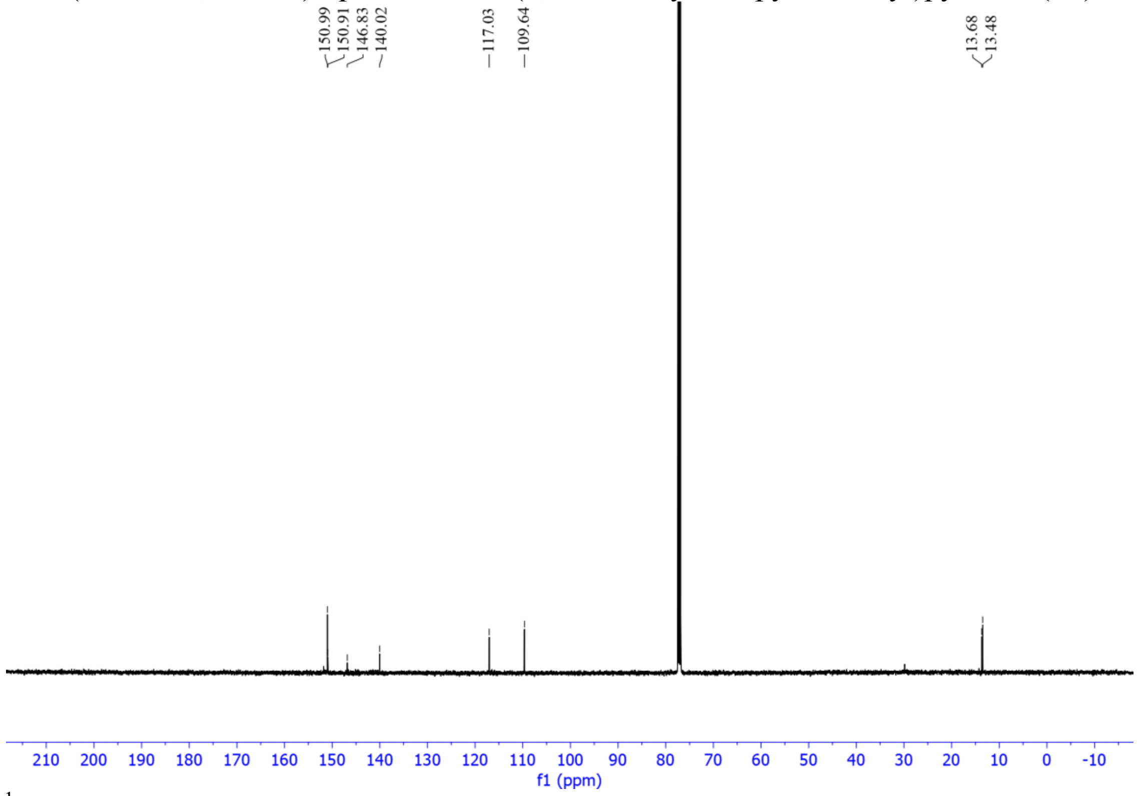

^{13}C{^{1}H} (125 MHz, $CDCl_3$) Spectrum of Spectrum of 4-(3,5-Dimethyl-1*H*-pyrazol-1-yl)pyridine (**3n**)

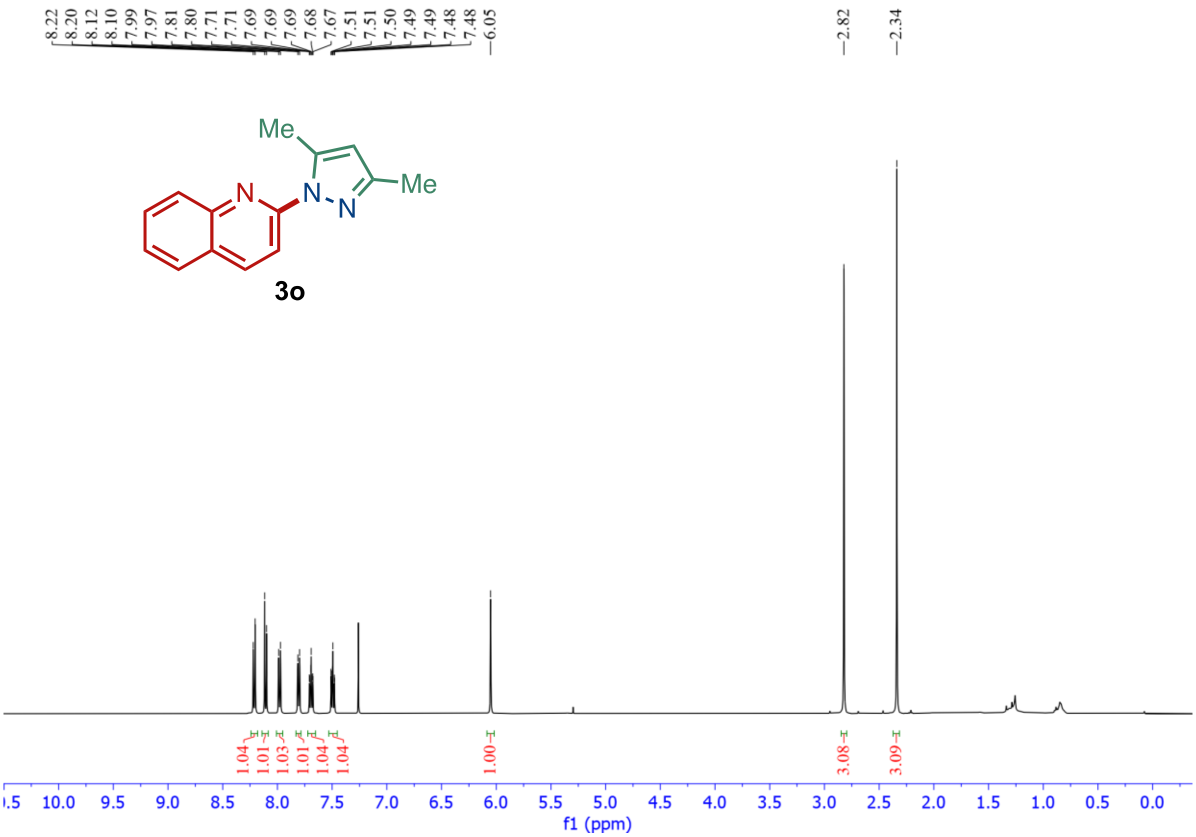

^{1}H NMR (500 MHz, CDCl$_3$) Spectrum of 2-(3,5-Dimethyl-1*H*-pyrazol-1-yl)quinoline (**3o**)

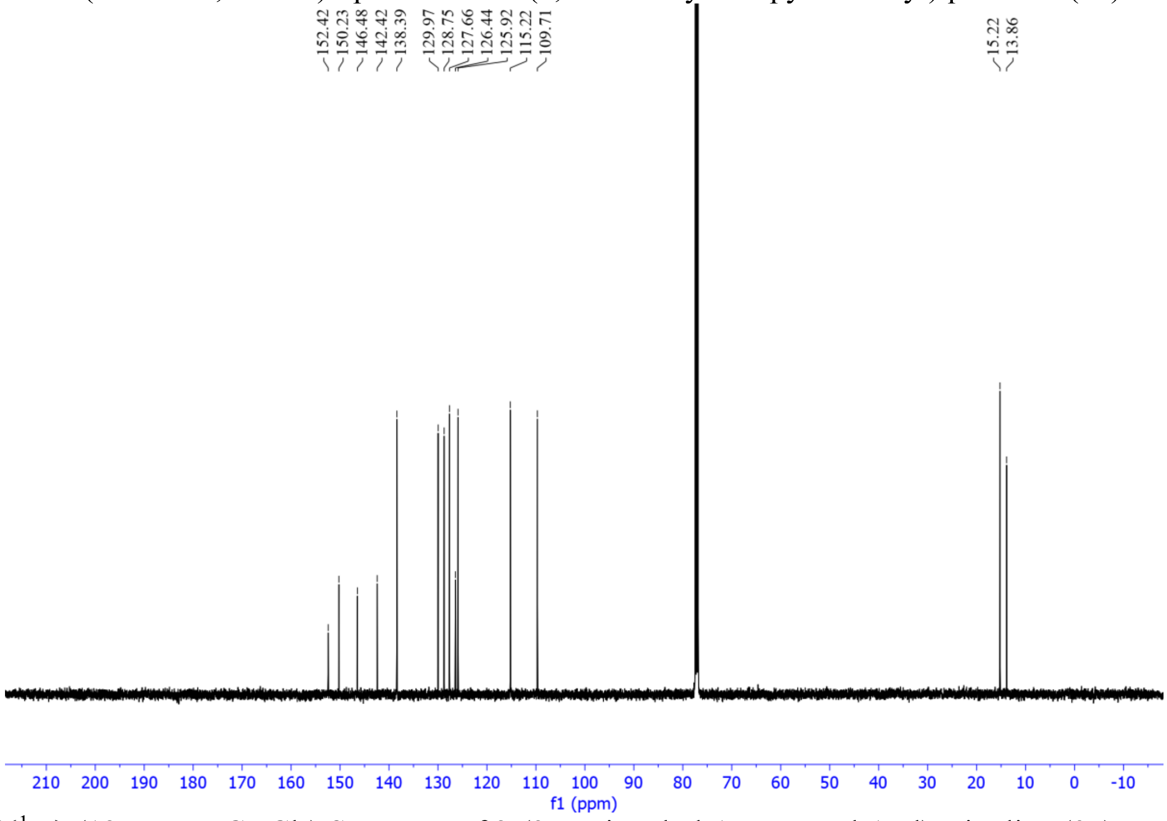

^{13}C{^{1}H} (125 MHz, CDCl$_3$) Spectrum of 2-(3,5-Dimethyl-1*H*-pyrazol-1-yl)quinoline (**3o**)

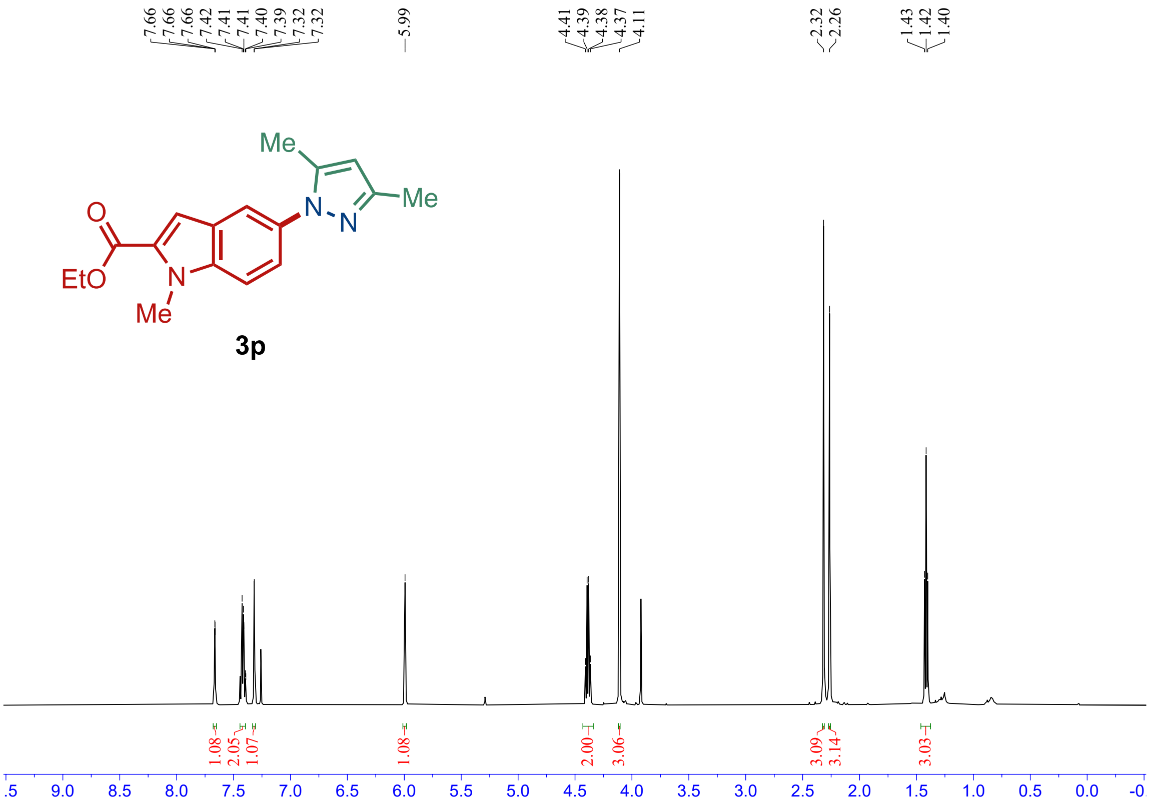

^{1}H NMR (500 MHz, $CDCl_3$) Spectrum of Ethyl 5-(3,5-dimethyl-1*H*-pyrazol-1-yl)-1-methyl-1*H*-indole-2-carboxylate (**3p**)

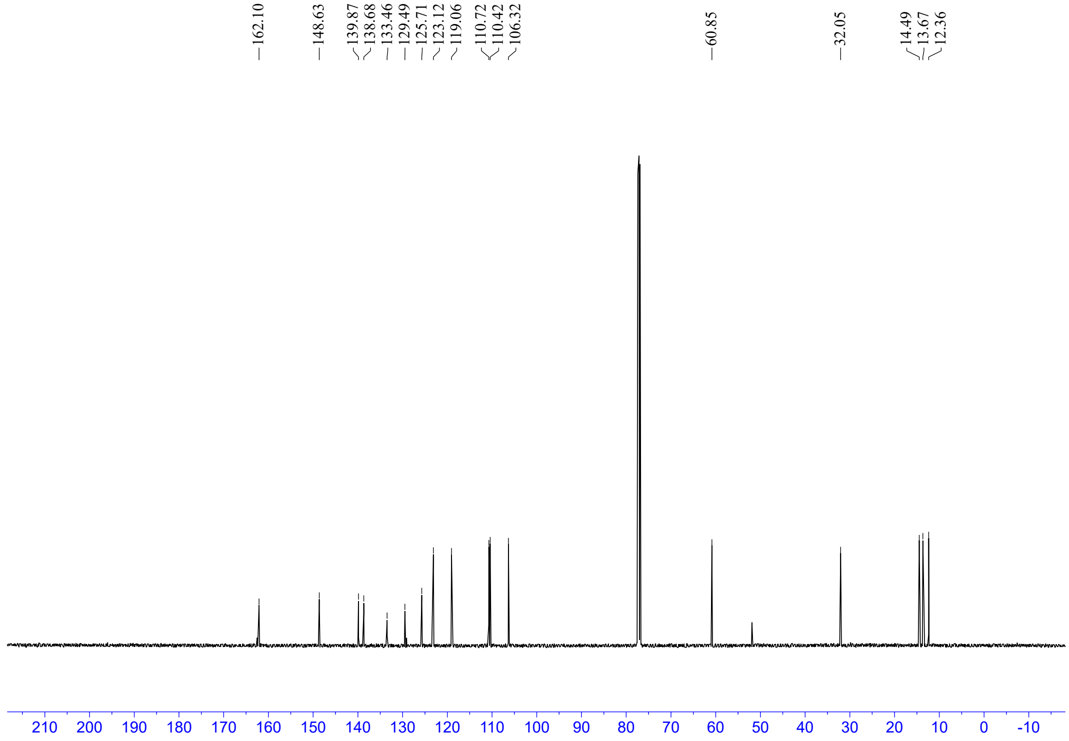

^{13}C{^{1}H} (125 MHz, $CDCl_3$) Spectrum of Ethyl 5-(3,5-dimethyl-1*H*-pyrazol-1-yl)-1-methyl-1*H*-indole-2-carboxylate (**3p**)

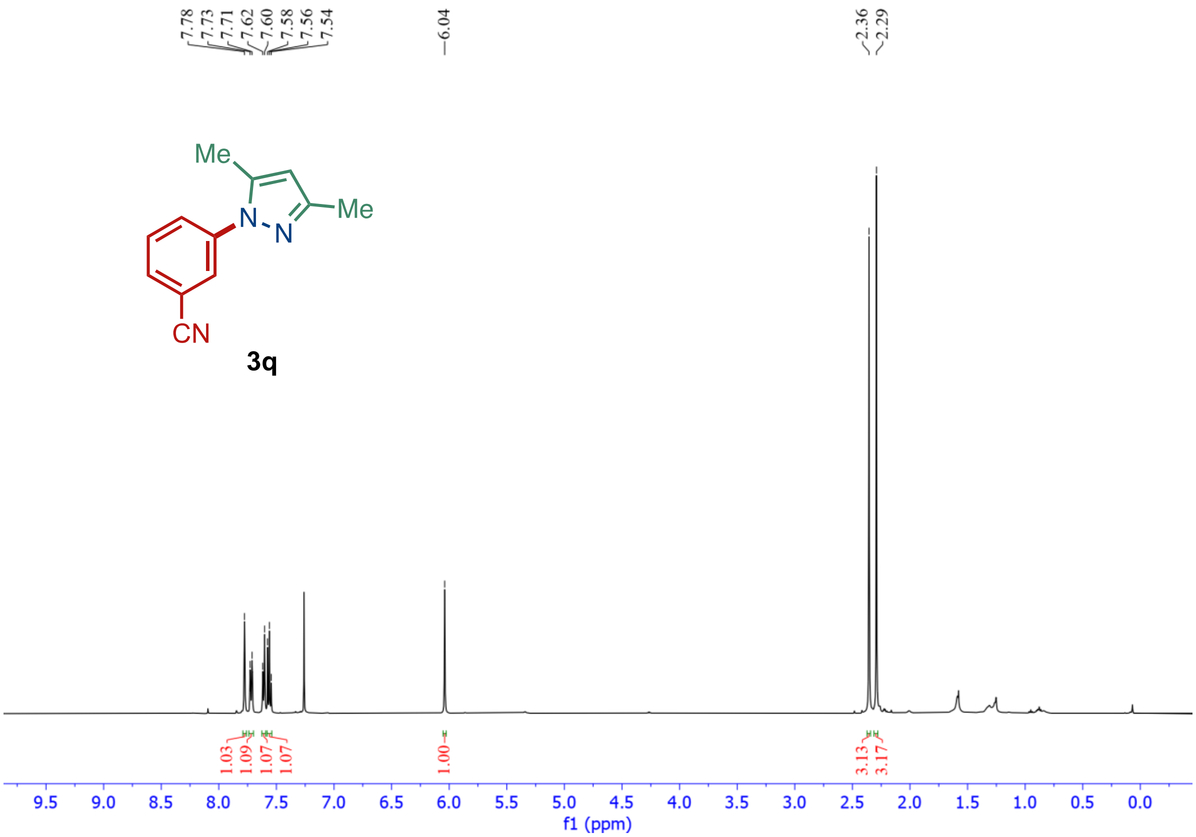

^{1}H NMR (500 MHz, $CDCl_3$) Spectrum of 3-(3,5-Dimethyl-1*H*-pyrazol-1-yl)benzonitrile (**3q**)

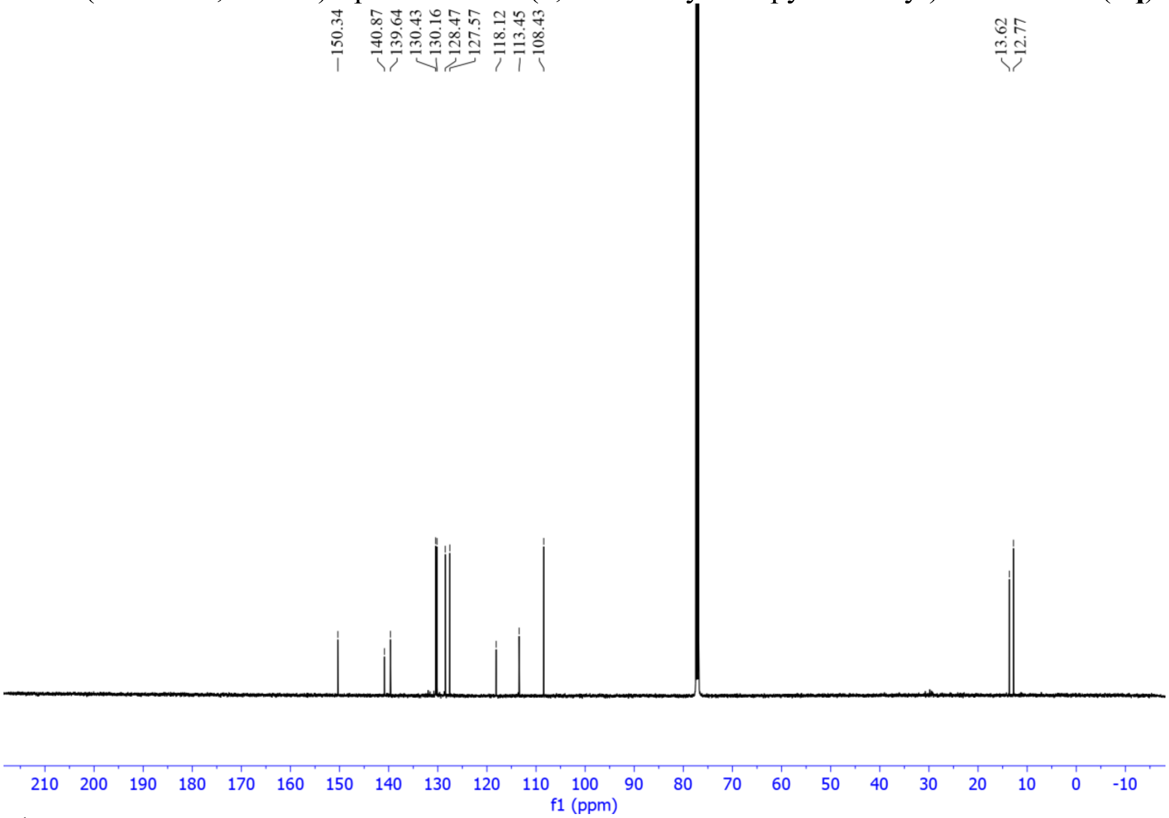

^{13}C{^{1}H} (125 MHz, $CDCl_3$) Spectrum of 3-(3,5-Dimethyl-1*H*-pyrazol-1-yl)benzonitrile (**3q**)

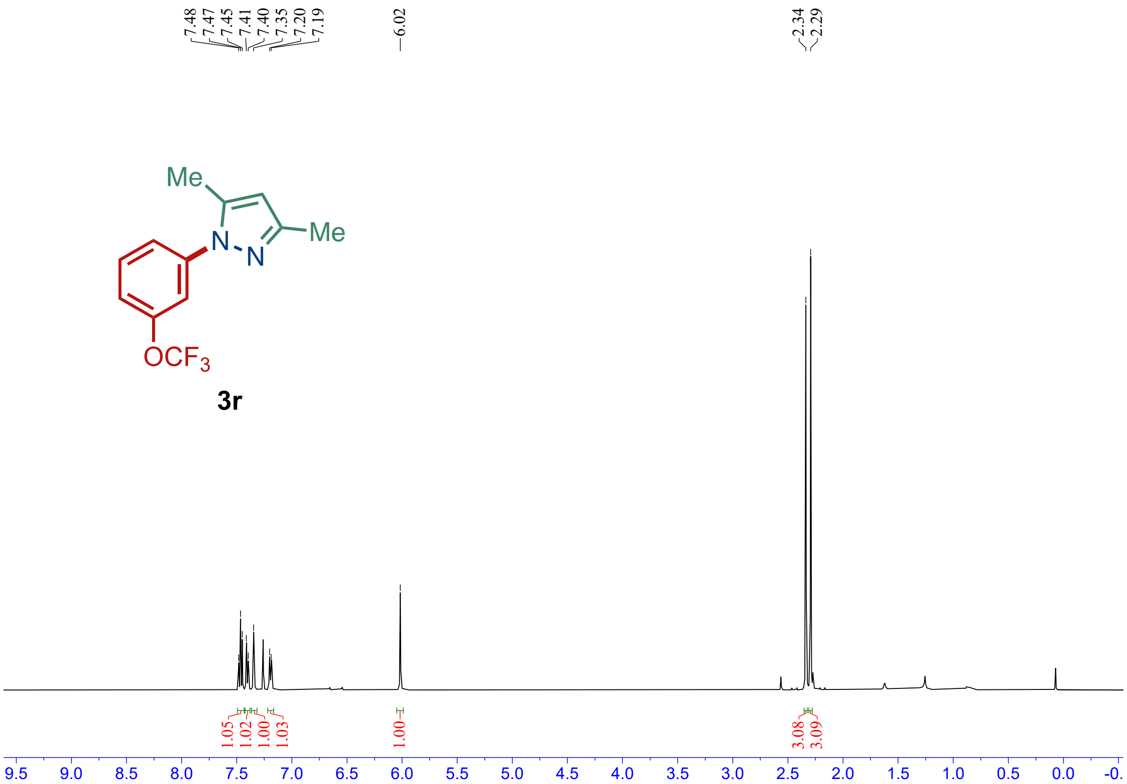

^{1}H NMR (500 MHz, $CDCl_3$) Spectrum of 3,5-Dimethyl-1-(3-(trifluoromethoxy)phenyl)-1*H*-pyrazole (**3r**)

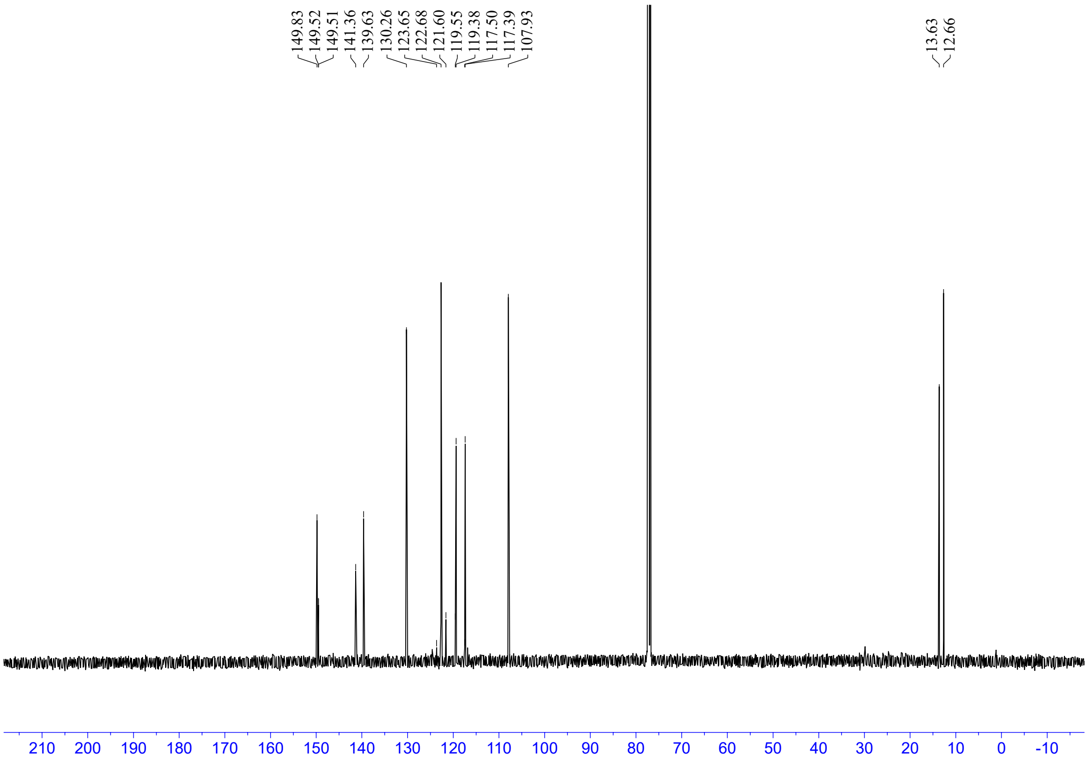

^{13}C{^{1}H} (125 MHz, $CDCl_3$) Spectrum of 3,5-Dimethyl-1-(3-(trifluoromethoxy)phenyl)-1*H*-pyrazole (**3r**)

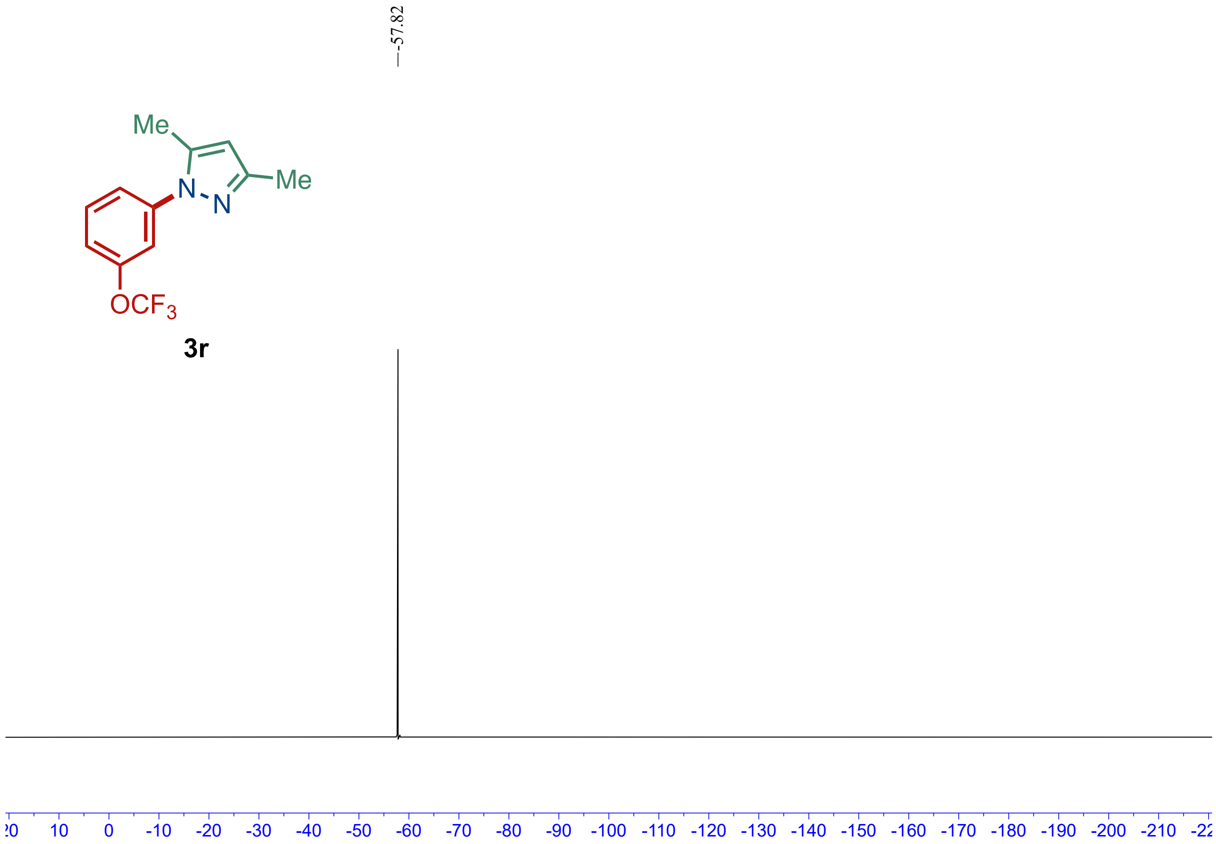

^{19}F NMR (471 MHz, $CDCl_3$) Spectrum of 3,5-Dimethyl-1-(3-(trifluoromethoxy)phenyl)-1*H*-pyrazole (**3r**)

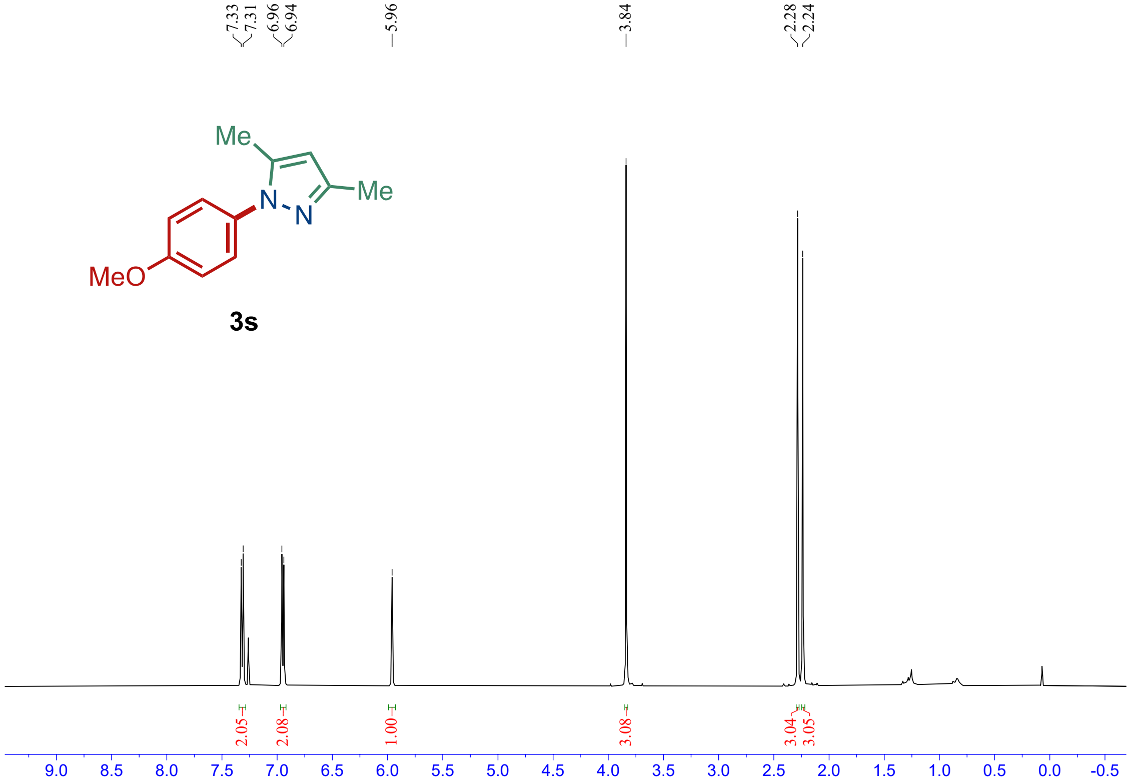

^{1}H NMR (500 MHz, CDCl3) Spectrum of 1-(4-Methoxyphenyl)-3,5-dimethyl-1*H*-pyrazole (**3s**)

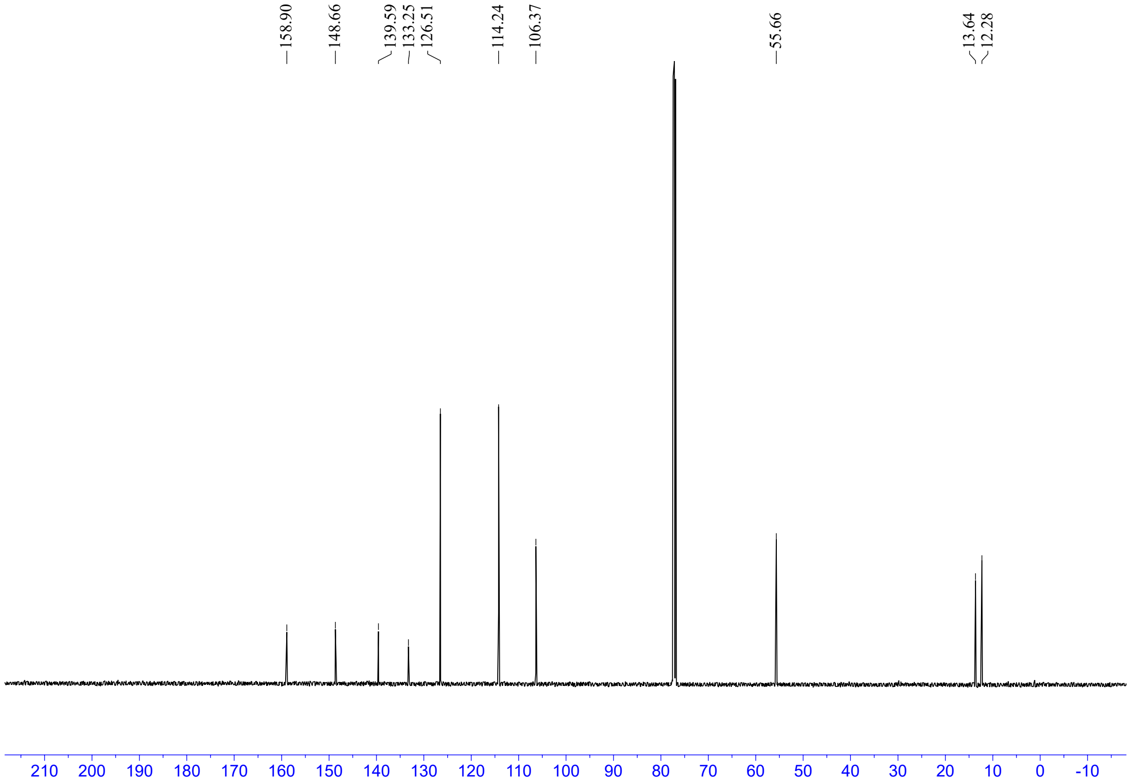

^{13}C{^{1}H} (125 MHz, CDCl3) Spectrum of 1-(4-Methoxyphenyl)-3,5-dimethyl-1*H*-pyrazole (**3s**)

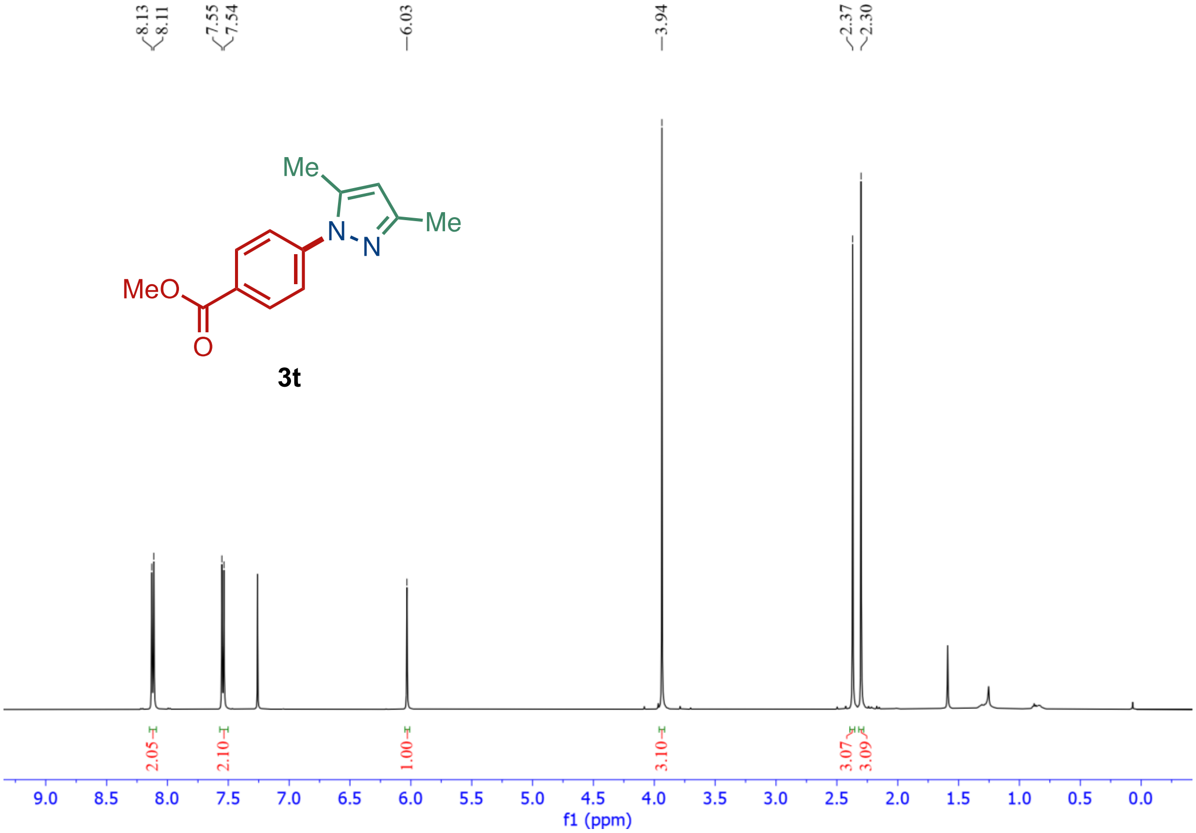

^{1}H NMR (500 MHz, $CDCl_3$) Spectrum of Methyl 4-(3,5-dimethyl-1*H*-pyrazol-1-yl)benzoate (**3t**)

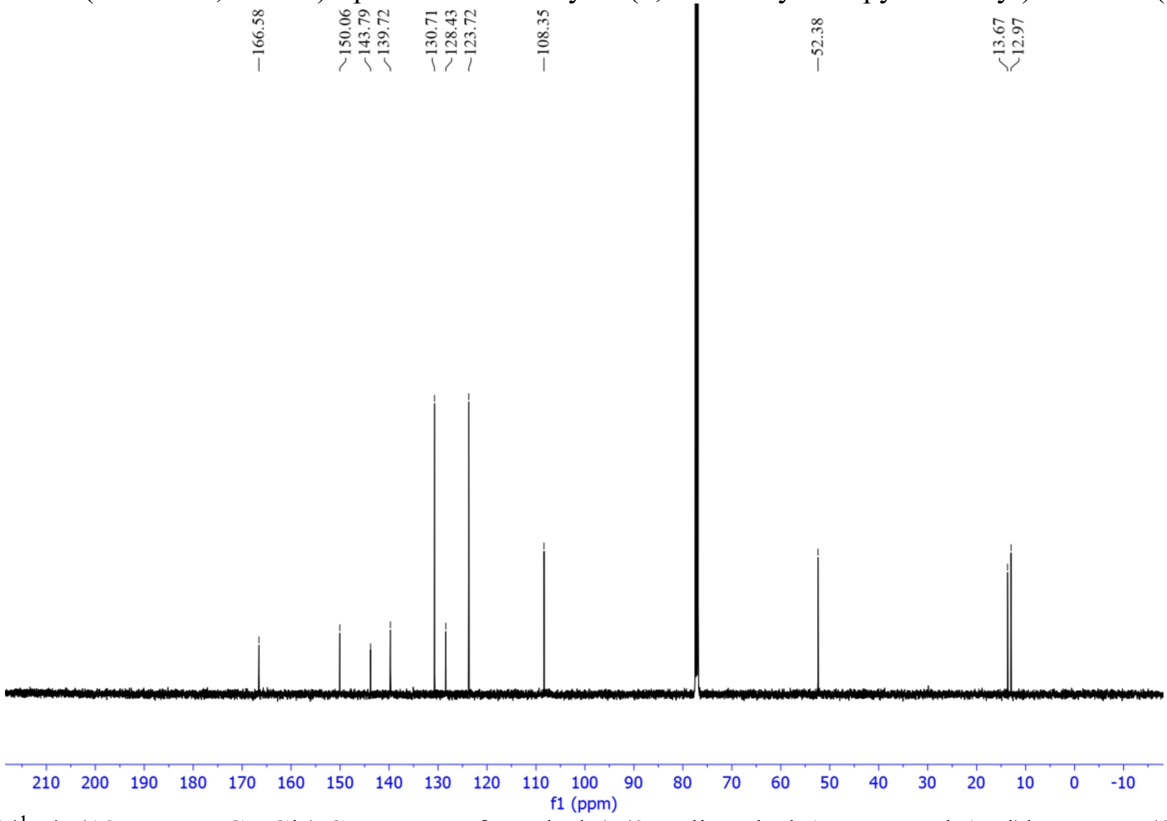

$^{13}C\{^{1}H\}$ (125 MHz, $CDCl_3$) Spectrum of Methyl 4-(3,5-dimethyl-1*H*-pyrazol-1-yl)benzoate (**3t**)

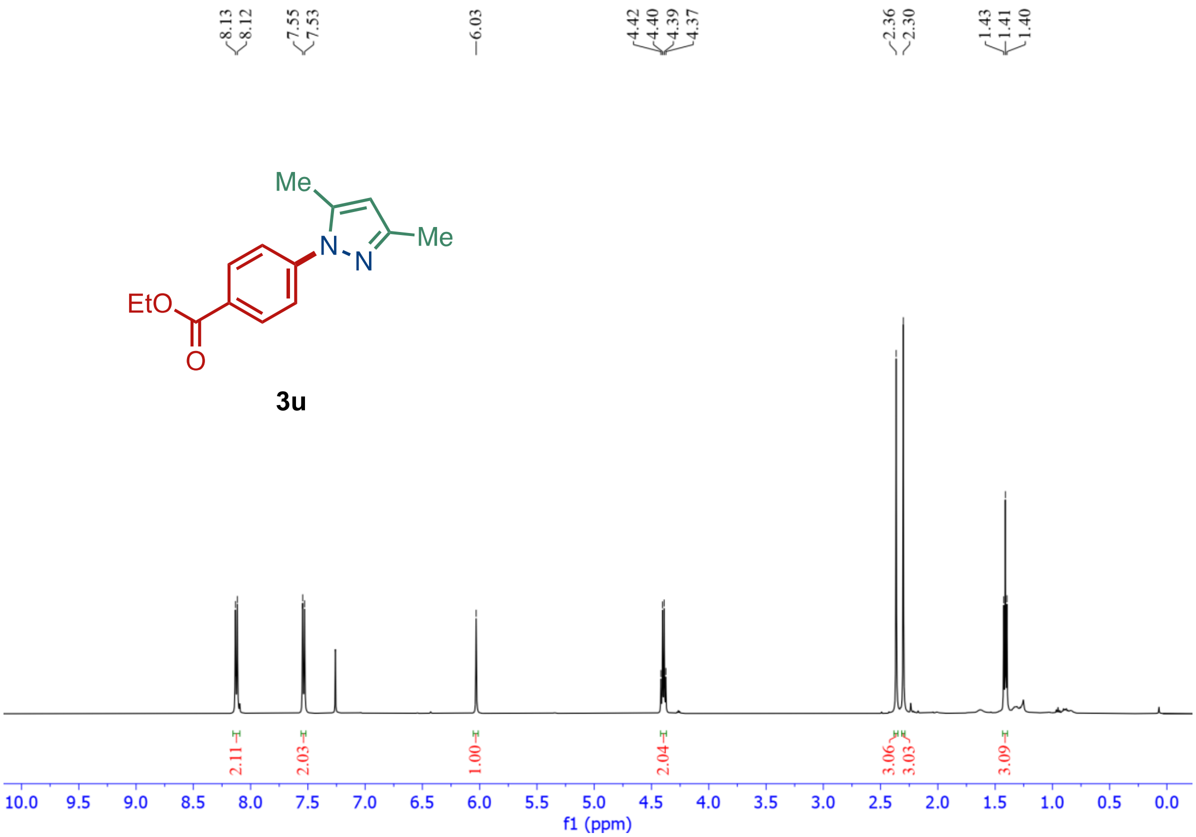

^{1}H NMR (500 MHz, $CDCl_3$) Spectrum of Ethyl 4-(3,5-dimethyl-1*H*-pyrazol-1-yl)benzoate (**3u**)

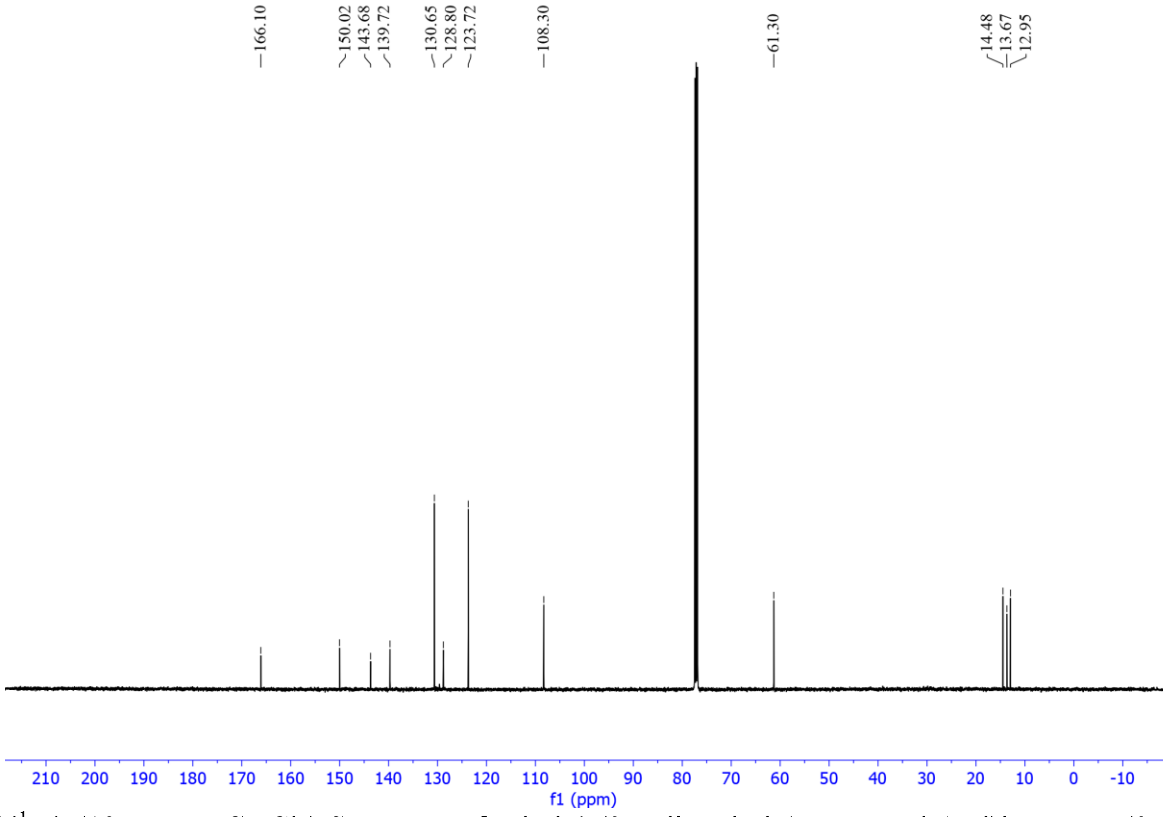

^{13}C{^{1}H} (125 MHz, $CDCl_3$) Spectrum of Ethyl 4-(3,5-dimethyl-1*H*-pyrazol-1-yl)benzoate (**3u**)

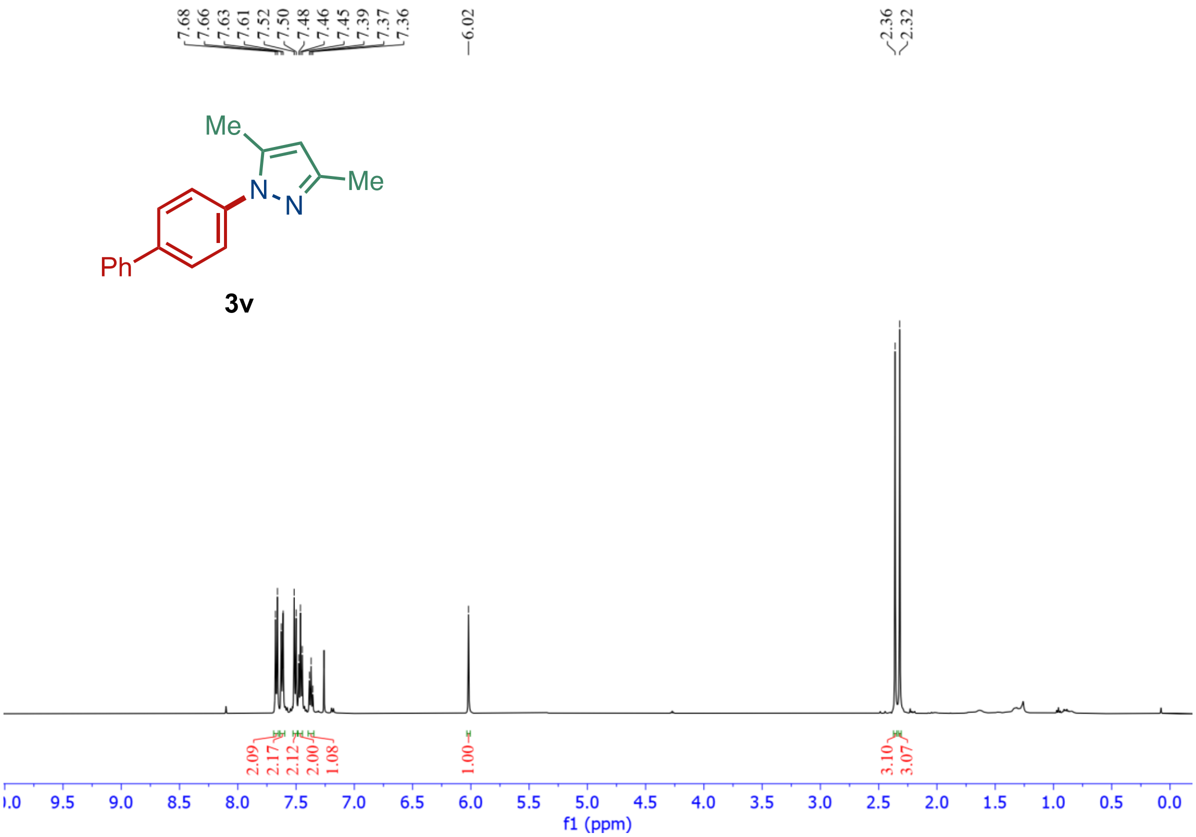

^{1}H NMR (500 MHz, $CDCl_3$) Spectrum of 1-(Biphenyl-4-yl)-3,5-dimethyl-1*H*-pyrazole (**3v**)

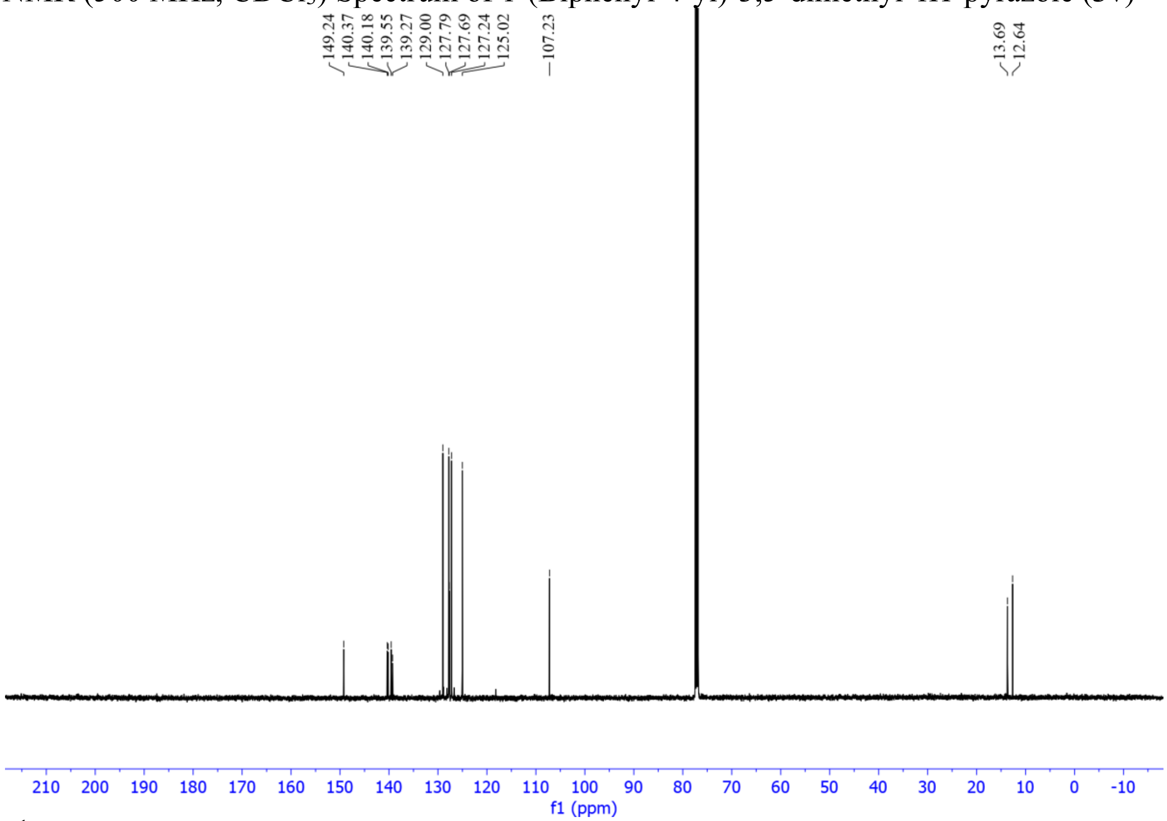

^{13}C{^{1}H} NMR (125 MHz, $CDCl_3$) Spectrum of 1-(Biphenyl-4-yl)-3,5-dimethyl-1*H*-pyrazole (**3v**)

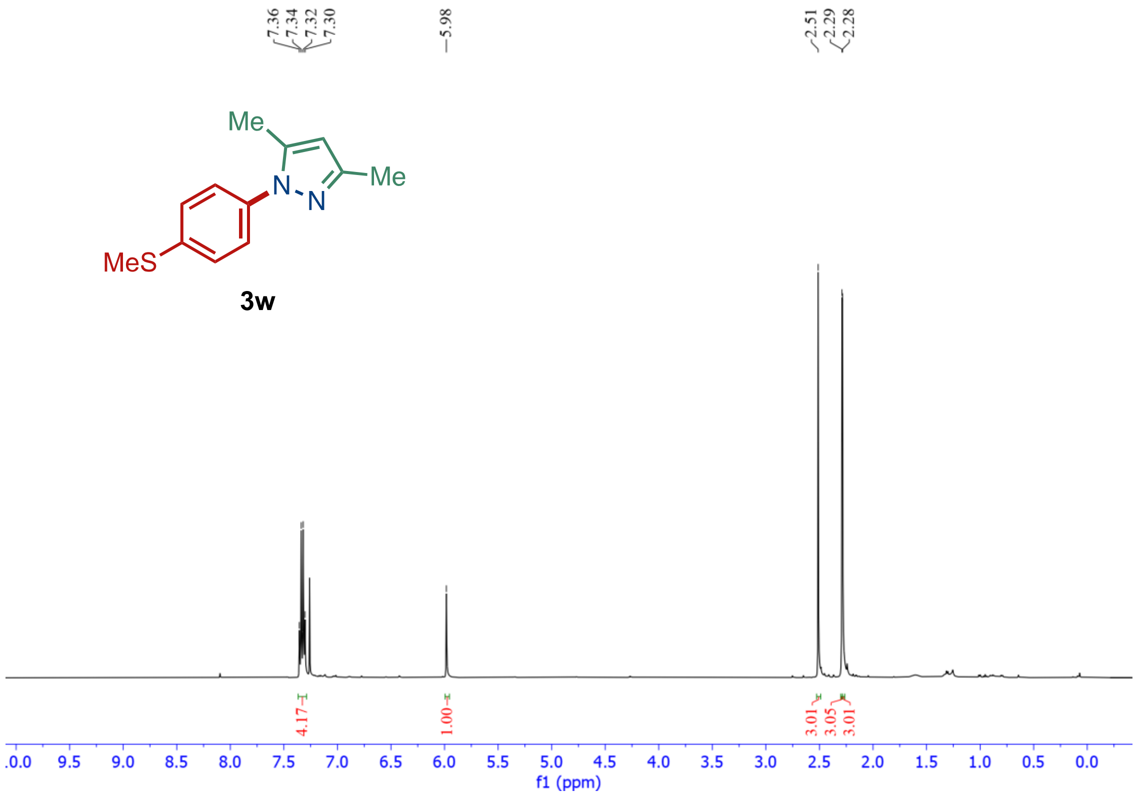

^{1}H NMR (500 MHz, $CDCl_3$) Spectrum of 3,5-Dimethyl-1-(4-(methylthio)phenyl)-1*H*-pyrazole (**3w**)

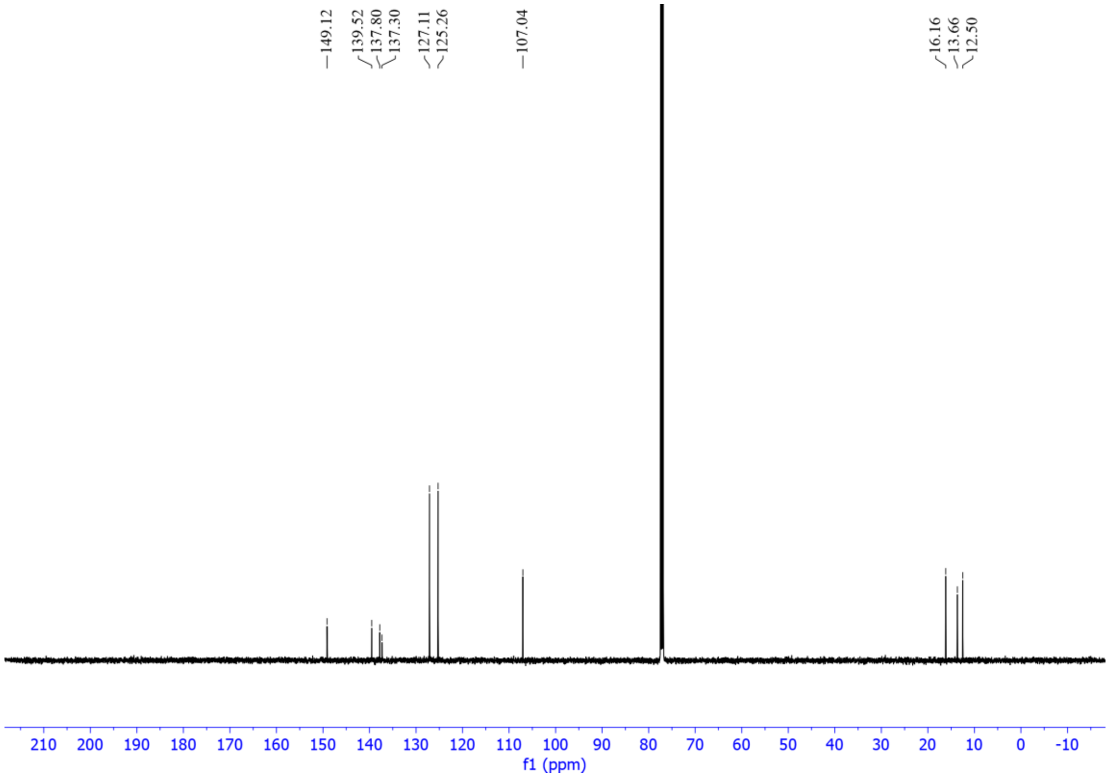

^{13}C{^{1}H} NMR (125 MHz, $CDCl_3$) Spectrum of 3,5-Dimethyl-1-(4-(methylthio)phenyl)-1*H*-pyrazole (**3w**)

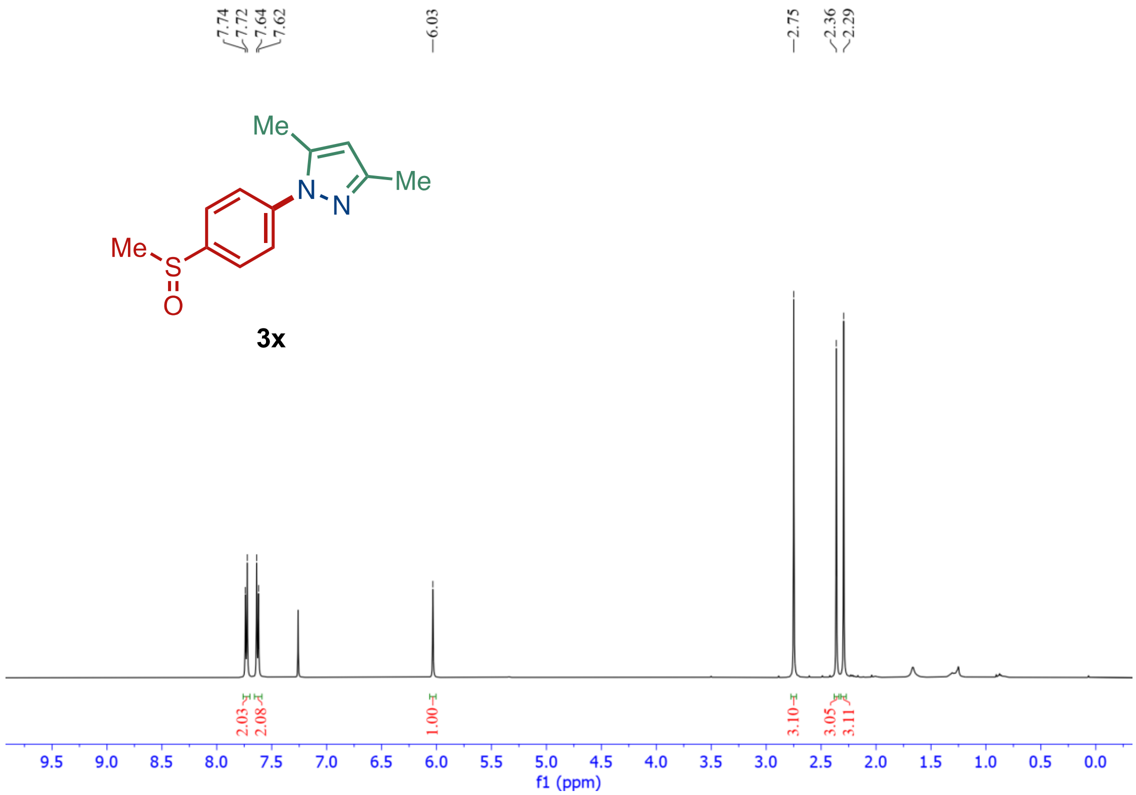

^{1}H NMR (500 MHz, $CDCl_3$) Spectrum of 3,5-Dimethyl-1-(4-(methylsulfinyl)phenyl)-1*H*-pyrazole (**3x**)

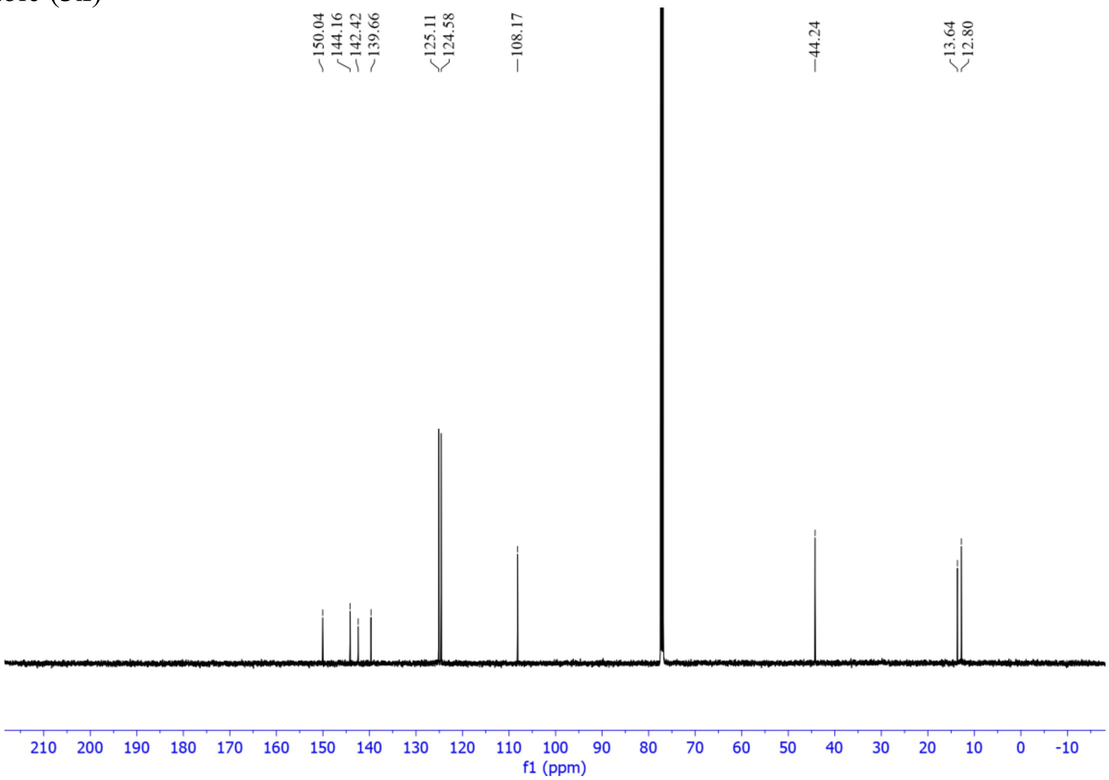

^{13}C{^{1}H} NMR (125 MHz, $CDCl_3$) Spectrum of 3,5-Dimethyl-1-(4-(methylsulfinyl)phenyl)-1*H*-pyrazole (**3x**)

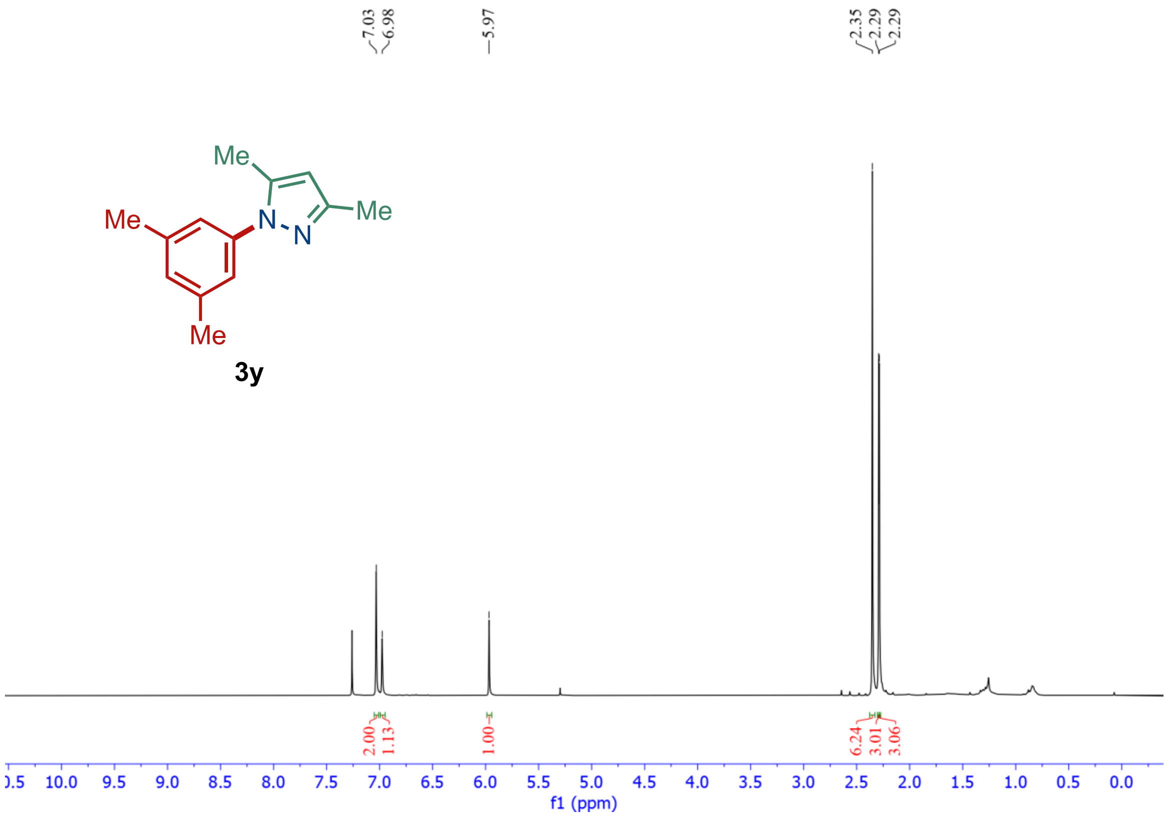

^{1}H NMR (500 MHz, $CDCl_3$) Spectrum of 1-(3,5-Dimethylphenyl)-3,5-dimethyl-1*H*-pyrazole (**3y**)

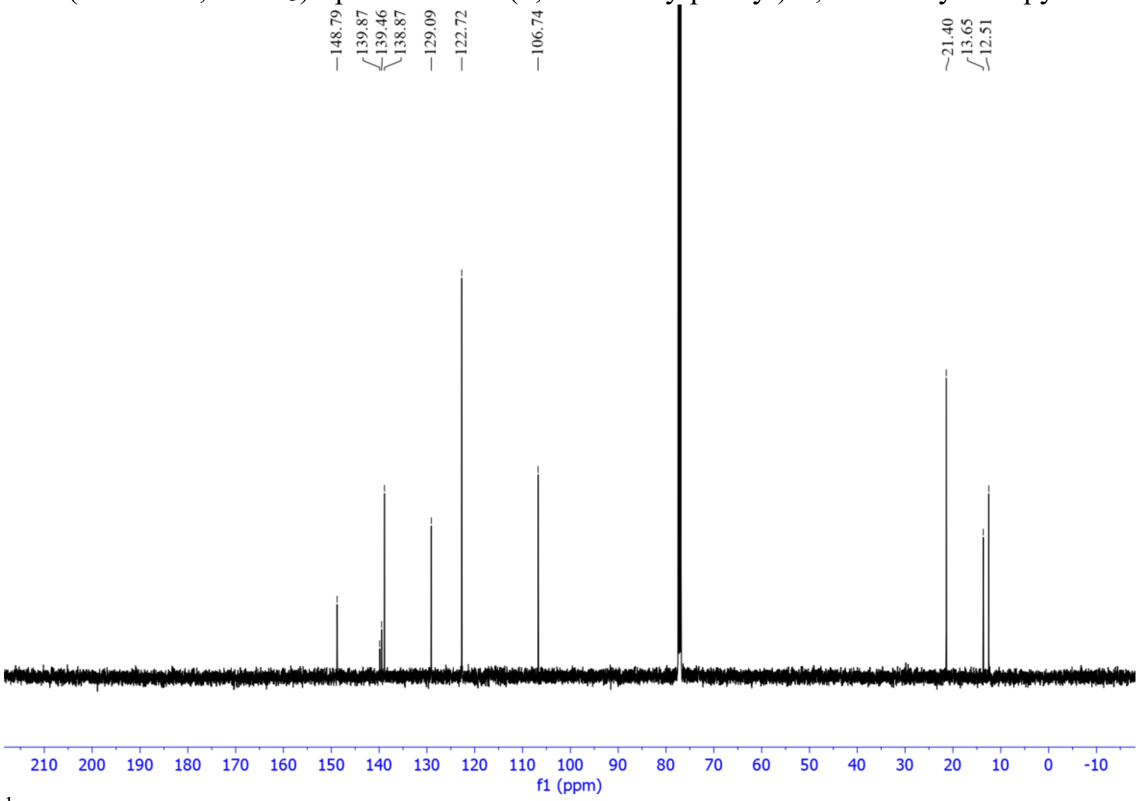

^{13}C{^{1}H} NMR (125 MHz, $CDCl_3$) Spectrum of 1-(3,5-Dimethylphenyl)-3,5-dimethyl-1*H*-pyrazole (**3y**)

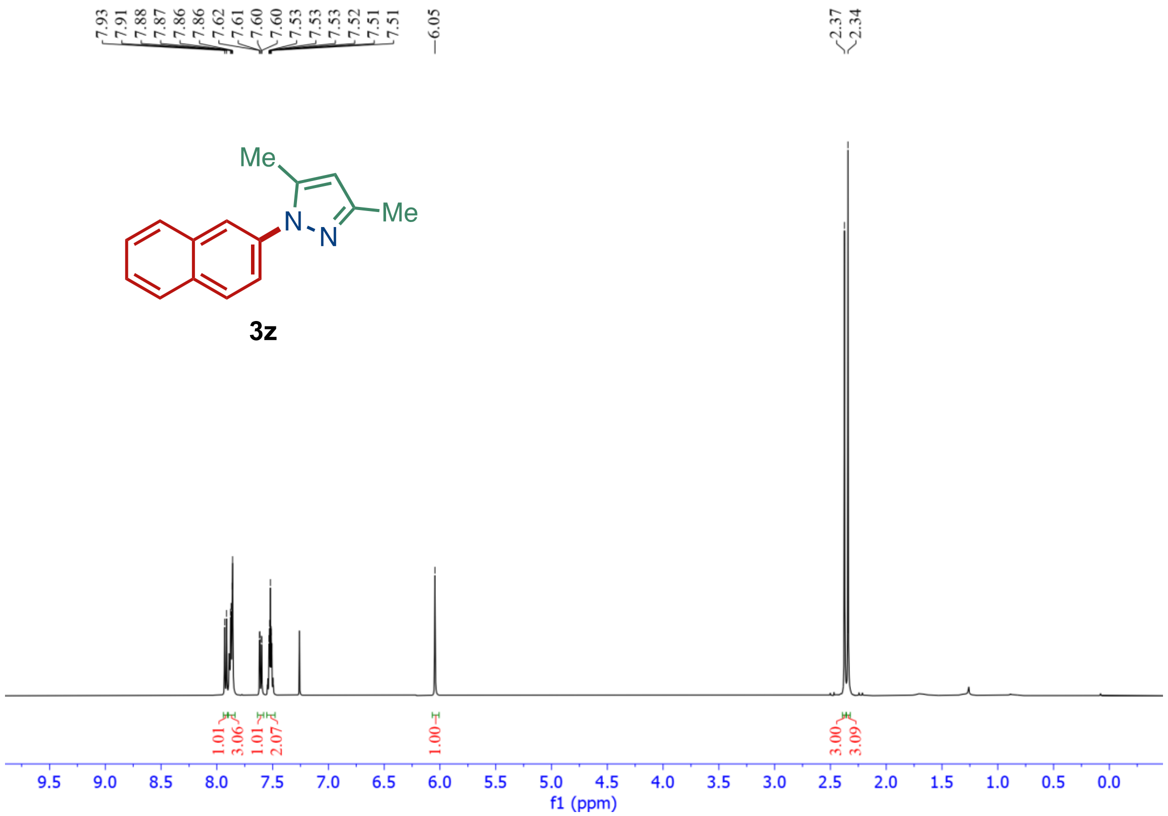

^{1}H NMR (500 MHz, $CDCl_3$) Spectrum of 3,5-Dimethyl-1-(naphthalen-2-yl)-1*H*-pyrazole (**3z**)

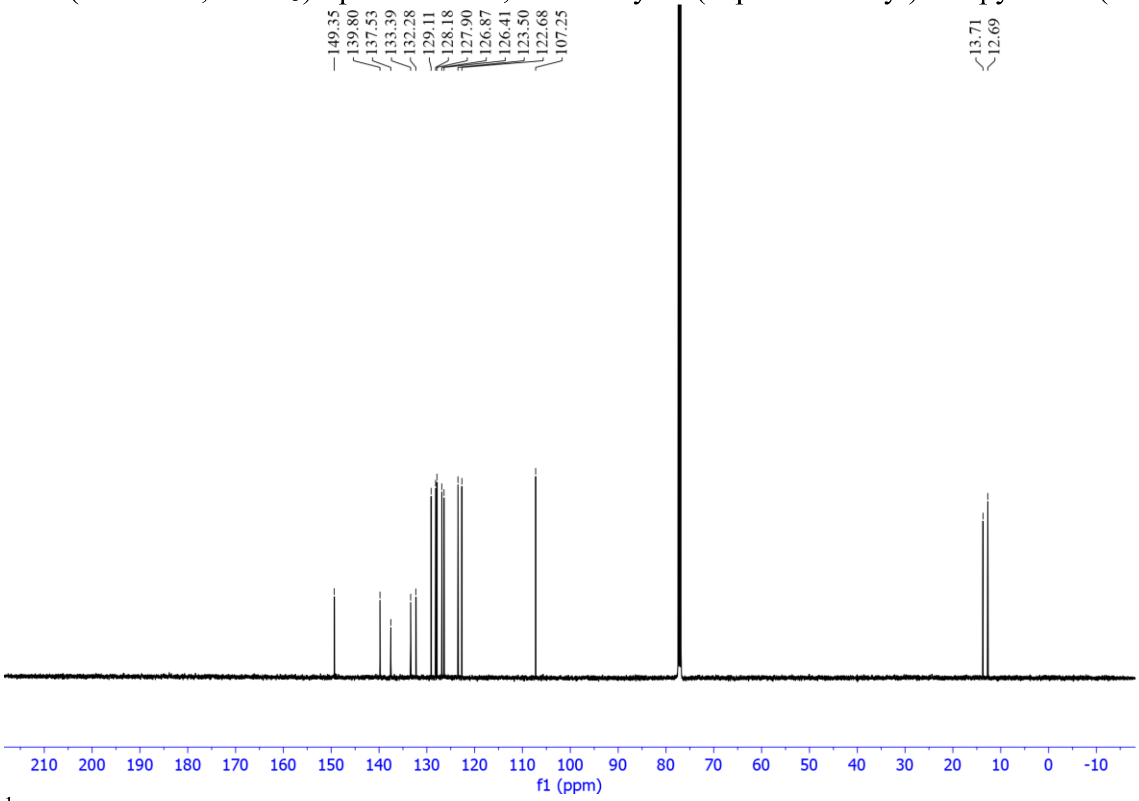

^{13}C{^{1}H} NMR (125 MHz, $CDCl_3$) Spectrum of 3,5-Dimethyl-1-(naphthalen-2-yl)-1*H*-pyrazole (**3z**)

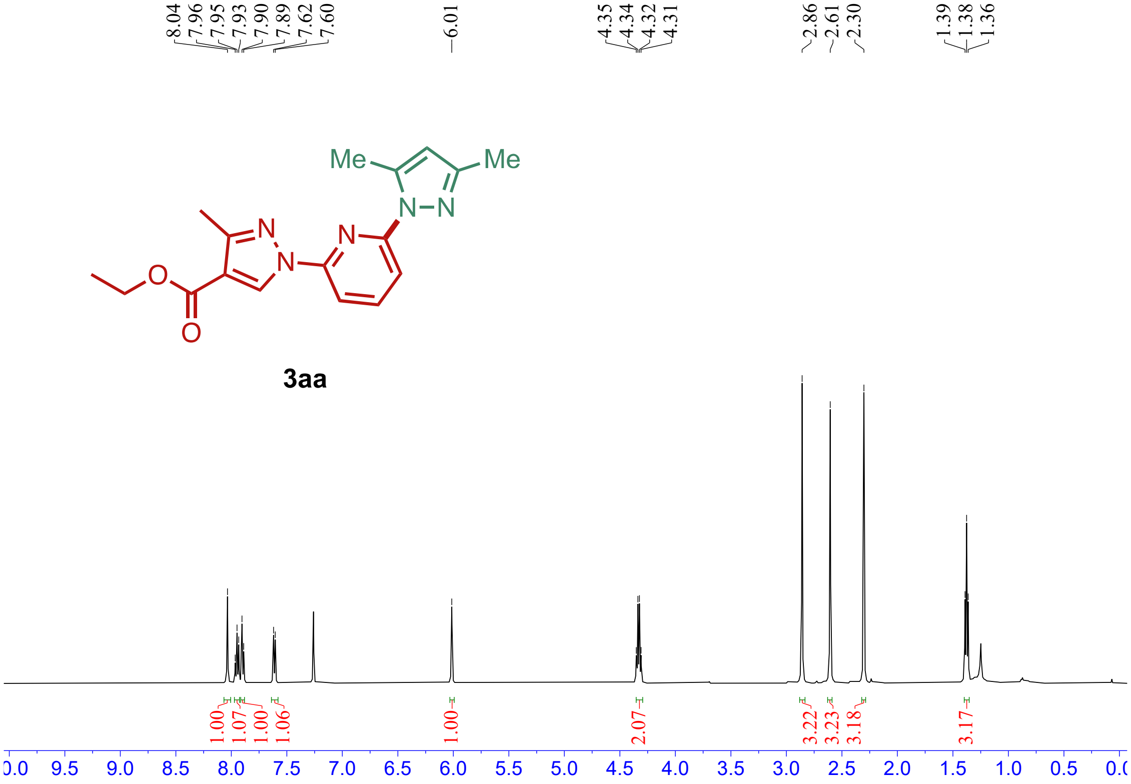

^{1}H NMR (500 MHz, CDCl$_3$) Spectrum of **3aa**

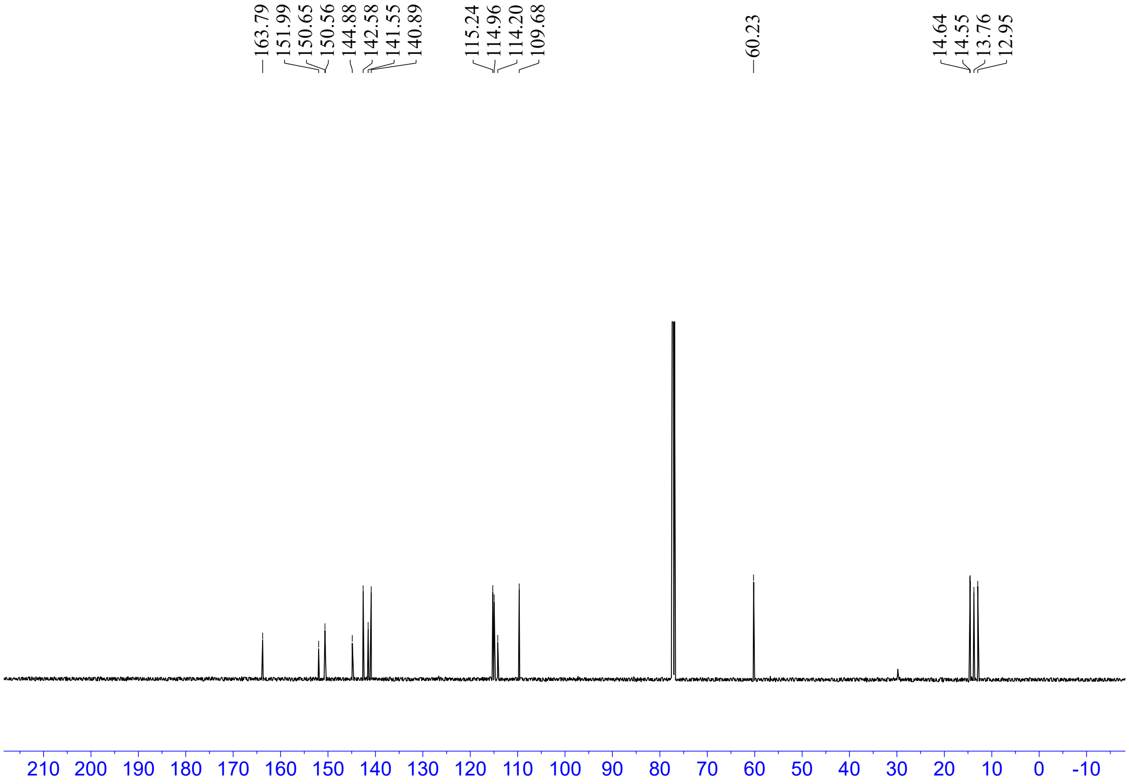

^{13}C{^{1}H} NMR (125 MHz, CDCl$_3$) Spectrum of **3aa**

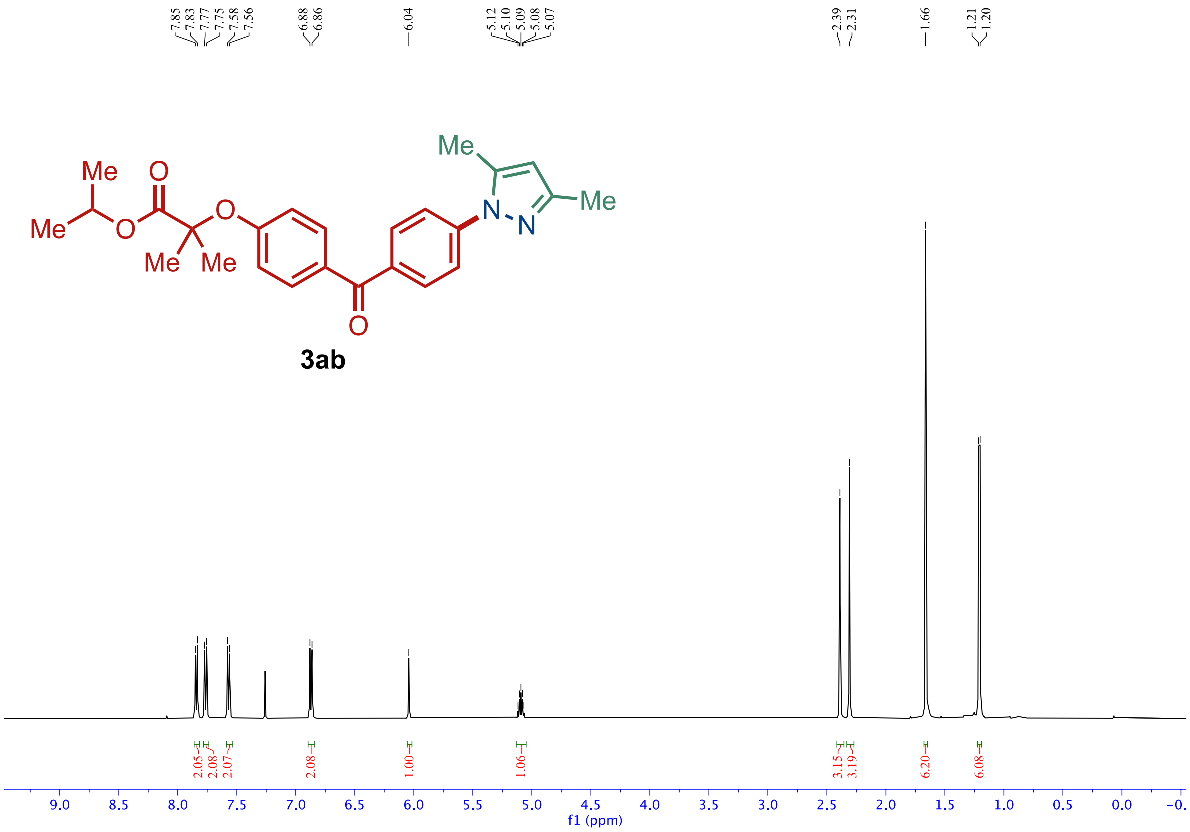

^{1}H NMR (500 MHz, $CDCl_3$) Spectrum of **3ab**

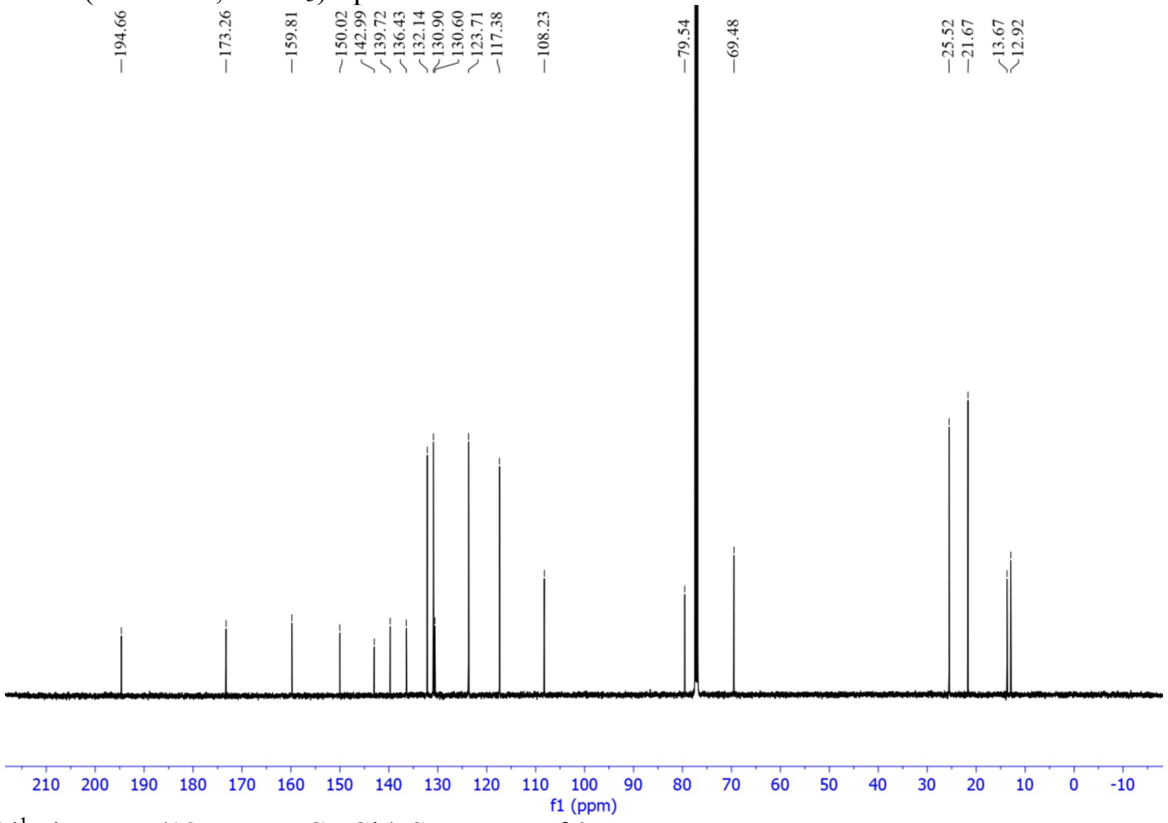

^{13}C{^{1}H} NMR (125 MHz, $CDCl_3$) Spectrum of **3ab**

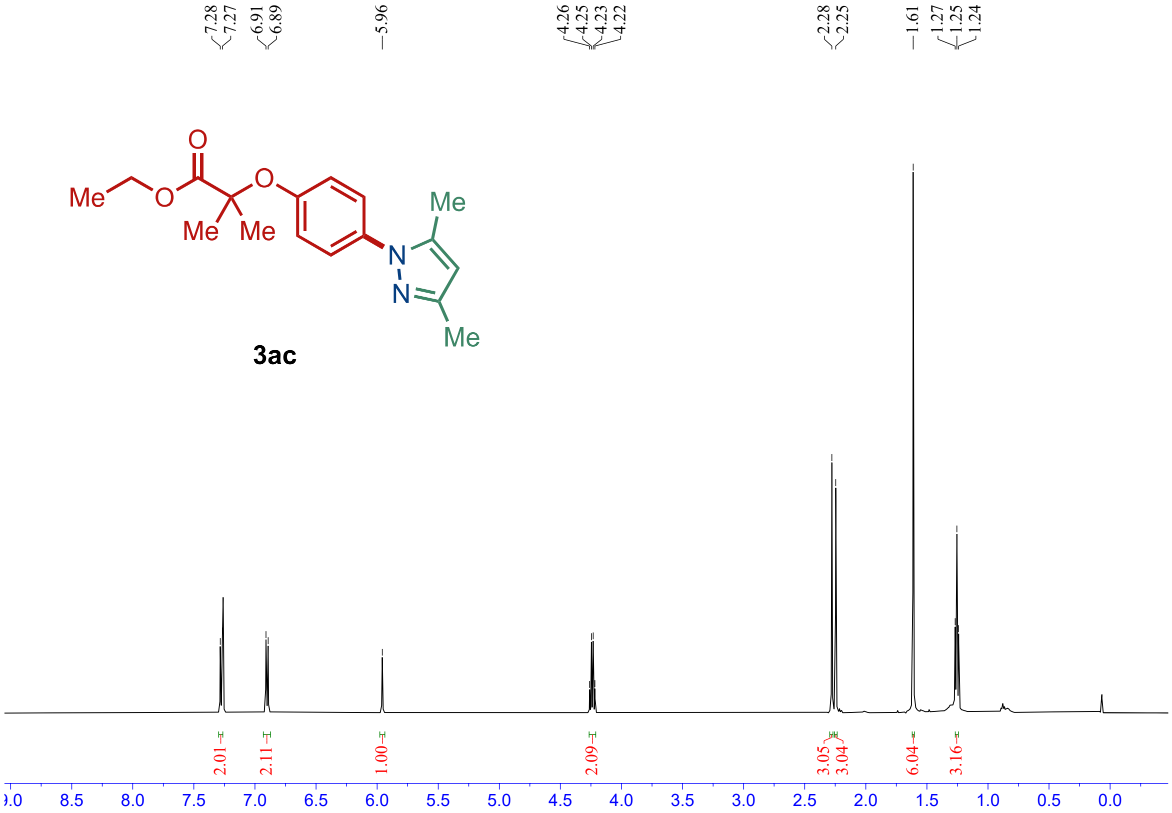

3ac

^{1}H NMR (500 MHz, $CDCl_3$) Spectrum of **3ac**

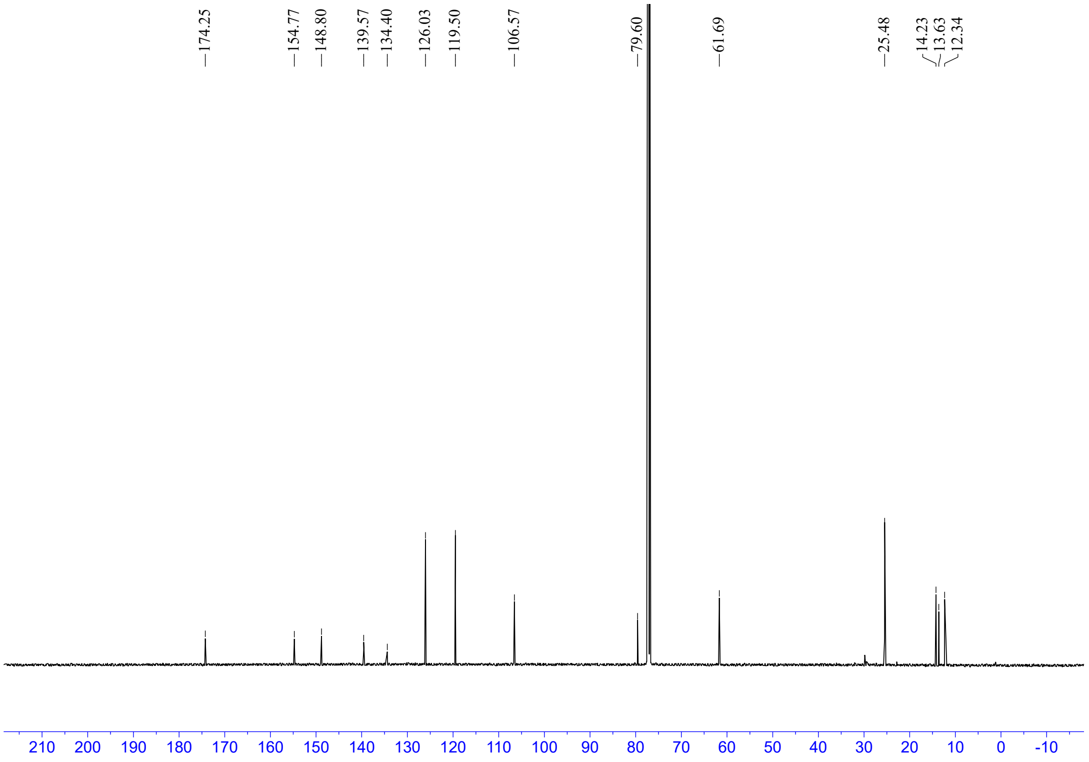

^{13}C{^{1}H} NMR (125 MHz, $CDCl_3$) Spectrum of **3ac**

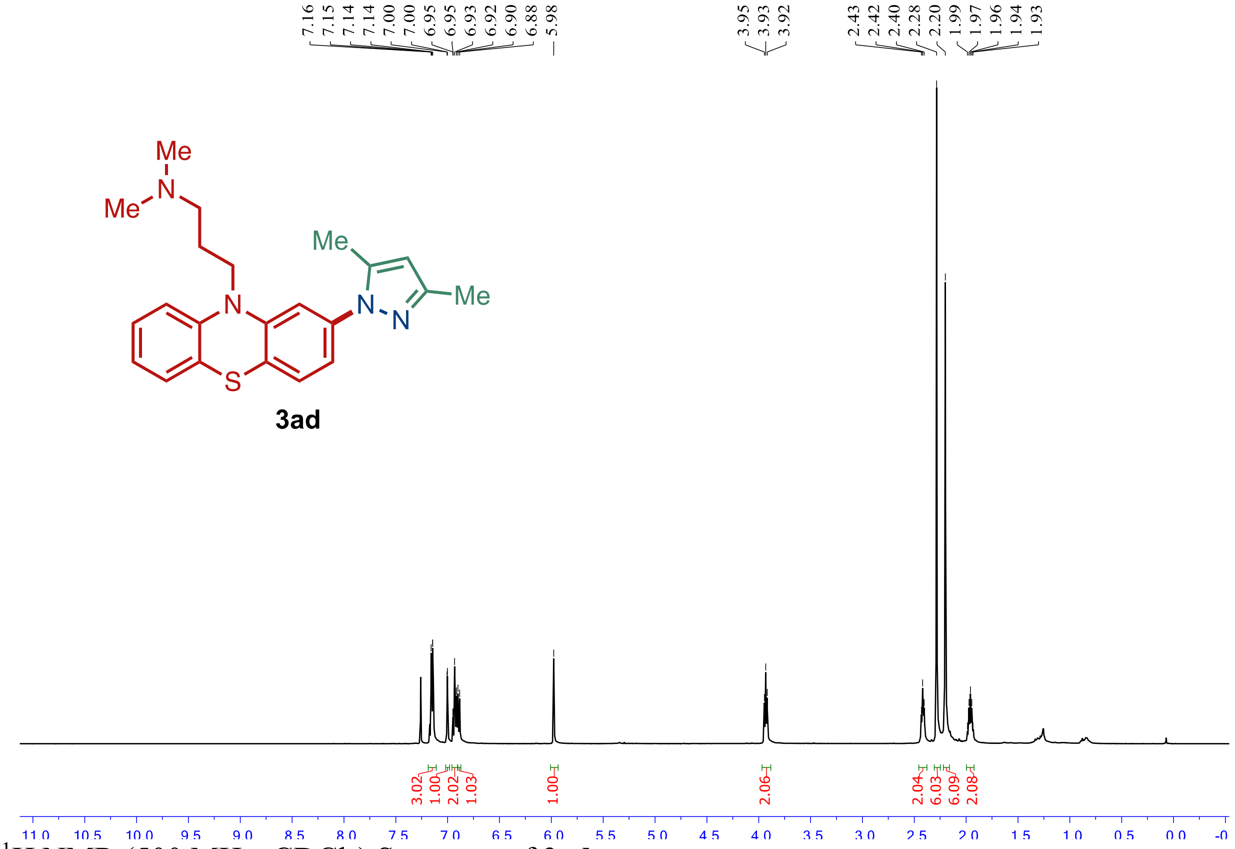

^{1}H NMR (500 MHz, $CDCl_3$) Spectrum of **3ad**

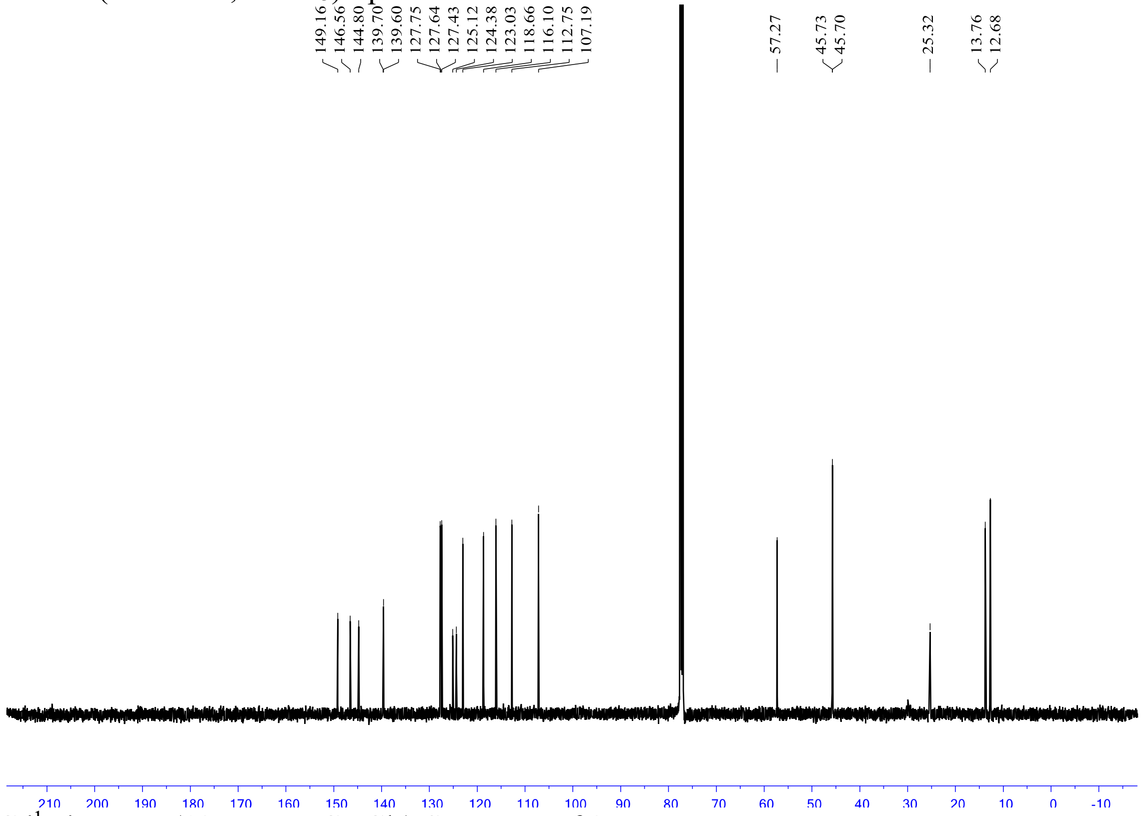

^{13}C{^{1}H} NMR (125 MHz, $CDCl_3$) Spectrum of **3ad**

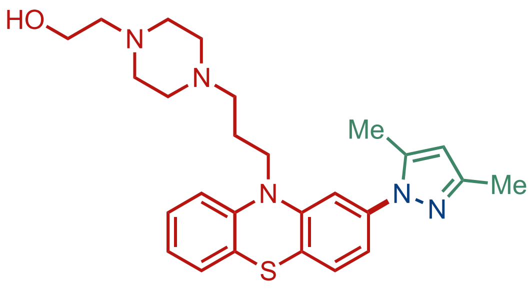

3ae

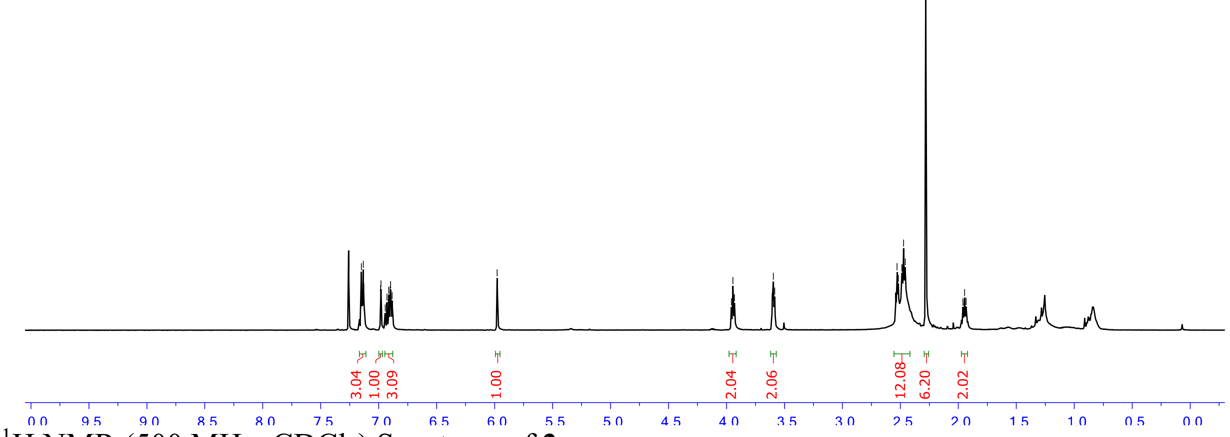

^{1}H NMR (500 MHz, CDCl$_3$) Spectrum of **3ae**

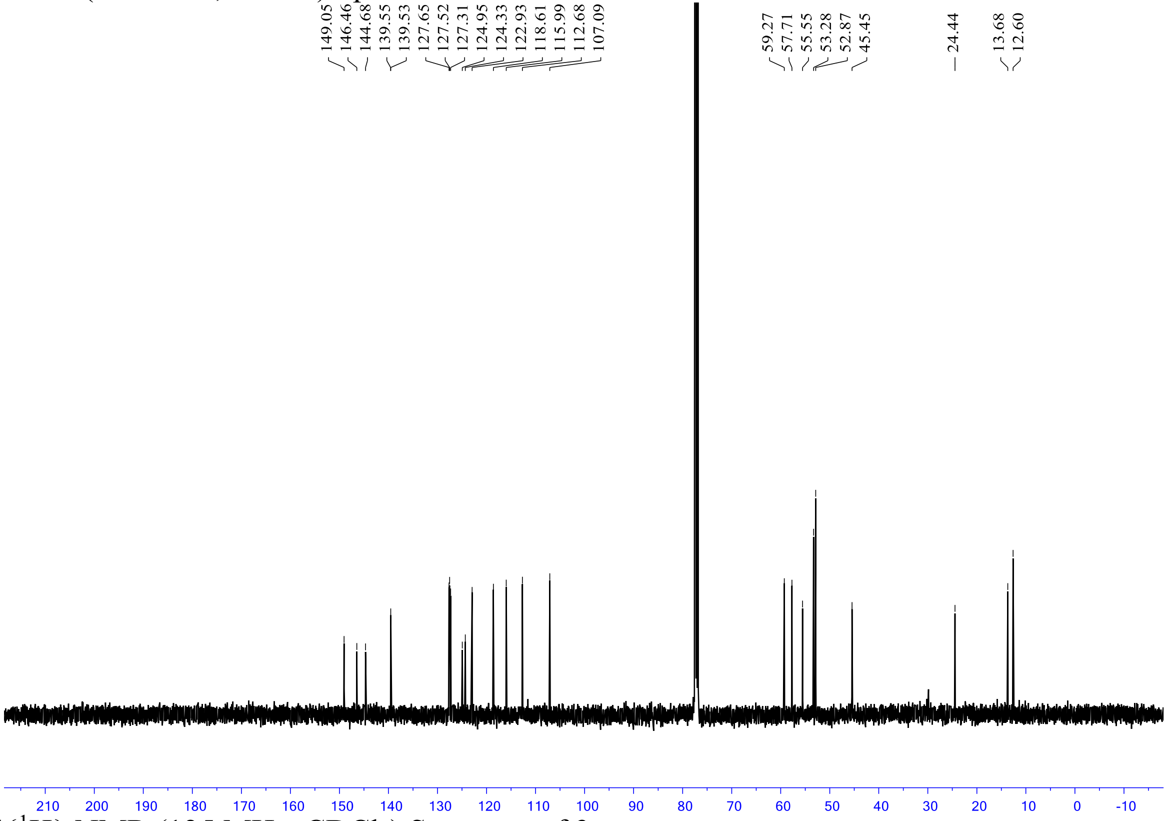

^{13}C{^{1}H} NMR (125 MHz, CDCl$_3$) Spectrum of **3ae**

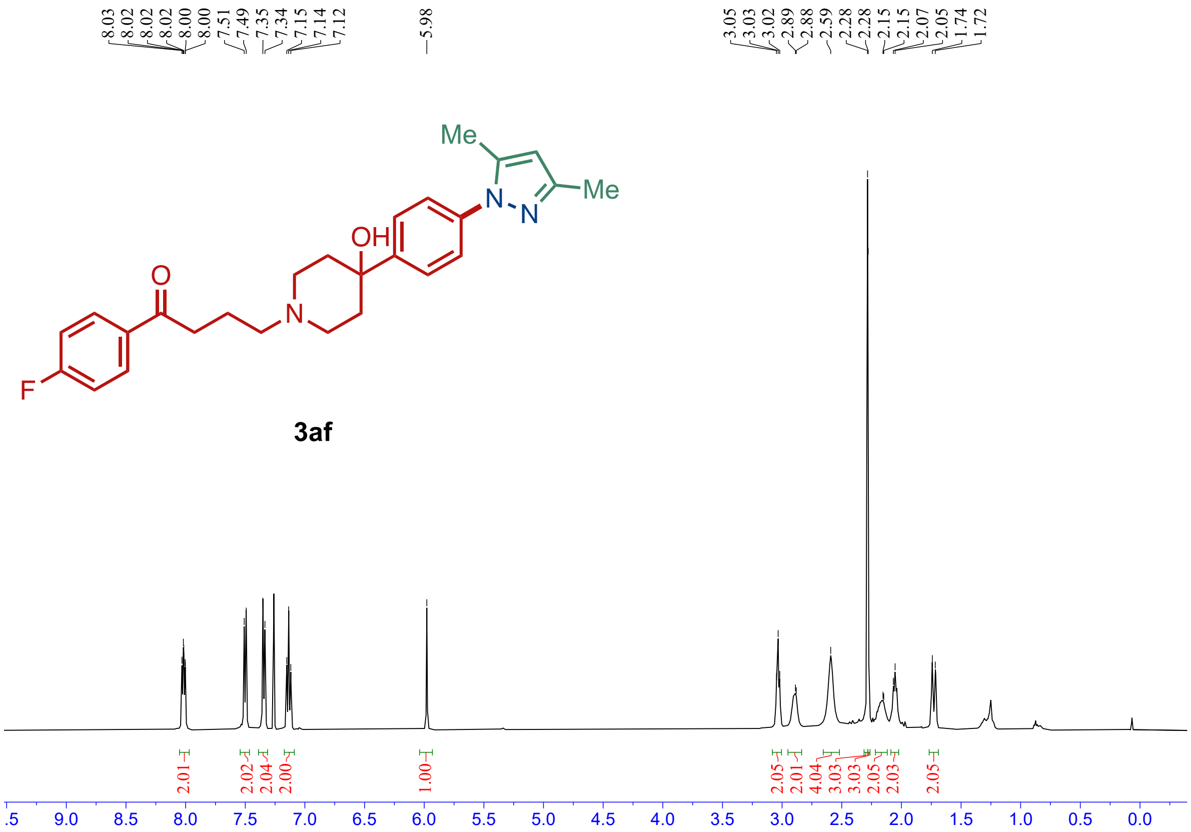

^{1}H NMR (500 MHz, $CDCl_3$) Spectrum of **3af**

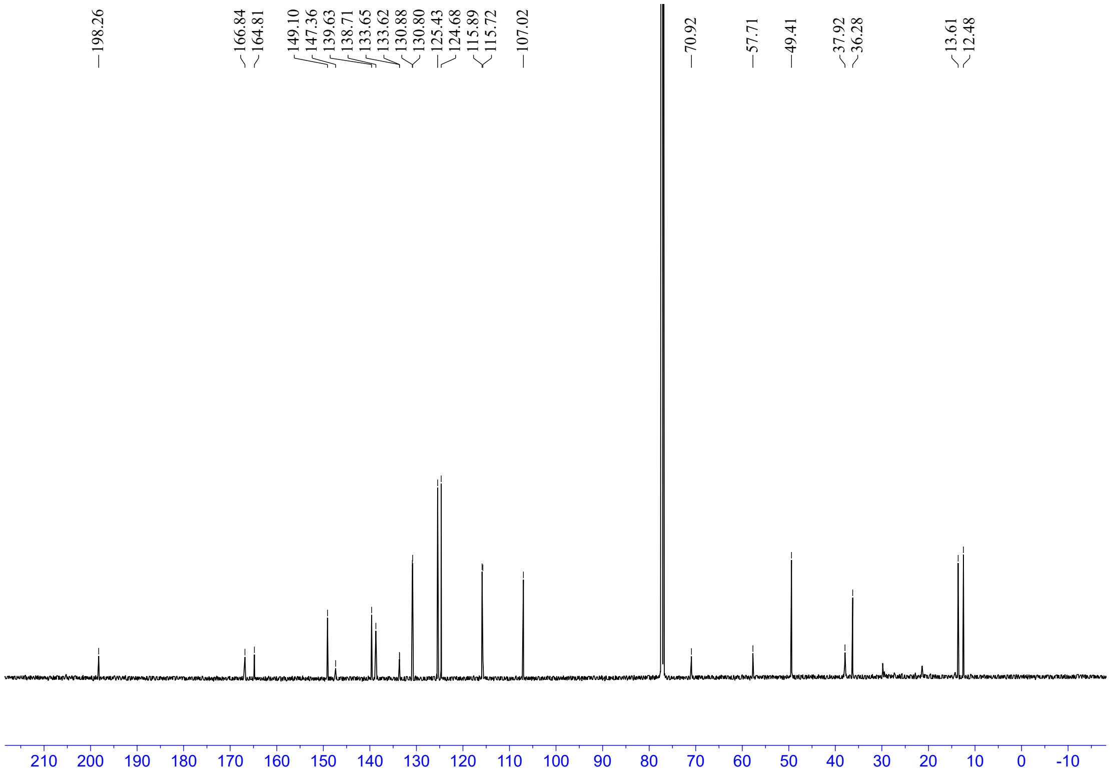

^{13}C{^{1}H} NMR (125 MHz, $CDCl_3$) Spectrum of **3af**

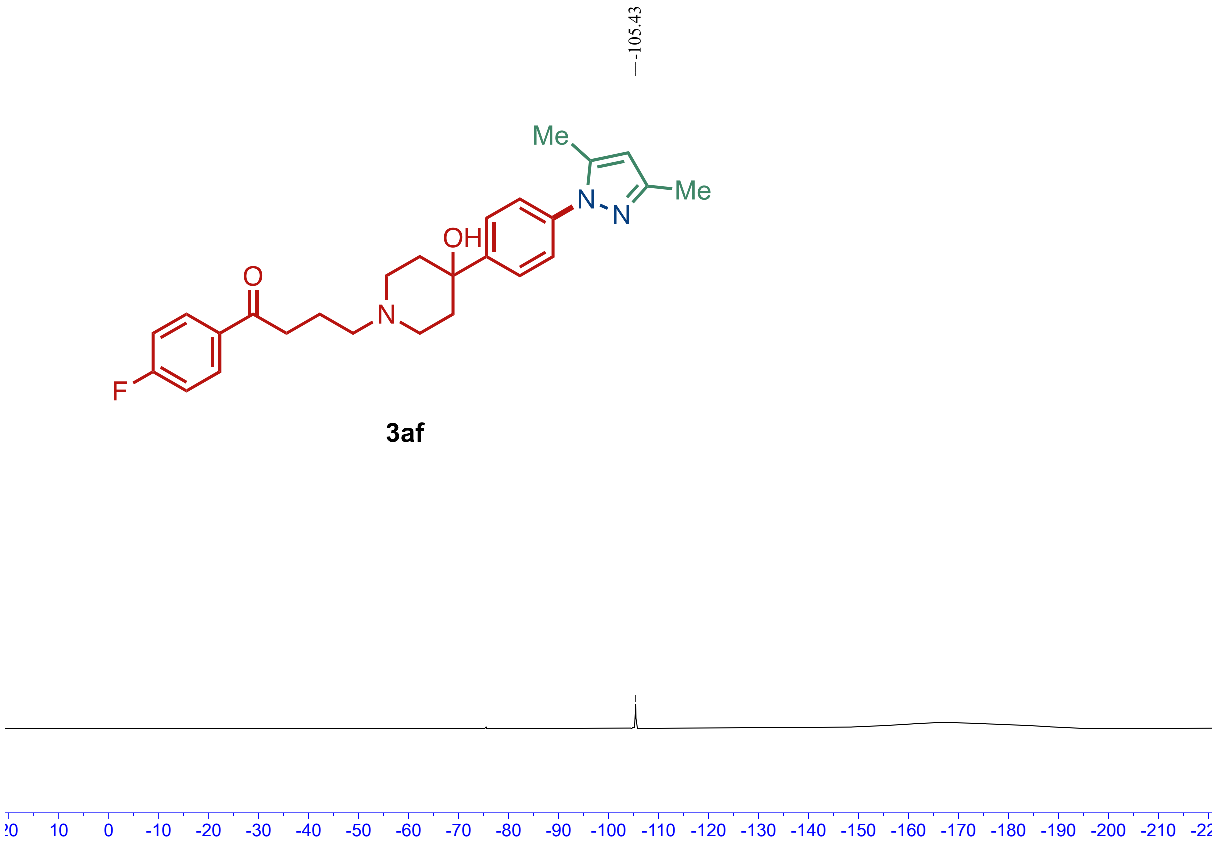

3af

^{19}F NMR (471 MHz, $CDCl_3$) Spectrum of **3af**

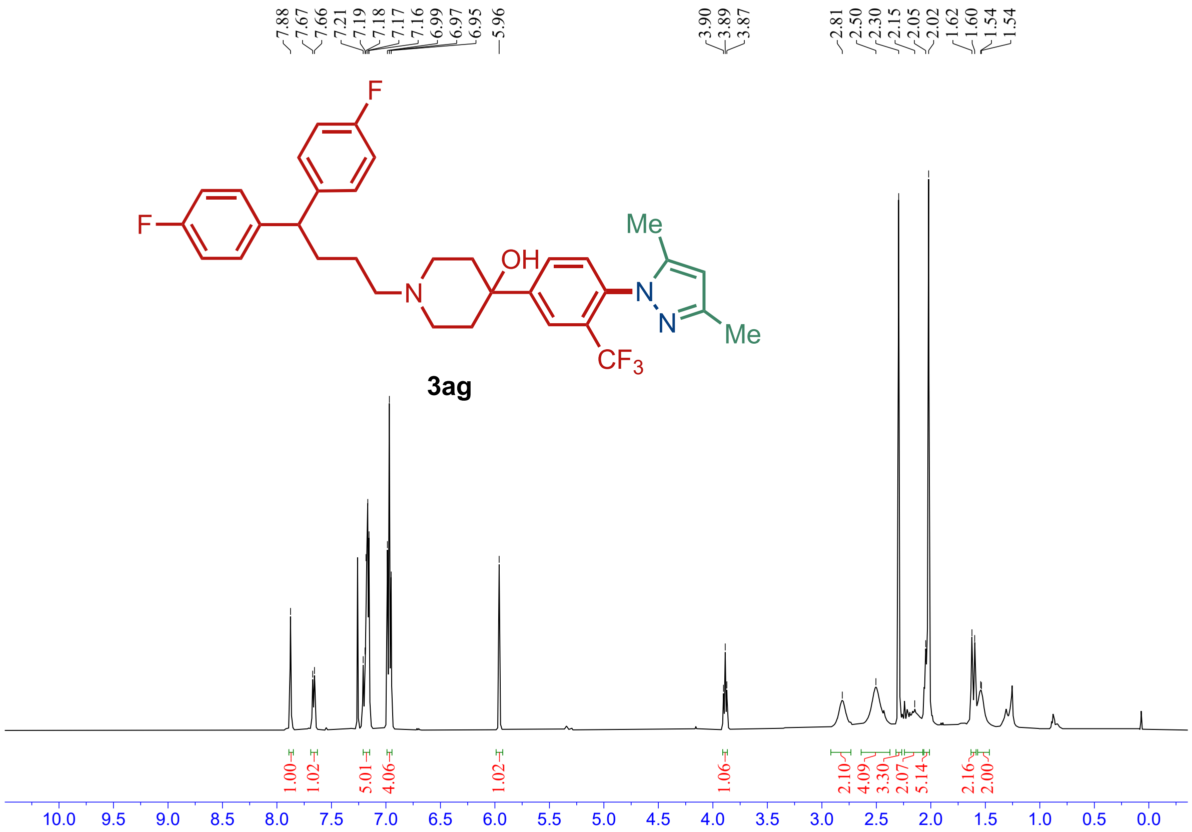

^{1}H NMR (500 MHz, $CDCl_3$) Spectrum of **3ag**

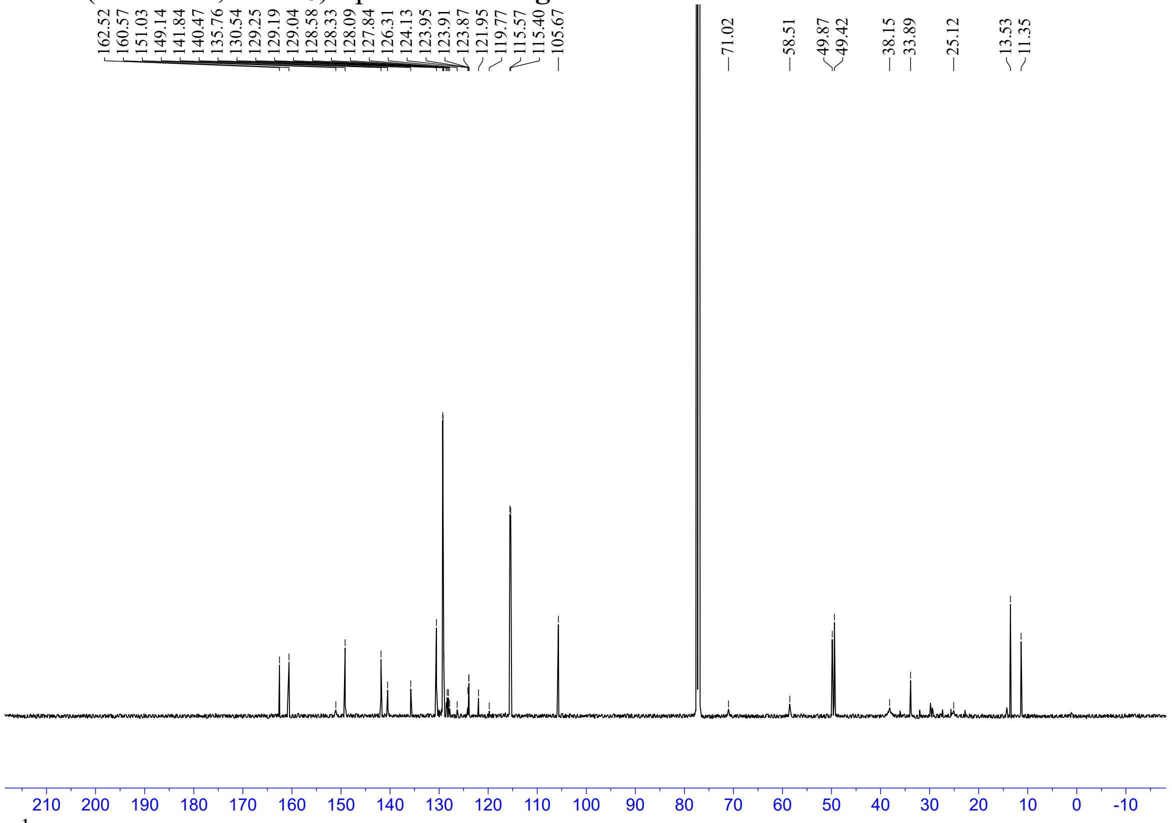

^{13}C{^{1}H} NMR (125 MHz, $CDCl_3$) Spectrum of **3ag**

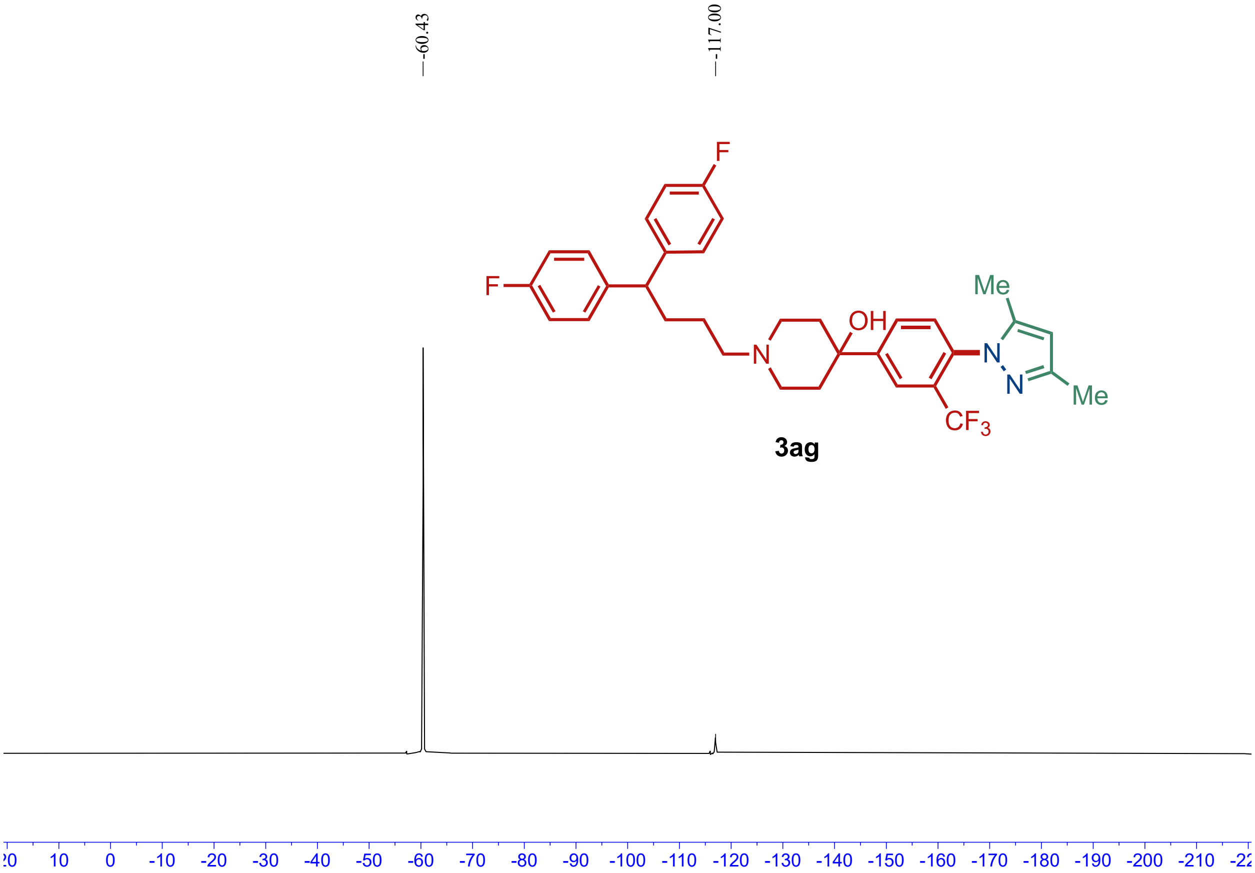

^{19}F NMR (471 MHz, $CDCl_3$) Spectrum of **3ag**

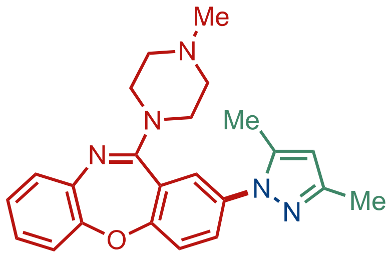

3ah

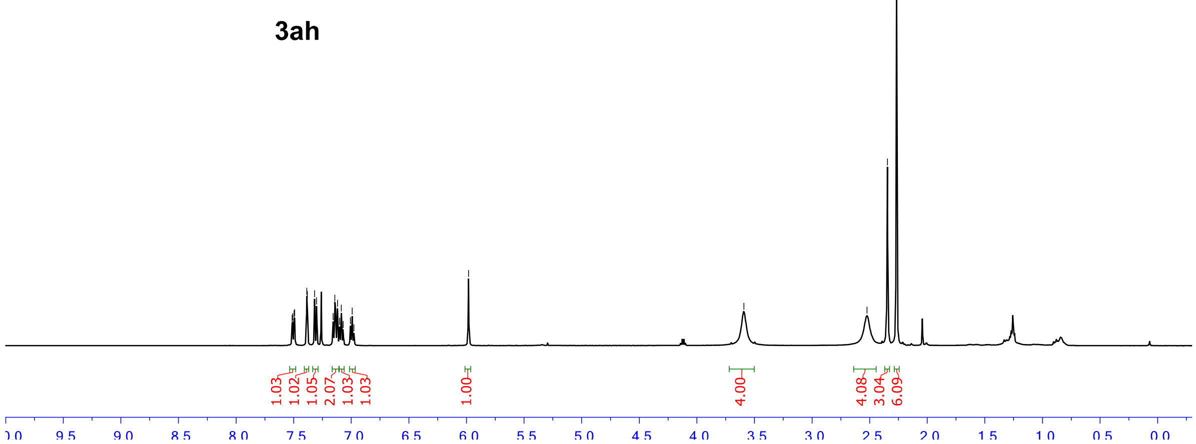

^{1}H NMR (500 MHz, $CDCl_3$) Spectrum of **3ah**

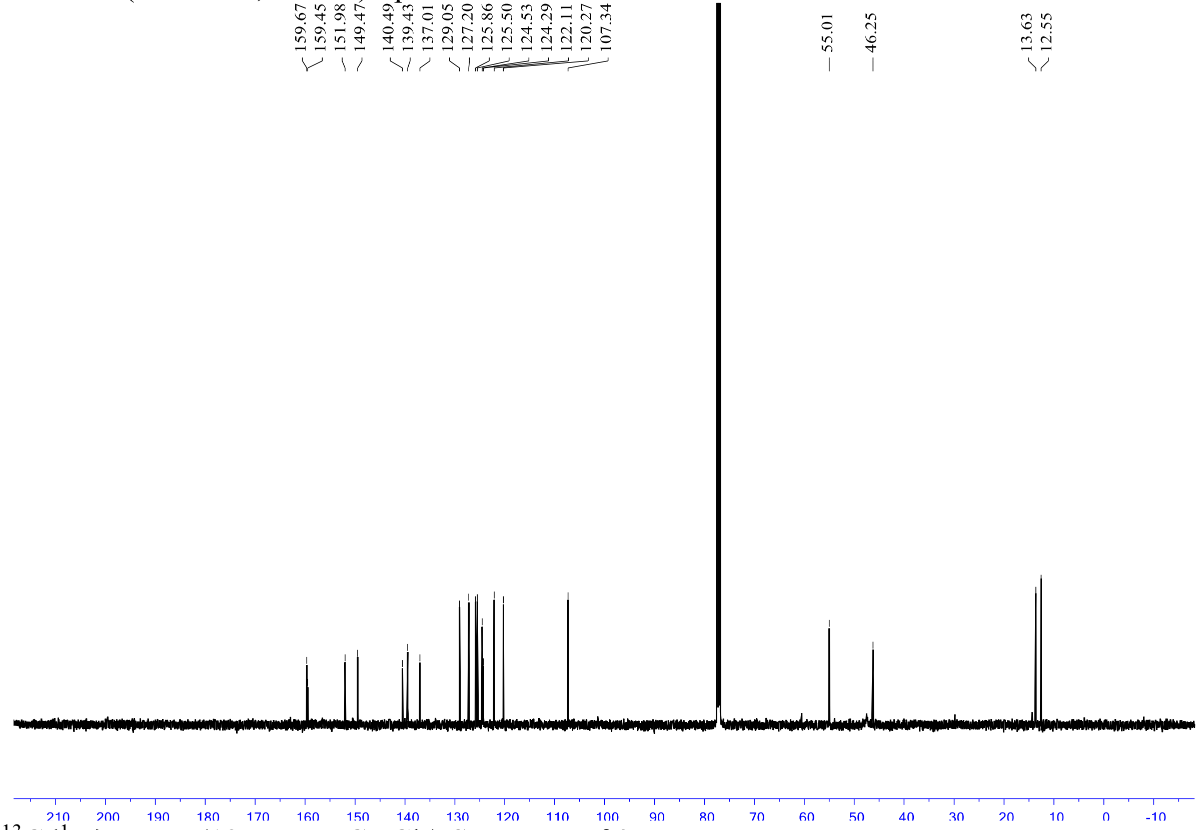

^{13}C{^{1}H} NMR (125 MHz, $CDCl_3$) Spectrum of **3ah**

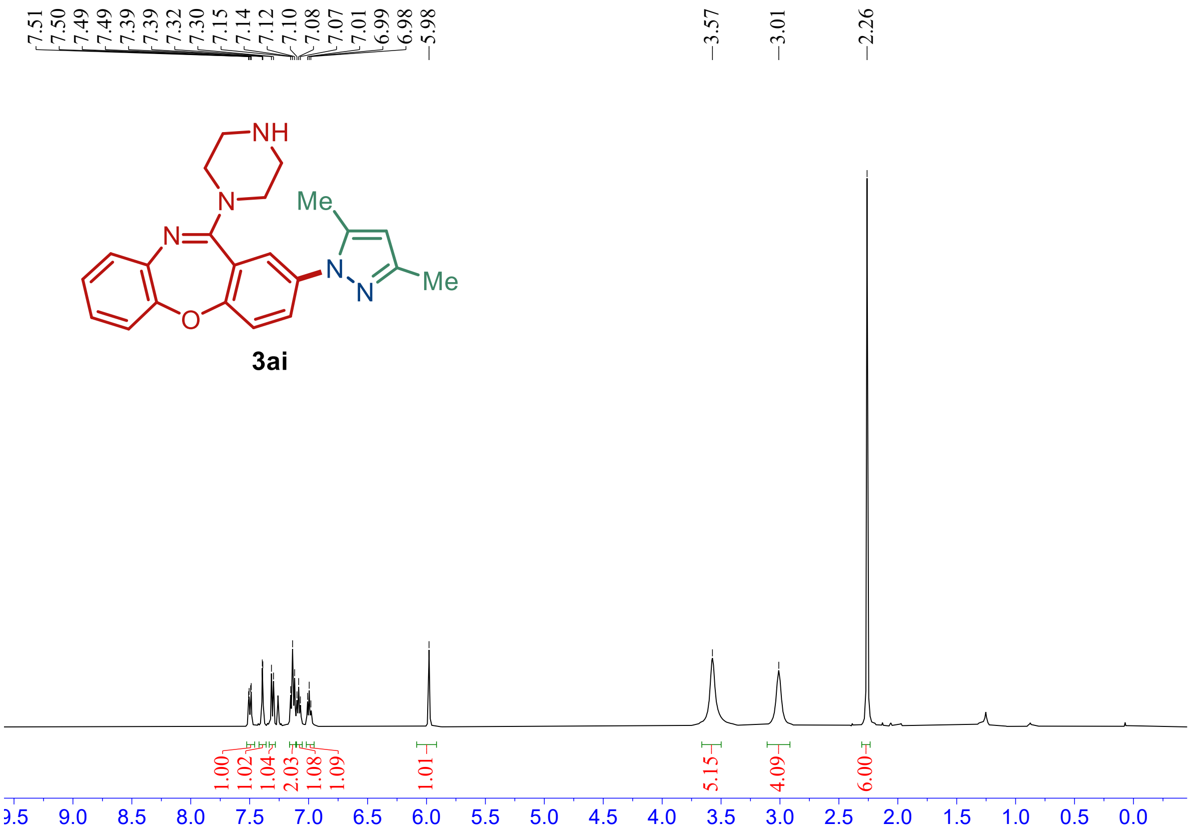

^{1}H NMR (500 MHz, $CDCl_3$) Spectrum of **3ai**

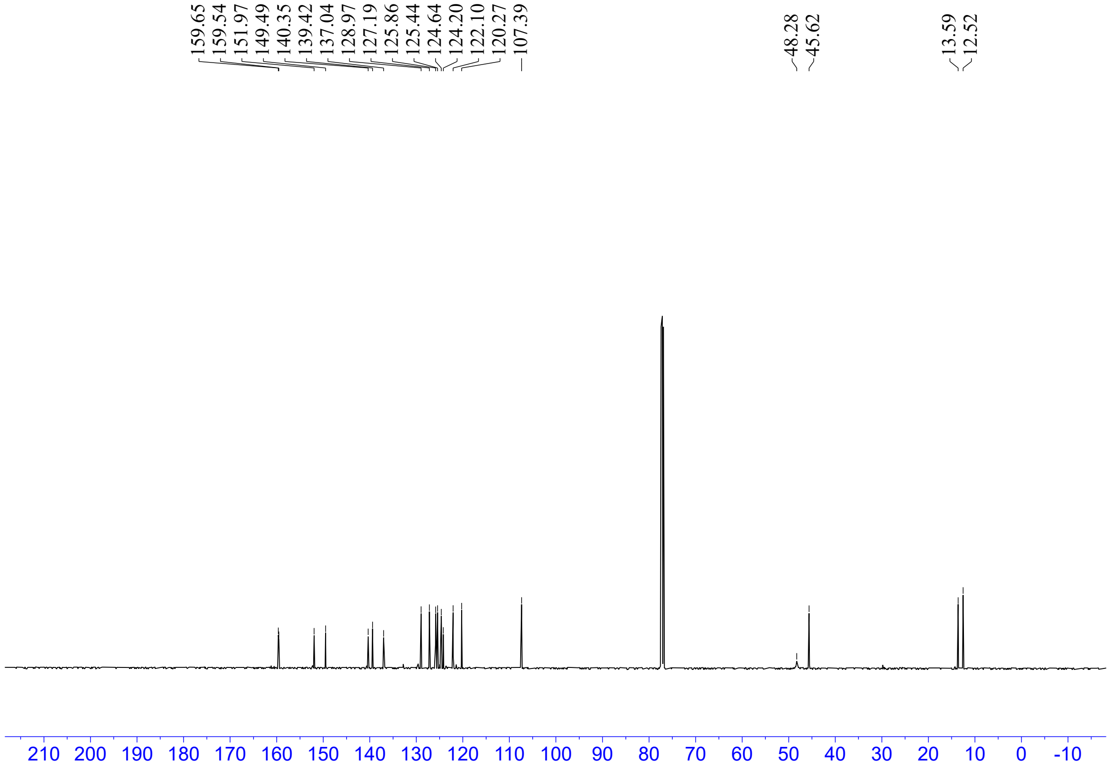

^{13}C{^{1}H} NMR (125 MHz, $CDCl_3$) Spectrum of **3ai**

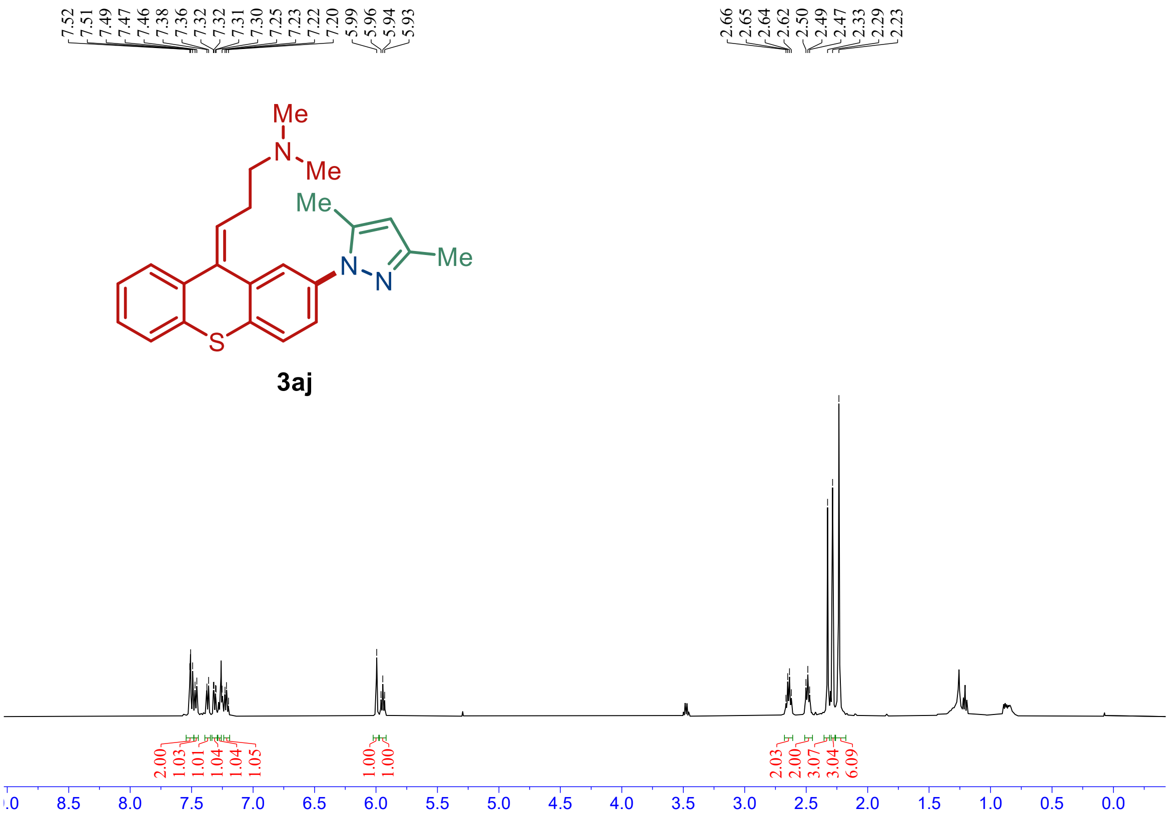

^{1}H NMR (500 MHz, CDCl$_3$) Spectrum of **3aj**

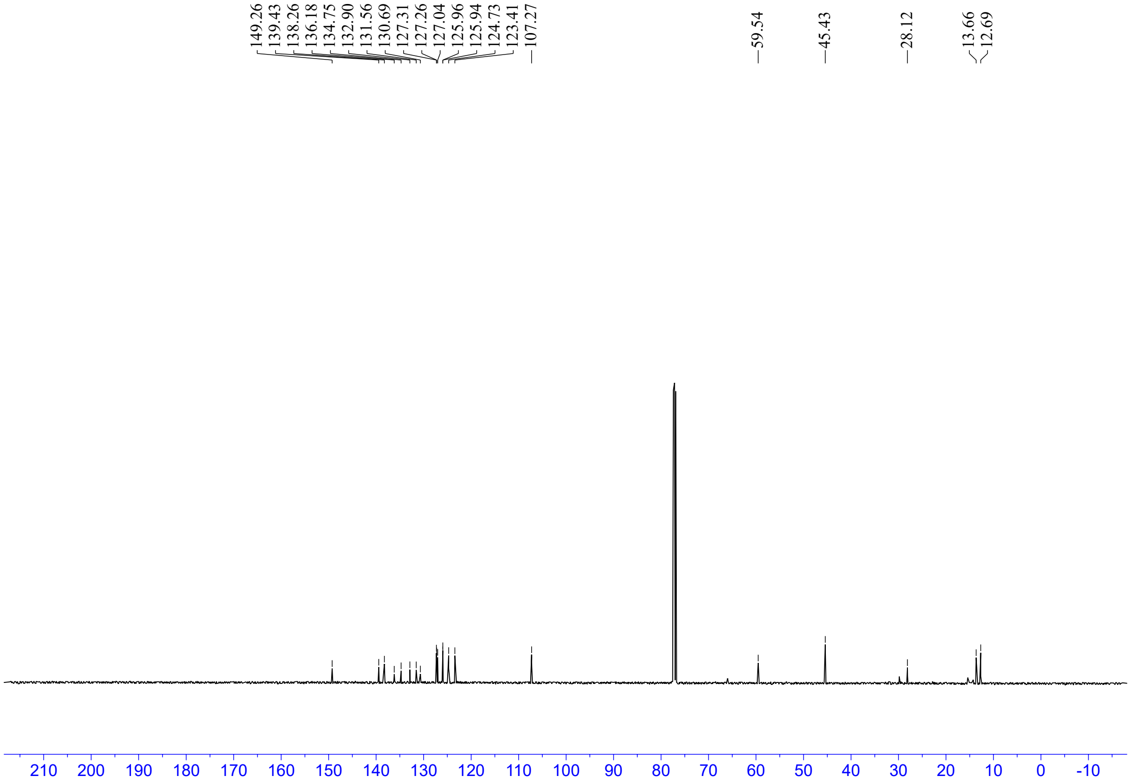

^{13}C{^{1}H} NMR (125 MHz, CDCl$_3$) Spectrum of **3aj**

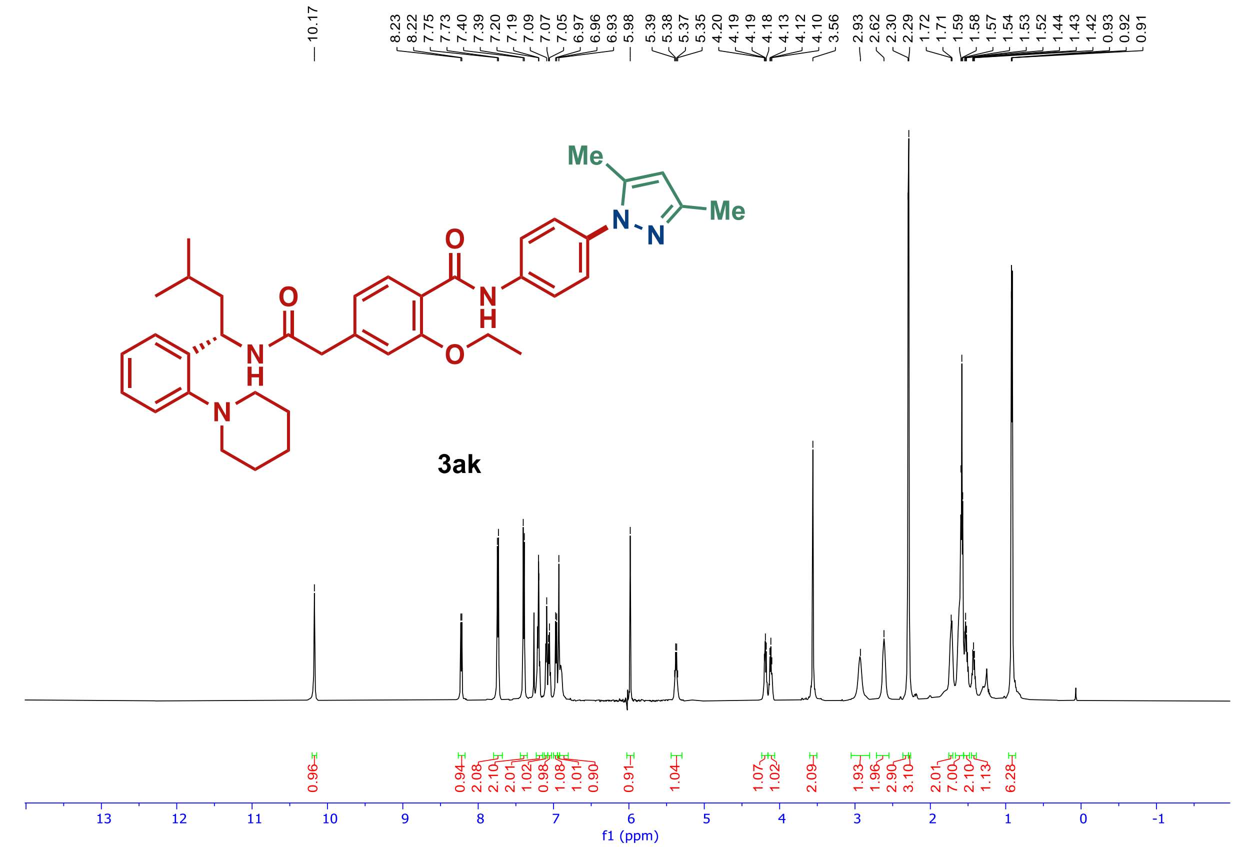

^{1}H NMR (600 MHz, $CDCl_3$) Spectrum of **3ak**

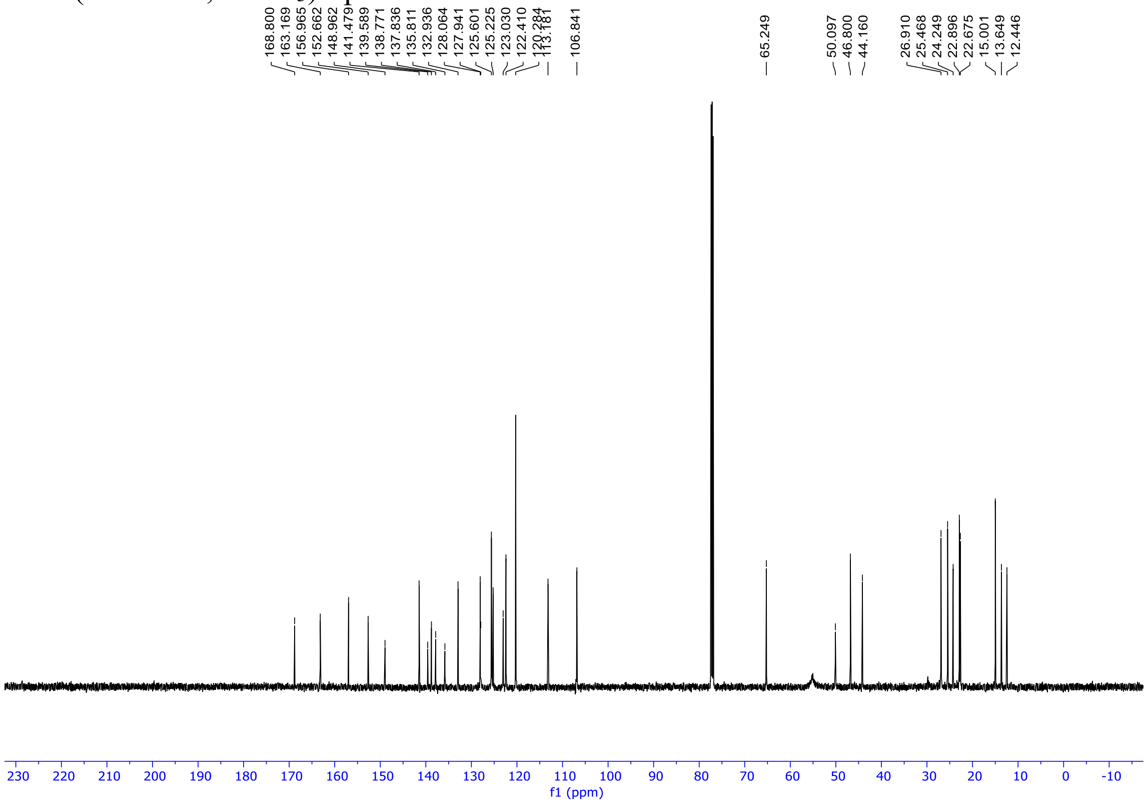

^{13}C{^{1}H} NMR (125 MHz, $CDCl_3$) Spectrum of **3ak**

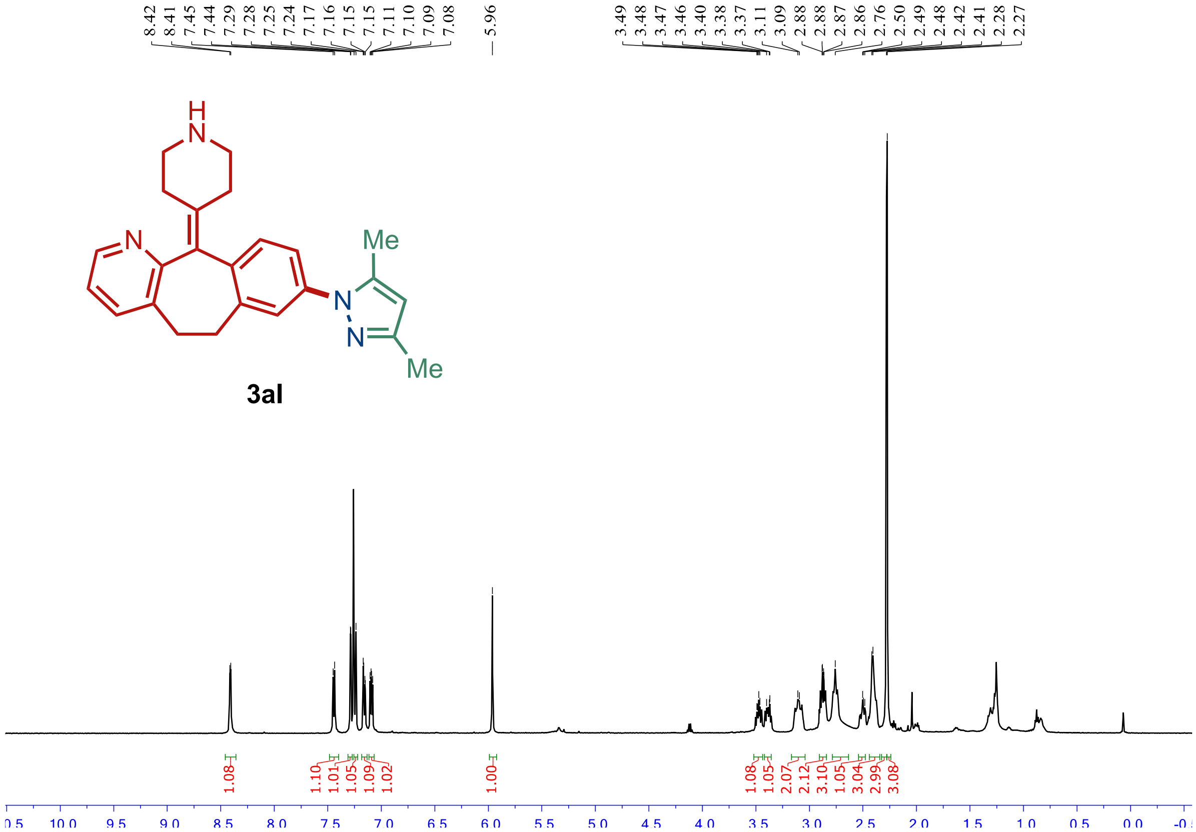

^{1}H NMR (500 MHz, CDCl$_3$) Spectrum of **3al**

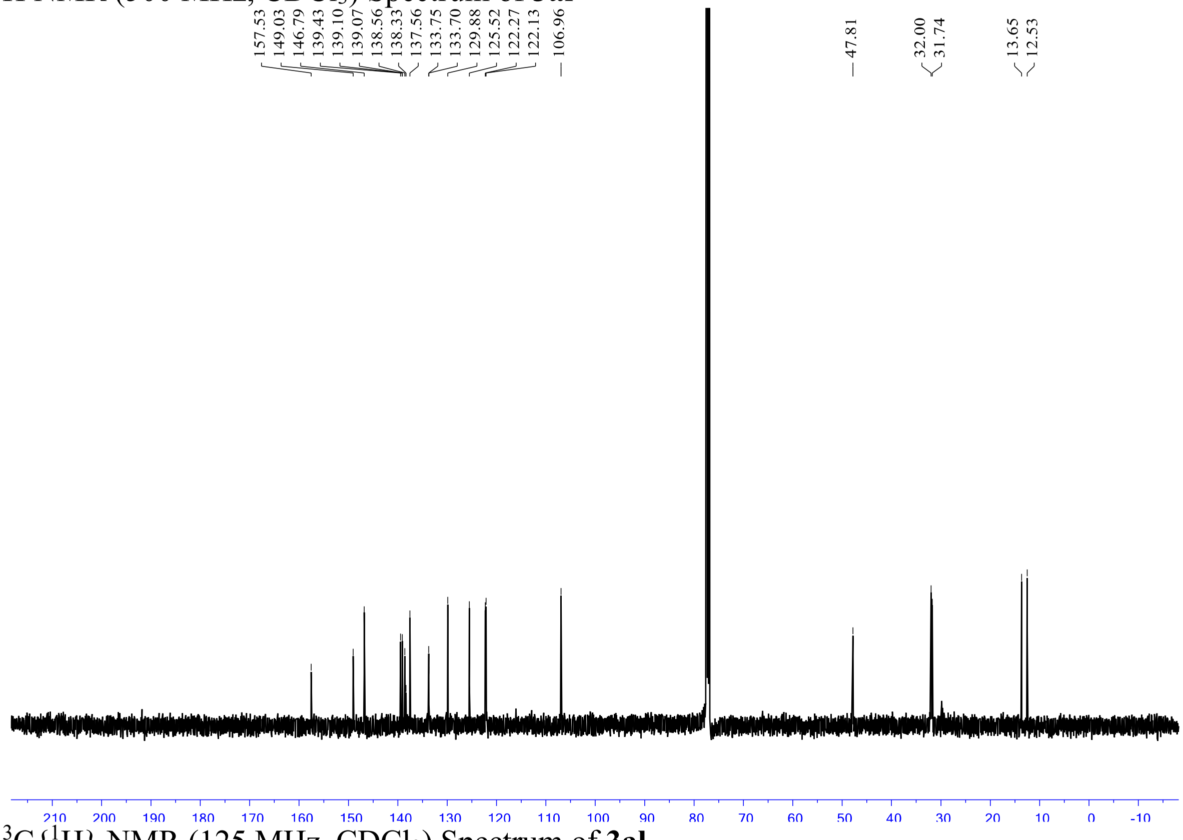

^{13}C{^{1}H} NMR (125 MHz, CDCl$_3$) Spectrum of **3al**

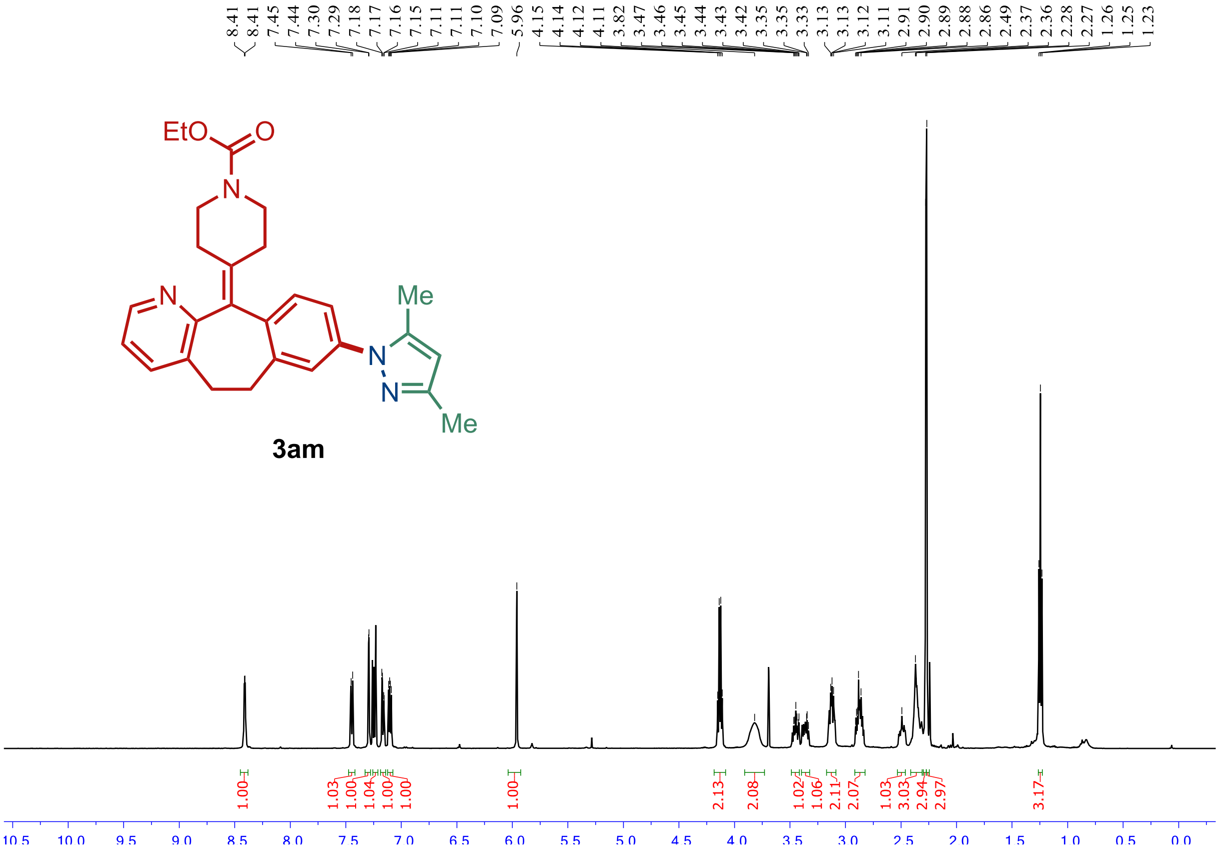

^{1}H NMR (500 MHz, CDCl$_3$) Spectrum of **3am**

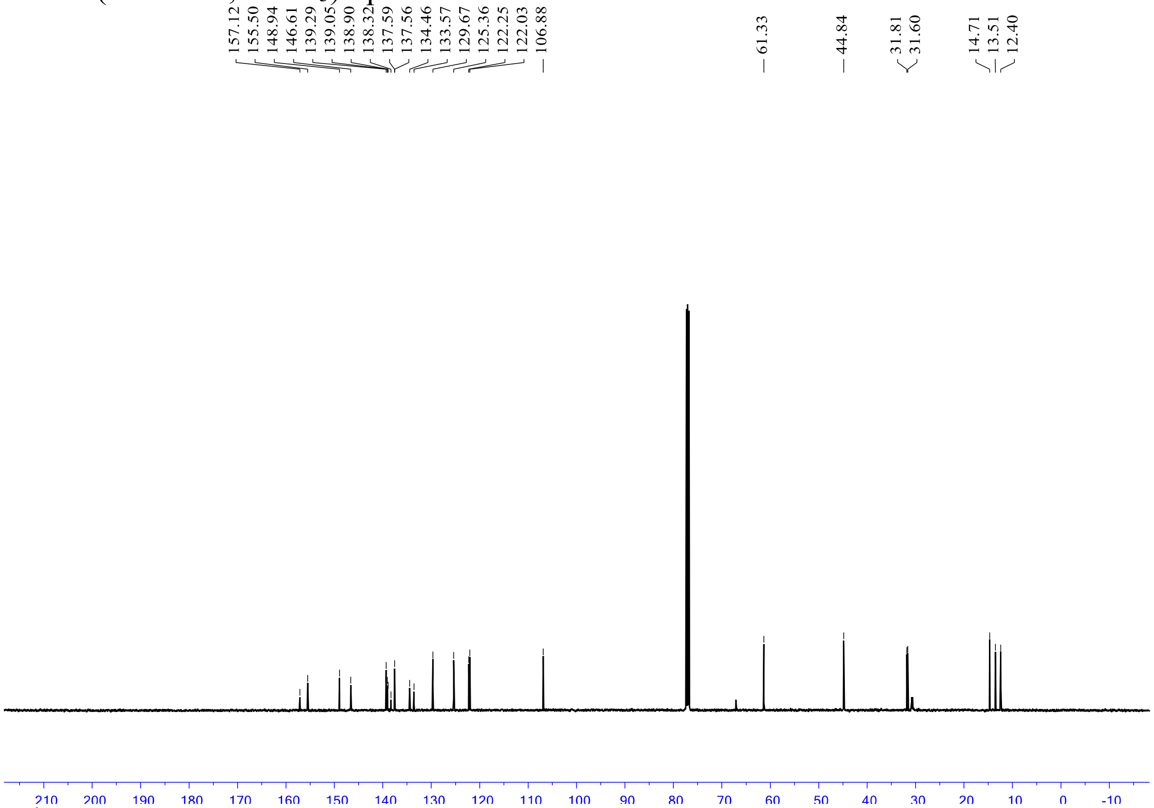

^{13}C{^{1}H} NMR (125 MHz, CDCl$_3$) Spectrum of **3am**

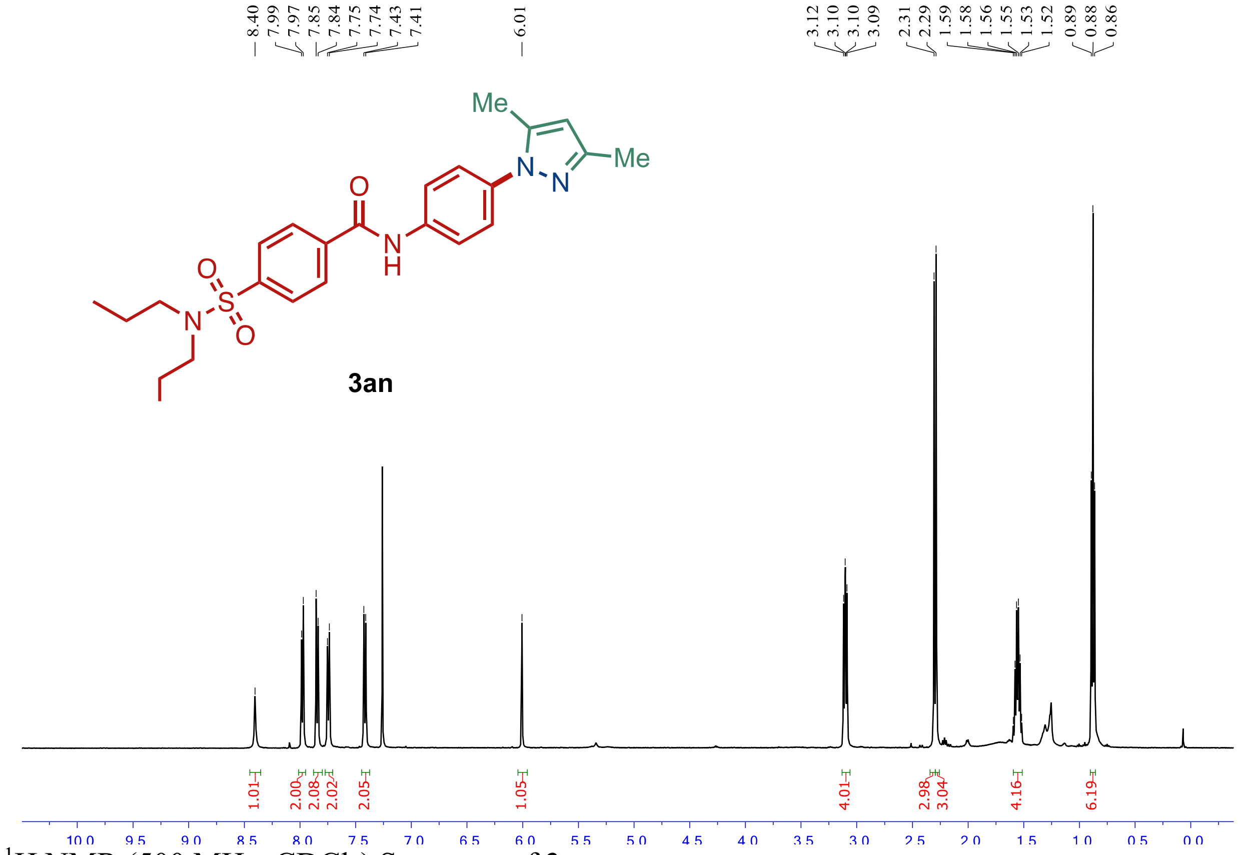

^{1}H NMR (500 MHz, CDCl$_3$) Spectrum of **3an**

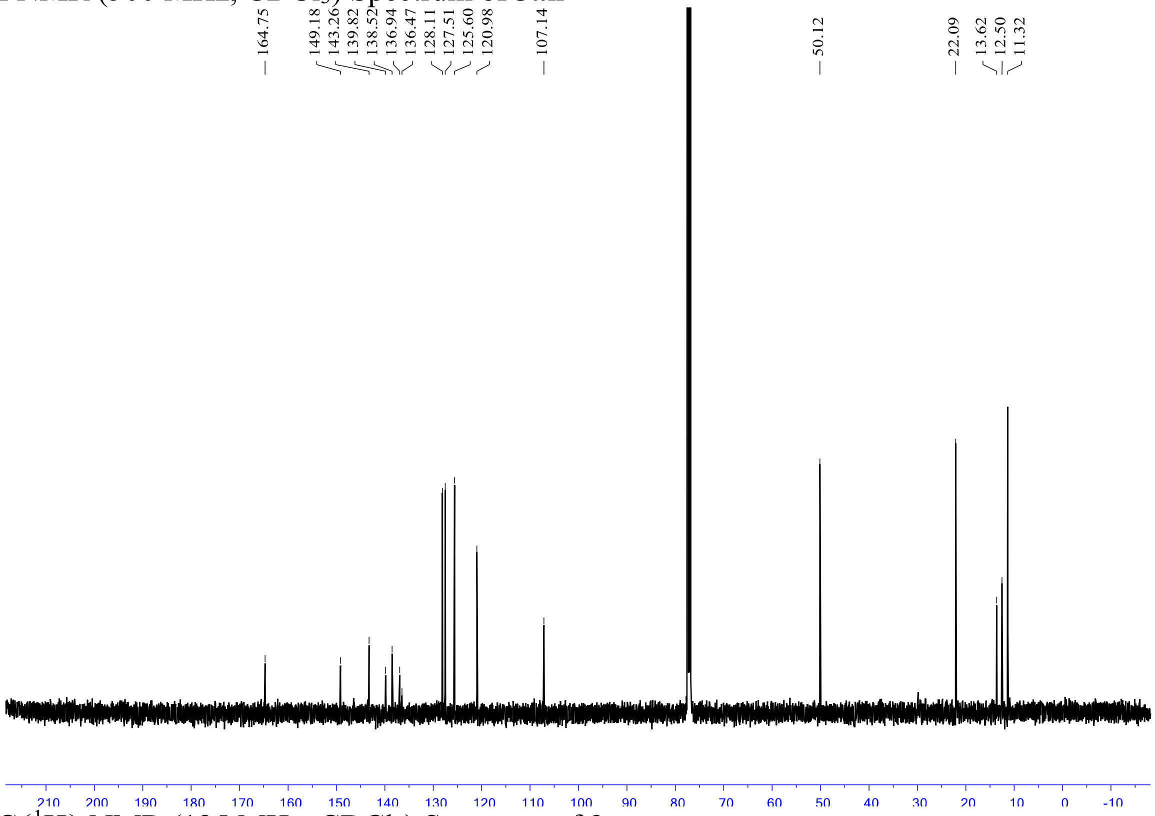

^{13}C{^{1}H} NMR (125 MHz, CDCl$_3$) Spectrum of **3an**

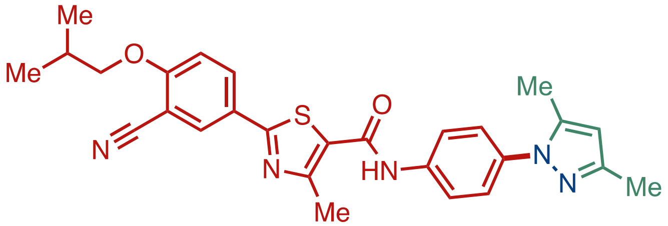

3ao

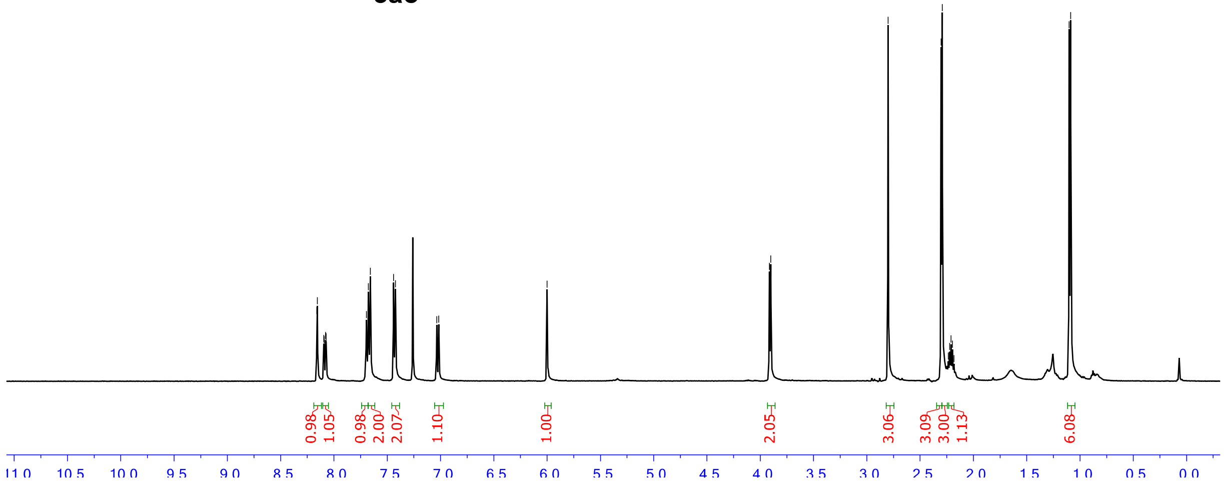

^{1}H NMR (500 MHz, $CDCl_3$) Spectrum of **3ao**

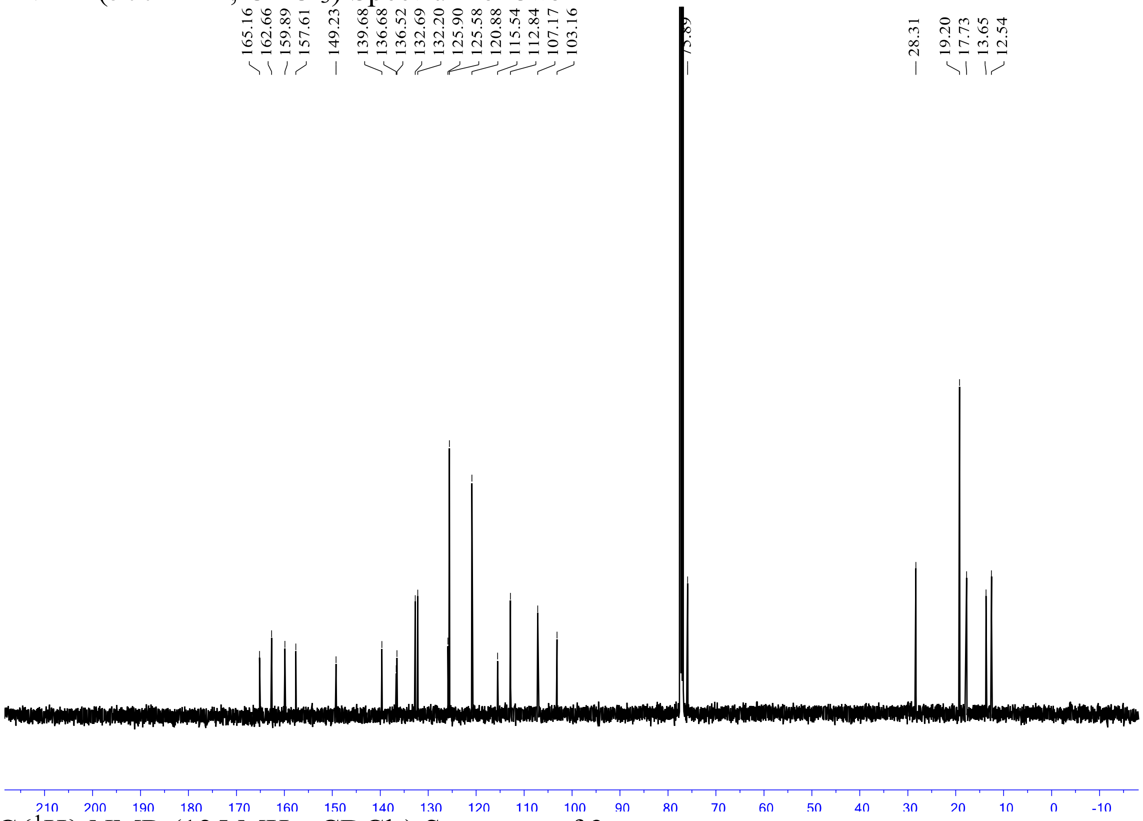

^{13}C{^{1}H} NMR (125 MHz, $CDCl_3$) Spectrum of **3ao**

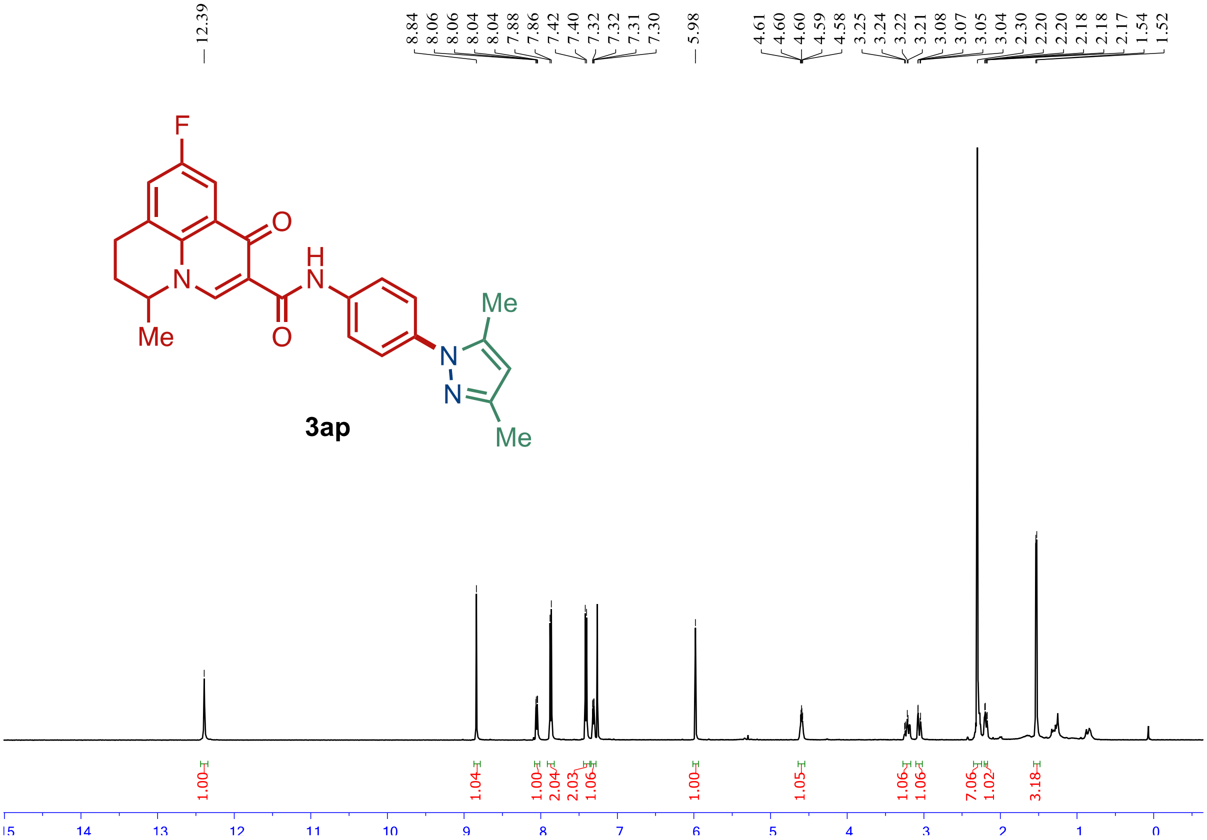

^{1}H NMR (500 MHz, CDCl$_3$) Spectrum of **3ap**

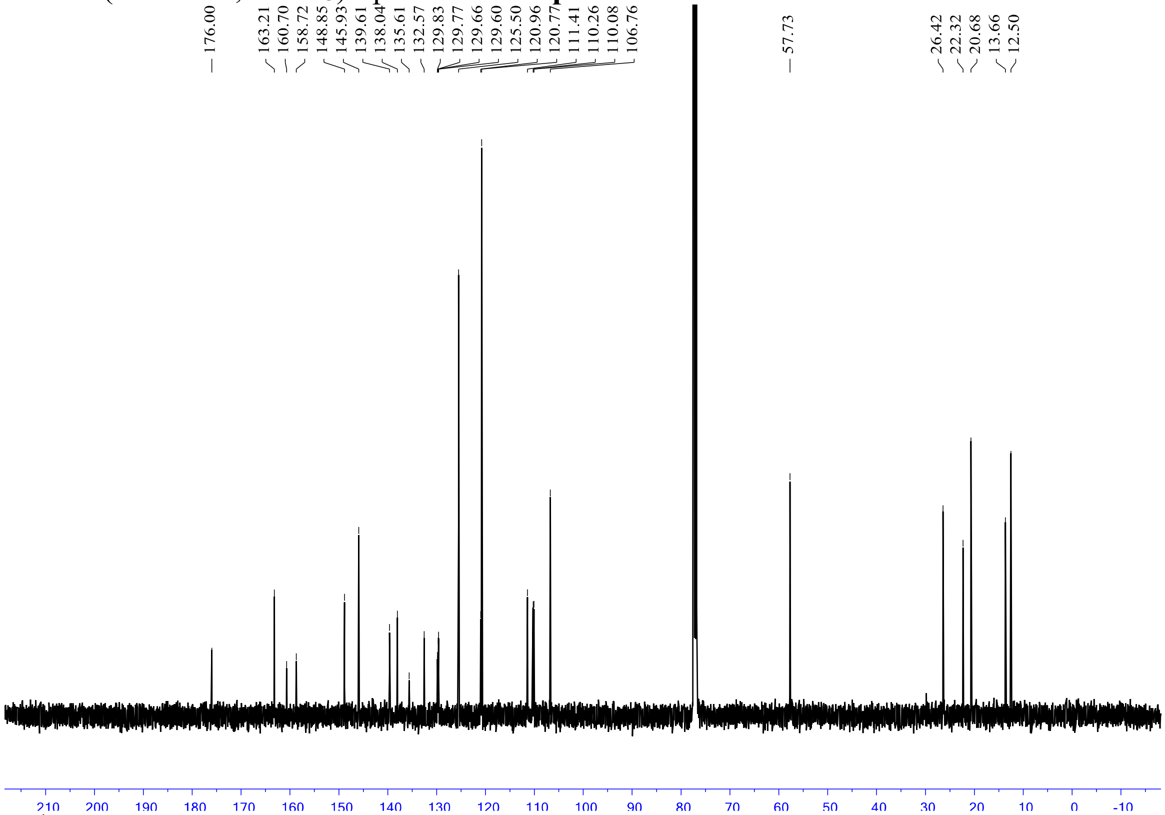

^{13}C{^{1}H} NMR (125 MHz, CDCl$_3$) Spectrum of **3ap**

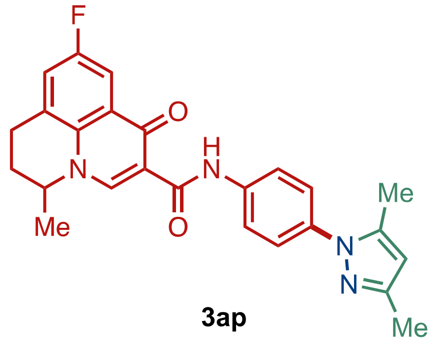

3ap

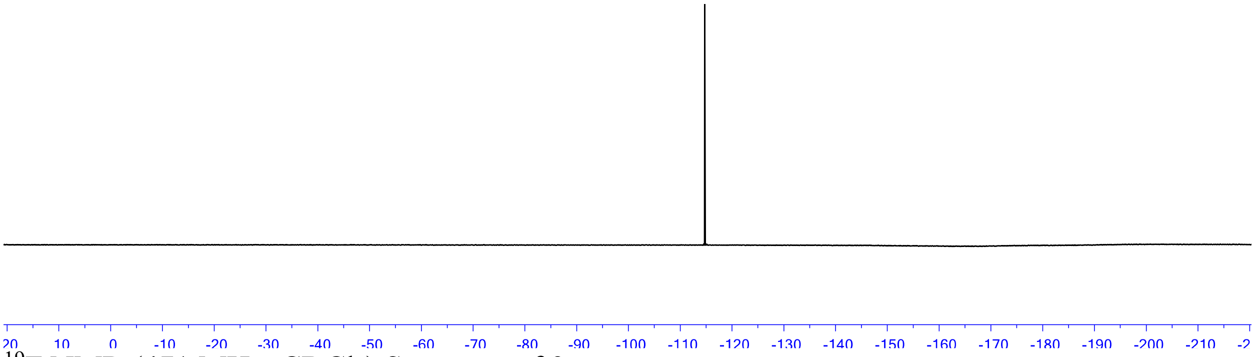

^{19}F NMR (471 MHz, $CDCl_3$) Spectrum of **3ap**

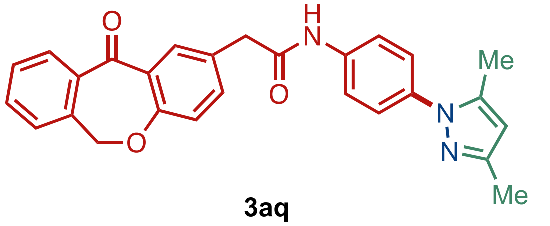

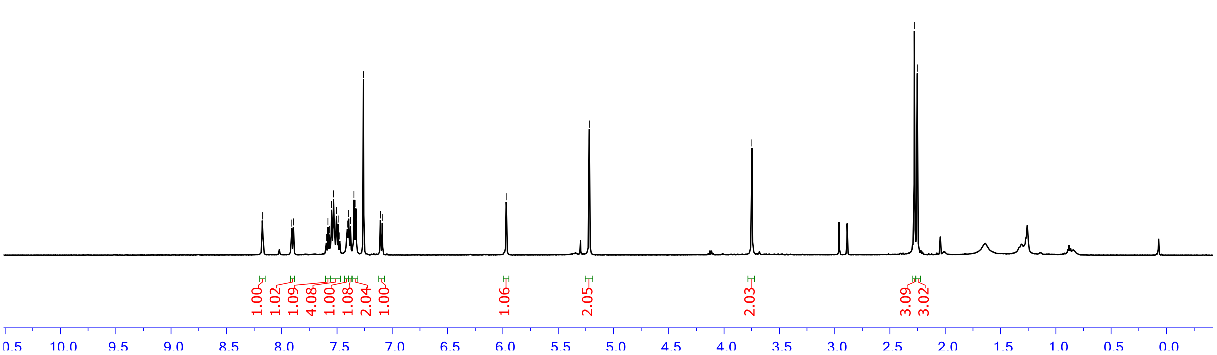

^{1}H NMR (500 MHz, CDCl$_3$) Spectrum of **3aq**

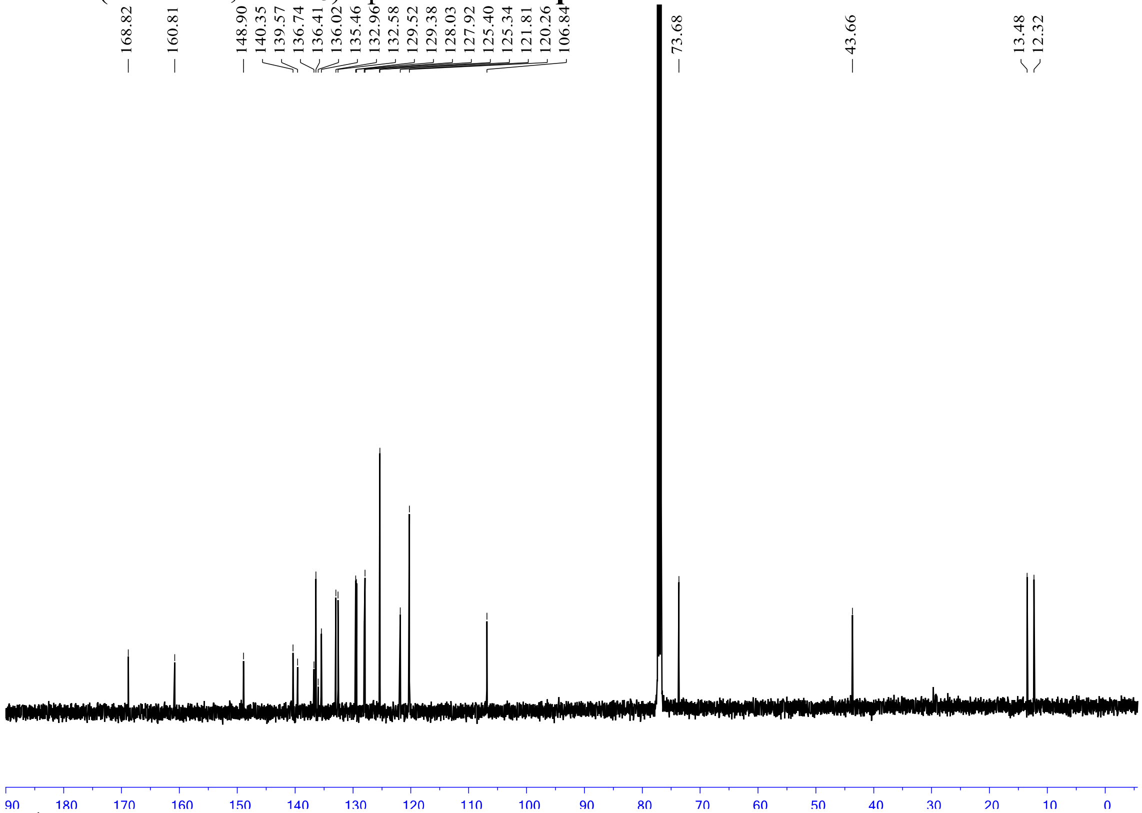

^{13}C{^{1}H} NMR (125 MHz, CDCl$_3$) Spectrum of **3aq**

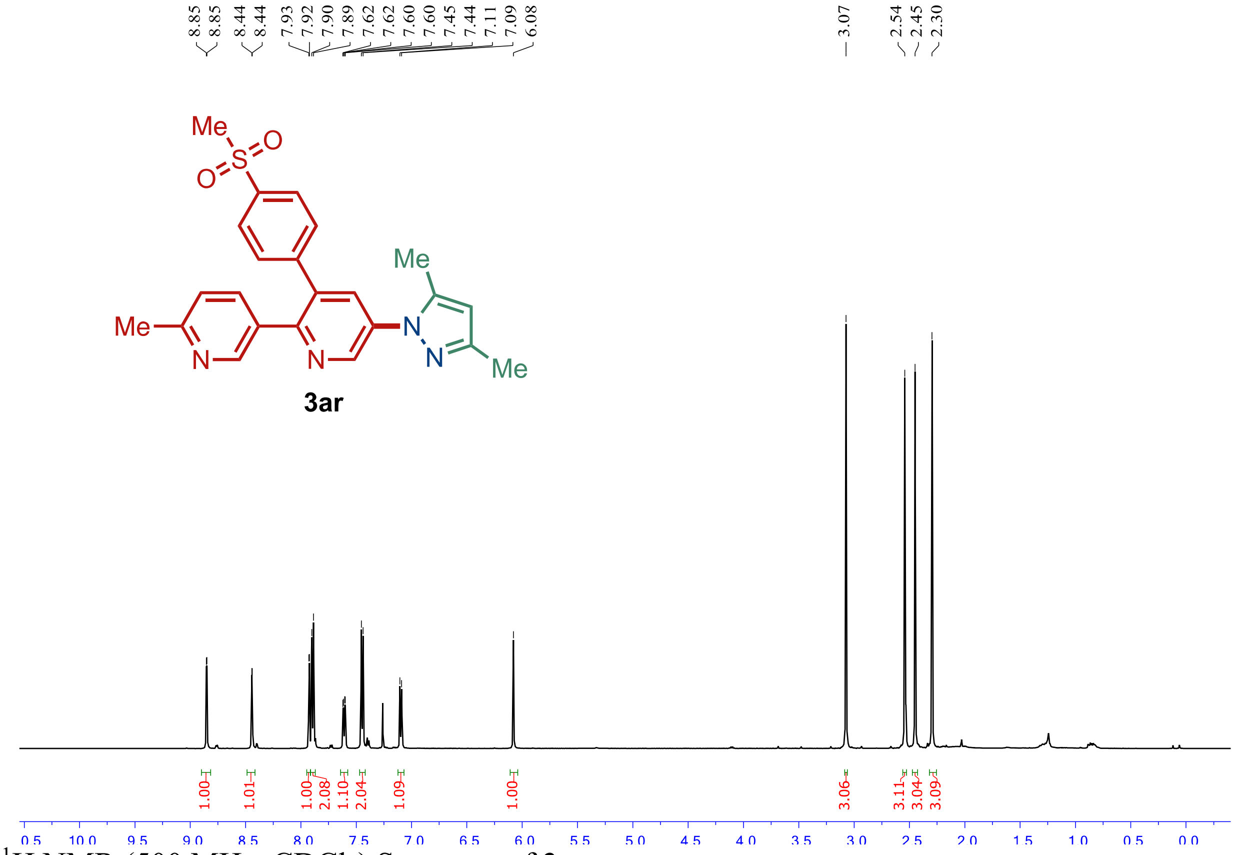

^{1}H NMR (500 MHz, $CDCl_3$) Spectrum of **3ar**

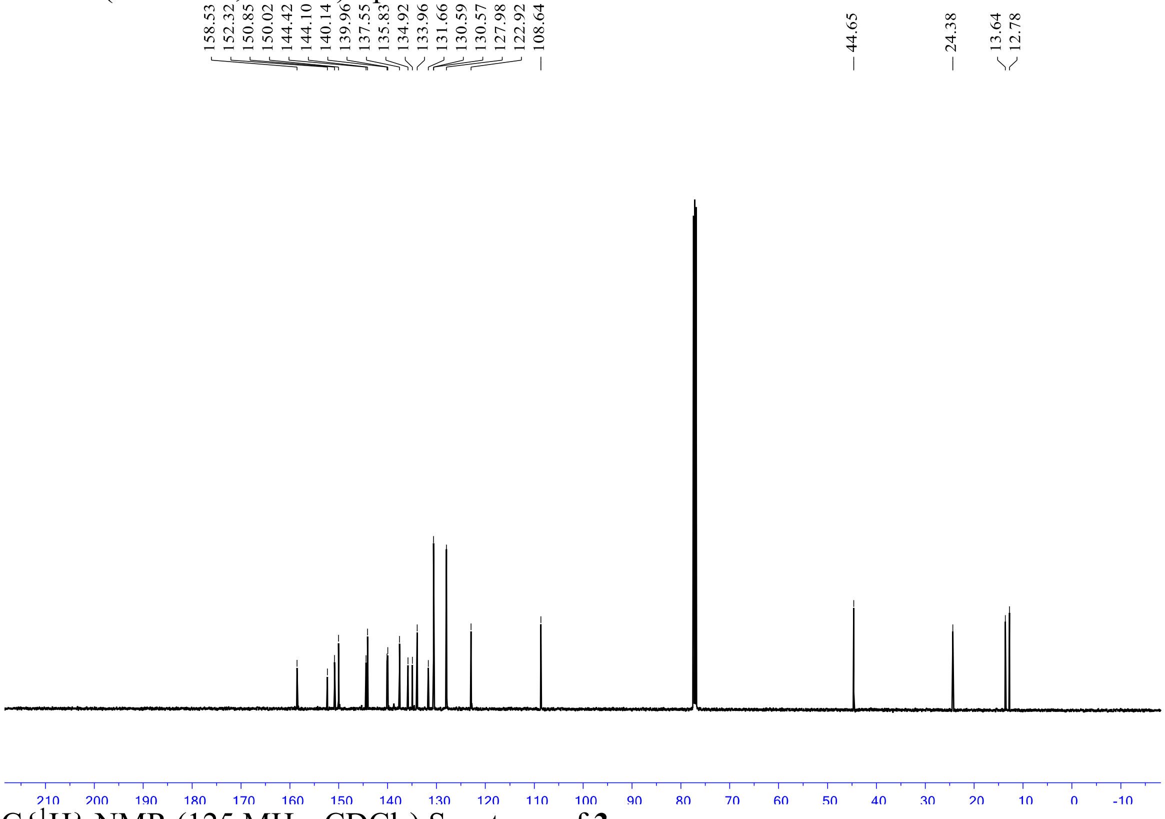

^{13}C{^{1}H} NMR (125 MHz, $CDCl_3$) Spectrum of **3ar**

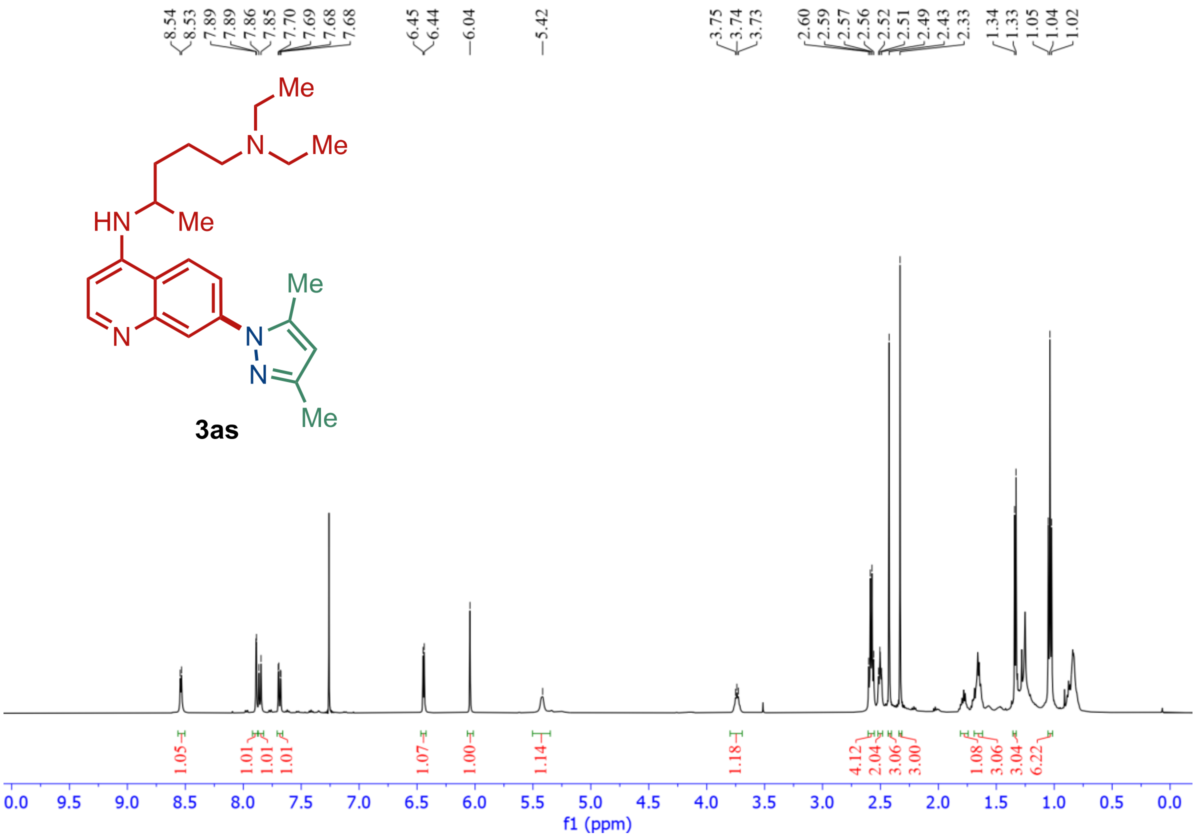

^{1}H NMR (500 MHz, $CDCl_3$) Spectrum of **3as**

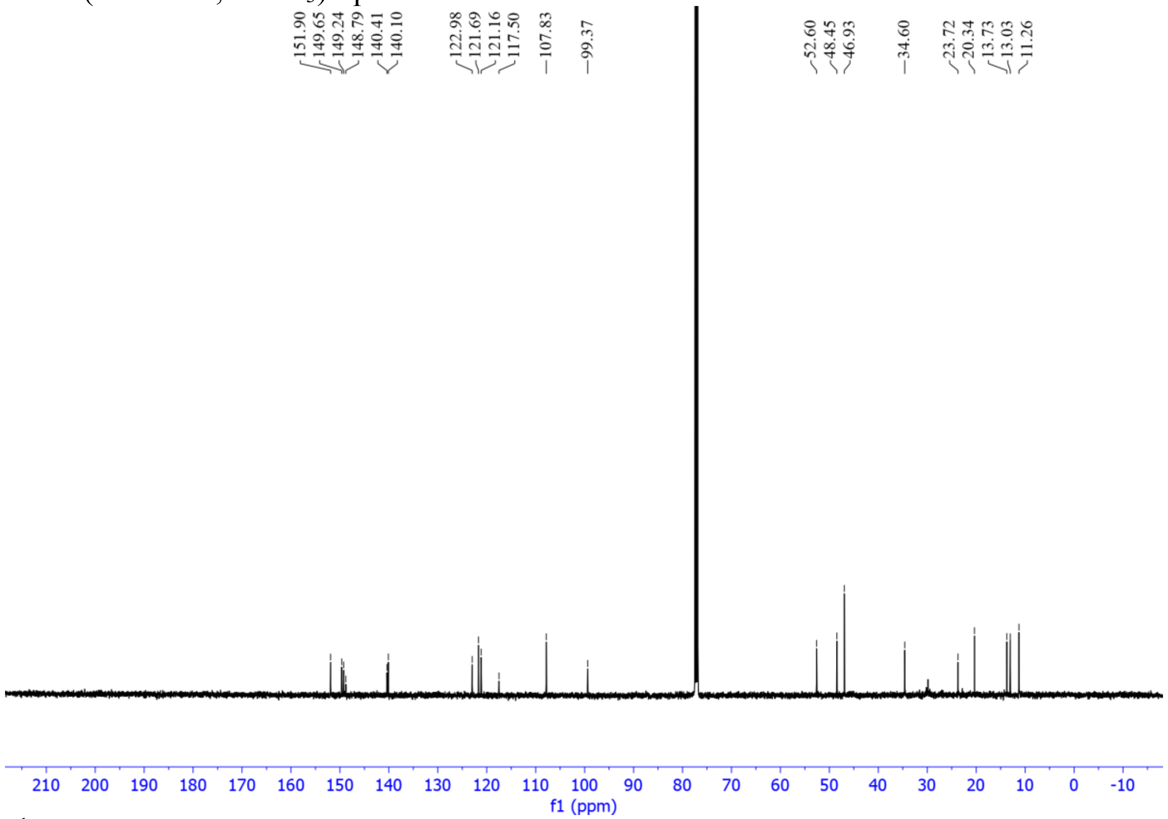

^{13}C{^{1}H} NMR (125 MHz, $CDCl_3$) Spectrum of **3as**

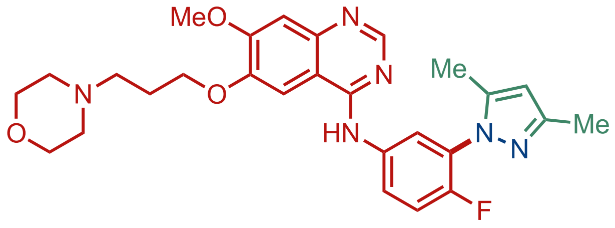

3at

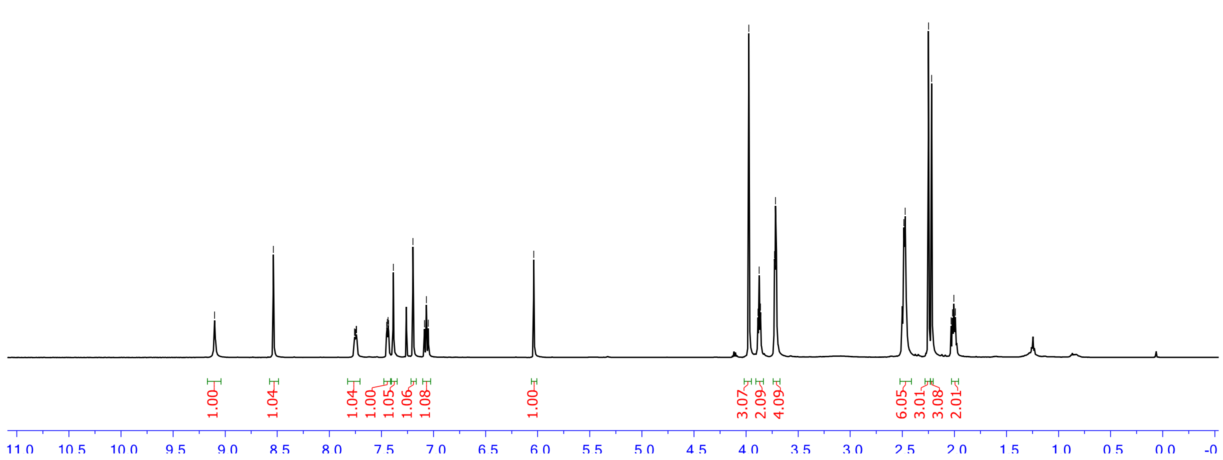

^{1}H NMR (500 MHz, $CDCl_3$) Spectrum of **3at**

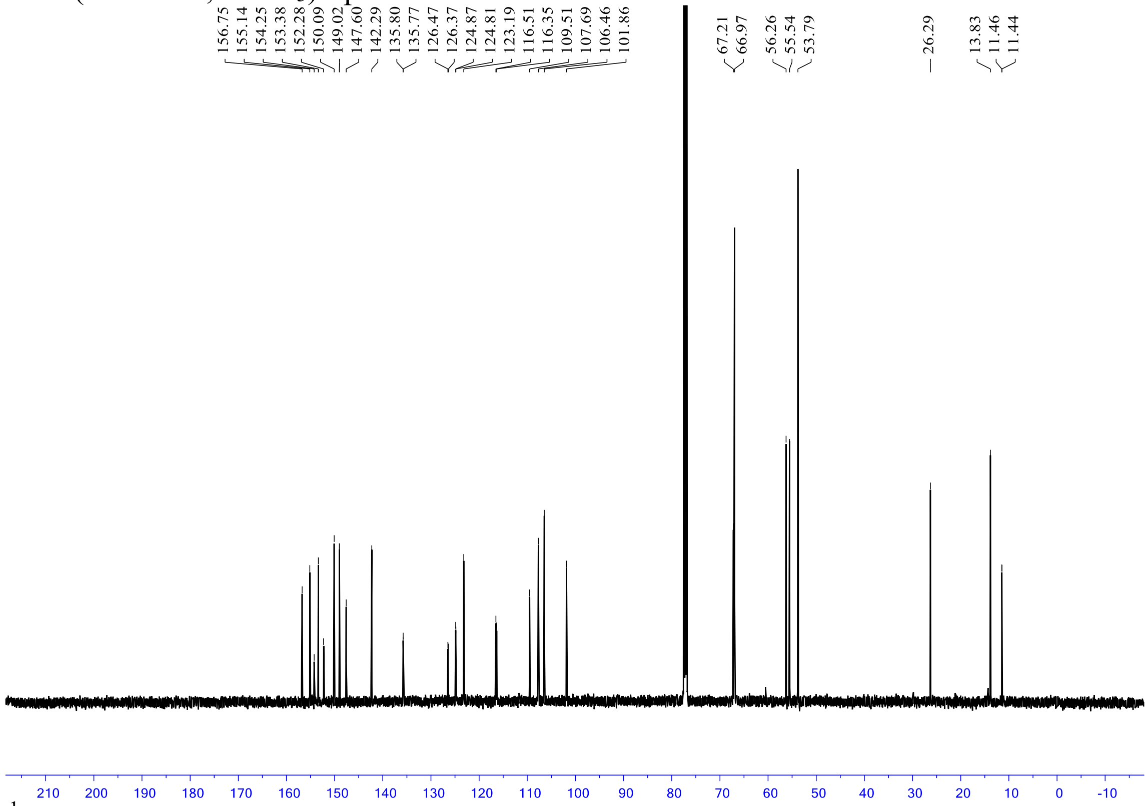

^{13}C{^{1}H} NMR (125 MHz, $CDCl_3$) Spectrum of **3at**

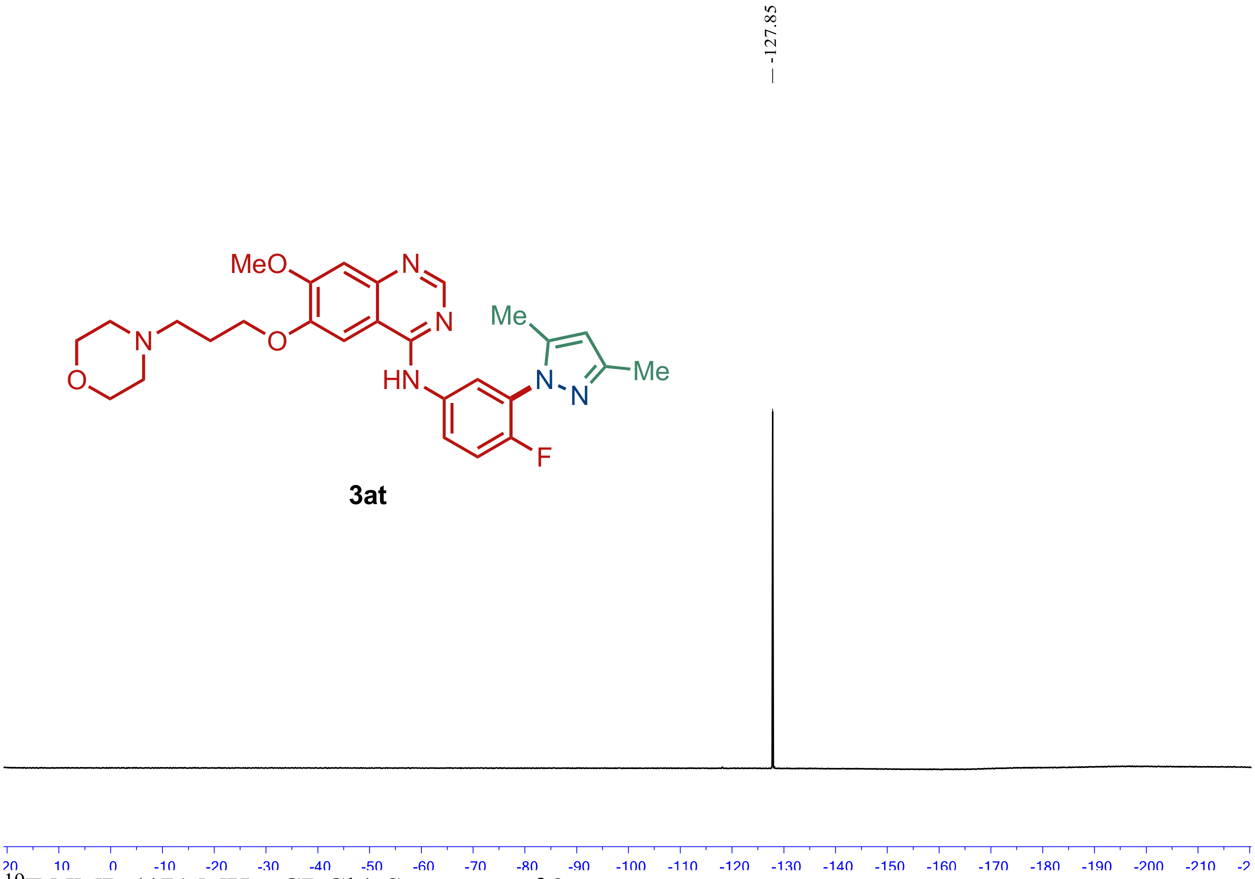

^{19}F NMR (471 MHz, $CDCl_3$) Spectrum of **3at**

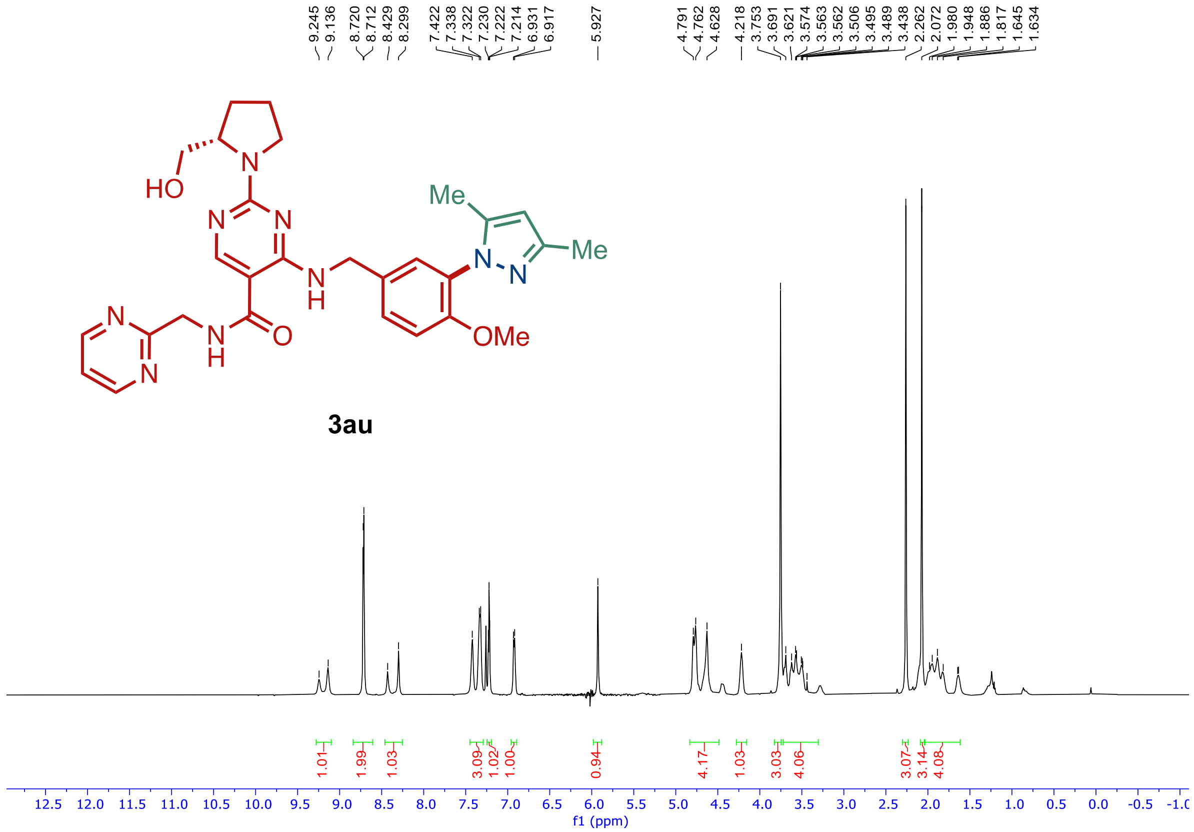

^{1}H NMR (600 MHz, CDCl$_3$) Spectrum of **3au**

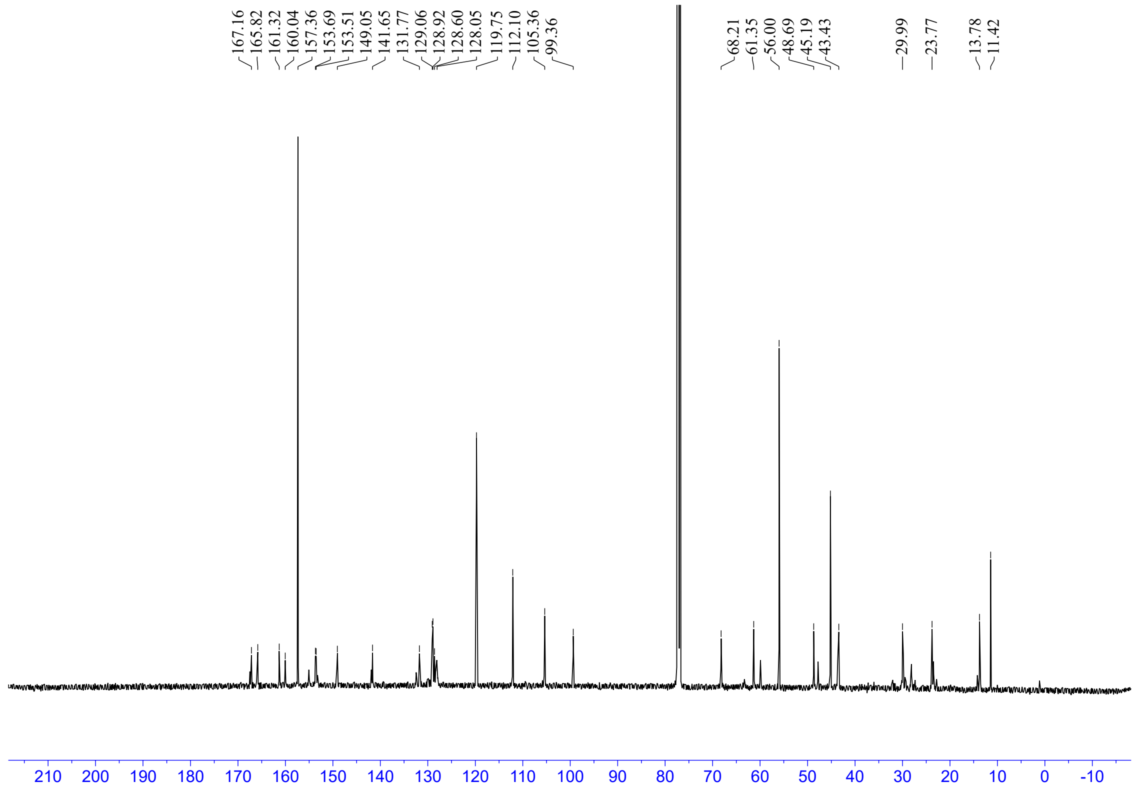

^{13}C{^{1}H} NMR (125 MHz, CDCl$_3$) Spectrum of **3au**

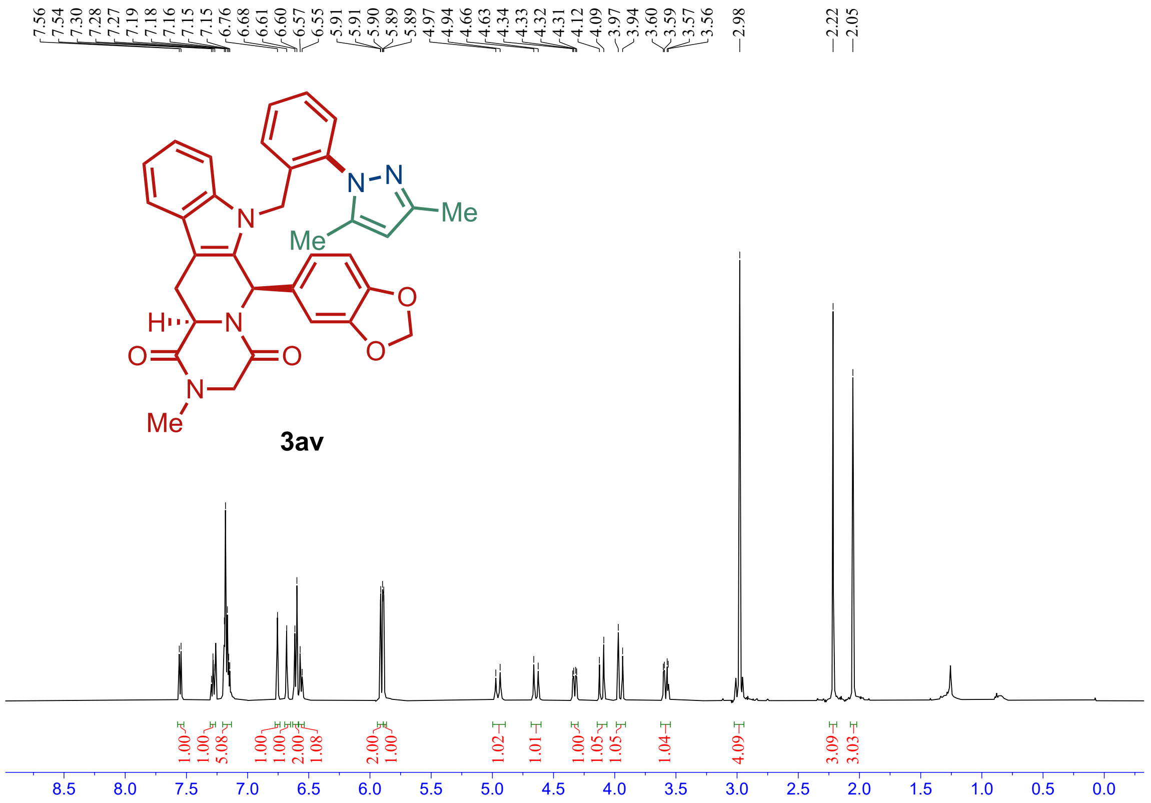

^{1}H NMR (500 MHz, $CDCl_3$) Spectrum of **3av**

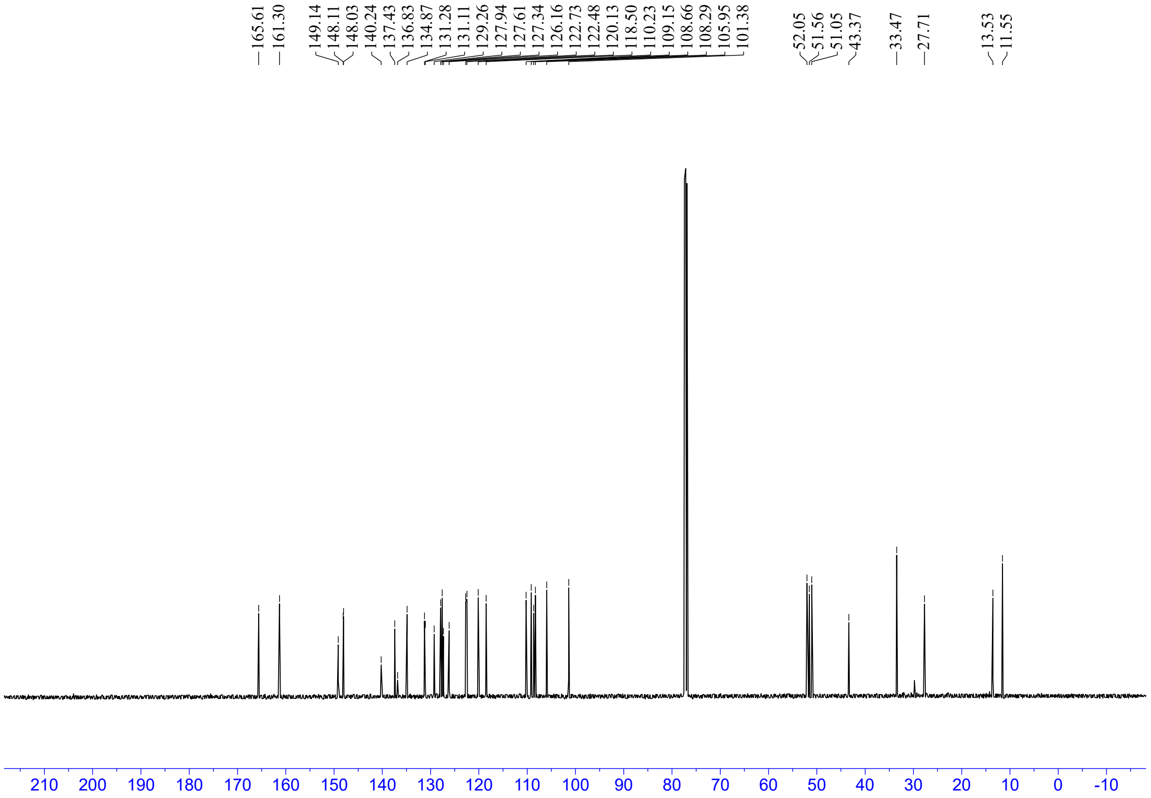

^{13}C{^{1}H} NMR (125 MHz, $CDCl_3$) Spectrum of **3av**

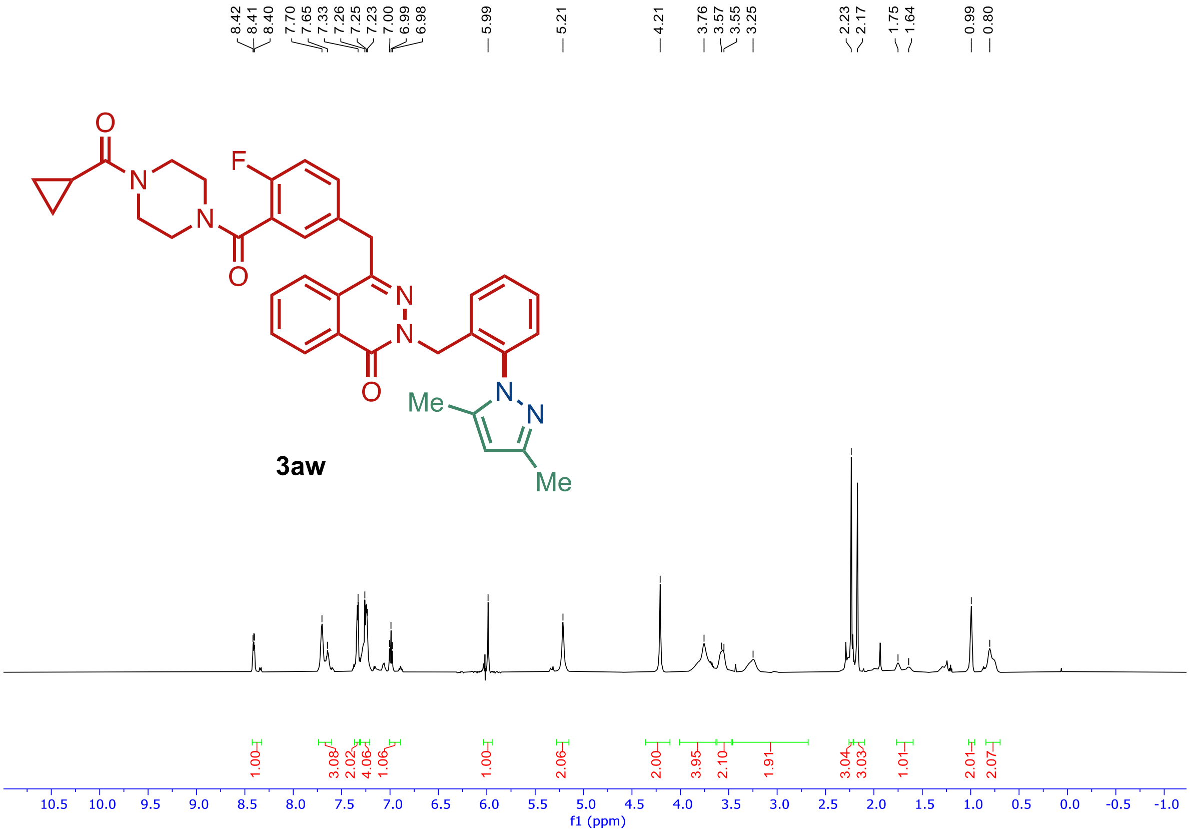

^{1}H NMR (600 MHz, $CDCl_3$) Spectrum of **3aw**

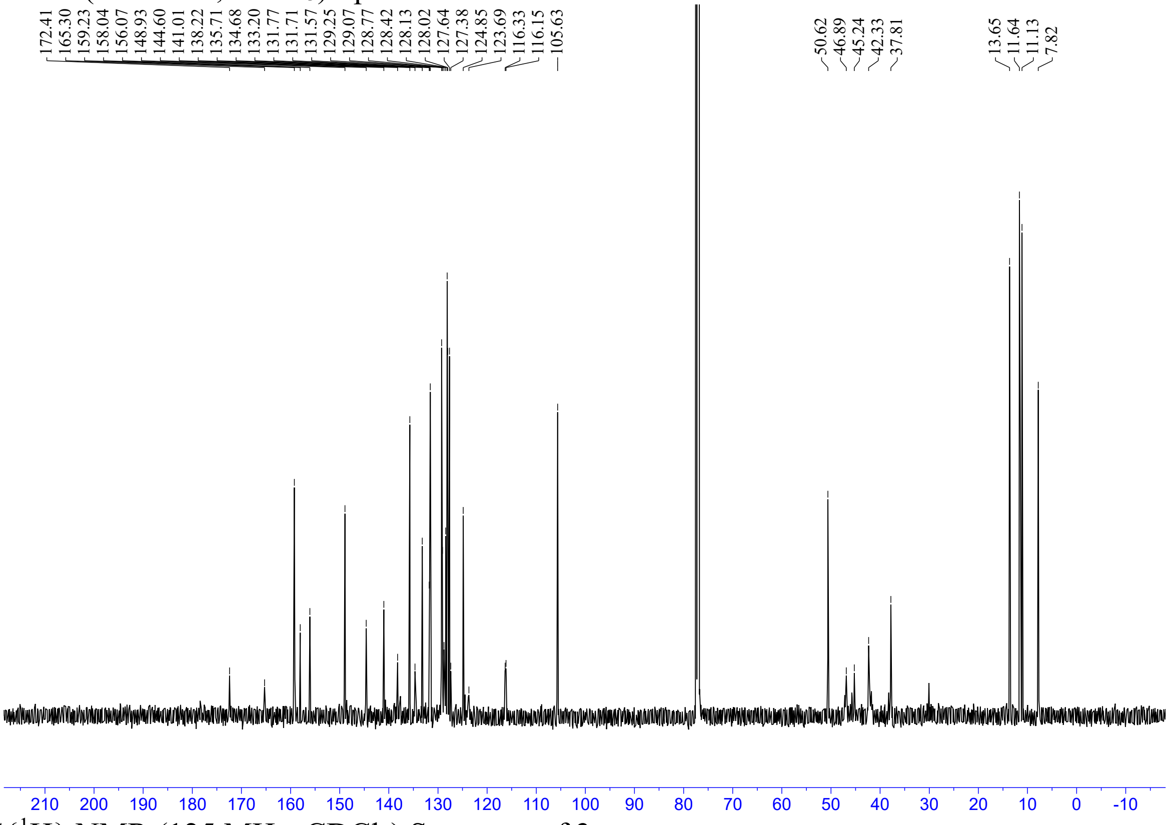

^{13}C{^{1}H} NMR (125 MHz, $CDCl_3$) Spectrum of **3aw**

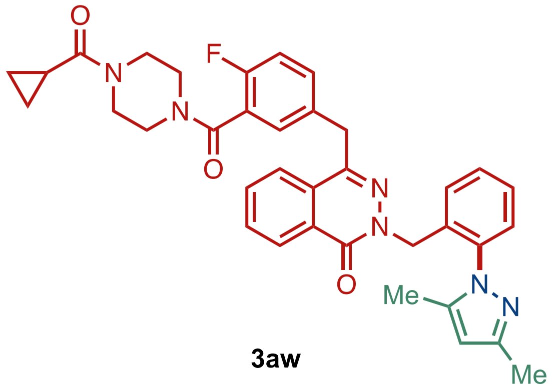

3aw

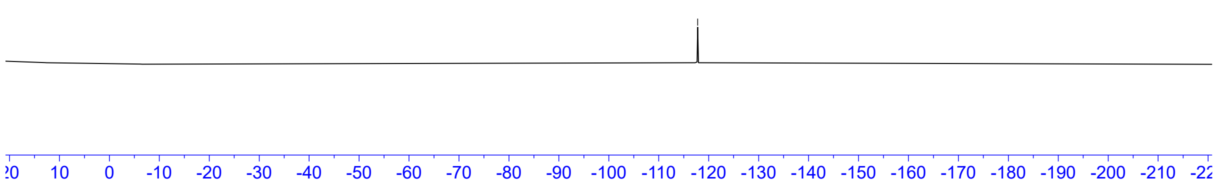

^{19}F NMR (471 MHz, $CDCl_3$) Spectrum of **3aw**

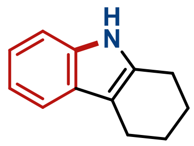

4a

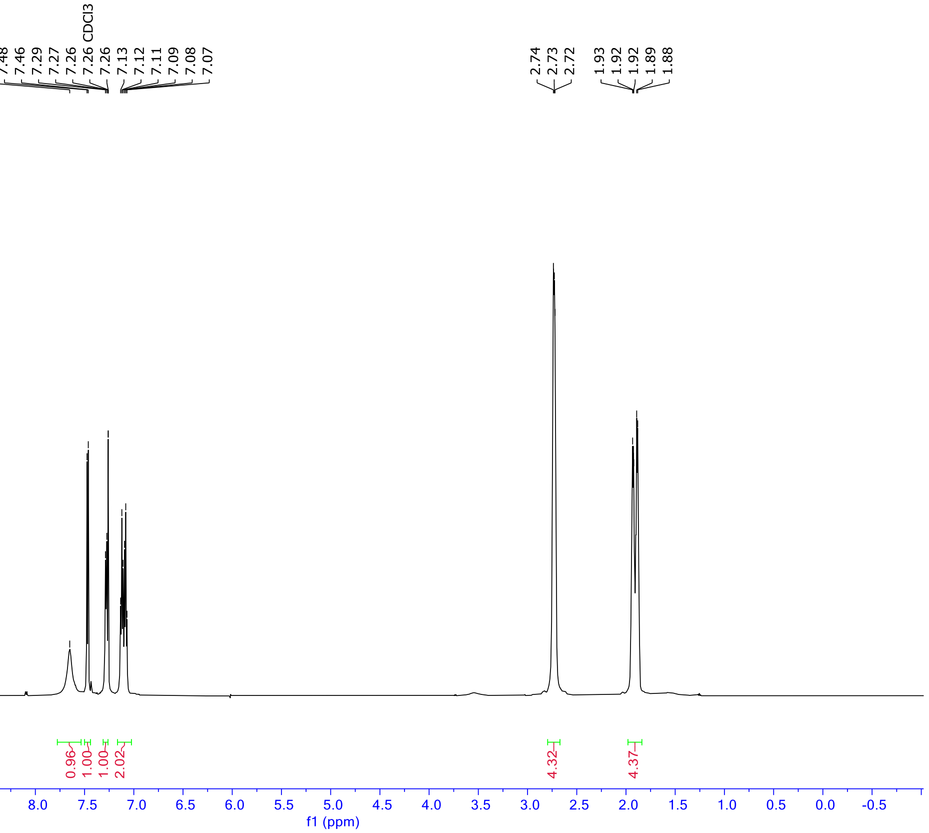

^{1}H NMR (600 MHz, CDCl$_3$) Spectrum of **4a**

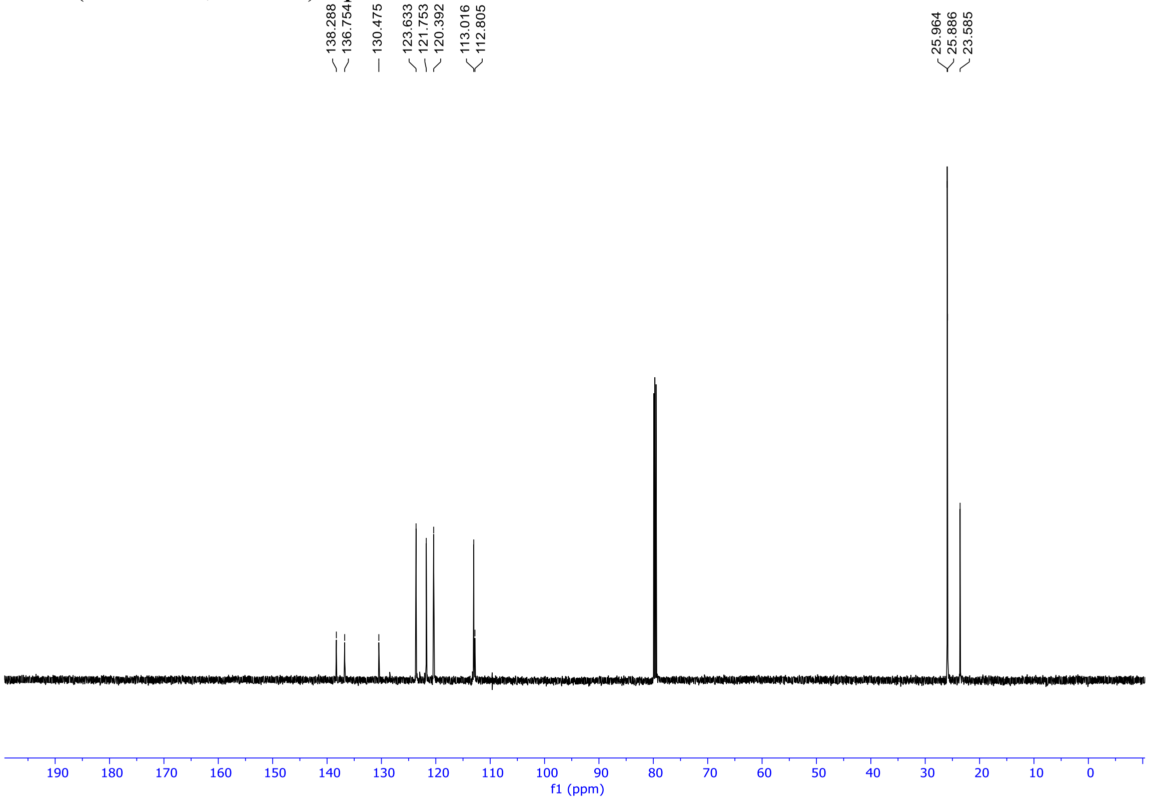

^{13}C{^{1}H} NMR (151 MHz, CDCl$_3$) Spectrum of **4a**

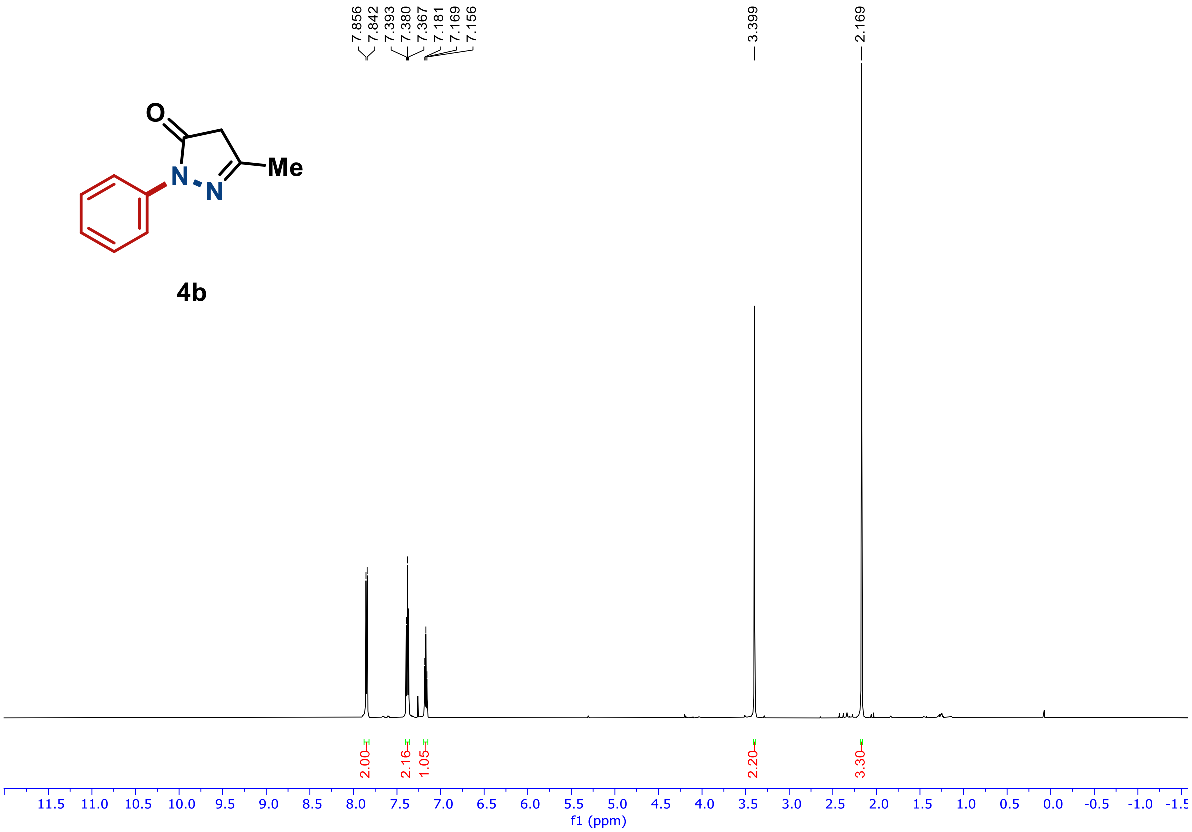

^{1}H NMR (600 MHz, CDCl$_3$) Spectrum of **4b**

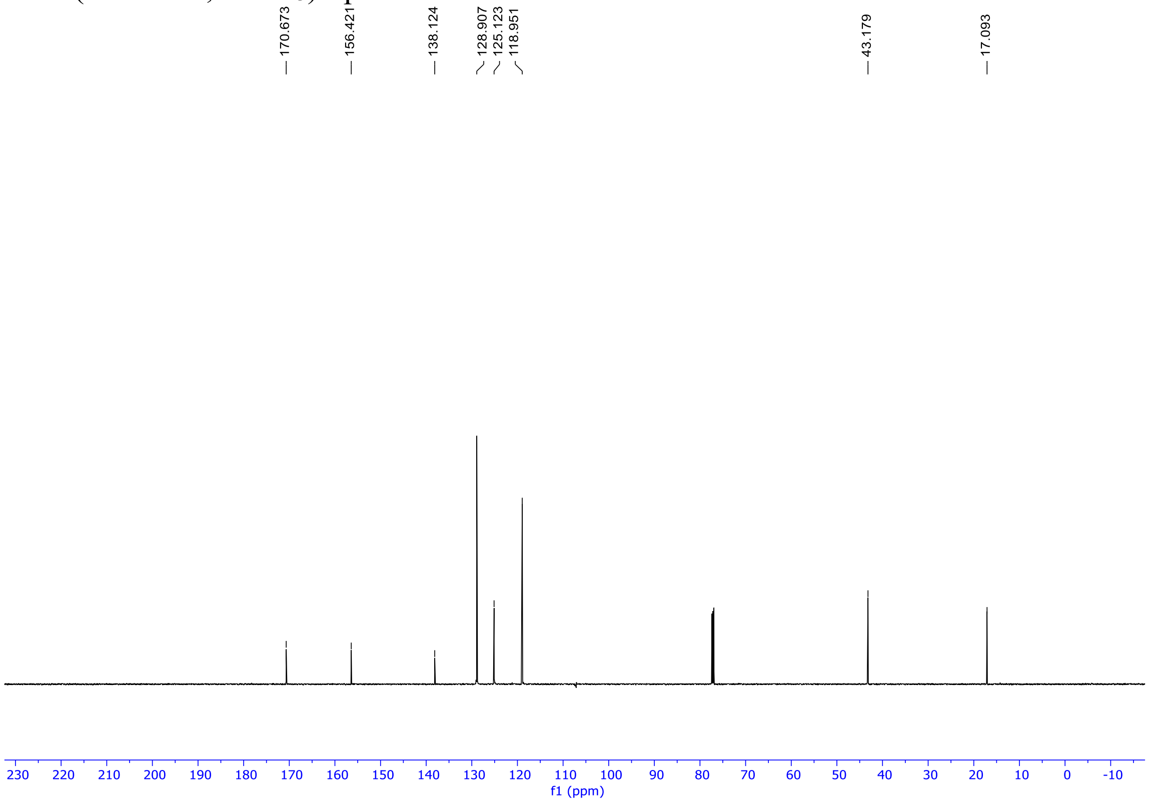

^{13}C{^{1}H} NMR (151 MHz, CDCl$_3$) Spectrum of **4b**

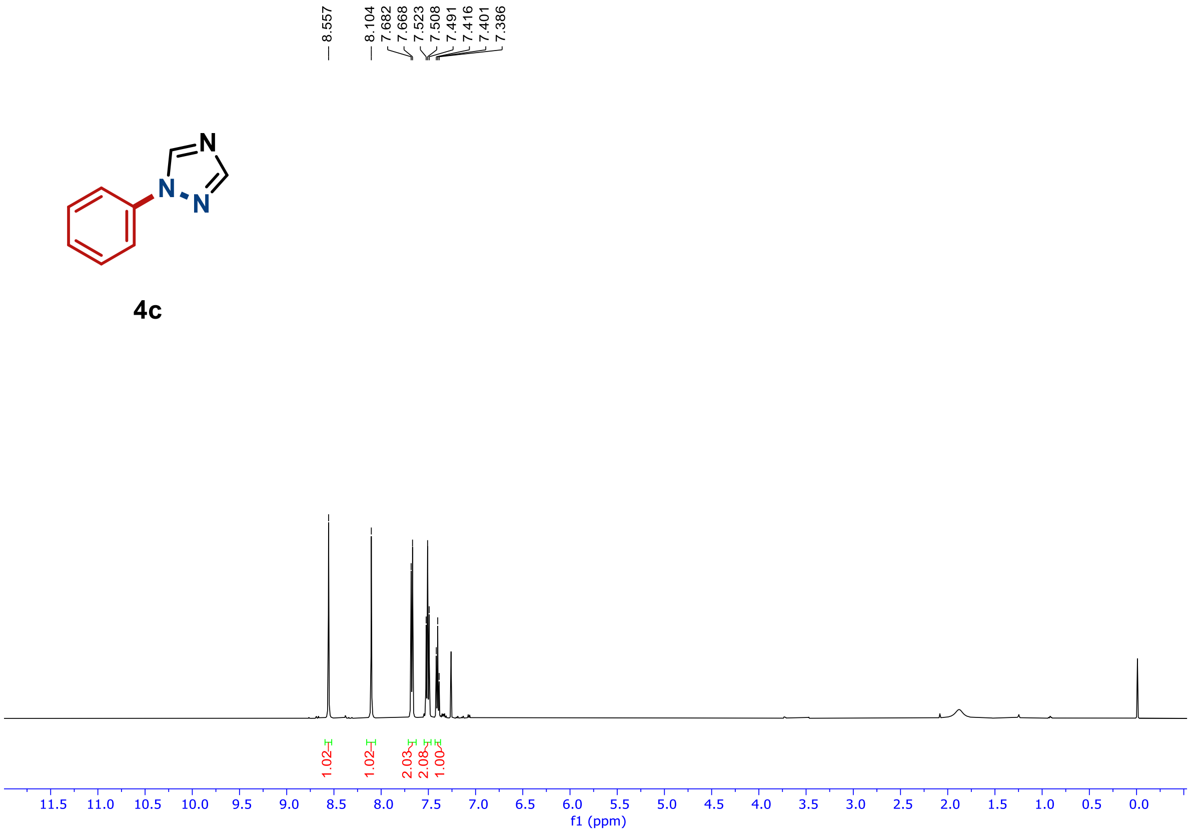

4c

^{1}H NMR (500 MHz, $CDCl_3$) Spectrum of **4c**

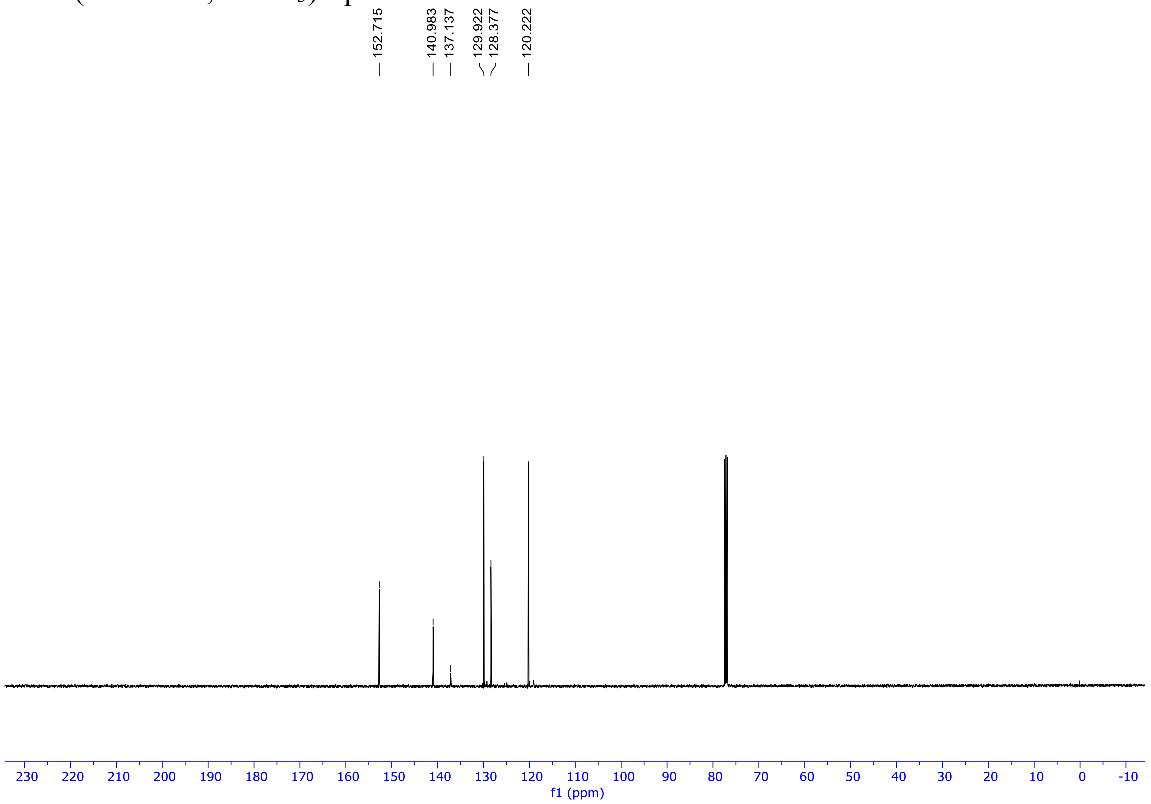

^{13}C{^{1}H} NMR (125 MHz, $CDCl_3$) Spectrum of **4c**

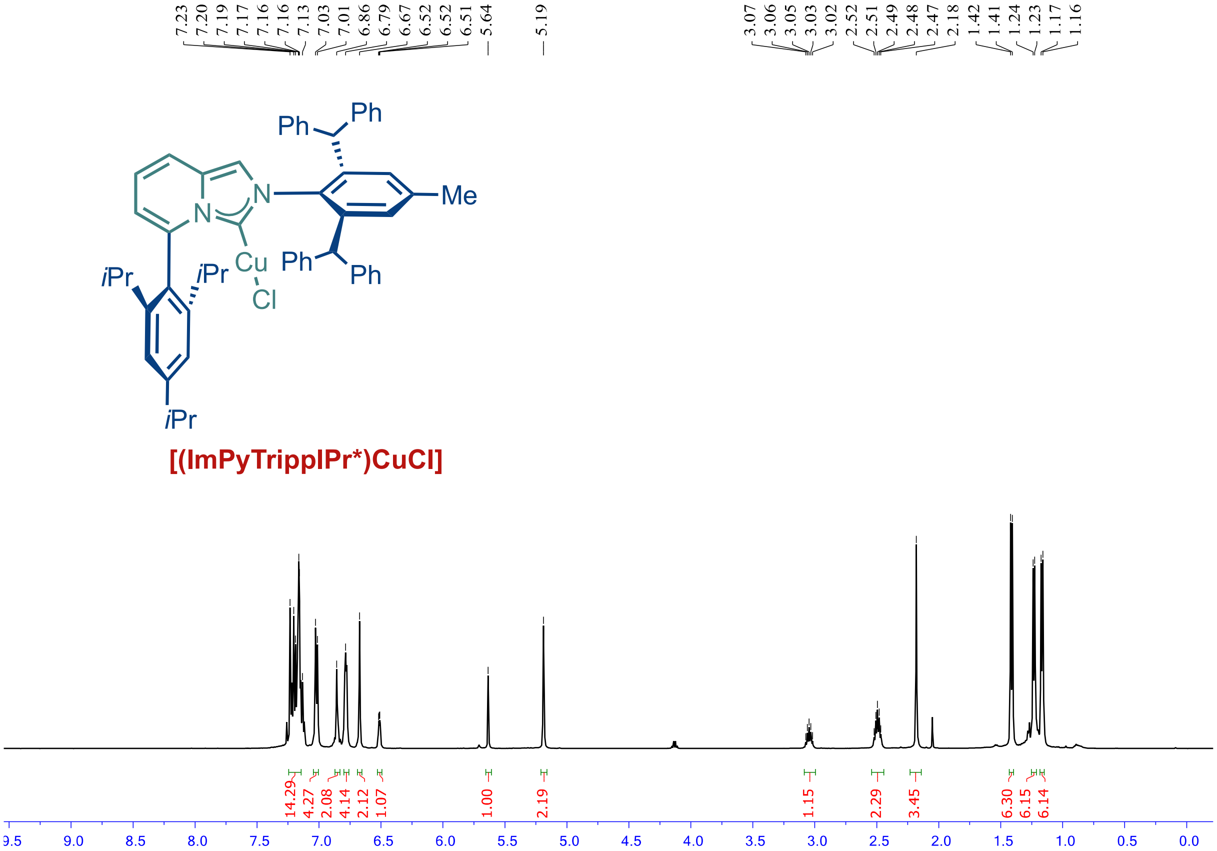

^{1}H NMR (500 MHz, CDCl$_3$) Spectrum of **[(ImPyTrippIPr*)CuCl]**

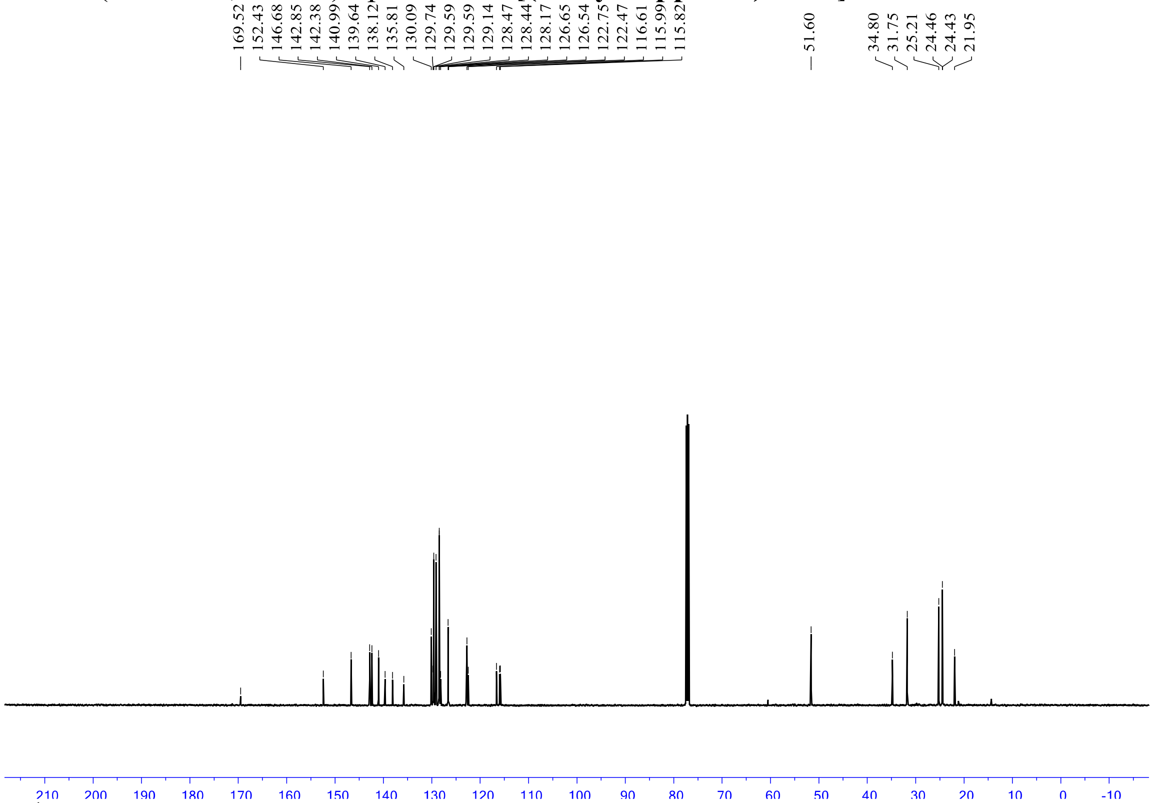

^{13}C{^{1}H} NMR (125 MHz, CDCl$_3$) Spectrum of **[(ImPyTrippIPr*)CuCl]**

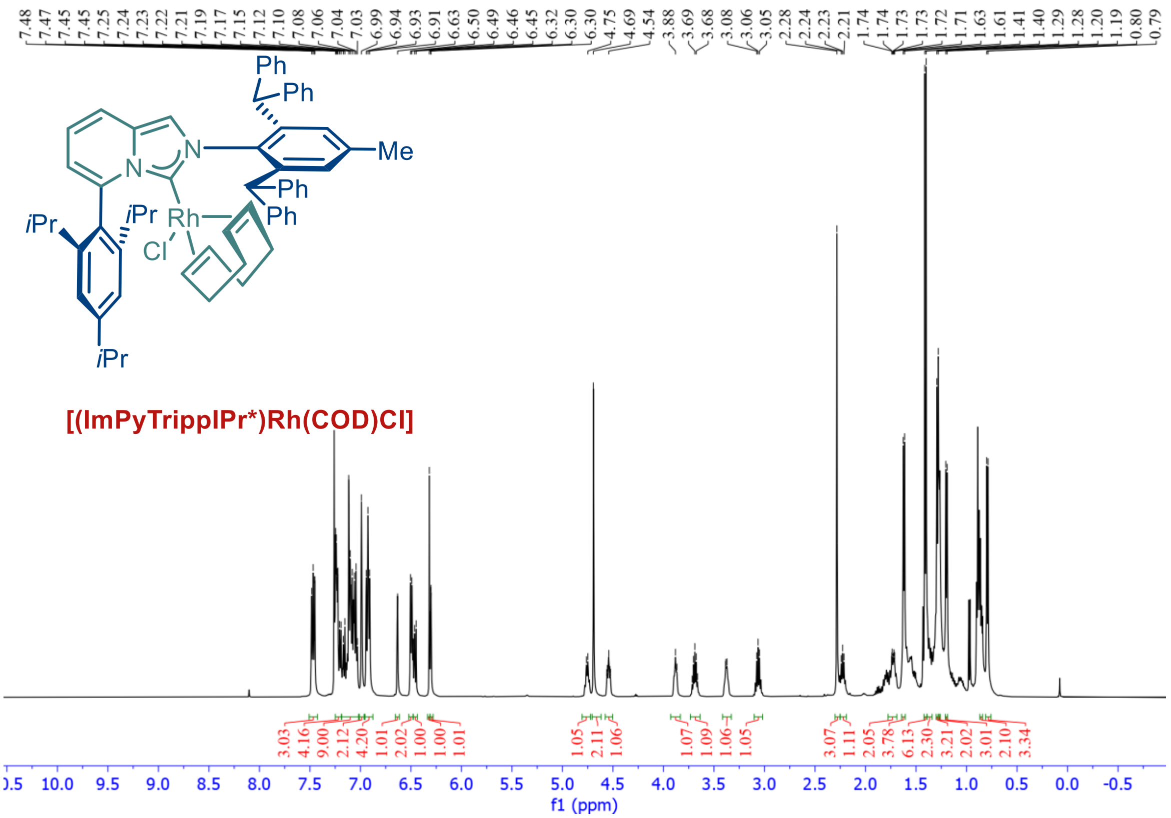

^{1}H NMR (500 MHz, $CDCl_3$) Spectrum of **[(ImPyTrippIPr*)Rh(COD)Cl]**

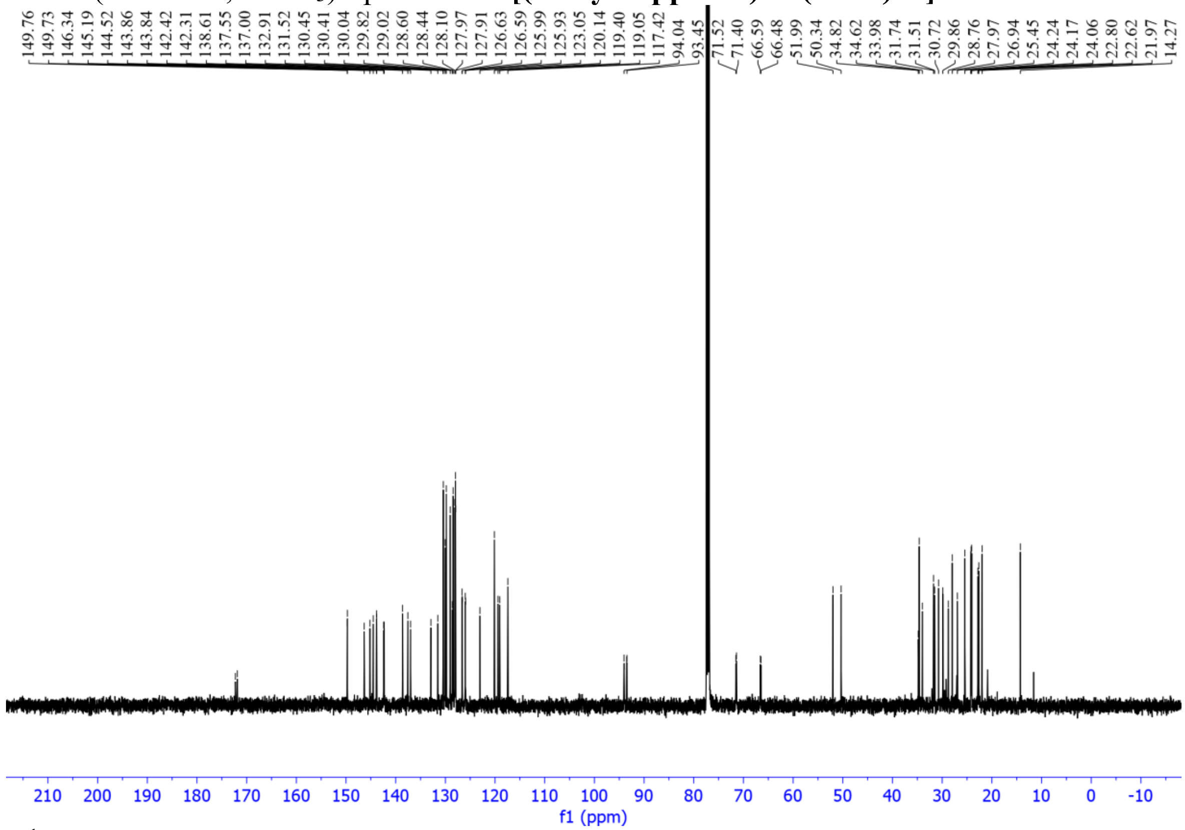

^{13}C{^{1}H} NMR (125 MHz, $CDCl_3$) Spectrum of **[(ImPyTrippIPr*)Rh(COD)Cl]**

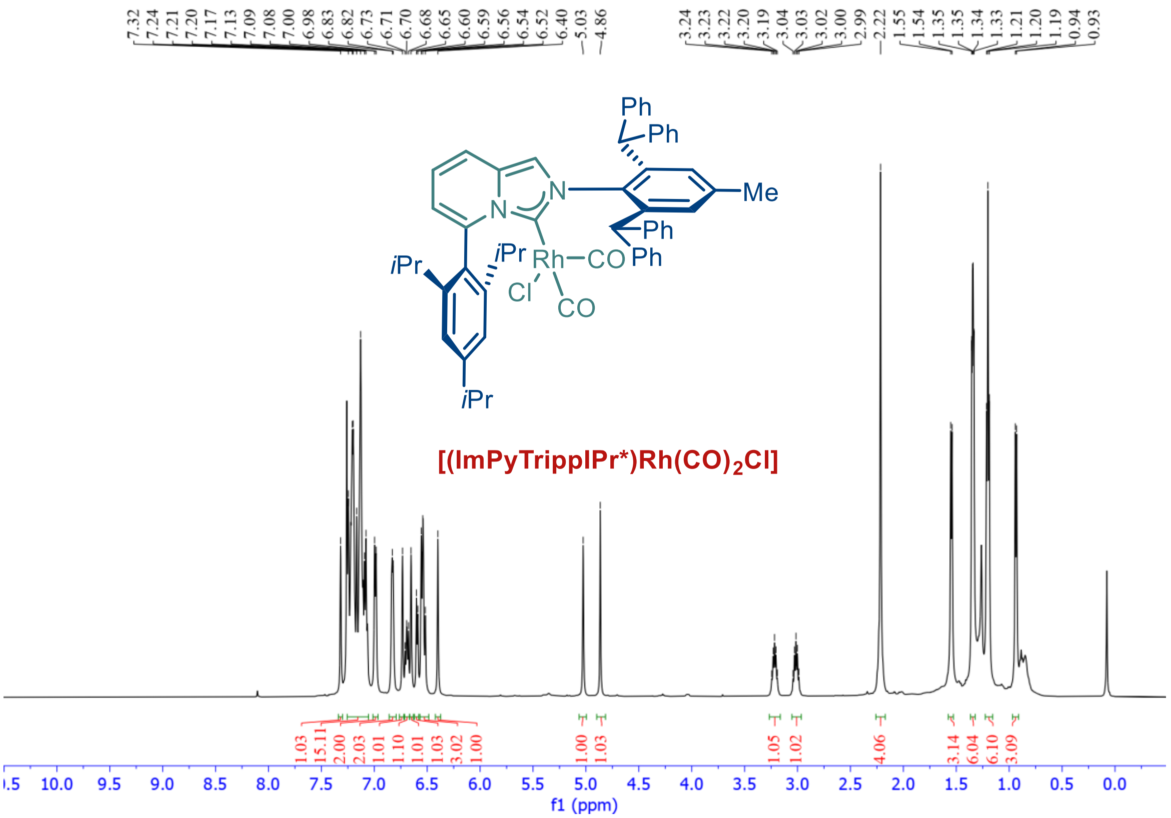

^{1}H NMR (500 MHz, CDCl$_3$) Spectrum of **[(ImPyTrippIPr*)Rh(CO)$_2$Cl]**

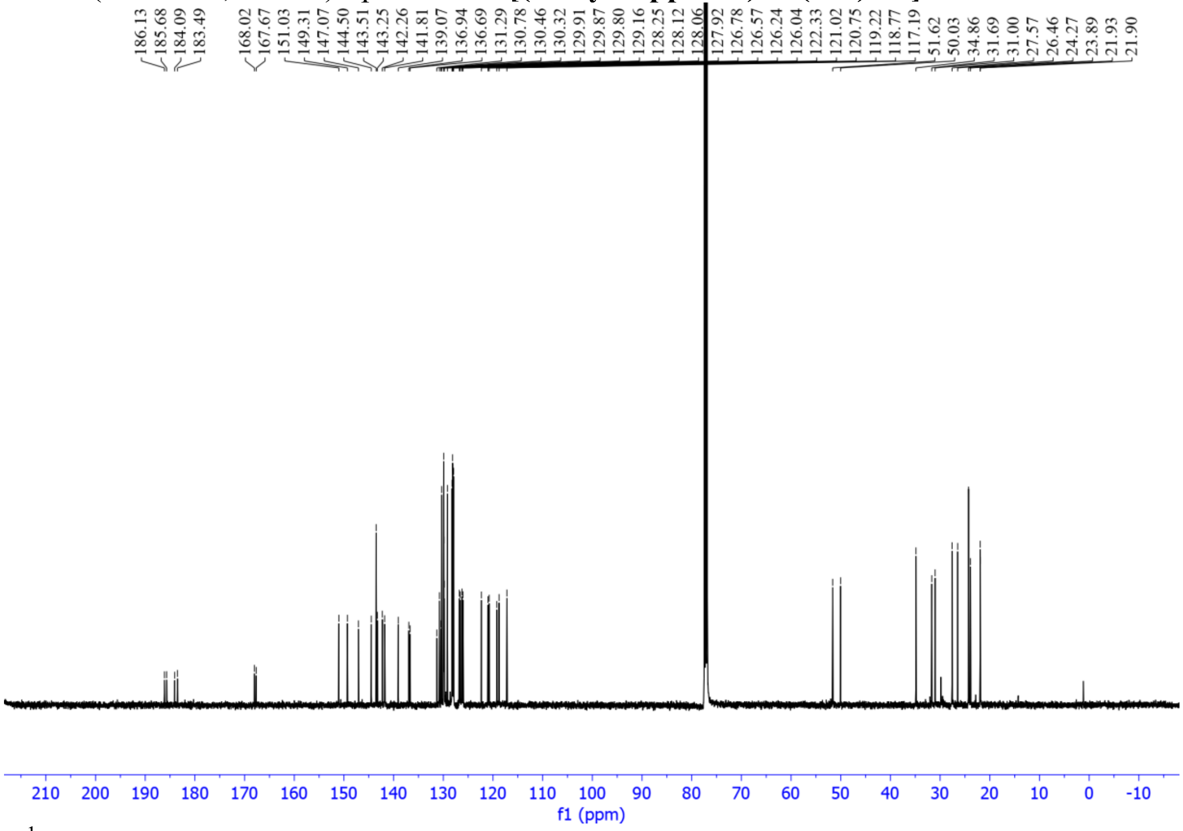

^{13}C{^{1}H} NMR (125 MHz, CDCl$_3$) Spectrum of **[(ImPyTrippIPr*)Rh(CO)$_2$Cl]**

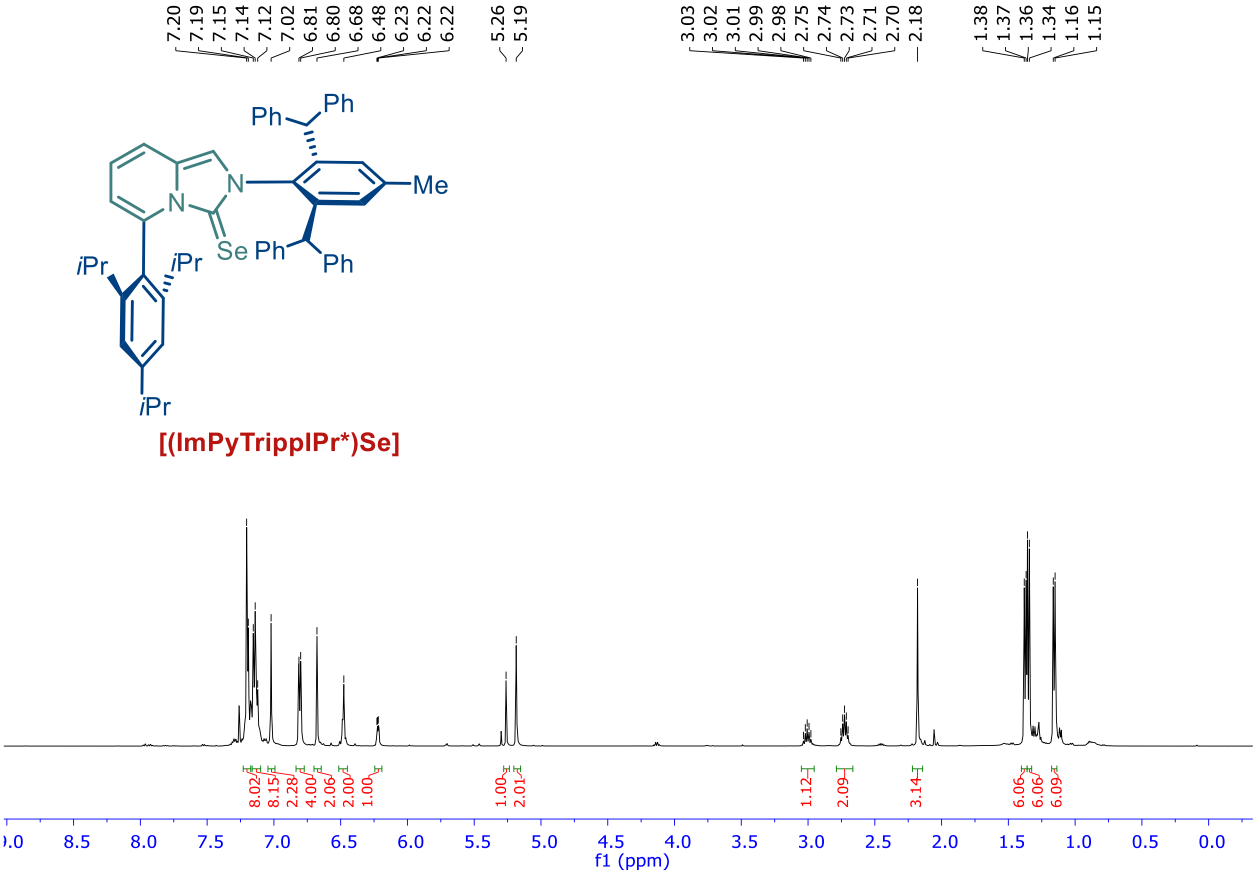

^{1}H NMR (500 MHz, CDCl3) Spectrum of **[(ImPyTrippIPr*)Se]**

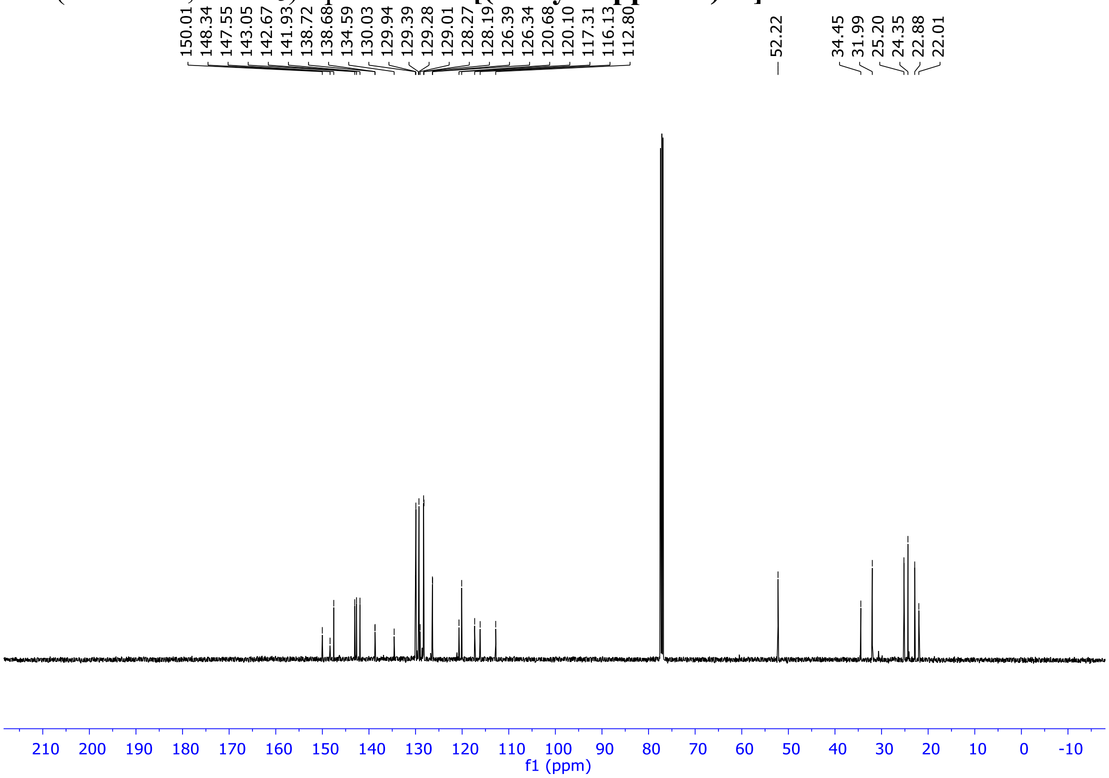

^{13}C{^{1}H} NMR (125 MHz, CDCl3) Spectrum of **[(ImPyTrippIPr*)Se]**

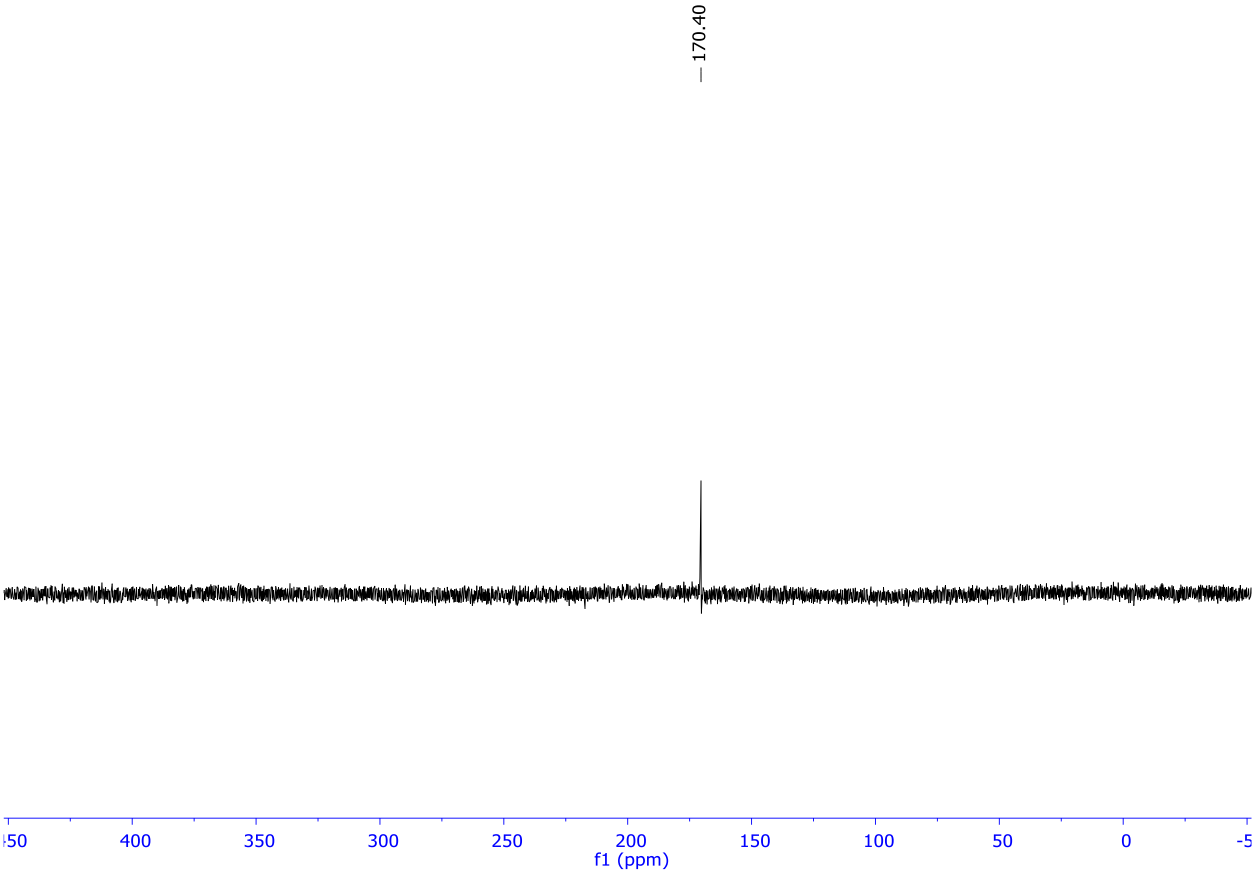

^{77}Se NMR (95 MHz, $CDCl_3$) Spectrum of **[(ImPyTrippIPr*)Se]**
